# ZFP36L2 orchestrates stress-adaptive plasticity in regeneration and cancer

**DOI:** 10.1038/s41586-026-10890-0

**Published:** 2026-08-05

**Authors:** Qingwen Jiang, Manisha S. Raghavan, Aileen M. Rodriguez, Morgan Lallo, Cyrus L. Tam, Saskia Hartner, Britney Forsyth, Ahmed Mahmoud, Fabian Zincke, Huiyong Zhao, Andrew Moorman, Sasha Balkaran, Kathleen Luckett, Jura Pintar, Yevgeniy Romin, Eric Chan, Anthony Santella, Bernadette Mödl, Farheen Shah, Ilyes Baali, Michael G. Kharas, Elisa de Stanchina, Nil Urganci, Jinru Shia, Dana Pe’er, Francisco Sanchez-Vega, Richard Koche, Quaid Morris, Joseph M. Chan, Karuna Ganesh

**Affiliations:** 1 Molecular Pharmacology Program, Sloan Kettering Institute, New York, NY USA; 2 Gerstner Sloan Kettering Graduate School of Biomedical Sciences, New York, NY USA; 3 Human Oncology and Pathogenesis Program, Memorial Sloan Kettering Cancer Center, New York, NY USA; 4 Computational and Systems Biology Program, Sloan Kettering Institute, Memorial Sloan Kettering Cancer Center, New York, NY USA; 5 Tri-Institutional PhD Program in Computational Biology and Medicine, Memorial Sloan Kettering Cancer Center, New York, NY USA; 6 Pharmacology Graduate Program, Sloan Kettering Institute, Memorial Sloan Kettering Cancer Center, New York, NY USA; 7 Antitumor Assessment Core Facility, Memorial Sloan Kettering Cancer Center, New York, NY USA; 8 Tri-Institutional MD–PhD Program, New York, NY USA; 9 Molecular Cytology Core Facility, Memorial Sloan Kettering Cancer Center, New York, NY USA; 10 Department of Epidemiology and Biostatistics, Memorial Sloan Kettering Cancer Center, New York, NY USA; 11 Department of Pathology, Memorial Sloan Kettering Cancer Center, New York, NY USA; 12 Howard Hughes Medical Institute, Chevy Chase, MD USA; 13 Department of Surgery, Memorial Sloan Kettering Cancer Center, New York, NY USA; 14 Center for Epigenetics Research, Memorial Sloan Kettering Cancer Center, New York, NY USA; 15 Department of Medicine, Memorial Sloan Kettering Cancer Center, New York, NY USA

**Keywords:** Metastasis, Intestinal stem cells, Regeneration, Stem-cell differentiation, Cancer stem cells

## Abstract

Phenotypic plasticity is a hallmark of cancer^1^; however the molecular switches required for cell-fate reprogramming are poorly understood. During intestinal wound-healing and colorectal cancer (CRC) metastasis, differentiated cells can dynamically dedifferentiate into an intestinal stem cell (ISC) state to drive epithelial regeneration and metastatic outgrowth^2–10^. Here we show that the RNA-binding protein ZFP36L2, which is mutated in 5–10% of CRC^11–15^, is a pivotal stress-responsive orchestrator of dynamic dedifferentiation. In mouse colon regeneration models, ZFP36L2 ablation inhibits dedifferentiation, ISC gene expression and function and impairs intestinal regeneration. In human CRC, loss of ZFP36L2 function abrogates metastatic seeding and the outgrowth of LGR5^+^ canonical metastases while promoting lineage plasticity and non-canonical differentiation into heterogeneous cell states. Mechanistically, ZFP36L2 binds to stress-associated mRNAs that contain AU-rich 3′ untranslated regions, which induces the formation of dynamic biomolecular condensates associated with mRNA degradation and termination of the stress response. Together, these data show that ZFP36L2 acts as an important molecular switch that couples stress sensing with phenotypic plasticity. This in turn drives cellular dedifferentiation essential for re-establishing the ISC state during wound healing and metastasis. In ZFP36L2-deficient CRC, the inability to re-enter the LGR5^+^ state during metastatic outgrowth promotes non-canonical lineage plasticity, which is associated with poor clinical outcomes.

## Abstract (web summary)

The RNA-binding protein ZFP36L2 mediates stress-adaptive plasticity in intestinal regeneration and colorectal cancer metastasis.

## Main

**Fig. 1:**
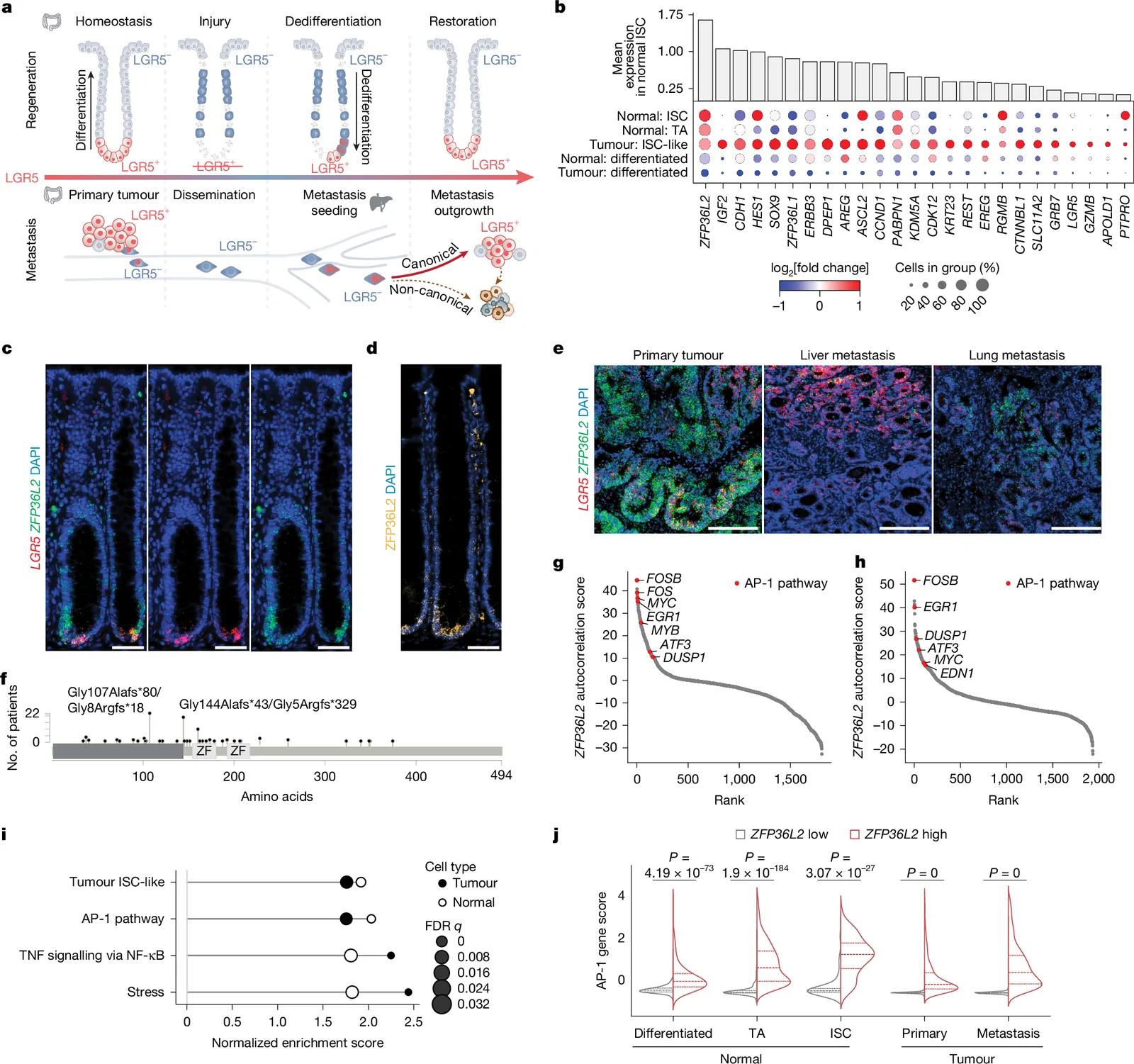
*ZFP36L2* is expressed by ISCs and is associated with the AP-1 gene program. **a**, Schematic of cell-state plasticity and dynamics during intestinal regeneration (top) and CRC (bottom). **b**, Dot plot of tumour ISC-like module^10^ gene expression in normal colon (Normal) and CRC (Tumour) epithelial cells from 25 patients. The dot size indicates the per cent cells (rows) expressing each gene (columns). Colour scale indicates the log_2_ fold change in average gene expression. Bar plots show the mean expression of each gene across normal colon ISCs. Genes meeting false discovery rate (FDR) < 0.05 using the Wilcoxon rank-sum test (ISC versus all other epithelial subtypes) are shown. **c**,**d**, Representative *LGR5* and *ZFP36L2* RNA FISH (**c**) and ZFP36L2 IF (**d**) images of human normal colon crypts. **e**, Representative *LGR5* and *ZFP36L2* RNA FISH images of human primary CRC tumours (*n* = 2), liver metastases (*n* = 2) and lung metastases (*n* = 2) from 2 donors. **f**, Lollipop plot showing *ZFP36L2* mutation frequency in the TCGA^63^, DFCI^64^ and MSKCC whole-exome sequencing CRC cohorts. *n* = 96 tumours. **g**,**h**, *ZFP36L2* autocorrelated gene programs in normal colon (**g**) and CRC epithelial cells (**h**). Red, top AP-1 family genes^65^. *n* = 12,955 normal colon (24 donors) and 25,317 CRC cells (25 patients). FDR < 0.05. **i**, GSEA of genes (Supplementary Table 1b–f) ranked by positive *ZFP36L2* autocorrelation (Methods), which revealed conserved pathways enriched in normal (white dots) and tumour (black dots) epithelial cells. *n* = 12,955 normal colon (24 donors) and 25,317 CRC cells (25 patients). FDR < 0.05. **j**, Violin plots showing AP-1 pathway gene expression in *ZFP36L2*-high and *ZFP36L2*-low populations (top and bottom 10 percentile expression). *n* = 1,806 differentiated, *n* = 1,326 TA and *n* = 230 ISC cells from normal colons (24 donors) and 2,622 primary CRC and 2,444 CRC metastasis cells (25 patients). Two-sided Mann–Whitney *U-*test. Scale bars, 50 µm (**c**,**d**) or 200 µm (**e**).

The intestinal epithelium is characterized by a hierarchy of cell states that balance self-renewal and differentiation to maintain homeostasis^2^. Crypt base ISCs, which are marked by the expression of genes such as *LGR5*, drive the continuous production of short-lived, specialized differentiated daughter cells that are eventually shed into the intestinal lumen^16^. However, when LGR5^+^ ISCs are lost during injury, the epithelium displays substantial plasticity. A broad range of differentiated cells have the capacity to undergo injury-induced reprogramming. Initially, cells enter into a transient regenerative state with fetal-like properties and then they dedifferentiate into a LGR5^+^ ISC state to repopulate the crypt base and restore homeostasis^2,3,17^ (Fig. 1a). Although the transcription factor ASCL2 is necessary for this process^18^, little is known about injury-induced triggers of dedifferentiation. In CRC, the capacity of the intestinal epithelium to undergo stress-induced reprogramming contributes to tumour progression, metastasis and therapy resistance^19^. Primary tumours largely adopt LGR5^+^ ISC states either through the acquisition of oncogenic driver mutations in crypt base ISCs or through the dedifferentiation of mutated differentiated cells into tumour-permissive ISC-like states^20–23^. Cells at the primary tumour-invasion front that detach from the tumour mass enter into LGR5^–^ metastasis-initiating states marked by increased expression of injury-associated regenerative and fetal markers (for example, *L1CAM*, *PROX1*, *TROP2* and *ANXA1*)^7,10,24^. These cells also exhibit increased expression of epithelial differentiation markers (for example, *EMP1*)^9^, and are associated with increased activity of the AP-1 family of stress-response transcription factors^25,26^ (henceforth termed an ‘injury–repair’ state). After extravasation in distant organs, differentiated LGR5^–^ metastasis-initiating cells can dedifferentiate into canonical LGR5^+^ ISC states to seed metastasis and regenerate tumours, a process that mirrors the restoration of homeostasis during intestinal regeneration^8–10,26–28^ (Fig. 1a). Alternatively, CRC metastases can undergo lineage plasticity into non-canonical squamous-like and neuroendocrine-like states not observed during regeneration in the intestinal niche^10,26^. Such non-canonical states are common among patients and are associated with adverse clinical outcomes compared with patients with tumours that primarily express canonical states. The mechanisms that underpin such cell-state reprogramming and cell-fate decision-making are largely unknown. To gain insight, we sought to identify the molecular switch that controls stress-induced dedifferentiation into a LGR5^+^ ISC state, a conserved process that is essential for both epithelial regeneration and canonical metastases.

## ZFP36L2 co-expression with stress programs

**Extended Data Fig. 1:**
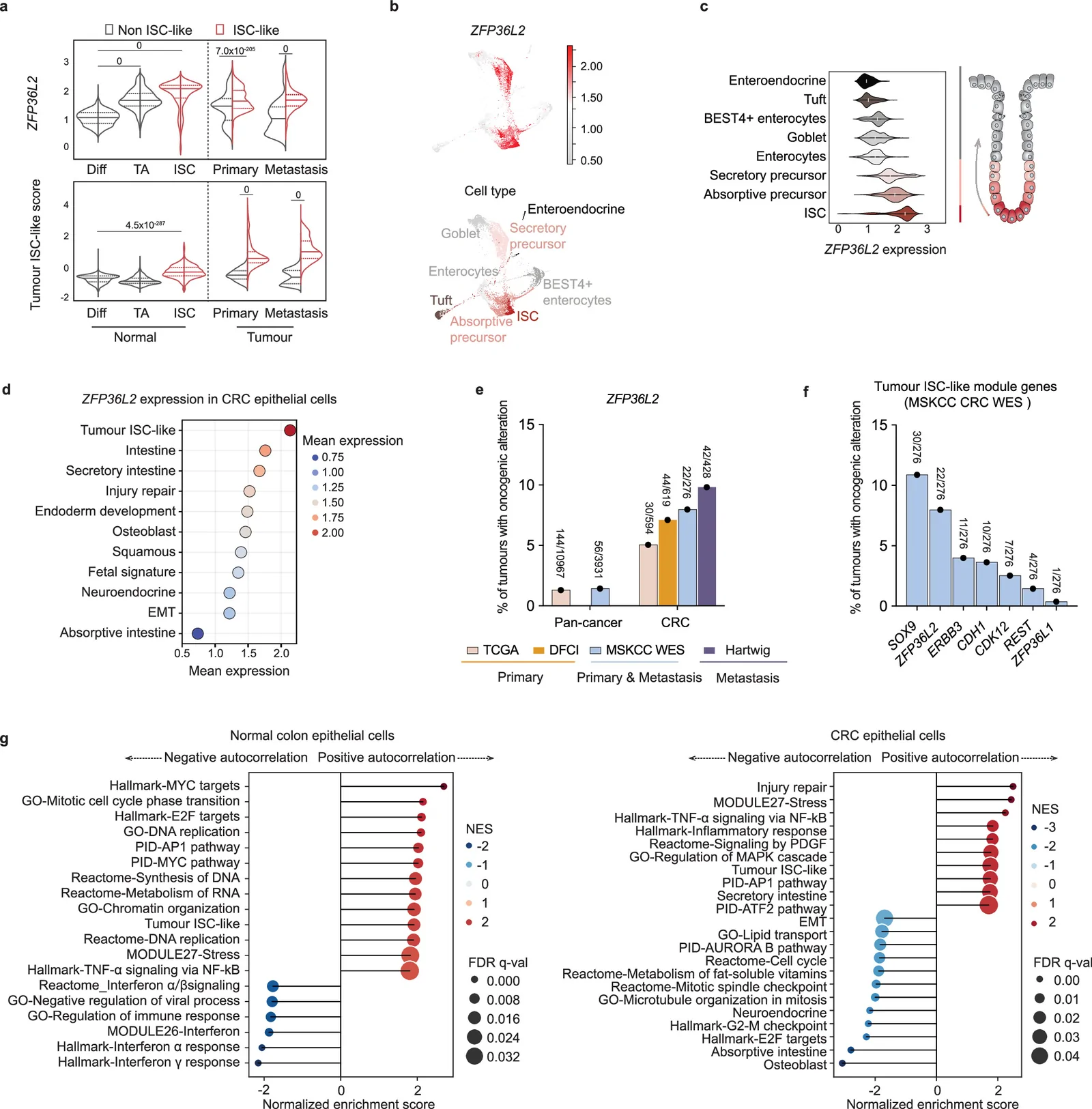
*ZFP36L2*-associated gene module analysis in normal and tumour epithelial cells. **a**, Violin plots showing *ZFP36L2* and tumour ISC-like module gene expression in differentiated (Diff), transit-amplifying (TA) and intestinal stem cells (ISC) from normal colon crypts, and in ISC-like versus non-ISC-like populations in CRC tumours, based on patient scRNA-seq data. n = 5,187 differentiated, 6,623 transit amplifying (TA), 1,145 intestinal stem cell (ISC) cells from normal colons from 24 donors and 13,102 primary CRC and 12,215 CRC metastasis cells from 25 patients. Two-sided. Mann-Whitney *U* test. **b**–**c**, Force-directed layout (**b**) and violin plots (**c, left**) depicting *ZFP36L2* expression (top) across intestinal epithelial cells (bottom): intestinal stem cells (ISC, dark red), transit-amplifying secretory and absorptive precursors (light red) and differentiated epithelial cells (grey, black). n = 12,955 cells from 24 normal human colon samples. (**c, right)** Schematic of the intestinal crypt illustrating differentiation trajectories. **d**, Mean of imputed *ZFP36L2* expression across primary and metastatic CRC cells expressing canonical and non-canonical gene programs, based on Hotspot analysis^10^, n = 26,145 cells from 28 CRC patients. **e**, Frequency of oncogenic *ZFP36L2* mutations across the DFCI (44/619 tumours, 7.1%)^64^, TCGA (30/594 tumours, 5.1%)^63^), MSKCC whole exome sequencing (WES) recapture (22/276 tumours, 8.0%), and Hartwig (42/428 tumours, 9.8%)^57^ cohorts, annotated using OncoKB^68^. **f**, Frequency of oncogenic mutations in tumour ISC-like module genes in the MSKCC CRC whole exome sequencing (WES) recapture cohort, annotated using OncoKB^68^. All genes with at least one oncogenic mutation detected are shown. **g**, Gene set enrichment analysis (GSEA) of genes ranked by autocorrelation with *ZFP36L2*, identifying pathways enriched in normal colon epithelial cells (left) and CRC epithelial cells (right) FDR < 0.05. Full genesets and references are provided in Supplementary Table 1b.

To identify mediators of cell-state switching, we focused on genes co-expressed in ISC-like cells in a cohort of same-patient trios of normal, primary and metastatic CRC tissue samples. Genes were identified through Hotspot gene module analyses of single-cell RNA sequencing (scRNA-seq) data from fresh tissue^10^, and we termed them ‘tumour ISC-like’. *ZFP36L2* (also known as *TIS11D*, *ERF2* and *BRF2*) stood out as the gene most highly expressed in ISC-like cells. * ZFP36L2* expression progressively decreased with differentiation in both normal intestinal crypts and in CRC, including canonical intestinal and non-canonical lineages (Fig. 1b–e and Extended Data Fig. 1a–d). Whole-exome and whole-genome sequencing studies^11–15^ have identified recurrent *ZFP36L2* mutations in 5–10% of tumours from patients with CRC (Extended Data Fig. 1e,f). Most of these mutations are frameshift deletions with truncation of the coding region upstream of the zinc finger domain, which probably results in loss of ZFP36L2 function (Fig. 1f). ZFP36L2 is an RNA-binding protein of the ZFP36 (also known as tristetraprolin) family of CCCH-tandem zinc finger proteins. These proteins bind AU-rich elements in 3′ untranslated regions (UTRs) and recruit the RNA exosome or the CCR4–NOT complex to mediate mRNA degradation in haematopoiesis and early embryogenesis^29–32^. However, little is known about ZFP36L2 function in epithelial cells and cancer.

**Extended Data Fig. 2:**
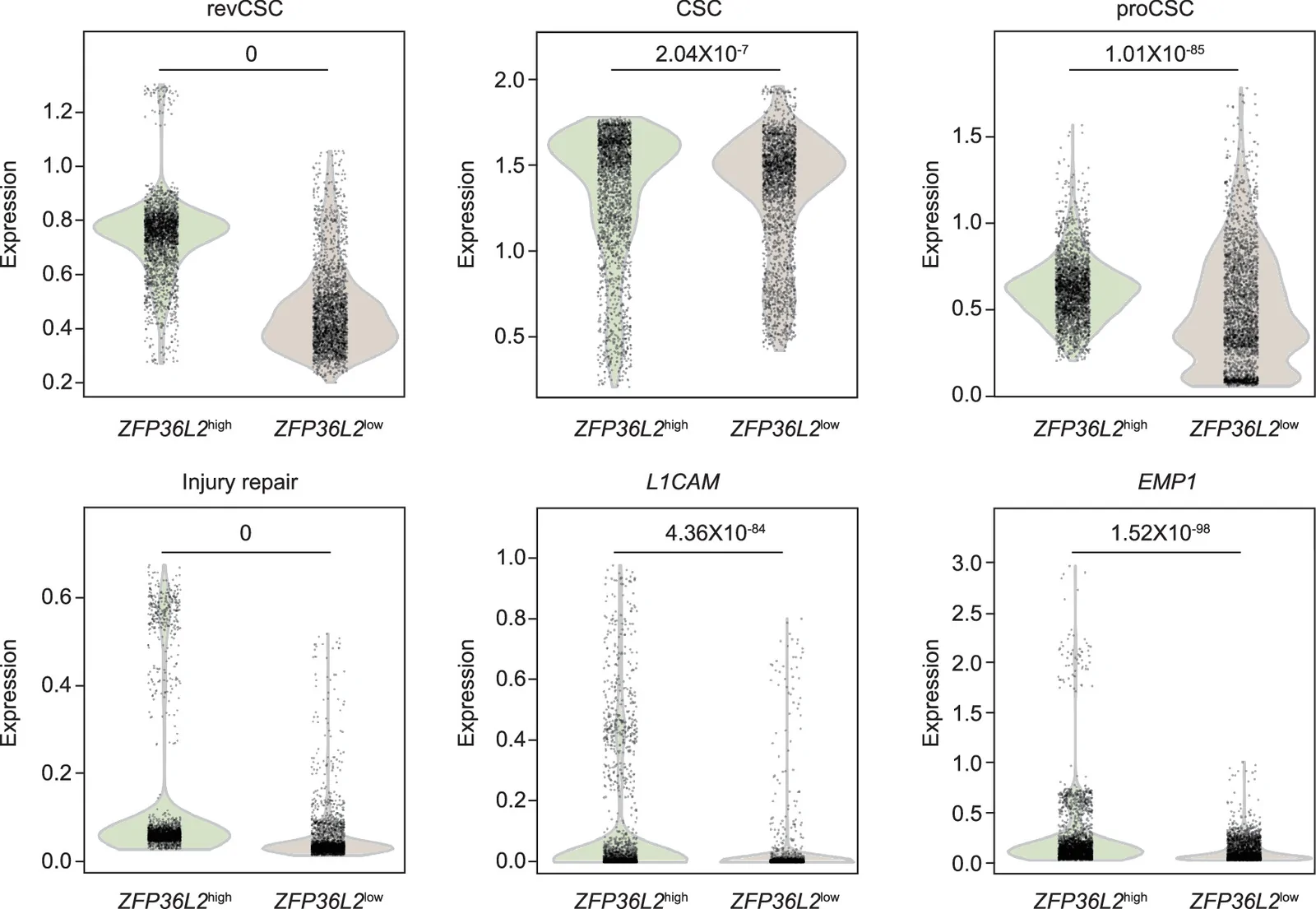
*ZFP36L2* is expressed in a subset of cells in the injury repair state. Violin plots showing distribution of gene expression of revCSC, CSC and proCSC signatures^28^, injury repair module^10^, and metastasis-initiating cell markers *L1CAM*^7^ and *EMP1*^9^ in *ZFP36L2*-high and *ZFP36L2*-low populations from tumour epithelial cells. *ZFP36L2*-high and -low cells were defined by the top and bottom 10% of *ZFP36L2* expression levels, respectively n = 2,622 primary CRC and 2,444 CRC metastasis cells from 25 patients.

To gain insight, we performed gene expression autocorrelation analysis across normal ISCs, transit-amplifying (TA) cells and tumour cells (Methods). Genes most strongly co-expressed with *ZFP36L2* in both normal colon and CRC samples included AP-1 transcription factors and other components of the AP-1 stress-response pathway (for example, *FOSB*,* FOS*,* MYC*,* EGR1*,* MYB*,* ATF3 *and* DUSP1*; Fig. 1g–j, Extended Data Fig. 1g and Supplementary Table 1c–f). Although ZFP36L2 was most broadly expressed in tumour ISC-like cells, a subset of ZFP36L2-high CRC cells displayed an injury–repair state marked by high expression of the RevCSC signature^28^, and the metastasis-initiating cell markers *L1CAM*^7^ and *EMP1*^9^ (Extended Data Fig. 2). The AP-1 transcription factor complex, formed by FOS, JUN and ATF family of heterodimers, has a central role in orchestrating stress-induced transcriptional responses during tissue regeneration^33^. AP-1 is rapidly activated in response to cytokines, growth factors or microbial or physical stressors. Subsequently, AP-1 integrates upstream signals—primarily via MAPK, JNK and ERK pathways—to drive expression programs that regulate proliferation, survival, differentiation and epithelial remodelling. ZFP36 family members are well-characterized immediate-early genes and are rapidly induced in response to such stimuli^34–37^. EGF stimulation of cells leads to the sequential induction of AP-1 followed by ZFP36L2^35^, which indicates that ZFP36L2 has a role downstream of mitogenic signalling. Together, these data suggest that ZFP36L2 might be a molecular link between AP-1-driven stress response in the injury–repair state and re-entry into the ISC state.

## ZFP36L2 mediates dedifferentiation

**Fig. 2:**
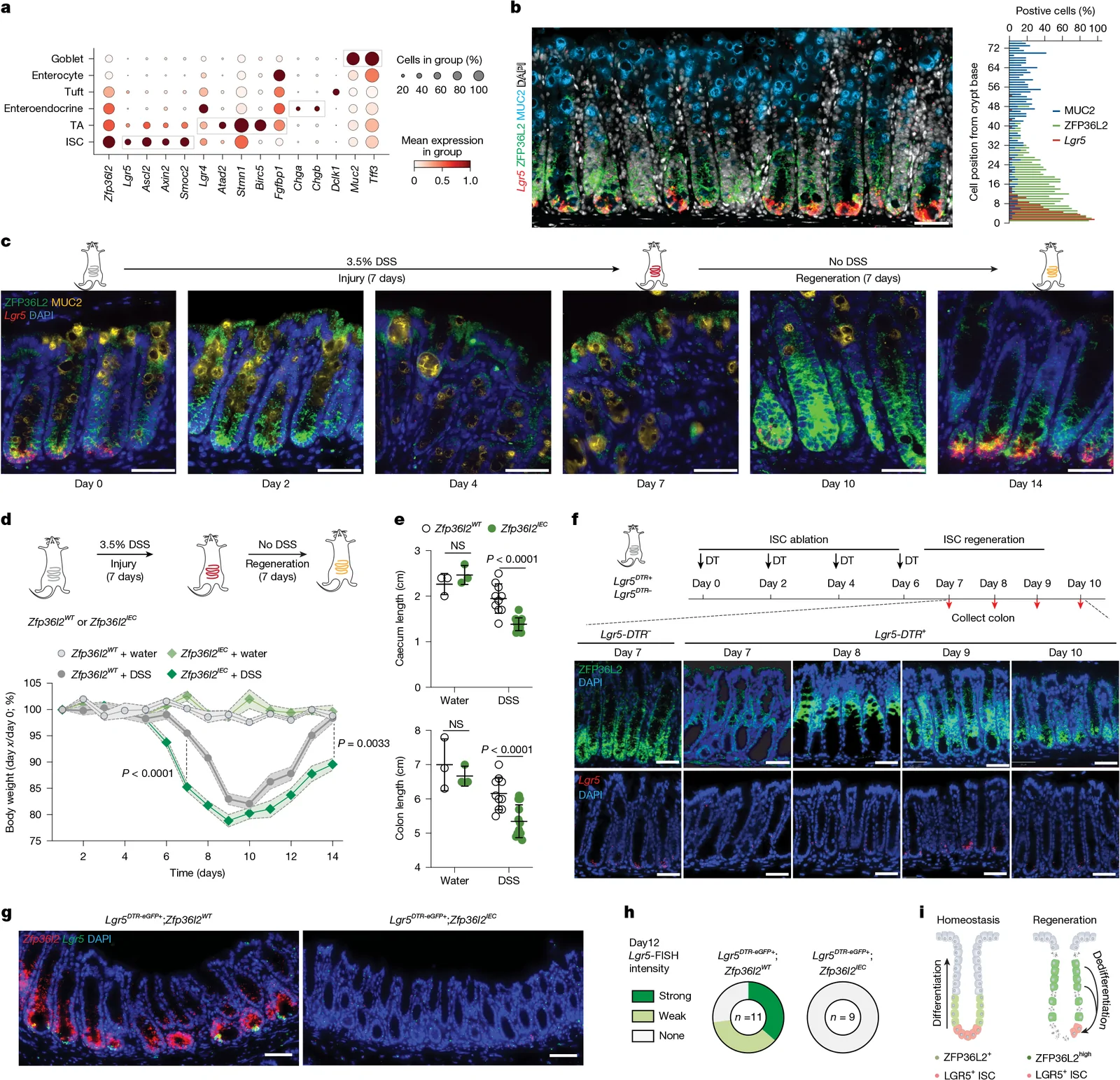
*Zfp36l2* is required for injury-induced dedifferentiation during intestinal regeneration. **a,** *Zfp36l2* expression across epithelial cells, as measured by scRNA-seq of colonic crypts (21,000 cells from 11 mice)^66^. Marker genes for each cell type are indicated by boxes. **b**, Representative images (left) and quantification (right) of *Lgr5* RNA FISH and ZFP36L2 and MUC2 IF in colon tissue from 7-week-old C57BL/6J mice based on the cell position along the crypt base (position 0) to lumen axis. *n* = 2,275 cells from 31 crypts. **c**, Representative images of *Lgr5* RNA FISH and ZFP36L2 and MUC2 IF in colon tissue collected at the indicated time points from mice treated with 3.5% DSS for 7 days followed by recovery for 7 days. *n* = 3 mice per time point. Expanded fields of view are provided in Supplementary Figs. 1 and 3. **d**, Top, schematic of the experiment. Bottom, daily body weight measurements. *n* = 5 (water) mice; *n* = 10 (wild-type (WT)) and 14 (IEC) (DSS-treated) mice. Mean (solid line; dots, individual time points) ± s.e.m. (shaded area; dashed lines, s.e.m. boundaries); two-tailed Mann–Whitney *U*-tests at day 7 and day 14. **e**, Caecum (top) and colon (bottom) lengths on day 14. *n* = 3 mice (water); *n* = 10 (WT) and 12 (IEC) (DSS-treated) mice. Mean ± s.d.; two-tailed Mann–Whitney *U-*test. NS, not significant. **f**, *Lgr5*^*DTR*^ ablation. Top, schematic of the experiment. Bottom, representative images (*n* = 2 mice) of colon sections stained by *Lgr5* RNA FISH or ZFP36L2 IF at the indicated time points. Expanded fields of view are provided in Supplementary Fig. 5. Controls: *Lgr5*^*DTR*^-negative mice treated with 4 doses of DT over 7 days. **g**,**h**, Mice (8–10 weeks old) were administered DT every 2 days for 4 doses. Mice were euthanized and colons were collected 12 days after the first DT dose. **g**, Images of mouse *Lgr5* and *Zfp36l2* mRNA FISH on day-12 Swiss-roll colon sections. **h**, The number of mice showing regeneration of the *Lgr5* FISH signal. **i**, Schematic of the expression and function of ZFP36L2 in injury-induced dedifferentiation to drive regeneration in the mouse colon. Scale bars, 50 µm (**b**,**c**,**f**,**g**). Source data

We first explored the relationship between ZFP36L2, the stress response and the ISC state. As in humans, mouse *Zfp36l2* mRNA (Fig. 2a) and ZFP36L2 protein (Fig. 2b) were most highly expressed in crypt base ISCs, with expression declining with differentiation. Colitis induced by dextran sodium sulfate (DSS) leads to destruction of intestinal epithelial cells (IECs), followed by dedifferentiation of surviving differentiated progenitors into LGR5^+^ ISCs and restoration of homeostasis once DSS is withdrawn^38^. ZFP36L2 expression increased by day 2 of DSS administration, concurrent with LGR5 loss, whereas both ZFP36L2 and LGR5 expression were downregulated at the peak of injury by day 7 (Fig. 2c). During regeneration, ZFP36L2 was re-expressed first in the mid-crypt (TA compartment) and crypt base (ISC compartment) by day 10, preceding restoration of crypt base *Lgr5* expression by day 14.

**Extended Data Fig. 3:**
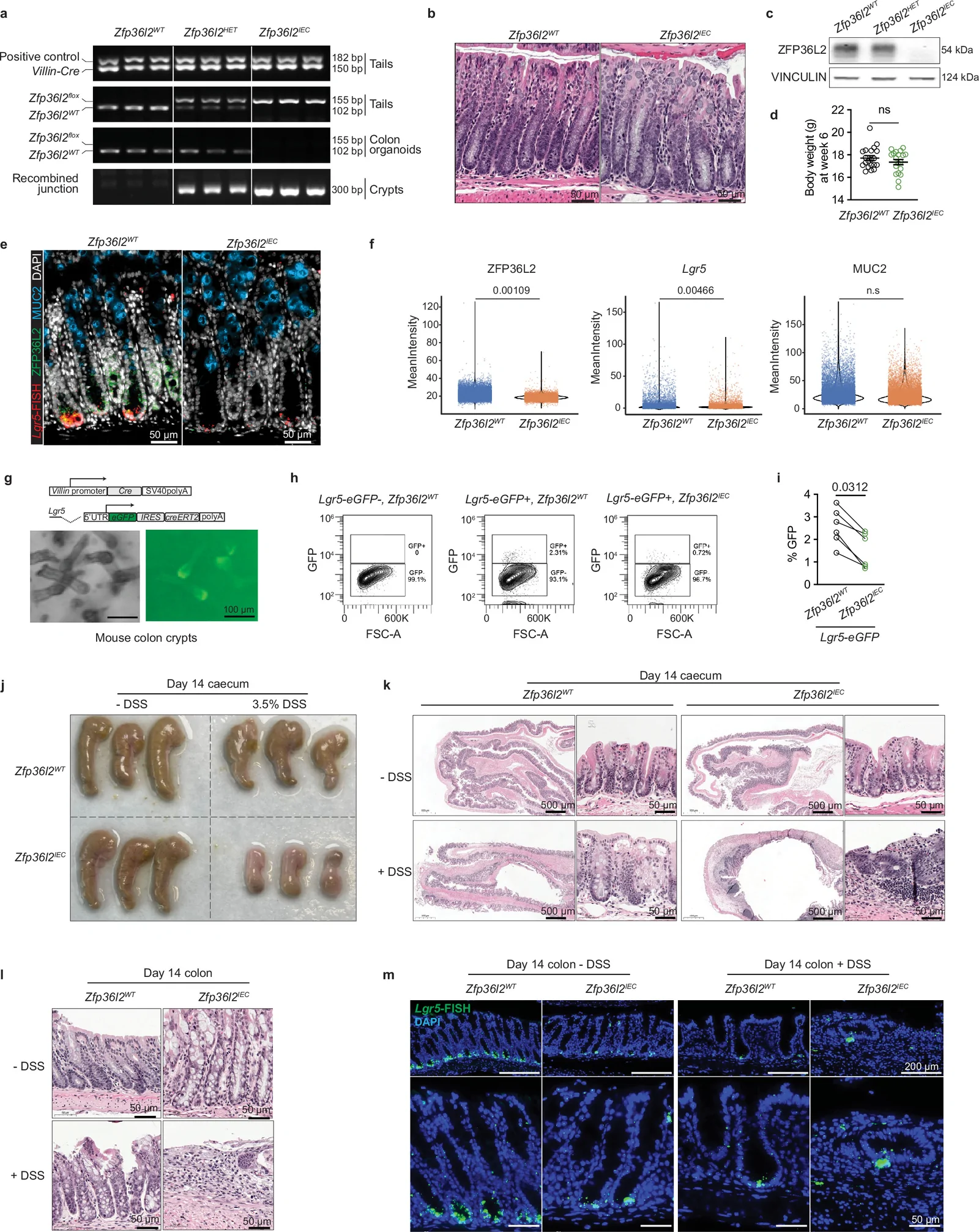
Experimental design and characterization of intestinal epithelial *Zfp36l2* knockout mice and their response to injury. a, Genotyping confirming efficient Villin-Cre-mediated deletion of *Zfp36l2* in colon-derived organoids from *Zfp36l2*^*WT*^, *Zfp36l2*^*HET*^, and *Zfp36l2*^*IEC*^ mice. 102 bp band: wild-type allele. 155 bp band: transgenic floxed allele which is excised upon successful recombination. ~300 bp band: loxP-flanked junction recombined by Villin-Cre in colon crypts. **b**, Representative images of hematoxylin and eosin (H&E) staining of colon tissue from 6-week-old *Zfp36l2*^*WT*^ and *Zfp36l2*^*IEC*^ mice. n = 3 mice per condition. **c**, Western blot showing efficient loss of ZFP36L2 protein in colon organoids from *Zfp36l2*^*IEC*^ mice compared to *Zfp36l2*^*WT*^ and *Zfp36l2*^*HET*^ controls. **d**, Body weight comparison of *Zfp36l2*^*WT*^ and *Zfp36l2*^*IEC*^ mice at 6 weeks of age prior to DSS treatment. n = 20 mice per group. Data are mean ± s.e.m. with all dots; two-tailed Mann-Whitney *U* test. ns: non-significant. **e**, Representative images of *Lgr5-*FISH, ZFP36L2-IF and MUC2-IF of colon tissue from 6-week-old *Zfp36l2*^*WT*^ and *Zfp36l2*^*IEC*^ mice. n = 3 mice per condition. Expanded field of view in Supplementary Figs. 2,4. f, Quantification of (**e**) ZFP36L2 and MUC2 mean fluorescence intensity by immunofluorescence, and *Lgr5* RNA abundance by RNA FISH, in colonic crypt cells from Swiss rolls of *Zfp36l2*^***WT***^ and *Zfp36l2*^***IEC***^ mice. Violin plots with individual cells overlaid as dots are shown. Each dot represents one cell. Swiss roll regions containing U-shaped crypts were quantified from 3 mice per group. Two-tailed Mann–Whitney *U* test. **g**–**i**, *Vil* ^*cre+*^/*Lgr5*^*eGFP-IRES-creERT2*^ intestinal stem cell reporter mice. **g**, (Top) Schematic and representative (bottom left) brightfield and (bottom right) green fluorescence images of crypts from *Vil* ^*cre+*^/*Lgr5*^*eGFP-IRES-creERT2*^ reporter mice. **h**, Flow cytometric analysis of GFP-positive cells in intestinal crypts isolated from *Vil*^*cre+*^*/Lgr5*^*eGFP-IRES-creERT2*^^*−*^*/Zfp36l2*^*WT*^, *Vil*^*cre+*^*/Lgr5*^*eGFP-IRES-creERT2*^^*+*^*/Zfp36l2*^*WT*^, *Vil*^*cre +*^*/Lgr5*^*eGFP-IRES-creERT2*^^*+*^*/ Zfp36l2*^*IEC*^ mice at age of 6-8 weeks. Representative of n = 6 mice/group. **i**, Percentage GFP^+^ crypt epithelial cells from *Vil*^*cre +*^*/Lgr5*^*eGFP-IRES-creERT2*^^*+*^*/Zfp36l2*^*WT*^, *Vil*^*cre+*^*/Lgr5*^*eGFP-IRES-creERT2*^^*+*^*/Zfp36l2*^*IEC*^ mice from flow cytometric analysis across two experimental batches. Littermates from each breeding and the same batch of experiment are connected by lines. n = 6, Wilcoxon matched-pairs signed rank test, two-sided. **j**, Representative images of caecum (n = 3 for untreated and n = 10 for treated) from *Zfp36l2*^*WT*^ and *Zfp36l2*^*IEC*^ mice treated with or without DSS for 7 days and allowed to recover for 7 days. Animals were euthanized and organs were harvested for fixation and imaging; images were taken on day 14. **k-l**, Representative H&E-stained Swiss-roll sections (n = 3) of mouse caecum (**k**) or colon (**l**) from *Zfp36l2*^*WT*^ and *Zfp36l2*^*IEC*^ mice treated with or without DSS, collected on day 14. **m**, Representative *Lgr5-*FISH of Swiss-roll sections (n = 3) of mouse colon from *Zfp36l2*^*WT*^ and *Zfp36l2*^*IEC*^ mice treated with or without DSS, collected on day 14.

These observations of a temporospatial sequence of ZFP36L2 and LGR5 expression during crypt regeneration suggested that ZFP36L2 might have a functional role in the injury-induced dedifferentiation of TA progenitors into a LGR5^+^ ISC state. To test this hypothesis, we generated *Vil1*^*c*^^*re*^*Zfp36l2*^*fl/fl*^ mice to knockout *Zfp36l2* in IECs^39,40^ (henceforth called *Zfp36l2*^*IEC*^ mice) and *Vil1*^*cre*^*Zfp36l2*^*+/+*^ (*Zfp36l2*^*WT*^) and *Vil1*^*cre*^*Zfp36l2*^*fl/+*^ (*Zfp36l2*^*HET*^) littermate controls (Extended Data Fig. 3a,b). *Zfp36l2*^*IEC*^ mice displayed normal colon morphology and body weight (Extended Data Fig. 3b-d). Immunofluorescence (IF) and RNA fluorescence in situ hybridization (FISH) staining of the colon revealed a reduction in LGR5^+^ ISCs from *Zfp36l2*^*IEC*^ mice (Extended Data Fig. 3e,f). This result was quantified by crossing *Zfp36l2*^*IEC*^ and *Zfp36l2*^*WT*^ mice with *Lgr5*^*eGFP-creERT2*^ reporter mice^16^ and performing flow cytometry for LGR5–eGFP^+^ cells on isolated colon crypts (Extended Data Fig. 3g–i). Thus, at homeostatic baseline, *Zfp36l2*^*IEC*^ mice seem to maintain normal intestinal morphology and function despite having a reduced LGR5^+^ ISC population. To determine the function of intestinal epithelial ZFP36L2 during stress, we treated mice with 3.5% DSS for 7 days to induce intestinal injury, followed by 7 days of regeneration once DSS treatment was stopped. *Zfp36l2*^*IEC*^ mice displayed increased weight loss during DSS treatment and incomplete weight recovery 7 days after DSS withdrawal relative to *Zfp36l2*^*WT*^ controls. This result was consistent with more substantial epithelial injury and impaired regeneration after *Zfp36l2* knockout (Fig. 2d). Pathology revealed shortening of the colon and caecum with persistent inflammation, crypt loss and decreased *Lgr5* expression at day 14 in *Zfp36l2*^*IEC*^ mice compared with *Zfp36l2*^*WT*^ controls (Fig. 2e and Extended Data Fig. 3j–m).

**Extended Data Fig. 4:**
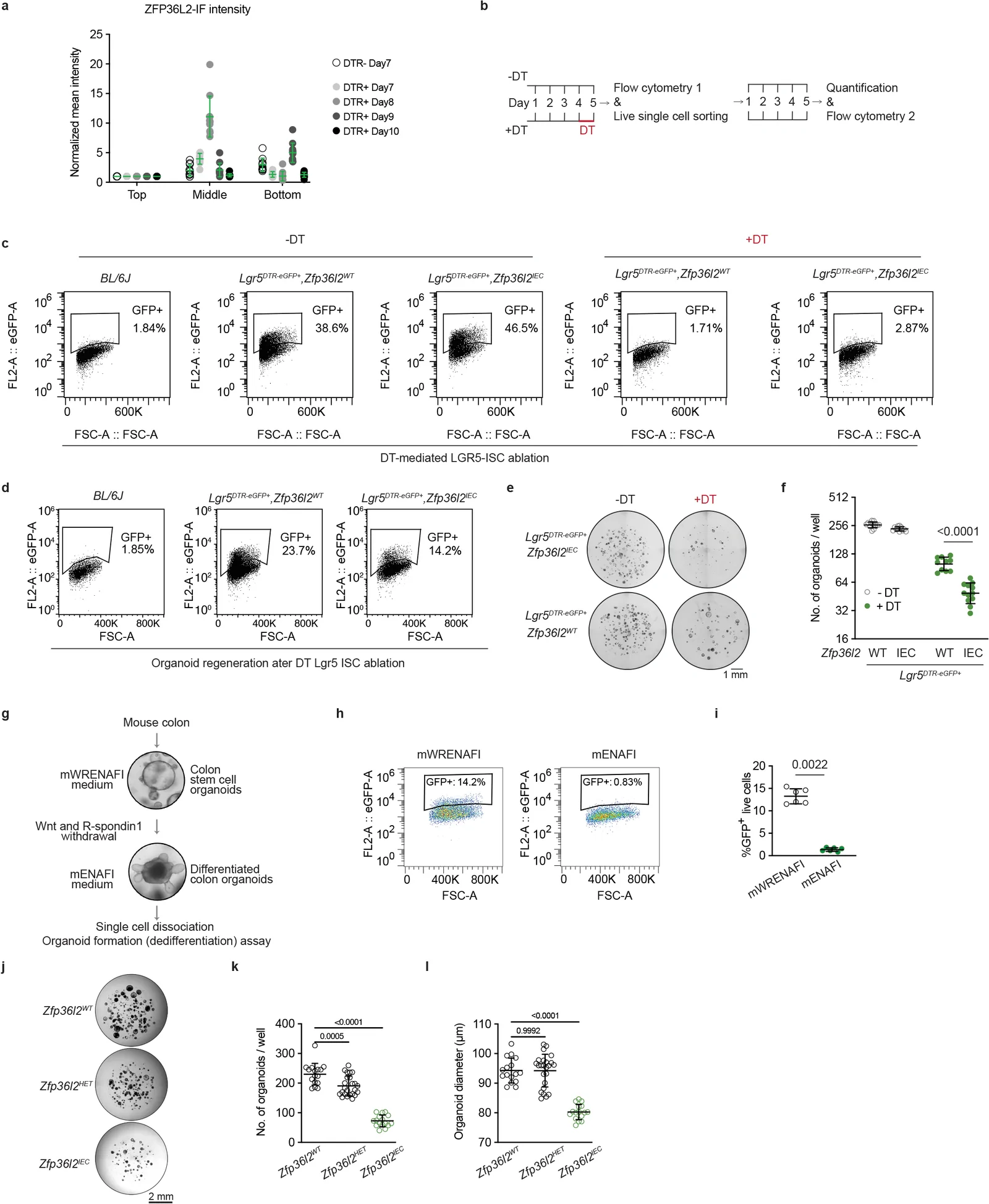
Experimental design and characterization of *Lgr5*-DTR-eGFP ablation and organoid dedifferentiation. **a**, Quantification of ZFP36L2-IF intensity dynamics shown in Fig. 2f. For each crypt region, cells were divided into the top, middle, and bottom thirds along the crypt axis. Top, top third; Middle, middle third; Bottom, bottom third. n = 11 regions. Mean ZFP36L2-IF intensity for each region was normalized to the local top one-third signal. Each dot represents one region; green bars indicate mean ± s.d. **b**, Schematic of *Lgr5*-DTR ablation and organoid dedifferentiation assay. Organoids were derived from colon crypts of 6–8-week-old *Lgr5*^*DTR-eGFP-*^*Zfp36l2*^*WT*^; *Lgr5*^*DTR-eGFP+*^*Zfp36l2*^*WT*^and *Lgr5*^*DTR-eGFP+*^*Zfp36l2*^*IEC*^ mice, and maintained in mouse intestinal stem cell medium (mWRENAFI). After 4 days in culture, diphtheria toxin (DT; 0.6 μg/mL) was added to the +DT group for 24 h. DT-treated and untreated organoids were then dissociated, live cells were harvested by flow sorting, and single cells were reseeded at low density (2,000 cells/40 μL Matrigel/well) in mWRENAFI to induce dedifferentiation. Regenerated organoids (number of organoids per well) were quantified 5 days after reseeding. **c**, Representative flow cytometry density plots showing the frequency of *Lgr5*-DTR-eGFP positive cells in organoids with or without 24 h DT treatment, measured at day 5 (the time point used for sorting in the dedifferentiation assay (**b**)). **d**, Representative flow cytometry density plots showing regeneration of *Lgr5*-DTR-eGFP positive cells 5 days following DT treatment and seeding of live single cells. **e**, Representative brightfield images of regenerated organoids in individual wells 5 days after reseeding of live single cells in mWRENAFI. n = 3 mice per genotype; 3–4 wells per mouse. Scale bar, 1 mm. **f**, Quantification of regenerated organoids per well from (**e**). Data are mean ± s.d. Two-tailed Mann–Whitney *U* test. n = 3 mice per genotype; 4 wells per mouse. **g**–**l**, Dedifferentiation assay. **g**, Schematic of organoid dedifferentiation assay. Colon organoids derived from *Zfp36l2*^*WT*^, *Zfp36l2*^*HET*^, or *Zfp36l2*^*IEC*^ mice were cultured in mouse intestinal stem cell medium (mWRENAFI). After 3 days, WNT and R-spondin 1 were withdrawn to induce differentiation. After 2 days, differentiated organoids were dissociated, sorted for live cells, and single cells were reseeded at low density at 2,000 cells/40 μL Matrigel/well in mWRENAFI medium to induce dedifferentiation. Organoid regeneration (organoid number and organoid size/well) was quantified 4 days following reseeding. **h**, Representative flow cytometry density plots showing the frequency of *Lgr5*-eGFP positive cells after 5 days of culture in mWRENAFI (left), or after 3 days in mWRENAFI followed by 2 days in mENAFI (right) medium lacking WNT and R-spondin 1. **i**, Quantification of *Lgr5*-eGFP positive cells from (**h**). n = 2 mice; 3 technical replicates per mouse. Data are mean ± s.d. Two-tailed Mann–Whitney *U* test. **j**, Representative images of organoids regenerating in single wells, 4 days following differentiated single cell reseeding in mWRENAFI. Scale bar, 2 mm. **k**–**l**, Quantification of (**k**) organoid number and (**l**) diameter from panel **j**. Organoids were generated from three mice (6-8 weeks old) per genotype; n = 16, 24, 16 wells from 3 mice per genotype. Data are mean ± s.d.; two-tailed Mann-Whitney *U* test.

To determine whether epithelial cell-intrinsic mechanisms inhibit dedifferentiation in colonic epithelia of *Zfp36l2*^*IEC*^ mice, we used an orthogonal *Lgr5*^*DTR*^ mouse model in which LGR5^+^ ISCs are directly ablated using diphtheria toxin (DT)^5^. Direct ablation of LGR5^+^ ISCs induced *Zfp36l2* expression first in the TA compartment, with expression moving down the crypt and preceding the restoration of crypt base *Lgr5* expression (Fig. 2f and Extended Data Fig. 4a). Next, we generated *Lgr5*^*DTR-eGFP+*^*Zfp36l2*^*IEC*^ and *Lgr5*^*DTR-eGFP+*^*Zfp36l2*^*WT*^ mice, administered DT to selectively ablate LGR5^+^ ISCs and assayed the restoration of *Lgr5* expression on the basis of the dedifferentiation of LGR5^–^ cells (Fig. 2g,h). Consistent with results from the DSS-induced colitis model, loss of *Zfp36l2* impaired the recovery of LGR5^+^ crypt base ISCs following the direct ablation of LGR5^+^ cells. To exclude potential confounders from in vivo DT administration, we established organoids from the colon epithelium of *Lgr5*^*DTR-eGFP+*^*Zfp36l2*^*IEC*^ and *Lgr5*^*DTR-eGFP+*^*Zfp36l2*^*WT*^ mice. Organoids were treated with vehicle or DT at day 4 for 24 h, and DT-dependent ISC ablation was verified by flow cytometry for LGR5–eGFP expression (Extended Data Fig. 4b,c). Flow-sorted single cells were seeded at low density to facilitate the regeneration of new organoids, a function that requires dedifferentiation into an LGR5^+^ ISC state^41^. *Lgr5*^*DTR-eGFP+*^*Zfp36l2*^*IEC*^ organoids regenerated significantly fewer organoids than *Lgr5*^*DTR-eGFP+*^*Zfp36l2*^*WT*^ controls (Extended Data Fig. 4e,f). Flow cytometry confirmed re-expression of LGR5–eGFP, with reduced LGR5–eGFP re-expression in organoids recovered from *Lgr5*^*DTR-eGFP+*^*Zfp36l2*^*IEC*^ mice (Extended Data Fig. 4d). Thus, *Zfp36l2* deletion inhibits the dedifferentiation of LGR5^*–*^ cells into LGR5^+^ cells and impairs organoid regeneration after DT-mediated LGR5^+^ ISC ablation.

We also established colon organoids from *Zfp36l2*^*IEC*^, *Zfp36l2*^*HET*^ and *Zfp36l2*^*WT*^ mice by plating intact crypts (Extended Data Fig. 4g–l). After 3 days of ISC culture in mWRENAFI medium^41^ (Methods), WNT and R-spondin1 were withdrawn to induce differentiation for 3 days (Extended Data Fig. 4g–i). To assess dedifferentiation capacity, differentiated organoids were dissociated into single cells, flow-sorted for live cells and reseeded at low density to regenerate new organoids. *Zfp36l2*^*IEC*^ cells re-established fewer and smaller organoids than *Zfp36l2*^*WT*^ and *Zfp36l2*^*HET*^ controls (Extended Data Fig. 4j–l). In summary, both the DSS and *Lgr5*^*DTR*^ ablation models demonstrated that ZFP36L2 is required for injury-induced dedifferentiation of IECs into an ISC state, which in turn is required for epithelial regeneration and wound healing (Fig. 2i).

## Loss of *ZFP36L2* abrogates CRC metastasis

**Fig. 3:**
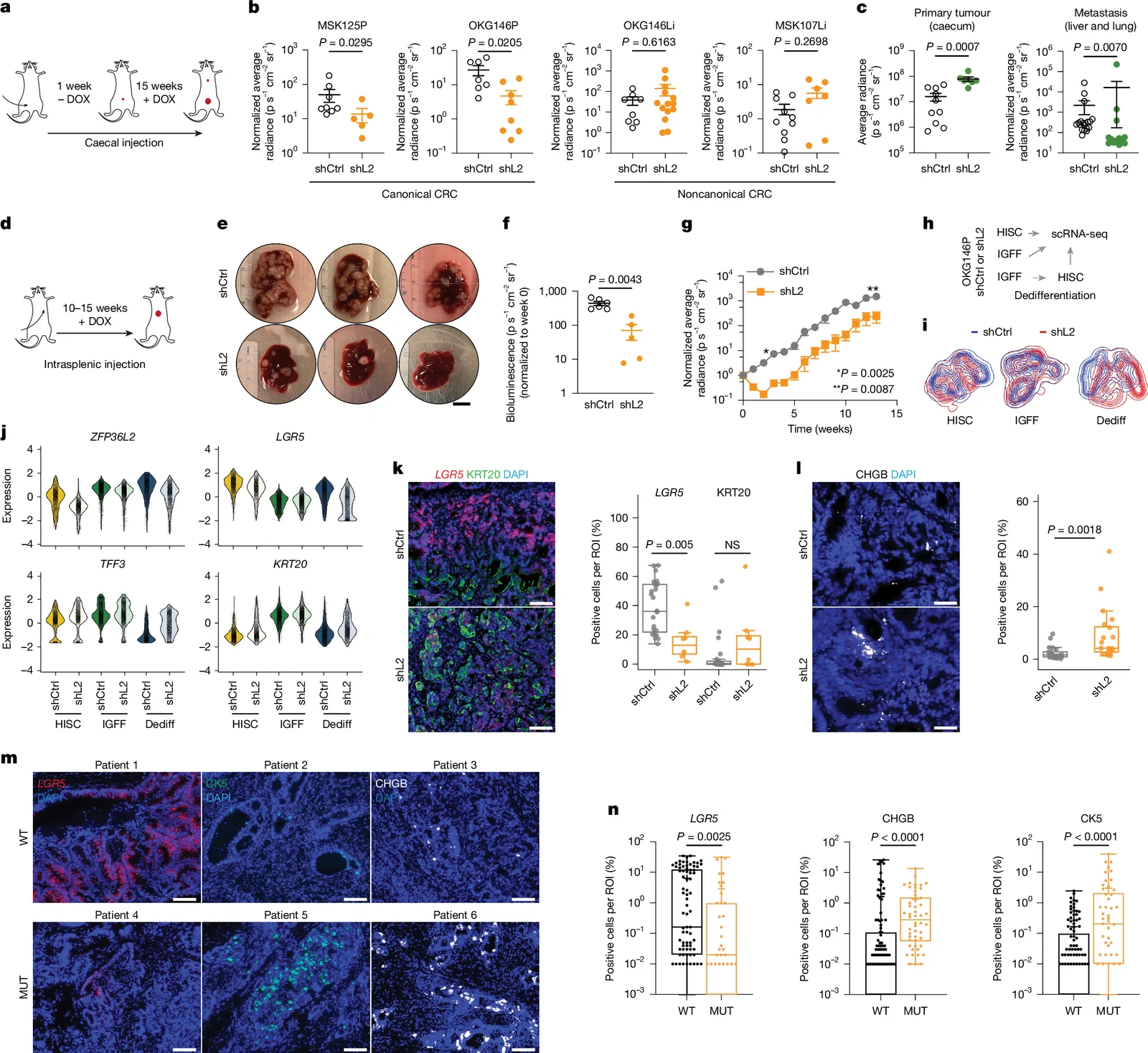
Loss of *ZFP36L2* inhibits CRC metastasis seeding and ISC dedifferentiation but promotes non-canonical differentiation. **a**–**c**, Orthotopic caecal xenotransplantation experiments. **a**, Schematic of the experiment. DOX, doxycycline. **b**, In vivo abdominal BLI average radiance, normalized to BLI at the time of doxycycline diet initiation. shL2, shZFP36L2. *n* (left to right) = 8, 5, 7, 8, 8, 14, 10 and 7 animals per group. Mean ± s.e.m.; two-tailed Mann–Whitney *U-*tests. **c**, End point ex vivo BLI of MSK107Li samples. Metastasis signals were normalized to orthotopic caecal signals in the same animals. *n* = 10 (shCtrl) and 7 (shL2) mice. Mean ± s.e.m.; two-tailed Mann–Whitney *U-*tests. **d**–**g**, Orthotopic liver metastasis seeding experiments. **d**, Schematic of the experiment. **e**, Representative ex vivo images of MSK107Li liver metastases. **f**, Average radiance of week 13 metastasis normalized to week 0 BLI. *n* = 6 (shCtrl) and 5 (shL2) mice. Mean ± s.e.m.; two-tailed Mann–Whitney *U-*tests. **g**, Weekly whole-body in vivo liver BLI (mean ± s.e.m.) normalized to week 0. *n* = 7 (shCtrl) and 5 (shL2) mice. Two-tailed Mann–Whitney *U-*tests. **h**, Schematic of the experiment. **i**. Kernel density estimate contour plots of scRNA-seq data from **h**, showing the overlap and divergence of cell states in OKG146P organoids transduced with shCtrl or shL2 and cultured in HISC, IGFF or dedifferentiated (Dediff) conditions. **j**, Violin plots showing expression of the indicated markers. **k**,**l**, Representative immunostaining (left) and quantification (right) of *LGR5* RNA FISH and KRT20 IF in MSK107Li (**k**; see also **d**–**g**) and CHGB IF in OKG146Li-MS2 liver metastasis (**l**). The graphs show the per cent *LGR5* and KRT20^+^ cells in each of the 26 regions of interest (ROIs) (252,330 cells; shCtrl) and 8 ROIs (194,530 cells; shL2) or CHGB^+^ cells in each of the 21 ROIs (254,403 cells; shCtrl) and 20 ROIs (66,338 cells; shL2) from 3 mice per group. Box plots show the interquartile range, with the line indicating the median, and whiskers the minimum and maximum values. Two-tailed Mann–Whitney *U-*tests. **m**,**n**, Representative images (**m**) and quantification (**n**) of *LGR5* RNA FISH and CK5 and CHGB IF in WT or *ZFP36L2* mutated (MUT) paired primary–metastasis CRC samples from patients. *n* = 8 (*ZFP36L2* WT) and 4 (*ZFP36L2* MUT) samples. *LGR5* RNA FISH: 91 ROIs (1,301,960 cells; *ZFP36L2* WT) and 43 ROIs (1,154,616 cells; *ZFP36L2* MUT). CHGB and CK5 IF: 113 ROIs (1,814,441 cells; *ZFP36L2* WT) and 50 ROIs (1,302,471 cells; *ZFP36L2* MUT). Box plots show the interquartile range, with the line indicating the median, and whiskers the minimum and maximum values. Two-tailed Mann–Whitney *U-*tests. Scale bars, 50 µm (**l**), 100 µm (**k**,**m**) or 1 cm (**e**). Source data

**Extended Data Fig. 5:**
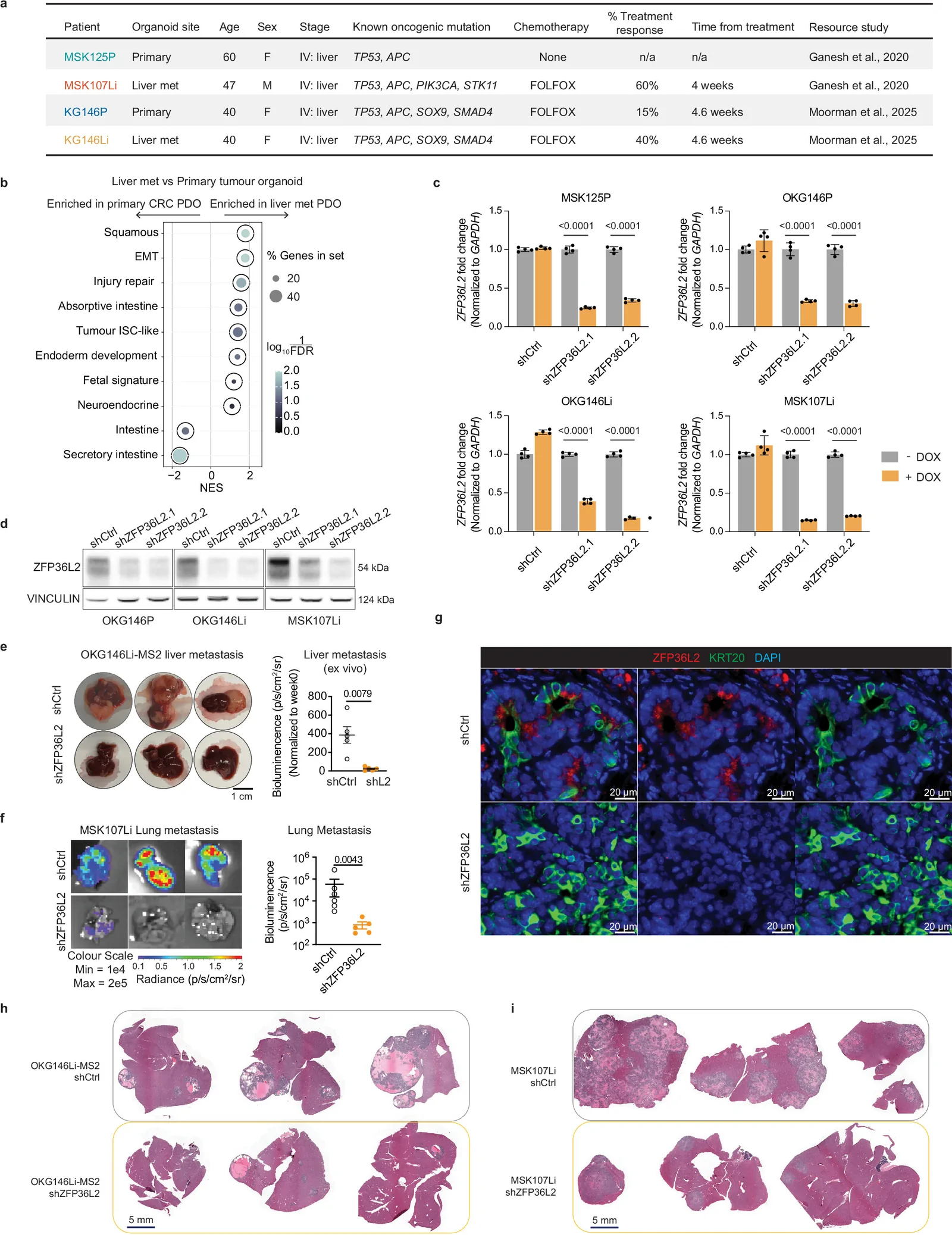
*ZFP36L2* knockdown impairs CRC metastasis. **a**, Clinical and mutational features of patient-derived CRC primary tumour and liver metastasis organoid lines used in this study. **b**, GSEA showing normalized enrichment scores (NES) for the indicated genesets from Moorman et al.^10^ (Supplementary Table 2b) in bulk RNA sequencing data, comparing liver metastasis derived organoids (MSK125P and OKG146P) with primary tumour derived organoids (MSK107Li and OKG146Li). **c**, Relative expression of *ZFP36L2*, normalized to *GAPDH*, in MSK125P, OKG146P, OKG146Li and MSK107Li organoids transduced with lentivirus directing doxycycline-inducible shRNAs targeting *ZFP36L2* (sh*ZFP36L2*.1, sh*ZFP36L2*.2) or control shRNA (shCtrl), with or without 2 μg/mL doxycycline treatment for 7 days. n = 4 replicates. Mean ± s.d. Two-sided Student’s *t*-tests. **d**, Western blots showing ZFP36L2 expression in the indicated organoids expressing sh*ZFP36L2* or shCtrl shRNA, 7 days following the addition of 2 μg/mL doxycycline. Vinculin served as a loading control. **e**, Representative ex vivo images and average radiance of week 12 normalized to week 0 BLI following intrasplenic injection of OKG146Li-MS2 organoids. n = 5 (shCtrl), 5 (sh*ZFP36L2*). Mean ± s.e.m. Two-tailed Mann-Whitney *U* tests. **f**, Representative BLI images (left) and quantification (right) of normalized average radiance of lung metastases (secondary seeding from liver metastasis) 13 weeks following intrasplenic injection of MSK107Li organoids as in Fig. 3. d–g). Mean ± s.e.m. n = 6 for shCtrl, n = 5 for sh*ZFP36L2*. Two-tailed Mann-Whitney *U* tests. **g**, Multiplexed immunofluorescence (IF) staining for ZFP36L2 (red) and KRT20 (green) of liver metastases derived from intrasplenic injection of shCtrl (top) and sh*ZFP36L2* (bottom) MSK107Li organoids, showing *ZFP36L2* knockdown and mutually exclusive staining of ZFP36L2 with KRT20 (canonical intestinal differentiation marker). **h**–**i**, H&E staining of liver metastases from (**h**) OKG146Li-MS2 and (**i**) MSK107Li PDO xenografts. Source data

As in tissue repair, disseminating metastasis-initiating cells enter injury-induced differentiated states. However, they can also dedifferentiate into an ISC state during metastatic seeding^7–10^ (Fig. 1a). To determine whether ZFP36L2 has a role in dedifferentiation into an ISC state during CRC progression, we engineered four patient-derived CRC organoid lines and used doxycycline-inducible short-hairpin RNAs (shRNAs) to knockdown *ZFP36L2* (shZFP36L2). Scrambled shRNA was used as a control (shCtrl) (Extended Data Fig. 5a–d). We also sought to clarify how the underlying canonical or non-canonical differentiation potential of the tumour cell population influences ZFP36L2 function. Therefore, we included two primary tumour-derived organoids (MSK125P and OKG146P) with canonical gene expression (largely ISC with some intestinal differentiation)^10^ and two metastasis-derived organoids (MSK107Li and OKG146Li) with both canonical and non-canonical differentiation potential^7,10^ (Extended Data Fig. 5a,b). To assess the role of ZFP36L2 in tumour growth in the intestinal niche, organoids transduced with shZFP36L2 or shCtrl were orthotopically injected into the caecum of NSG mice, and animals were administered doxycycline to knockdown *ZFP36L2* (Fig. 3a). Primary tumour-derived canonical organoids (OKG146P and MSK125P) exhibited decreased primary tumour growth after *ZFP36L2* knockdown (Fig. 3b, left). By contrast, non-canonical organoids derived from liver metastases (OKG146Li and MSK107Li) did not exhibit a decrease in primary tumour growth. (Fig. 3b, right). MSK107Li can generate spontaneous metastasis from orthotopic caecal primary tumours. However, despite having larger caecal primary tumours, mice with shZFP36L2 MSK107Li organoids had significantly decreased liver and lung metastatic burden (Fig. 3c).

Spontaneous metastasis requires multiple cell-state switches from ISC to differentiated during dissemination and to dedifferentiation during metastasis seeding (Fig. 1a). To isolate the function of ZFP36L2 specifically in the dedifferentiation switch, we used a metastasis-seeding model using intrasplenic injection (Fig. 3d). As the original OKG146Li organoid is capable of efficient intrahepatic outgrowth^10^ but does not efficiently initiate metastatic seeding, we serially passaged OKG146Li cells through mouse liver twice to generate a highly metastatic seeding line (OKG146Li-MS2; Methods). *ZFP36L2* knockdown in both MSK107Li and OKG146Li-MS2 organoids significantly impaired liver metastasis seeding after intrasplenic injection, and fewer and smaller tumours were observed (Fig. 3e,f and Extended Data Fig. 5e,h,i). shCtrl-expressing MSK107Li organoids generated secondary lung metastases from the liver, a process nearly completely blocked by shZFP36L2 (Extended Data Fig. 5f). Notably, the first 2 weeks after intrasplenic injection is a period of high attrition. Disseminating cancer cells extravasating from liver sinusoids and infiltrating into the hepatic parenchyma must overcome immune, biochemical and growth factor stressors to survive, with only a small fraction of injected cells overcoming these hurdles to reinitiate tumour growth^4^. During this early seeding period, shCtrl tumours displayed a slow but steady increase in bioluminescence imaging (BLI) signals. By contrast, shZFP36L2 tumours showed a sustained decrease in BLI signals from immediately after injection until week 2, a result consistent with the observed substantial reduction in early metastatic seeding and reinitiation of growth in the liver. Beginning around week 2, the BLI signal in shZFP36L2 tumour-bearing mice increased, which suggests that the rare cells that successfully seeded were able to subsequently regrow. Indeed, growth kinetics were similar to controls and ultimately gave rise to macroscopic tumour nodules (Fig. 3g). Thus, *ZFP36L2* knockdown substantially impairs the ability of tumour cells to initially seed metastasis in the liver and lungs.

**Extended Data Fig. 6:**
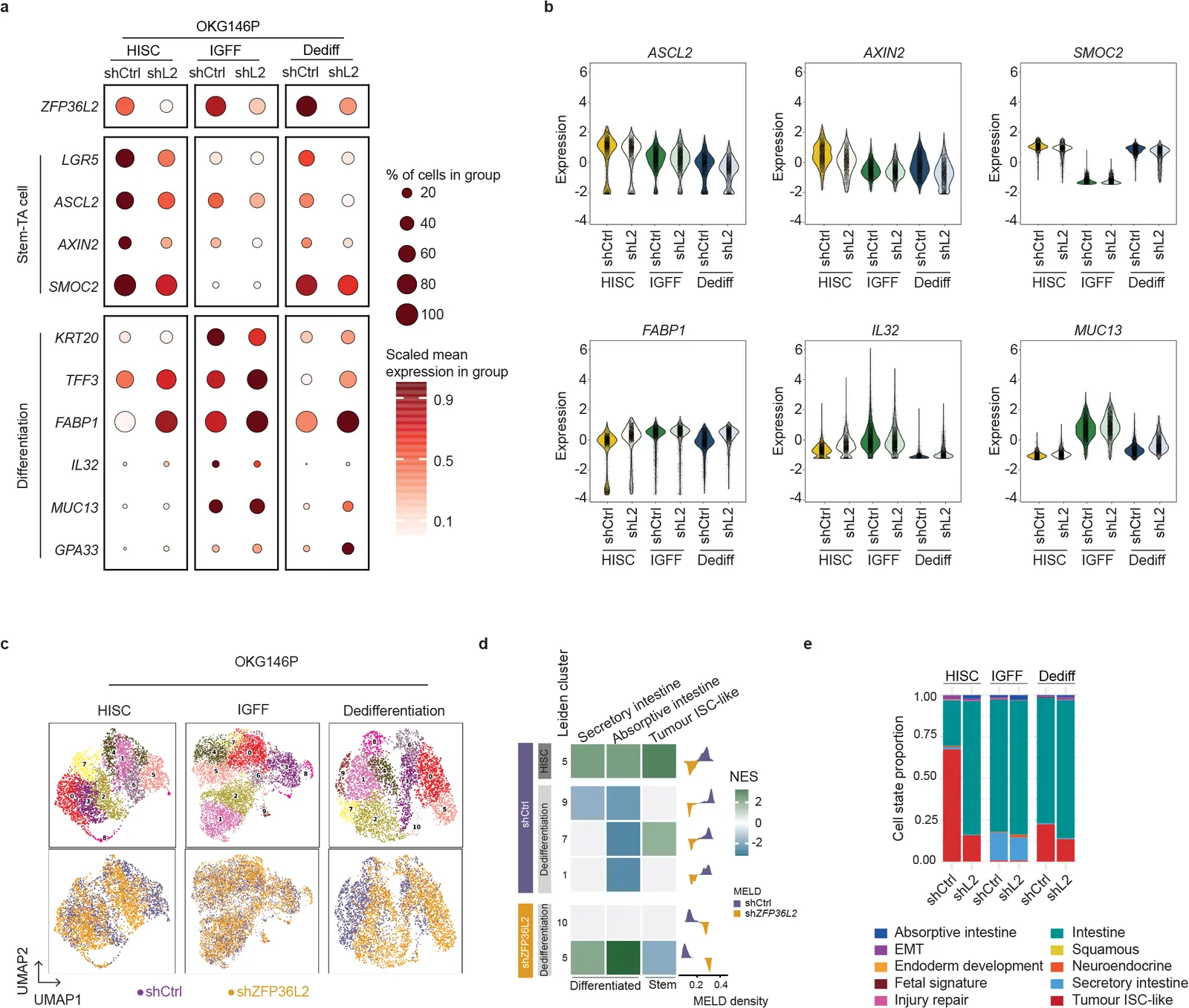
Single cell analysis of *ZFP36L2*-dependent ISC dedifferentiation. **a**, Dot plot showing expression of the indicated stem-TA and differentiation marker genes in HISC, IGFF or dedifferentiation (Dediff) conditions (See Fig. 3j for experimental design) in OKG146P organoids expressing shRNA targeting *ZFP36L2* (sh*L2*) or control shRNA (shCtrl). **b**, Violin plots showing the expression of stem cell markers (*ASCL2*, *AXIN2*, *SMOC2*) and canonical differentiation markers (*FABP1*, *IL32*, *MUC13*) across all conditions. *ZFP36L2* expression was upregulated in IGFF and further elevated in dedifferentiated cells. **c**, Leiden clustering of single-cell transcriptomes from OKG146P PDOs, with or without *ZFP36L2* knockdown, highlighting distinct cellular subpopulations. **d**, MELD analysis of Leiden clusters from (**c**), identifying clusters specifically enriched in control or *ZFP36L2* knockdown in each culture condition. Only significant condition-specific clusters are shown, with MELD density indicating enrichment. The cell states of these clusters were further characterized by differential gene expression analysis, assessed through normalized enrichment scores (NES) by Gene Set Enrichment Analysis (GSEA) using gene sets listed in Supplementary Table 2b. **e**, Proportion of each indicated cell state across conditions of shCtrl or sh*ZFP36L2* (shL2) in OKG146P. Genesets used to designate cell states were derived from single-cell RNA sequencing of human CRC tumours.

To investigate the mechanism underlying the inefficient metastatic seeding of shZFP36L2 organoids, we tested the response of canonical primary CRC organoid cells to ex vivo differentiation and dedifferentiation cycles. OKG146P CRC organoids were cultured in human intestinal stem cell (HISC) medium, differentiated through growth factor withdrawal for 7 days (ref. ^10^) and then induced to dedifferentiate by passaging in HISC medium for 7 days (Fig. 3h). *ZFP36L2* knockdown decreased the proportion of tumour ISC cells in both HISC and dedifferentiated conditions (Extended Data Fig. 6a–e). Control organoids re-expressed ISC markers (*LGR5*,* ASCL2* and* AXIN2*) and downregulated differentiation markers (*TFF3* and* KRT20*) within 7 days of the addition of ISC growth factors (to induce dedifferentiation) compared with differentiated cells cultured in intestinal growth-factor-free (IGFF) conditions, albeit to a slightly lower level than in stable HISC conditions. By comparison, shZFP36L2-treated organoids showed persistently marked reductions in ISC markers and elevated differentiation marker expression following dedifferentiation (Fig. 3i,j and Extended Data Fig. 6a–e). These data suggest that *ZFP36L2* knockdown enforces differentiation and renders cancer cells incompetent to dedifferentiate back into an ISC state, which is critical for metastasis seeding. *ZFP36L2* expression was itself markedly upregulated during dedifferentiation (Fig. 3j and Extended Data Fig. 6a), which mirrors the observed induction of ZFP36L2 expression during injury–repair in the mouse colon (Fig. 2c,f).

**Extended Data Fig. 7:**
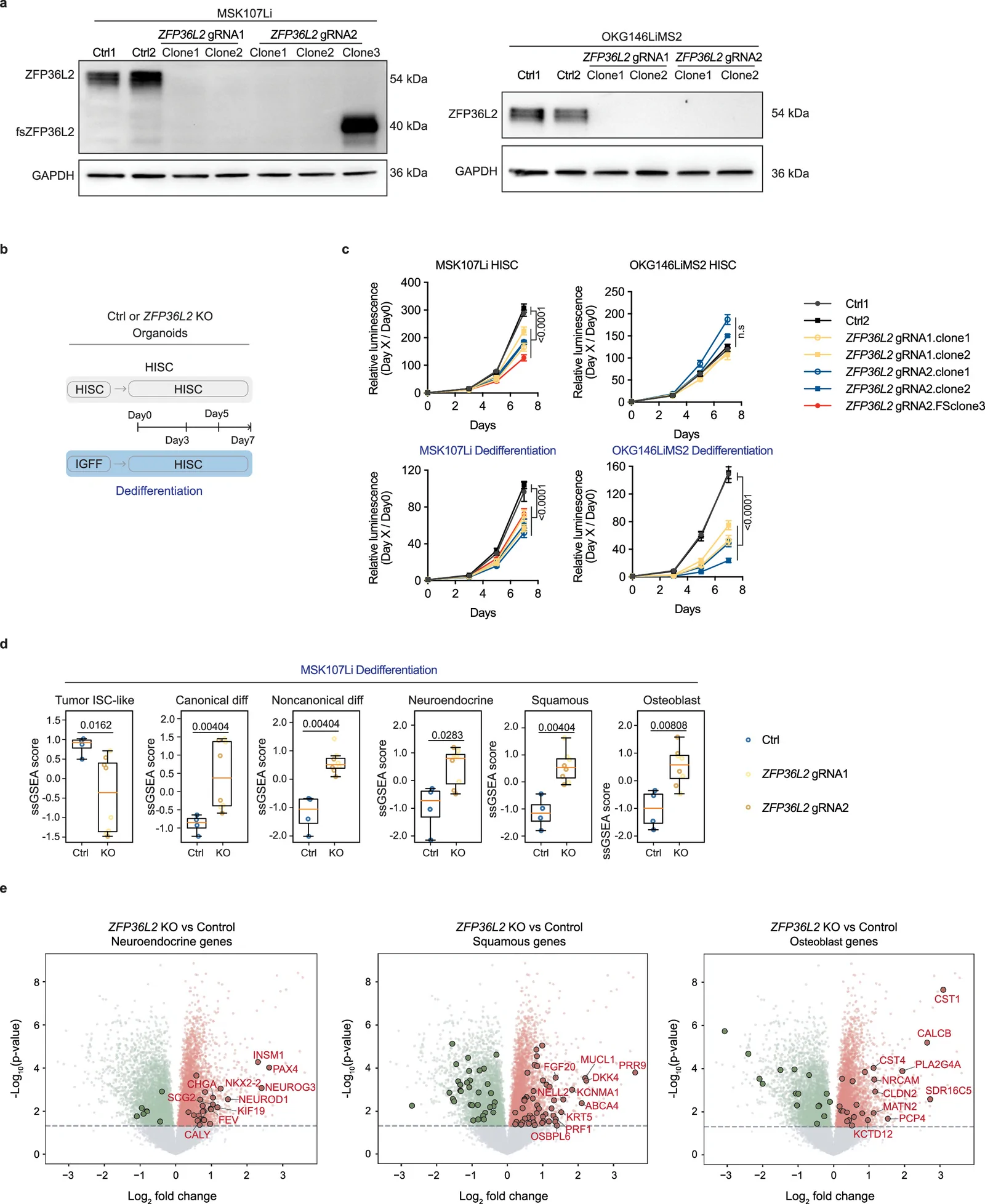
*ZFP36L2* knockout inhibits CRC dedifferentiation into an ISC state. **a**, Western blot validation of ZFP36L2 protein knockout/frameshift indel truncating mutation in MSK107Li and OKG146Li-MS2 organoids. Two individual clones for each of two gRNAs targeting the N-terminal region of ZFP36L2 were analysed. Organoids cultured in HISC medium for 7 days were harvested for western blotting. **b-d**, Organoid dedifferentiation assay **b**, Schematic of organoid dedifferentiation assay. Organoids were either continuously cultured in HISC medium (HISC group) or cultured in IGFF medium for 13 days, then dissociated into single cells and passaged into HISC medium (Dedifferentiation group), and analyzed at the indicated time points. **c**, Organoid growth measured by CellTiter-Glo viability assays at the indicated time points and normalized to day 0 immediately after plating as single cells (1000 cells/ 20 μL Matrigel/well). Data are mean ± s.d. n = 6 replicates/group. Two-tailed Mann–Whitney *U* tests between control and knockout groups. **d**, ssGSEA scores for the indicated gene sets (Supplementary Table 2a-b) from bulk RNA sequencing of MSK107Li control (n = 4) and *ZFP36L2* knockout organoid lines (n = 8) (Supplementary Table 3a). Two independent clones from each gRNA were pooled. Organoid on day 7 of dedifferentiation were harvested for bulk RNA sequencing. Box plots show the interquartile range (25th to 75th percentiles), horizontal line: median; whiskers: minimum and maximum values. Two-tailed Mann–Whitney *U* tests. **e**, Volcano plots showing differential gene expression from the bulk RNA-seq data shown in **d**. Welch’s *t*-test (two-sided) with Benjamini-Hochberg FDR correction. Genes from the indicated gene sets from Supplementary Table 2b are highlighted. The top ten upregulated genes from each gene set are labelled.

To exclude potential shRNA off-target effects, we used an independent CRISPR–Cas9 *ZFP36L2* knockout strategy (Extended Data Fig. 7). Consistent with our observations with shRNA-mediated *ZFP36L2* knockdown, CRISPR–Cas9 *ZFP36L2* knockout or truncation decreased dedifferentiation and organoid regeneration capacity (Extended Data Fig. 7a–c). Single-sample gene set enrichment analysis (ssGSEA) of bulk RNA-seq of the CRISPR-mediated knockout organoids similarly revealed decreased ISC-associated gene signatures and increased expression of canonical and multiple non-canonical differentiation programs (osteoblast, squamous and neuroendocrine) in *ZFP36L2* knockout organoids compared with *ZFP36L2* WT controls (Extended Data Fig. 7d,e). In summary, ZFP36L2 function is important for the maintenance and progression of canonical primary CRC tumours (Fig. 3b, left). Metastatic CRC caecal xenografts are less reliant on ZFP36L2 for the propagation of established tumours in the intestinal microenvironment (Fig. 3b, right); however, ZFP36L2 depletion reduces metastatic seeding (Fig. 3c–g). When metastases grow in ZFP36L2-depleted contexts, the surviving cancer cell population exhibits more non-canonical gene expression (Extended Data Fig. 7d,e).

## *ZFP36L2* loss drives non-canonical fates

**Extended Data Fig. 8:**
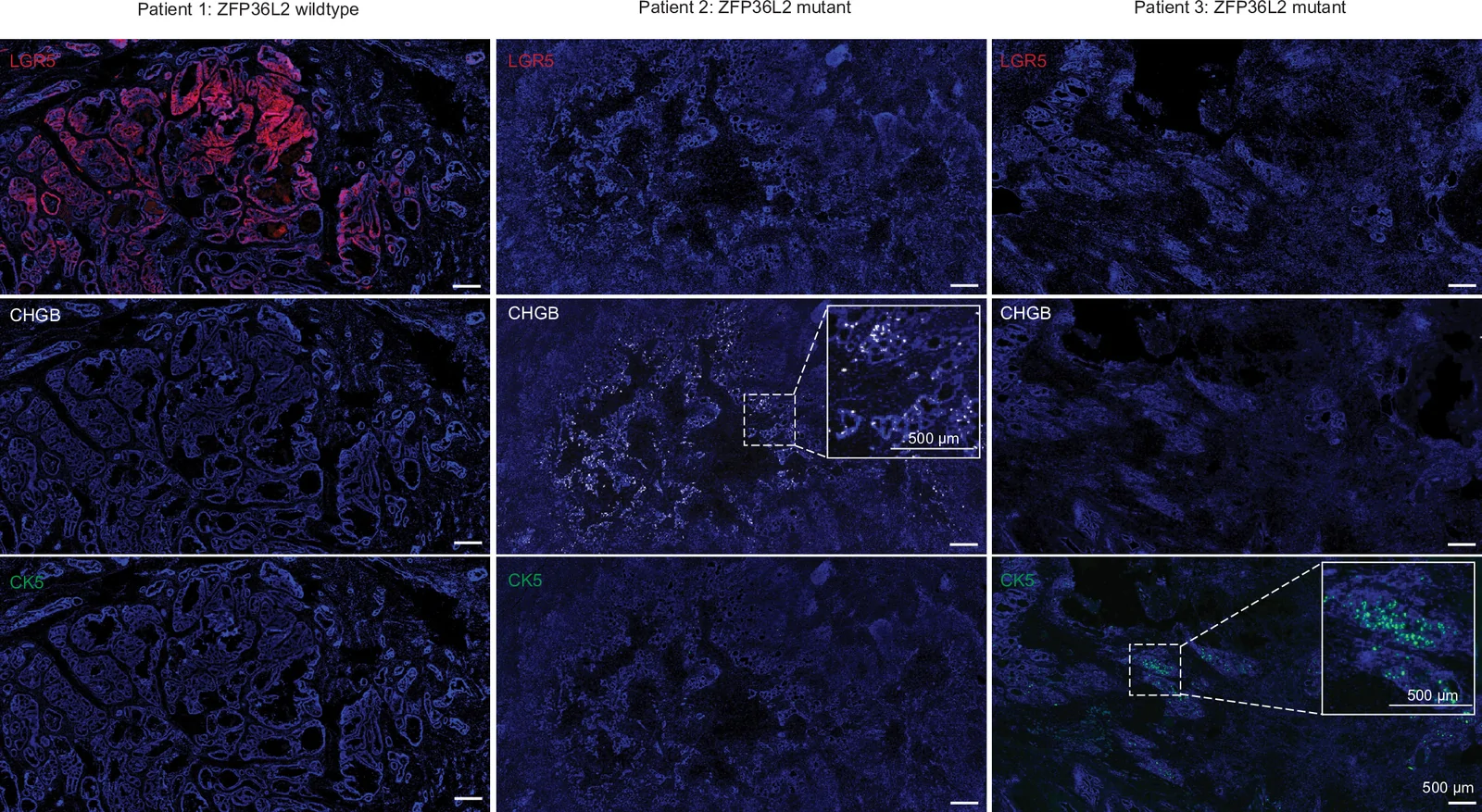
Non-canonical differentiation in patient CRC metastases with loss of function ZFP36L2 mutations. Representative images of LGR5-FISH or IF staining for CK5 (noncanonical squamous differentiation marker) or CHGB (noncanonical neuroendocrine differentiation marker) in patient CRC metastases with wildtype (WT) or mutant (MUT) ZFP36L2, showing loss of LGR5-FISH staining and divergent gain of CHGB or CK5 staining in focal areas of metastases from different patients.

shZFP36L2-expressing MSK107Li and MSK146Li-MS2 liver metastases displayed significantly decreased *LGR5* expression and increased expression of the canonical differentiation marker *KRT20* (Fig. 3k and Extended Data Fig. 5g). In patients, CRC metastases progressively adopt a non-canonical differentiation trajectory that is associated with poor clinical outcomes, and the ISC program is simultaneously switched off^10^. We therefore proposed that metastases that grow out in the absence of ZFP36L2-dependent ISC dedifferentiation might adopt non-canonical lineage identities. Immunostaining of shZFP36L2-expressing OKG146Li-MS2 liver metastases revealed significantly increased staining for the neuroendocrine differentiation marker chromogranin B (CHGB) compared with liver metastases from shCtrl organoids (Fig. 3l). We reasoned that loss-of-function *ZFP36L2* mutations might confer a selective disadvantage to canonical, intestinal-lineage-restricted primary tumours (for example, KG146P and MSK125P) that depend on ZFP36L2-driven dedifferentiation to continually replenish the primary tumour ISC state and seed metastasis (Fig. 3b). Conversely, by preventing ISC dedifferentiation, *ZFP36L2* loss-of-function mutations would exert selective pressure for the emergence and outgrowth of tumours expressing ISC-independent non-canonical programs, which are associated with metastasis and poor prognosis^10^. To test this hypothesis, we performed immunostaining for *LGR5* mRNA (ISC), CK5 (squamous) and CHGB (neuroendocrine) on *ZFP36L2* mutated and wild-type CRC metastasis samples from patients (Fig. 3m,n and Supplementary Table 3b). Expression of the ISC marker *LGR5* was significantly decreased, whereas neuroendocrine (CHGB) and squamous (CK5) non-canonical differentiation marker expression was increased in *ZFP36L2* mutated relative to *ZFP36L2* wild-type CRC metastasis samples (Fig. 3m,n). Among individual *ZFP36L2* mutated tumours and across patients, clusters of cells expressed either CK5 or CHGB, but seldom both markers (Extended Data Fig. 8). This result is consistent with *ZFP36L2* loss promoting a shift towards a more plastic state permissive for divergent differentiation into non-canonical states.

Together, these data indicate that ZFP36L2 acts as a crucial stress-induced mediator of dedifferentiation into an ISC state. In cancers with less plasticity (that is, primary tumours with intestinal-lineage-restricted programs), *ZFP36L2* loss is a selective disadvantage. By contrast, in more plastic settings (that is, cancer cells adapted to metastatic environments), *ZFP36L2* loss blocks re-entry into the ISC state, but the inherent plasticity of metastatic cells enables survival and metastatic outgrowth through the adoption of non-canonical programs.

## ZFP36L2 forms biomolecular condensates

**Fig. 4:**
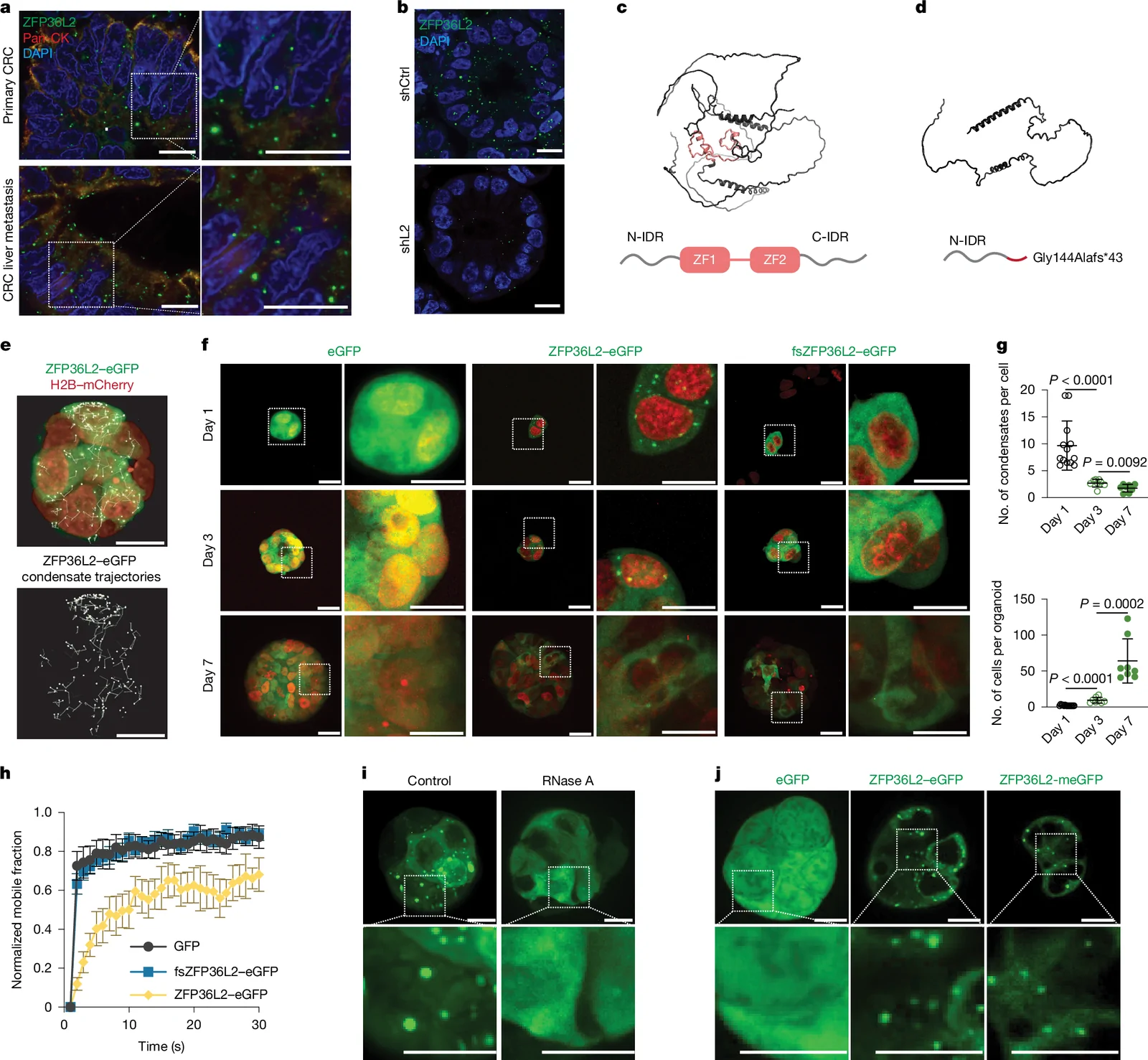
ZFP36L2 forms RNA-dependent, stress-responsive biomolecular condensates. **a**, Representative endogenous ZFP36L2 and pan-cytokeratin (Pan-CK) IF images of primary CRC and CRC liver metastasis from patient KG146. **b**, ZFP36L2 IF in MSK107Li organoids transduced with shCtrl (top) or shZFP36L2, demonstrating efficient knockdown and staining specificity. **c**,**d**, Top, AlphaFold2-predicted protein structure of human WT ZFP36L2 (**c**) and CRC patient hotspot mutation Gly144Alafs*43 (**d**). Bottom, schematic of the protein structures indicating the CCCH zinc fingers (ZFs) and the N-terminal and C-terminal IDRs. Red, ZF domains (amino acids 153–219); grey, IDRs. **e**, Representative images of maximum-intensity projection of motion-artefact-corrected 3D time-lapse video of OKG146Li organoids co-expressing ZFP36L2–eGFP and H2B–mCherry. Top, condensate positions and trajectories overlaid on the original image. Bottom, corresponding trajectory rendering shown separately. **f**, Representative images of single *z *plane confocal live-cell microscopy of MSK107Li H2B–mCherry (red) organoids with doxycycline-inducible expression of eGFP, WT ZFP36L2–eGFP or fsZFP36L2–eGFP. Organoids were plated as single cells on day 0, and doxycycline was added 24 h before imaging. **g**, Mean number of condensates per cell (top) and number of cells per organoid (bottom) at the indicated time points after single-cell seeding of ZFP36L2–eGFP-expressing MSK107Li H2B–mCherry organoids. Data are the mean ± s.d., and data points represent individual organoids (*n* = 8). Two-tailed Mann–Whitney *U-*tests. **h**, Normalized fluorescence intensity of MSK107Li organoids after photobleaching. Data representative of four replicates (mean ± s.d.). **i**, Live-cell imaging of ZFP36L2–eGFP-expressing MSK107Li organoids, treated with or without 1 μg μl^–1^ RNase A, 30 min after treatment. **j**, Live-cell imaging of OKG146Li organoids expressing doxycycline-inducible eGFP, ZFP36L2–eGFP or ZFP36L2–meGFP. Day 3 organoids were treated with 2 μg ml^−1^ doxycycline for 24 h before imaging. Insets are shown at higher magnification (bottom). ZFP36L2–meGFP exhibited condensate formation comparable to that of ZFP36L2–eGFP. Scale bars, 10 µm (**a**,**b**,**e**,**f** (zoom),**i**,**j**) or 20 µm (**f**, non-zoom).

**Extended Data Fig. 9:**
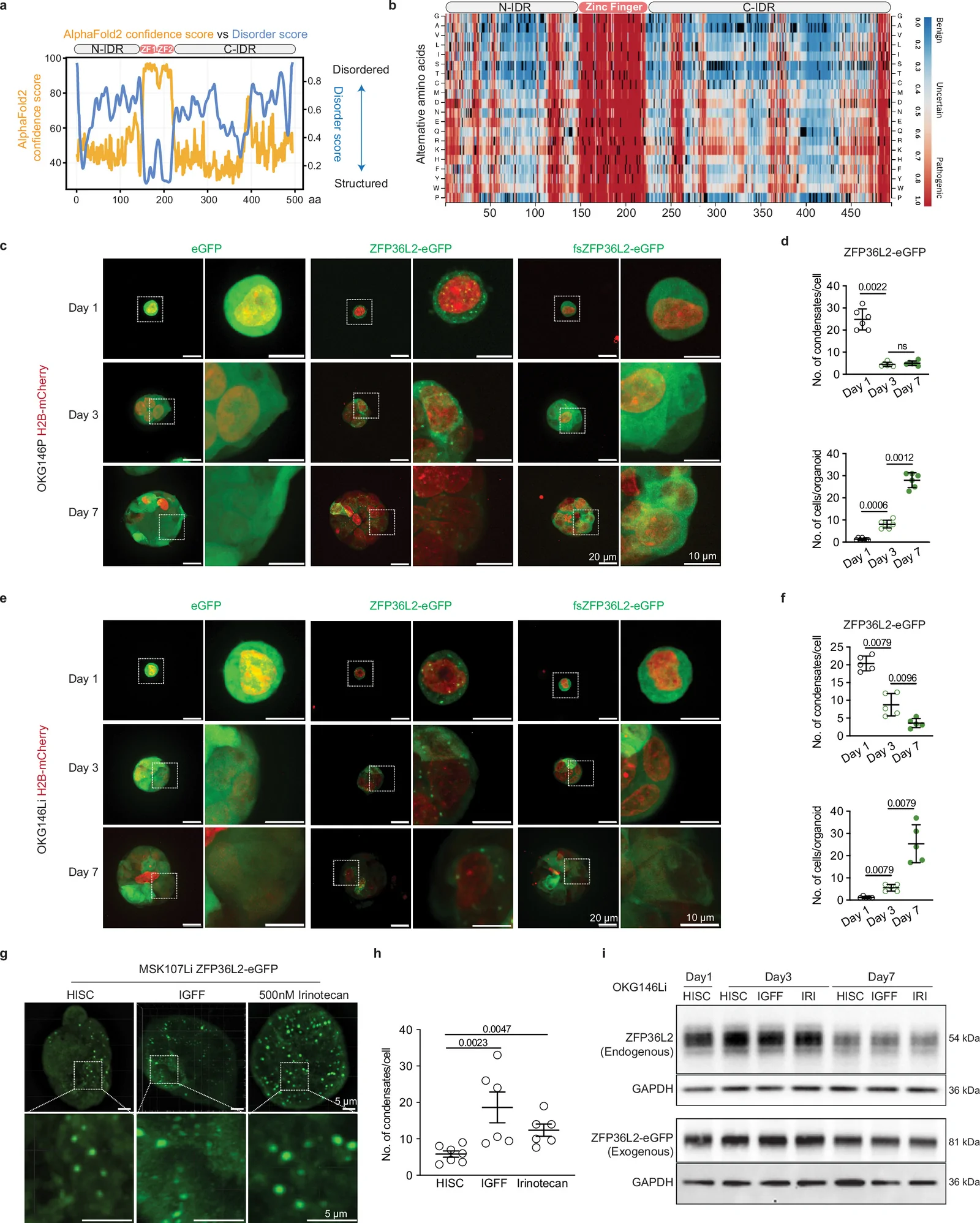
Structural and functional characterization of ZFP36L2 showing dynamics of ZFP36L2 condensates during organoid regeneration and stress responses. **a**, AlphaFold structure prediction score (yellow) and disorder propensity (blue) predicted by flDPnn^52^. **b**, AlphaMissense pathogenicity heatmap^85^ illustrating the predicted pathogenic potential of ZFP36L2 mutations. Mutations in critical structured regions are more likely to be pathogenic. **c**, Representative images of confocal live-cell microscopy of OKG146P H2B-mCherry organoids with doxycycline-inducible expression of eGFP, wt ZFP36L2-eGFP or G144Afs*43 fsZFP36L2. Organoids were plated as single cells on day 0, and doxycycline was added to induce gene expression 24 h prior to imaging for each time point. **d**, Quantification of (top) mean number of condensates per cell and (bottom) number of cells per organoid at the indicated timepoints following single cell seeding of ZFP36L2-eGFP expressing OKG146P H2B-mCherry organoids. Data are mean ± s.d., data points represent individual organoids. n = 6 organoids. Two-tailed Mann-Whitney *U* tests. **e**, Representative images of confocal live-cell microscopy of OKG146Li H2B-mCherry organoids with doxycycline-inducible expression of eGFP, wt ZFP36L2-eGFP or G144Afs*43 fsZFP36L2. Organoids were plated as single cells on day 0, and doxycycline was added to induce gene expression 24 h prior to imaging for each time point. **f**, Quantification of (top) mean number of condensates per cell and (bottom) number of cells per organoid at the indicated timepoints following single cell seeding of ZFP36L2-eGFP expressing OKG146Li H2B-mCherry organoids. Data are mean ± s.d., data points represent individual organoids. n = 5 organoids. Two-tailed Mann-Whitney *U* tests. **g**, Representative images of ZFP36L2-eGFP condensates in MSK107Li organoids 3 days following single cell seeding and organoid culture. Human intestinal stem cell (HISC) organoid medium containing EGF, Noggin, A-8301, FGF2, IGF-I (see Methods) was replaced with either fresh HISC, intestinal growth factor free (IGFF) medium lacking EGF, Noggin, A-8301, FGF2, IGF-I, or HISC + 500 nM irinotecan. Live cell images were taken 30 min after media change. **h**, Mean number of condensates per cell in MSK107Li organoids under the conditions described. Dots represent number of condensates in individual cells. Data are mean ± s.e.m. n = 4,3,3 organoids in HISC, IGFF and irinotecan treatment conditions respectively. Two-tailed Mann-Whitney *U* tests. **i**, Western blot of endogenous ZFP36L2 or lentivirally expressed (exogenous) ZFP36L2–eGFP in OKG146Li organoids harvested on day 1, day 3 and day 7 after single-cell plating. Organoids were also exposed to the same conditions used for live-cell imaging shown in (**g-h**), including 1 h of growth factor withdrawal in IGFF medium or 500 nM irinotecan treatment or at day 3 or day 7 before collection.

Next, we sought to understand the molecular mechanism of ZFP36L2-dependent stress adaptation. Immunostaining of primary and metastatic CRC tissue samples showed that endogenous ZFP36L2 localized to largely cytoplasmic punctate structures (Fig. 4a). ZFP36L2 puncta were conserved in CRC organoids and xenografts, and decreased after *ZFP36L2* knockdown (Fig. 4b and data not shown). AlphaFold2 structural analysis predicted extensive intrinsically disordered regions (IDRs) in ZFP36L2 N and C termini, which were characterized by low structural prediction scores, an absence of a secondary structure and high disorder propensity^42,43^ (Fig. 4c and Extended Data Fig. 9a,b). This result suggests that ZFP36L2 might form IDR-driven biomolecular condensates^44^. In patients with CRC, we identified a frequently mutated hotspot region downstream of the N-terminal IDR that leads to frameshift deletions and loss of the zinc finger domains (Figs. 1f and 4d). We therefore asked whether ZFP36L2 forms dynamic condensates with phase-separation properties. We engineered CRC organoids (MSK107Li, OKG146P and OKG146Li) with doxycycline-inducible constructs expressing eGFP, wild-type ZFP36L2–eGFP or the clinical frameshift deletion mutation Gly144Alafs*43/Gly5Argfs*329 (fsZFP36L2–eGFP) together with H2B–mCherry (Fig. 4e,f and Supplementary Video 1a–c). Tracking of ZFP36L2 puncta in motion-corrected 3D time-lapse images revealed that they were mobile in cells (Fig. 4e). Neither fsZFP36L2–eGFP nor eGFP formed condensates (Fig. 4f, Extended Data Fig. 9c–f and Supplementary Video 1). To track condensate dynamics during organoid formation, we dissociated 7-day-old organoids, seeded them as single cells and performed live-cell imaging during organoid regeneration (Fig. 4f,g and Extended Data Fig. 9c–f). ZFP36L2 condensates could be identified about 2 h after dissociation, and the number of condensates peaked around 10–12 h after dissociation (day 0) (Supplementary Video 2). This result is consistent with the previously established timing of entry into an L1CAM-high injury–repair or metastasis-initiating state^7^. The number of condensates reduced with further cell divisions and organoid regeneration (Fig. 4f,g, Extended Data Fig. 9c–f and Supplementary Video 2). In addition to tissue-disruptive stress, the withdrawal of growth factors or treatment with low-dose irinotecan (a chemotherapy) significantly increased the number of ZFP36L2 condensates. This result suggests that diverse cellular stresses converge to promote ZFP36L2 condensate formation (Extended Data Fig. 9g,h). ZFP36L2 protein levels increased on days 1–3 of organoid regeneration and declined to steady-state by day 7 (Extended Data Fig. 9i). Conversely, peak condensation occurred on day 1, preceding peak protein levels on day 3 (Fig. 4f,g). At each time point, growth factor withdrawal or irinotecan treatment induced condensate formation (Extended Data Fig. 9g,h) without changing overall ZFP36L2 protein levels (Extended Data Fig. 9i). These observations suggest that although ZFP36L2 protein levels are one determinant of condensate formation, cellular stresses can rapidly nucleate condensation through mechanisms independent of protein abundance.

**Extended Data Fig. 10:**
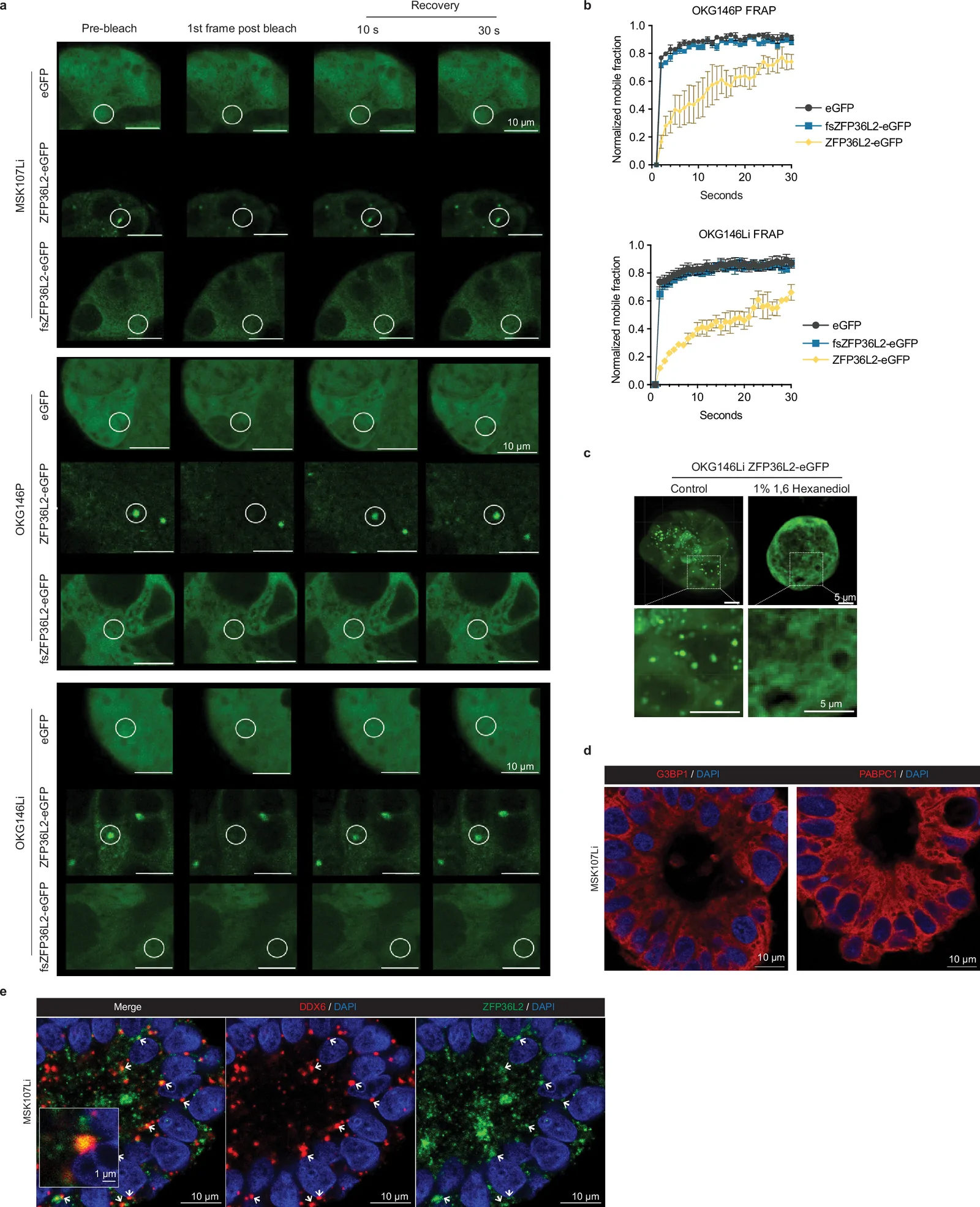
ZFP36L2 forms biomolecular condensates. **a**, Fluorescence recovery after photobleaching (FRAP) experimental setup. The circled area indicates laser photobleaching region. Representative images captured pre-bleach, and at the indicated time points post-bleach and recovery for 3 organoid lines (MSK107Li, OKG146P, OKG146Li). n = 4 organoids per line. **b**, Normalized fluorescence intensity recovery after photobleaching regions of interest in OKG 146P (top) and OKG146Li (bottom) organoids expressing the indicated proteins over time. Fluorescence intensity values are normalized to pre-bleaching intensity of the photobleached regions. Data are mean ± s.e.m. **c**, Live-cell imaging of OKG146Li organoids expressing ZFP36L2-eGFP, treated with or without 1% 1’6-hexanediol. Live cell images were taken 30 min post-treatment. **d**, Representative images of stress granule markers G3BP1 and PABPC1 immunostaining in MSK107Li organoids 1 h after growth factor withdrawal. G3BP1 and PABPC1 failed to form condensates under these conditions. e, Representative images of dual immunofluorescence staining for P-body marker DDX6 and ZFP36L2 in MSK107Li organoids 1 h after growth factor withdrawal, showing partial colocalization between DDX6 and ZFP36L2 puncta. Arrows indicate representative colocalized puncta. Inset shows a higher-magnification view.

Fluorescence recovery after photobleaching (FRAP) revealed that ZFP36L2–eGFP condensates exhibit gradual fluorescence recovery compared with the freely diffusible eGFP. This result is characteristic of phase-separated biomolecular condensates, which exhibit reduced fluidity (Fig. 4h, Extended Data Fig. 10a,b and Supplementary Video 3a–c). The clinical hotspot mutation fsZFP36L2, which lacks the zinc finger domain, exhibited free diffusion and did not form condensates, similar to eGFP (Fig. 4h and Extended Data Fig. 10a,b). Consistent with phase-separation characteristics, ZFP36L2 condensates could be dissolved by treating cells with 1% 1,6-hexanediol^45^ (Extended Data Fig. 10c). Treatment with 1 μg μl^–1^ RNase A caused dissipation of ZFP36L2–eGFP condensates (Fig. 4i), consistent with a requirement for RNA binding to nucleate condensate formation. Replacing eGFP with monomeric eGFP (meGFP), which has low fluorophore-autonomous condensation levels^46^, did not alter the pattern of intracellular puncta (Fig. 4j). Staining patterns were similar for ZFP36L2–eGFP, ZFP36L2–meGFP (Fig. 4j) and endogenous ZFP36L2 (Fig. 4a), which indicated that the fluorescent protein tags do not induce aberrant condensation. ZFP36L2 condensates showed partial overlap with DDX6, an mRNA decay factor also associated with P bodies, but did not overlap with the stress granule components G3BP1 and PABPC1^47^, which did not form condensates under the same conditions (Extended Data Fig. 10d,e). Together, these results highlight the distinct biology of ZFP36L2: it forms dynamic, RNA-dependent, stress-inducible biomolecular condensates in tumour tissues and organoids.

## ZFP36L2 mediates stress transcript decay

**Fig. 5:**
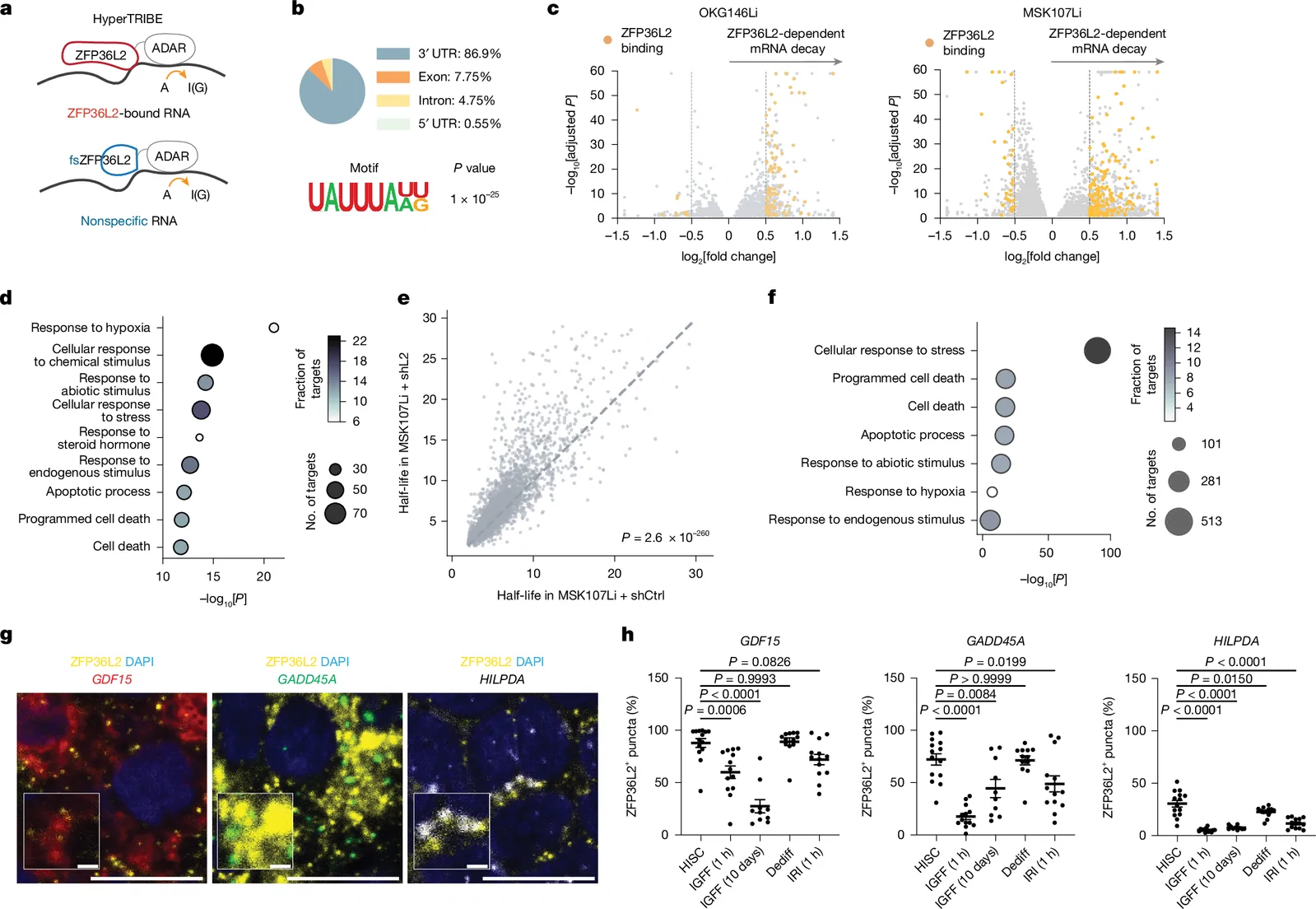
ZFP36L2-dependent mRNA binding and decay of stress-associated transcripts. **a**, Schematic of the HyperTRIBE experiment. **b**, Features of ZFP36L2-specific ADAR-edited mRNA sequences shared between OKG146Li and MSK107Li organoids. **c**, ZFP36L2-bound mRNAs (HyperTRIBE, yellow dots) undergoing ZFP36L2-dependent mRNA decay (based on ActD treatment and sequencing). Volcano plots show differential mRNA decay in shL2 versus shCtrl organoids. Wald test (negative binomial), two-sided, Benjamini–Hochberg FDR. HyperTRIBE-identified genes were significantly enriched among transcripts with increased stability after *ZFP36L2* knockdown. Fisher’s exact test: MSK107Li, odds ratio = 2.21, *P* = 2.4 × 10^–35^; OKG146Li, odds ratio = 2.47, *P* = 9.2 × 10^–75^. **d**, GO biological process enrichment analysis of ZFP36L2-dependent mRNA decay (after ActD treatment and sequencing), showing targets shared between OKG146Li and MSK107Li organoids. *n* = 379 genes, *P* < 0.05, fold change > 1 (Supplementary Table 5c). Hypergeometric test (one-sided) with Benjamini–Hochberg correction. **e**, Scatter plot showing SLAM-seq RNA half-lives in MSK107Li organoids expressing shL2 or shCtrl. Each dot indicates one transcript. One-sided Wilcoxon tests. **f**, Over-representation analysis of transcripts from SLAM-seq datasets, with significantly increased half-life in shL2 versus shCtrl treatments. Hypergeometric test (one-sided) with Benjamini–Hochberg correction. GO terms are as in **d**. **g**,**h**, Representative images (**g**) showing multiplexed ZFP36L2 IF and *GDF15*, *GADD45A* and *HILPDA* RNA FISH in OKG146Li-MS2 organoids, and per cent ZFP36L2 puncta positive for the indicated RNA FISH signal (**h**) in OKG146Li-MS2 organoids. Organoids were cultured in HISC for 7 days, cultured in IGFF for 10 days before switching to HISC for 7 days to induce dedifferentiation (Dediff), or cultured in HISC for 7 days before switching to IGFF (IGFF 1 h) or HISC containing 500 μmol irinotecan (IRI 1 h) for 1 h. Each dot indicates one organoid.* n* (left to right) = 14, 13, 10, 12 and 13 organoids per condition. Mean ± s.e.m.; two-tailed Mann–Whitney *U-*test. Scale bars, 1 µm (**g**, insets) or 10 µm (**g**).

**Extended Data Fig. 11:**
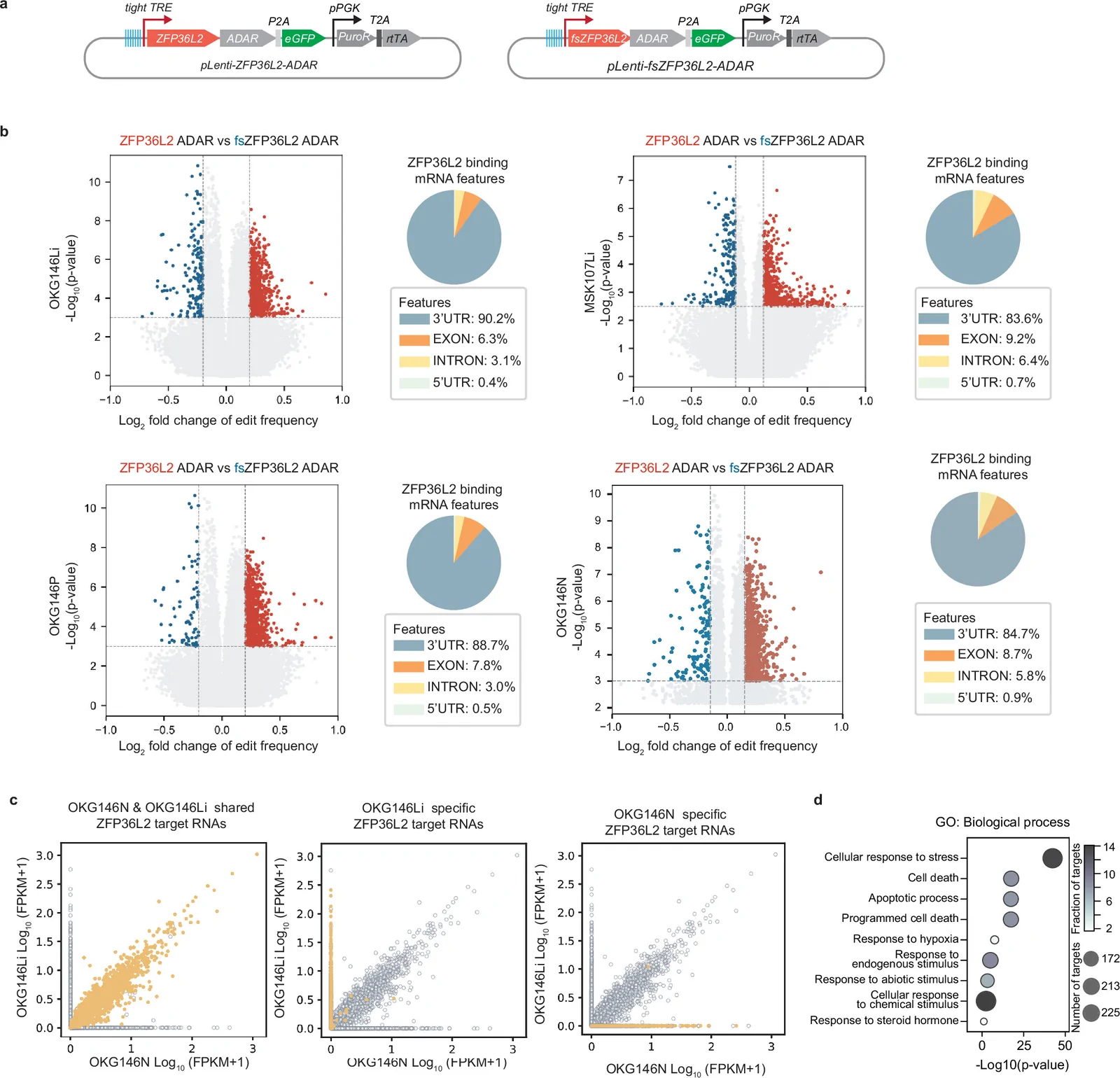
Profiling of ZFP36L2-bound mRNAs. **a**, Schematic of *ZFP36L2-ADAR* (left), *fsZFP36L2-ADAR* (right) expression constructs. The constructs were under a Tet-on inducible promoter and followed by a P2A-eGFP to indicate expression. **b***,* Volcano plots showing ZFP36L2-bound mRNAs identified through ADAR-mediated RNA editing, comparing wild-type (wt) ZFP36L2 to the frameshift mutant (fsZFP36L2) in OKG146Li (top left), MSK107Li (top right), OKG146P (bottom left), and OKG146N (bottom right). Likelihood ratio test (beta-binomial), One-sided, Benjamini-Hochberg (BH) FDR. Pie charts show genomic features of ZFP36L2-specific ADAR edit sites (-Log_10_ p-value > 3 and log_2_ fold change in edit frequency>0.1 (wt vs. fsZFP36L2). **c**, Scatter plots showing RNA expression in OKG146N and OKG146Li organoids, plotted as Log_10_ (FPKM + 1). Shared ZFP36L2-bound RNAs, as well as OKG146Li-specific and OKG146N-specific ZFP36L2 targets, are highlighted in yellow. The identities of the mRNAs and GO terms enriched in each group are provided in Supplementary Table 5a,d. **d**, Gene Ontology (GO) enrichment analysis of shared ZFP36L2-bound mRNAs identified in OKG146N, OKG146P and OKG146Li organoids (Supplementary Table 5d), using GO Biological Process gene sets. The dot plot shows representative GO terms from Fig. 5d. The x axis shows the −log10(*P*−value) of enrichment, dot size indicates the number of target transcripts in each category, and colour indicates the fraction of targets in each term. Hypergeometric test (one-sided). with Benjamini–Hochberg correction for multiple comparisons.

**Extended Data Fig. 12:**
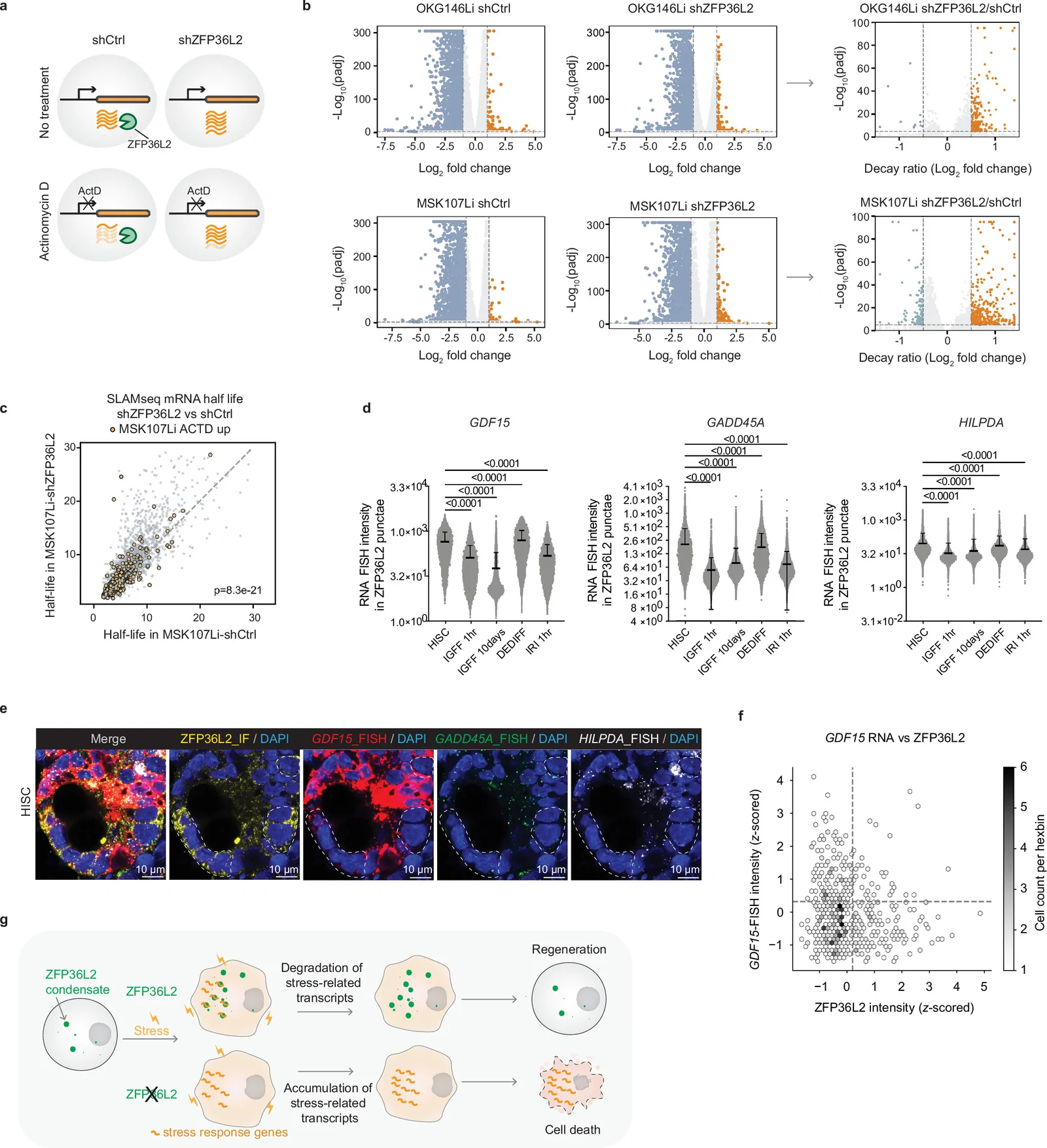
ZFP36L2-mediates mRNA decay. **a**, Schematic illustrating the measurement of ZFP36L2-dependent transcriptome-wide mRNA decay. In shCtrl cells, ZFP36L2 binding to target mRNAs induces degradation of select mRNAs, whose abundance increases upon *ZFP36L2* knockdown. Upon treatment with 5 μg/mL Actinomycin D (ActD) for 5 h, nascent mRNA transcription is inhibited, enabling quantification of mRNA decay by comparing transcript abundance at 0 h vs. 5 h of ActD treatment. ActD downregulated mRNAs whose expression increases upon *ZFP36L2* knockdown are surmised to have undergone ZFP36L2-dependent mRNA decay. **b**, (left) mRNA decay rate analysis in each condition (shCtrl and sh*ZFP36L2*) in OKG146Li (top) and MSK107Li (bottom), calculated as the fold change in gene expression following 5-hour actinomycin D treatment compared to untreated controls. (right) ZFP36L2-dependent mRNA decay in OKG146Li (top) and MSK107Li (bottom), plotting differential fold changes in mRNA decay between sh*ZFP36L2* and control shRNA (shCtrl) conditions (Supplementary Table 5b). Wald test (negative binomial), Two-sided, Benjamini-Hochberg (BH) FDR. c, Transcripts identified as significantly stabilized in the actinomycin D decay assay (**a-b**) in MSK107Li organoids, highlighted on the SLAM-seq half-life plot (Fig. 5e), establishing concordance between ActD decay and SLAM-seq assays in identifying ZFP36L2**-**dependent decay targets. Statistical significance was assessed by one-sided Wilcoxon tests. **d**, Target mRNA FISH intensity within ZFP36L2 puncta in OKG146Li-MS2 organoids. Organoids were cultured in HISC (HISC) for 7 days, cultured in IGFF for 10 days prior to switching to HISC for 7 days to induce dedifferentiation (Dediff), or cultured in HISC for 7 days prior to switching to IGFF (IGFF 1 hr) or HISC containing 500 μmol irinotecan (IRI 1 hr) for 1 h. Each dot: one ZFP36L2 puncta. n = 14,13,10,12,13 organoids/condition. Mean ± s.e.m. Two-tailed Mann–Whitney U test. e-f, Representative images (**e**) and quantification (**f**) of ZFP36L2 IF and target mRNA (GDF15) FISH intensity in each cell from 14 organoids. **g**, Working model of ZFP36L2-mediated detection and termination of cellular stress response. Upon cellular stress, ZFP36L2 is upregulated together with AP-1 related transcripts, binds to AU-rich 3’UTRs of stress-program associated mRNAs, in turn nucleating the formation of RNA-dependent biomolecular condensates. ZFP36L2 biomolecular condensates sequester stress-related transcripts, facilitating their degradation. This post-transcriptional regulation enables termination of the stress response, in turn facilitating cell survival after stress and eventual tissue regeneration. In the absence of ZFP36L2, unresolved stress responses lead to further accumulation of stress-inducible transcripts, ultimately resulting in cell death.

Next, we used HyperTRIBE^48,49^ to investigate ZFP36L2 RNA-binding targets. Full-length ZFP36L2 and fsZFP36L2 (which cannot bind RNA) were tagged with the hyperactive catalytic domain of adenosine deaminase acting on RNA (ADAR) containing the E488Q mutation, which enhances editing efficiency and reduces sequence bias^48^ (Fig. 5a). Two patient-derived CRC liver metastasis organoids (MSK107Li and OKG146Li) and matched primary tumour and normal colon organoids from patient KG146 (OKG146P and OKG146N) expressing doxycycline-inducible ZFP36L2–ADAR or fsZFP36L2–ADAR were subjected to RNA-seq to identify adenosine to inosine (read as guanosine) edits (Supplementary Table 5a). The majority of edits were in the ZFP36L2–ADAR-expressing organoids, a result consistent with the requirement of full-length protein-specific mRNA interactions, which are absent in the fsZFP36L2–ADAR control (Fig. 5b,c and Extended Data Fig. 11a–d). More than 80% of ZFP36L2-specific edits occurred in mRNA 3′ UTRs, predominantly at the conserved UAUUUA motif (Fig. 5b and Extended Data Fig. 11b), which is consistent with the known binding properties of the ZFP36 protein family^34^. As ZFP36L2 3′ UTR binding can promote mRNA decay^37,50,51^, we treated OKG146Li and MSK107Li organoids with the transcriptional inhibitor actinomycin D (ActD) to inhibit de novo mRNA synthesis^51^, and measured RNA decay rates (Extended Data Fig. 12a). Among the mRNA transcripts for which abundance decreased after 5 h of ActD treatment relative to 0 h baseline (indicative of mRNA decay), we identified mRNAs for which expression increased in shZFP36L2 organoids relative to shCtrl organoids (that is, ZFP36L2-dependent mRNA decay) (Fig. 5c and Extended Data Fig. 12b). Overlaying ZFP36L2-bound (HyperTRIBE) and ZFP36L2-dependent mRNA decay (ActD pulse-chase) transcripts revealed substantial overlap (Fig. 5c). Gene ontology (GO) enrichment analysis of ZFP36L2-dependent mRNA decay targets shared between the OKG146Li and MSK107Li datasets revealed significant enrichment in biological processes related to stress and stimulus responses (Fig. 5d). Notably, these pathways included responses to diverse stressors, including hypoxia (for example, *FOXO3*,* STC2* and* CA9*), TNF signalling (for example, *BIRC2*,* IL1B* and* FOSL2*) and apoptosis (for example, *CDKN1A*,* BNIP3L* and* RHOB*) (Supplementary Table 5c). As ActD can itself induce cellular stress, we used SLAM-seq^52^ to measure transcript half-lives after 4-thiouridine (4sU) labelling, which does not disrupt transcription. Consistent with ActD-mediated decay, *ZFP36L2* knockdown significantly stabilized transcripts associated with stress-response programs (Fig. 5e,f, Extended Data Fig. 12c and Supplementary Table 5e,f).

Dual mRNA FISH and ZFP36L2 IF for three putative ZFP36L2 mRNA targets (*GDF15*, *GADD45A* and *HILPDA*) in OKG146Li-MS2 organoids identified colocalization of a subset of these mRNAs with ZFP36L2 condensates (Fig. 5g). Acute exposure to growth factor withdrawal or irinotecan, conditions that induce ZFP36L2 condensate formation (Extended Data Fig. 9g,h), decreased the percentage of ZFP36L2 foci positive for target mRNA expression and the intensity of mRNA staining in ZFP36L2 foci (Fig. 5g,h and Extended Data Fig. 12d). Passaging organoids that had undergone long-term IGFF culture back into HISC medium, thus alleviating mitogen-deprivation-associated stress and inducing dedifferentiation (Figs. 2 and 3), promoted recovery of mRNA–ZFP36L2 colocalization (Fig. 5h and Extended Data Fig. 12d). We observed an inverse correlation between the intensity of ZFP36L2 and target mRNA staining per cell (Extended Data Fig. 12e,f). These data support a model in which stress-induced ZFP36L2 foci engage and promote rapid degradation of target mRNAs, which reduces their steady-state abundance and apparent colocalization. These observations spatiotemporally link ZFP36L2 condensate dynamics with its stress-response mRNA targets. That is, expression and colocalization are detectable at baseline and decrease during acute stress, consistent with dynamic mRNA degradation in ZFP36L2 condensates. This process reaches a minimum under prolonged growth factor stress conditions, is reversible after the reintroduction of growth factors and temporally coupled with dedifferentiation into an LGR5^+^ ISC state (Extended Data Fig. 12g).

## Discussion

We uncovered a pivotal role for the RNA-binding protein ZFP36L2 in linking epithelial stress responses with cellular plasticity. In association with AP-1 stress responses, ZFP36L2 binds to AU-rich 3′ UTRs of stress-associated mRNAs, which in turn mediates the formation of biomolecular condensates and the degradation of stress transcripts, thus terminating the stress response. This process subsequently triggers dedifferentiation into an ISC state, which enables epithelial regeneration and restoration of homeostasis. Loss-of-function mutations in *ZFP36L2* are enriched in metastatic CRC^14,53^ relative to non-metastatic tumours, and are associated with decreased recurrence-free survival^15^ and overall survival^53^ in patients with CRC. Our study provides a mechanistic explanation for the context-specific functions of ZFP36L2. In canonical primary tumours, in which the tumour mass is maintained through continual dedifferentiation into LGR5^+^ ISC states^6^, *ZFP36L2* mutations provide an initial selective disadvantage, manifesting as decreased tumour growth of canonical primary tumours (Fig. 3b). In metastasis, the *ZFP36L2* mutation-dependent inability to dedifferentiate from an LGR5^–^ injury–repair metastasis-initiating state into an LGR5^+^ ISC state confers an initial seeding disadvantage (Fig. 3c–g). However, this property selects for the outgrowth of metastases with non-canonical gene expression, which is associated with poor clinical outcomes^10^ (Fig. 3k–n). *ZFP36L2* mutations therefore act as a two-way molecular switch, blocking the entry of metastasis-initiating cells into canonical ISC state but at the same time triggering lineage plasticity into heterogeneous non-canonical states. Although this study focused on CRC, in which *ZFP36L2* mutations occur most frequently, *ZFP36L2* is also recurrently mutated in pancreatic cancer^54,55^, urothelial cancers^56^ and melanoma, and its paralogues *ZFP36L1* and *ZFP36* are mutated in other cancer types^15,57^. ZFP36L2 was recently identified in a screen for non-genetic drivers of acute myeloid leukaemia^58^. In this context, ZFP36L2 acts by degrading myeloid differentiation-associated mRNAs to maintain an undifferentiated leukaemic state. Moreover, the paralogue ZFP36L1 was identified as a regulator of neuroendocrine plasticity in a screen for LSD1 inhibitor sensitivity in small cell lung cancer^59^. These studies suggest that the function of the ZFP36L2 family in regulating differentiation and plasticity might be conserved across tissue lineages.

The dynamic interplay between differentiation and reprogramming underscores a broader paradigm in which cellular plasticity governs both tissue maintenance and malignant progression. In tissue repair, plasticity is tightly regulated and self-limiting. By contrast, metastatic progression reflects a failure of this stress ‘off switch’, which fuels sustained canonical or non-canonical differentiation programs that drive tumour growth and dissemination. Notably, our work identified a role for ZFP36L2 in maintaining the ISC pool even under homeostatic conditions. Moreover, ZFP36L2 binds a similar repertoire of stress-related mRNA transcripts in both normal and neoplastic cells but is dispensable for epithelial function at homeostasis. Basal ZFP36L2 expression in ISCs may reflect a preparedness to buffer transient stress-associated transcriptional programs and replenish stem cell pools during homeostasis^60–62^. By identifying ZFP36L2 as a crucial orchestrator of stress-induced cellular plasticity, this study opens many avenues for further investigation. Discerning the mechanisms that control context-dependent ZFP36L2 function—through regulation of its RNA binding and condensate formation—could lead to approaches that prevent or reverse plasticity into aggressive phenotypic states, metastasis and therapy resistance. The development of strategies to exploit these vulnerabilities could pave the way for new cancer treatments.

## Methods

### Patient biospecimens and data

All patient material was obtained through Memorial Sloan Kettering (MSK) Institutional Review Board protocols 06-107, 12-245, 14-244 and 22-404. No statistical method was used to predetermine sample sizes. scRNA-seq datasets from matched normal colon, primary tumour and metastasis samples were obtained from a previous study^10^. Patient-derived organoids from two primary tumours (MSK125P and OKG146P) and two metastases (MSK125Li and OKG146Li) were generated and validated as previously described^7,10^. Tumour whole-exome sequencing (WES) was performed by recapturing DNA processed originally for targeted exon sequencing using MSK-IMPACT^67^. The median WES target coverage for tumour and normal samples was 129× and 101×, respectively. WES data were analysed using the TEMPO pipeline (available from GitHub: https://github.com/mskcc/tempo). The OncoKB precision oncology knowledgebase, a US Food and Drug Administration (FDA)-recognized human genetic variant database curated by experts at MSK^68^, was used to distinguish between oncogenic alterations (presumed drivers) and variants of unknown significance (presumed passengers). Only somatic alterations labelled as oncogenic, likely oncogenic or predicted oncogenic by OncoKB were included for analyses. Archival formalin-fixed, paraffin-embedded (FFPE) ZFP36L2 WT and ZFP36L2 mutant clinical tissue blocks for immunostaining were identified through WES of corresponding tumour DNA originally collected for MSK-IMPACT. Tissue processing, FFPE section selection and histopathological data interpretation were overseen by an expert gastrointestinal pathologist (J.S.).

### scRNA-seq data analysis of CRC samples from patients

Normalized gene expression matrices were obtained from the Human Tumour Atlas Network (HTAN) Data Portal (http://humantumouratlas.org/publications/hta8_crc_moorman_2024) and processed as previously described^10^. Downstream analyses were restricted to patient-matched pairs of primary tumour and liver metastasis samples (*n* = 25 patients). After sample filtering, a total of 38,272 cells remained: primary tumour (13,102 cells), metastatic (12,215 cells) ISC (1,145 cells), absorptive precursor (2,805 cells), enterocyte (1,214 cells), BEST4^+^ enterocyte (1,339 cells), secretory precursor (3,818 cells), goblet (1,720 cells), tuft (751 cells) and enteroendocrine (163 cells). All subsequent differential expression, pathway analyses and correlation-based analyses were restricted to this filtered epithelial compartment.

#### Data visualization

Two-dimensional embeddings were generated using Scanpy (v.1.9.1). A *k*-nearest neighbours (KNN) graph was constructed on the principal components using Euclidean distance (*k* = 20), followed by visualization with a force-directed layout (ForceAtlas2). Subsequent plots were produced using Matplotlib (v.3.6.0)

#### Gene signature scores

Gene signature scores were calculated using the score_genes function from Scanpy (v.1.9.1), which calculates the average expression of a given gene set relative to matched reference genes. To reduce bias from differences in gene expression levels in each signature, we used* z*-scored expression data as input.

#### Generation of ISC-like cell annotations and gene signature

To identify tumour cells exhibiting an ISC-like transcriptional program, we first filtered the dataset to retain only malignant epithelial cells. Raw counts from these primary tumour and metastatic cells were normalized using total-count normalization followed by log-transformation. Highly variable genes (HVGs; *n* = 3,000) were selected for principal component analysis (PCA), neighbourhood graph construction (n_neighbours=20) and Leiden clustering.

To assess stemness expression across cells, we computed the median expression per cluster of ISC-specific marker genes from Hotspot-derived tumour ISC-like gene module 29 (Supplementary Table 1a), as previously identified^10^. The distribution of cluster-level ISC module scores was bimodal, which informed the selection of a threshold in identifying clusters that were ISC-like or not ISC-like. This tumour-derived ISC-like threshold value was then kept constant in labelling ISC-like normal cell types. We calculated a gene signature score on the *z*-normalized expression of these genes using the score_genes function in Scanpy (v.1.9.1) (Fig. 1b and Extended Data Fig. 1a).

#### DEG analysis between normal and tumour ISC-like cells

Differential gene expression (DEG) analysis was performed using the Wilcoxon rank-sum test as implemented in scanpy.tl_rank_genes_groups, comparing normal and tumour cells stratified by their ISC-like annotations. The analysis was conducted on the log-normalized gene expression matrix. We filtered for previously defined^10^ tumour ISC-like Hotspot gene module 29 genes and selected the most significant ones for downstream visualization (Fig. 1b). The log_2_ fold changes were computed relative to each gene across the specified cell-type groups.

To further characterize activity of the tumour ISC-like module, we computed the mean imputed expression of each gene in ISC module 29 across canonical ISC cells. Genes were then ranked by mean imputed expression to highlight ISC-enriched transcripts.

#### Computation of gene autocorrelation signatures in normal and tumour cells

We used Hotspot^69^ (v.0.9.1) to calculate pairwise local correlations between genes on the basis of the KNN graph, which captures local cell–cell similarity and genes with high local autocorrelation. This local strategy is also inherently more robust to gene dropouts and technical noise, which enables sensitive detection of context-specific gene co-expression patterns.

We first partitioned the data to only include tumour cells (25,317 cells) or normal cells (12,955 cells). HVGs (*n* = 2,000) were selected on the untransformed expression layer, with the manual inclusion of *ZFP36L2*. Genes prone to technical or non-informative variation (for example, ribosomal, mitochondrial and long non-coding RNAs) were excluded from the analyses, as were genes with non-zero counts across the dataset. A 50-nearest neighbour graph was constructed using the precomputed PCA embedding, and raw UMI counts were re-added to a desiccated counts layer. The resulting dataset was passed to Hotspot, using a depth-adjusted negative binomial model with the neighbourhood structure defined by a 15-nearest neighbourhood graph. Spatial autocorrelation scores (Moran’s I) were computed for all genes. Genes with a FDR < 0.05 were considered significant and retained for downstream analyses (Fig. 1i and Extended Data Fig. 1g).

#### GSEA

GSEA using GO biomolecular process, all Hallmark, PID and KEGG gene sets and Hotspot modules have been previously defined^10^ (Supplementary Table 1b). GSEA was performed using the prerank function of the Python package GSEApy (v.0.14.0) with 10,000 permutations and the default parameters.

#### Statistical analyses

Pairwise comparisons of PID–AP-1 pathway^65^ scores (Fig. 1j), Hotspot tumour ISC-like module 29 scores^10^ and *ZFP36L2* expression (Extended Data Fig. 1) were calculated across all cell-type groups by Mann–Whitney *U*-tests, and *P *values were adjusted for multiple testing using Bonferroni correction (*q *< 0.05).

### Mouse colon epithelial cell scRNA-seq data analysis

#### Data acquisition and preprocessing

scRNA-seq data (GSE168448)^66^ were downloaded from the Gene Expression Omnibus (GEO). Cells were first filtered on the basis of mitochondrial content, excluding those with mitochondrial transcript percentages exceeding 0.2. Outliers were identified using the median absolute deviation (MAD) method, following criteria adapted from previous studies^70,71^. A cell was classified as an outlier if it met at least two of the following five conditions: (1) log-transformed library size below 3 MADs under the median or above 5 MADs over the median; (2) log-transformed number of expressed genes below 3 MADs under the median or above 5 MADs over the median; (3) percentage of counts from the top 20 most highly expressed genes exceeding 5 MADs below or above the median; (4) feature-count distance (as previously defined^70^) exceeding 5 MADs below or above the median; and (5) percentage of mitochondrial counts exceeding 2.5 MADs above the median and greater than 8%. Same-sample doublets were identified and removed using scDblFinder (v.1.16.0)^72^. Outliers and doublets were excluded from downstream analyses.

#### Normalization, HVG selection, PCA and denoising

Count data were median-normalized and log-transformed with a pseudo-count of 1. The top 2,000 HVGs were selected using Scanpy^73^ (v.1.9.8) with the Seurat (v.3) method^74^. PCA was performed on these HVGs, retaining 280 principal components that accounted for 75% of the variance. Denoising was conducted using MAGIC^74–76^ via the Scanpy external API, using default parameters except for the number of principal components, for which all 280 retained components were used for neighbourhood calculations.

#### Cell typing

Cell-type annotation was performed by scoring the denoised expression of cell-type marker gene sets using Scanpy’s score_genes function, which reimplements a previously described method^77^. Each cell was assigned to the cell type for which it exhibited the highest score.

### Mouse experimental methods

#### Mouse strains

All animal procedures were approved by the Institutional Animal Care and Use Committee of the Memorial Sloan Kettering Cancer Center (MSKCC). The following mouse strains were obtained from The Jackson Laboratory: C57BL/6J (strain 000664); *Vil1*^*c*^^*re*^ mice^39^ (B6.Cg-Tg(Vil1-cre)1000Gum/J; stock 021504); *Lgr5*^*eGFP-IRES-creERT*^^*2*^ mice^16^ (B6.129P2-*Lgr5*^*tm1(cre/ERT2)Cle*^/J; stock 008875); and NSG mice (NOD.Cg-*Prkdc*^*scid*^*Il2rg*^*tm1Wjl*^/SzJ; stock 005557). *Zfp36l2*^*fl/fl*^ mice^40^ were provided by M. Turner and *Lgr5*^*DTR-eGFP*^ mice^5^ by F. de Sauvage. Primers and relevant information used for genotyping are listed in Supplementary Table 4b.

Xenograft experiments used only female NSG mice. All other experiments used male and female mice in equal proportions, with littermates allocated across groups to minimize variability. Sample sizes were not predetermined by statistical methods; group sizes (*n* = 5–15 per condition) were based on previous experience with similar xenograft and colitis models, keeping animal numbers to the minimum required under the 3Rs principles. Mice were randomly assigned to treatment groups, with blinding applied wherever feasible.

#### DSS-induced colitis

Experimental cohorts were generated by inbreeding on a C57BL/6J background, and all experiments included littermate controls. To evaluate the role of ZFP36L2 in colonic regeneration, *Zfp36l2*^*fl/fl*^ mice were crossed with *Vil1*^*cre*^ mice to generate intestinal epithelial knockout mice (*Zfp36l2*^*IEC*^). Mice (6–8 weeks old) received 3.5% (w/v) DSS (molecular weight 36–50 kDa; MP Biomedicals) in drinking water ad libitum for 7 days, followed by 7 days of regular water before euthanasia. Body weight was recorded daily. Caecal and colon tissues were collected at specified time points, processed as Swiss rolls, fixed in 4% paraformaldehyde and embedded in paraffin. Tissue sections were subjected to haematoxylin and eosin staining, immunostaining or FISH (see below).

#### Quantification of LGR5–eGFP by crypt flow cytometry

*Lgr5*^*eGFP-IRES-creERT2*^ mice (Jackson Laboratory, stock 008875) were crossed with *Vil1*^*cre*^ (stock 004586) and *Zfp36l2*^*fl/fl*^ mice to generate *Lgr5*^*e*^^*GFP-IRES-creERT2+*^*;Vil1*^*cre+*^;*Zfp36l2*^*IEC*^ mice. Littermate controls included *Lgr5*^*eGFP-IRES-creERT2*^^+^;*Vil1*^*cre+*^;*Zfp36l2*^*WT*^ *and Lgr5*^*eGFP-IRES-creERT2*–^;*Vil1*^*c*^^*re+*^*;Zfp36l2*^*WT*^ mice. For each experimental batch, three littermate pairs of *Lgr5*^*eGFP-IRES-creERT2*^^+^;*Vil1*^*cre+*^;*Zfp36l2*^*WT*^ and* Lgr5*^eGFP-IRES-creERT2+^;*Vil1*^*cre+*^;*Zfp36l2*^*IEC*^ mice (aged 6–8 weeks) were collected. Colons were dissected, flushed with PBS to remove faecal content, opened longitudinally and cut into around 1-cm segments. Tissue samples were incubated in dissociation buffer (PBS with 8 mM EDTA, 0.5 mM DTT and 10 U ml^–1^ DNase I (Roche 04716728001)) at 4 °C with gentle shaking for 60 min. Crypts were detached by vigorous shaking, pelleted and further dissociated with TrypLE (Thermo Fisher Scientific). The resulting cell suspension was treated with DNase at room temperature for 5 min and passed through a 40 μm strainer to generate single-cell suspensions for flow cytometry. Viable epithelial single cells were gated by forward scatter, side scatter and negative staining for 4′,6-diamidino-2-phenylindole (DAPI). *Lgr5*^*eGFP-IRES-creERT2*^^–^;*Vil1*^*cre+*^*;Zfp36l2*^*WT*^ mice were processed in parallel as gating controls. Two independent experimental batches were performed, and data were pooled for analyses.

#### In vivo *Lgr5*^*DTR*^ ablation

Mice were bred to generate *Lgr5*^*DTR-eGFP+*^ and littermate *Lgr5*^*DTR-eGFP−*^ controls*. Lgr5*^*DTR-eGFP+*^*ZFP36L2*^*IEC*^ mice were generated by crossing *Lgr5*^*DTR-eGFP+*^*;Vil1*^cre+^ and* ZFP36L2*^*fl/fl*^ together with* Lgr5*^*DTR-eGFP+*^;*Vil1*^cre−^ and * ZFP36L2*^*fl/fl*^ to generate littermate controls. For in vivo depletion of *Lgr5*^*DTR*^-expressing cells, DT (0.005 μg μl^–1^ in PBS) was administered intraperitoneally at a dose of 0.05 μg per g of body weight every 48 h, for a total of 4 doses. Mice aged 8–10 weeks were used for all experiments. Colons were collected at the time points indicated in the figures or figure legends, Swiss-rolled and fixed in 4% paraformaldehyde for 24 h at room temperature. Tissues were then transferred to 70% ethanol and stored at 4 °C before paraffin embedding.

#### In vitro *Lgr5*^*DTR*^ ablation and dedifferentiation assay

Colon organoids were established from *Lgr5*^*DTR-eGFP+*^;*Vil1*^cre+^;*ZFP36L2*^*fl/fl*^ (*Lgr5*^*DTR-eGFP+*^*ZFP36L2*^*IEC*^) and *Lgr5*^*DTR-eGFP+*^*;Vil1*^cre−^;*ZFP36L2*^*fl/fl*^ (*Lgr5*^*DTR-eGF*P*+*^*ZFP36L2*^*W*T^) mice (6–8 weeks old; *n* = 3 mice per genotype). Approximately 1,000 crypts, isolated as described above, were suspended in 40 μl Matrigel and cultured in mouse WRENAFI (mWRENAFI) medium containing advanced DMEM/F12 (AdDF12; Thermo Fisher Scientific), 2 mM GlutaMAX (Thermo Fisher Scientific), 10 mM HEPES (Thermo Fisher Scientific), 1 mM *N*-acetyl-l-cysteine (Sigma-Aldrich), 1:50 B27 supplement (Thermo Fisher Scientific), 1:100 N2 supplement (Thermo Fisher Scientific) and 100 μg ml^–1^ Pimocin (InvivoGen), supplemented with growth factors and the following inhibitors: 50 ng ml^–1^ EGF (Peprotech), 100 ng ml^–1^ murine Noggin (Peprotech), 500 nM A8301 (Sigma-Aldrich), 50 ng ml^–1^ FGF2 (Peprotech), 100 ng ml^–1^ IGF-I (Peprotech), 1 nM NGS-WNT (ImmunePrecise N0001) and 1 μg ml^–1^ murine R-spondin1 (Peprotech). After stable organoid lines were established, organoids were dissociated into single cells and seeded at 20,000 cells per 40 μl Matrigel. Cells were cultured in mWRENAFI medium for 4 days, with medium changed every 2 days. On day 4, cultures were switched to fresh mWRENAFI medium with or without 0.6 μg ml^–1^ DT for 24 h. Organoids were then dissociated into single cells, and depletion of LGR5–eGFP^+^ cells in the DT-treated group was confirmed by flow cytometry using organoids derived from C57BL/6J mice as a gating control. Live DAPI-negative cells were sorted, embedded in Matrigel at 2,000 cells per 40 μl and cultured in mWRENAFI medium for 5 days to induce dedifferentiation. Regenerated organoid numbers were quantified using BioTek bright-field imaging with whole-dome* z*-projection, and eGFP^+^ cells from each group were quantified by flow cytometry.

#### Orthotopic xenograft experiments

NSG mice (Jackson Laboratory, stock 005557) were used for transplantation experiments at 6 weeks of age. Mice were housed in a specific pathogen-free facility under controlled temperature and humidity, with a 12-h light–dark cycle and ad libitum access to either standard chow or an irradiated diet supplemented with 2,500 ppm doxycycline (Modified LabDiet 5053), along with water.

For orthotopic caecal and intrasplenic injections, organoid lines expressing thymidine kinase–eGFP–luciferase and transduced with pTRIPZ lentivirus expressing doxycycline-inducible shRNAs targeting *ZFP36L2* or a Renilla control were transduced with lentivirally expressed pLenti-PGK-Akaluc. Transplantation procedures were performed as previously described^7,10^. For generation of the OKG146Li-MS2 line, OKG146Li organoids were transduced with pLenti-PGK-Akaluc, and around 500,000 cells were injected orthotopically into the mouse liver in 50% Matrigel as previously described^10^ to generate liver tumours. At the end point, tumours were collected, minced and plated in HISC medium containing antibiotics to select for CRC organoids. This in vivo selection procedure was repeated twice to generate the liver-metastasis-selected OKG146Li-MS2 organoid line. For caecal injections, 5 × 10^5^ cells from canonical primary tumour organoids (MSK125P and OKG146P) or liver metastasis (OKG146Li and MSK107Li) organoids, representing canonical and non-canonical cell states, respectively, transduced with lentivirus expressing eGFP–luciferase and either shZFP36L2 or shCtrl were resuspended in 10 μl (50% Matrigel, 50% PBS) and injected into the caecal submucosa of 6-week-old NSG mice. Mice were monitored by BLI 1 week after injection to confirm primary tumour engraftment. Any BLI-signal-negative animals were excluded from subsequent analyses. Mice were then started on a doxycycline diet with weekly BLI monitoring for 15 weeks before euthanasia and organ collection. For splenic vein injections, 6-week-old NSG mice were placed on a doxycycline diet 24 h before surgery. CRC liver-metastasis-derived organoids MSK107Li and OKG146Li-MS2 transduced with lentivirus expressing eGFP-luciferase and either shZFP36L or shCtrl were treated with doxycycline ex vivo for 7 days before surgery and injected into the spleens of mice (5 × 10^5^ cells per mouse). Around 500,000 cells were injected into the splenic vein in 50 μl PBS supplemented with 10% Matrigel, immediately followed by splenectomy. BLI was performed immediately after surgery to establish a post-injection baseline (week 0). Mice were maintained on doxycycline diet for 10–15 weeks until they reached the humane end point.

BLI was performed immediately after surgery and weekly thereafter using an IVIS Spectrum Xenogen system (Caliper Life Sciences). Data were analysed using Living Image (v.2.50) software. Experimental group sizes (*n* ≥ 5 mice per group) were determined based on practical constraints (five mice per cage) and were age-matched and sex-matched.

Mice with caecal or intrasplenic tumours were monitored daily. Humane end points were defined as the onset of distress, including hunched posture, impaired grooming or weight loss of >20%, together with a route-specific criterion: a maximum experimental duration of 6 months for caecal tumours, or tumour burden exceeding 10% of body mass and/or a body condition score <2 for intrasplenic tumours (with ascites as an additional distress sign specific to intrasplenic tumours). These criteria were not exceeded in any experiment. Animals were euthanized at the end point, and tissues were collected for further analyses. Where indicated, tissues were fixed in 4% paraformaldehyde for 24 h. Histopathological scoring was performed in a blinded manner.

### Mouse organoid dedifferentiation assay

Three mice from each genotype (aged 6–8 weeks) were euthanized, and colons were dissected and processed to collect crypts as described above. Approximately 1,000 crypts were suspended in 40 μl Matrigel and cultured in mWRENAFI medium, consisting of advanced DMEM/F12 (AdDF12; Thermo Fisher Scientific), 2 mM GlutaMAX (Thermo Fisher Scientific), 10 mM HEPES (Thermo Fisher Scientific), 1 mM *N*-acetyl-l-cysteine (Sigma-Aldrich), 1:50 B27 supplement (Thermo Fisher Scientific), 1:100 N2 supplement (Thermo Fisher Scientific) and 100 μg ml^–1^ Primocin (InvivoGen), supplemented with growth factors and the following inhibitors: 50 ng ml^–1^ EGF (Peprotech), 100 ng ml^–1^ murine Noggin (Peprotech), 500 nM A8301 (Sigma-Aldrich), 50 ng ml^–1^ FGF2 (Peprotech), 100 ng ml^–1^ IGF-I (Peprotech), 1 nM NGS-WNT (ImmunePrecise N0001) and 1 μg ml^–1^ murine R-spondin1 (Peprotech). Once stable organoid lines were established, 2,000 single cells from the organoids were plated in 40 μl Matrigel and cultured in mWRENAFI medium supplemented with 10 μM Y-27632 for 3 days. The medium was then switched to mENAFI (removing NGS-WNT and R-spondin1) to induce organoid differentiation for 2 days. Efficient loss of LGR5^+^ ISCs under these differentiation conditions was confirmed by flow cytometry analysis of GFP signal in LGR5–eGFP^*+*^ organoids cultured under the same conditions. The differentiated mature organoids were dissociated into single cells using TrypLE, and viable cells were sorted and plated at 2,000 cells per 40 μl Matrigel domes to evaluate dedifferentiation via organoid formation. Five to six technical replicates from three biological replicates were pooled for subsequent analysis and plotting.

### Multiplexed staining and imaging

#### Organoid collagen embedding

Day 7 patient-derived organoids (PDOs) from patients with CRC were recovered from Matrigel as described above. Intact organoids were resuspended in neutralized collagen I (250 μl collagen I solution mixed with 28.4 μl of 10× PBS and 5.7 μl of 1 M NaOH) and deposited into a tissue capsule in a 6-well plate to form small domes. Once the collagen I droplets solidified in the incubator for 30 min, the domes were covered with tissue culture medium and cultured overnight.

The medium was aspirated the following day, and the tissue capsule was washed two to three times with PBS until the solution appeared clear, free of residual medium. The tissue capsule was then transferred to freshly prepared 4% paraformaldehyde for 24 h with gentle agitation at room temperature. After fixation, the capsule was washed with PBS and stored in 70% ethanol at 4 °C until paraffin embedding.

#### FISH

Paraffin-embedding and tissue sectioning were performed by the MSKCC Molecular Cytology Core. Paraffin-embedded tissue sections (5 μm) were stored at 4 °C until use. Sections were processed on a Leica Bond RX stainer, baked for 30 min at 60 °C, dewaxed using Bond Dewax solution (Leica, AR9222) and subjected to EDTA-based epitope retrieval (Leica, AR9640) for 15 min at 95 °C.

For single FISH, RNA probes (Advanced Cell Diagnostics, ready-to-use) were hybridized to the tissue sections for 2 h at 42 °C. Probe detection was performed using a RNAscope 2.5 LS Reagent kit–Brown (ACD, 322100) following the manufacturer’s instructions, with the modification of substituting DAB application with Fluorescent CF594/Tyramide (Biotium, 92174) for 20 min at room temperature. After washing in PBS, slides were incubated with 5 μg ml^–1^ DAPI (Sigma-Aldrich) in PBS for 5 min, rinsed and mounted using Mowiol 4–88 (Calbiochem).

For double FISH, a mixture of two probes (hLGR5-C1 and hZFP-C2) was hybridized to 5-μm FFPE tissue sections at 42 °C for 2 h. The hybridized probes were detected using a RNAscope LS Multiplex Reagent kit (ACD, 322800) according to the manufacturer’s protocol. Fluorescence detection was achieved by incubating the slides with Alexa Fluor 488 Tyramide (Life Technologies, B40953) and CF594 Tyramide (Biotium, 92174) for 30 min at room temperature. After PBS washing and DAPI incubation (5 μg ml^–1^ for 5 min), slides were mounted in Mowiol 4–88.

The following RNAscope 2.5 LS probes were used in this study: *Hs-ZFP36L2* (581268), *Mm-Zfp36l2* (518908), *Mm-Lgr5* (312178), *Hs-LGR5* (311028), *Hs-GDF15* (600308), *Hs-HILPDA* (501958), *Hs-GADD45A* (477518), positive control probe_*Hs-PPIB* (313908) and negative control probe_*dapB* (312038).

#### Multiplex FISH with IF

After FISH staining, slides were scanned, and coverslips were carefully removed. The slides were then processed following the standard IF protocol (as detailed below).

#### Single IF staining

For single IF, 5-μm FFPE tissue sections were loaded onto a Leica Bond RX system and dewaxed as the first step. Epitope retrieval was performed using EDTA-based ER2 solution (Leica, AR9640) for 20 min at 100 °C. Primary antibodies against each target were incubated for 1 h at room temperature. Following primary antibody incubation, slides were incubated with Leica Bond Polymer anti-rabbit HRP (included in the Polymer Refine Detection kit, Leica, DS9800) for 8 min. For pan-cytokeratin staining, after primary antibody incubation, slides were incubated with Leica Bond Post-Primary reagent (rabbit anti-mouse linker, included in the Polymer Refine Detection kit, Leica, DS9800) for 8 min, followed by an additional 8 min of incubation with Leica Bond Polymer anti-rabbit HRP (included in the Polymer Refine Detection kit, Leica, DS9800). Signal detection was performed using either Alexa Fluor Tyramide signal amplification reagents (Life Technologies, B40953, B40958) or CF dye Tyramide conjugates (Biotium, 92172, 96053, 92174). After IF staining, slides were washed in PBS and incubated with 5 μg ml^–1^ DAPI (Sigma Aldrich) in PBS for 5 min, rinsed in PBS and mounted using Mowiol 4–88 (Calbiochem). Slides were stored overnight at –20 °C before imaging.

#### Multiplex IF staining

For multiplex IF, antibody staining and detection were performed sequentially according to the single IF staining procedures. Secondary antibody detection was performed using Leica Bond Post-Primary rabbit anti-mouse linker (Leica, DS9800) for human samples stained with mouse-derived primary antibodies, or with rabbit anti-mouse secondary antibody linker (Abcam, ab133469) for mouse samples stained with mouse-derived primary antibodies, followed by Leica Bond Polymer anti-rabbit HRP (Leica, DS9800). After each round of IF staining, epitope retrieval was carried out to denature the primary and secondary antibodies before applying the next primary antibody. After the final staining round, slides were washed in PBS, incubated with DAPI, rinsed, and mounted in Mowiol 4–88 (Calbiochem). Slides were stored at –20 °C overnight before imaging.

The following primary antibodies were used for IF: ZFP36L2 (mouse, 200 µg ml^–1^, Santa Cruz, sc-365908); MUC2 (rabbit, 0.0625 μg ml^–1^, Abcam, ab97386); pan-cytokeratin (mouse, 0.125 μg ml^–1^, Abcam, ab8068); KI67 (rabbit, 0.25 μg ml^–1^, Abcam, ab15580); CK20 (rabbit, 1 μg ml^–1^, Novus Biologicals, NBP3-03605); CK5 (rabbit, 0.016 μg ml^–1^, Cell Signaling Technology, 81817); CHGB (rabbit, 0.1 μg ml^–1^, Invitrogen, PA5-52605); DDX6 (rabbit, 0.517 mg ml^–1^, Abcam, ab307418); G3BP1 (mouse, 1 mg ml^–1^, Abcam, ab56574) and PABPC1 (rabbit, 1 mg ml^–1^, Abcam, ab21060). The following secondary antibodies were used for IF: Leica Bond Post-Primary (rabbit anti-mouse linker) (Leica, DS9800) for staining human samples with mouse-derived antibodies, rabbit anti-mouse secondary antibody linker (Abcam, ab133469) for staining mouse samples with mouse-derived antibodies, Leica Bond Polymer anti-rabbit HRP (Leica, DS9800).

#### FISH and IF imaging and quantification

FISH or IF slides were scanned on a Pannoramic scanner (3DHistech) using a ×40/0.95NA objective.

#### Mouse crypt FISH and IF multiplex quantification

A total of 31 crypts were drawn and exported as TIFF files from these scans using Slide Viewer (3DHistech). These images were then analysed using ImageJ/FIJI (NIH). Thresholding and Watershedding were used to segment the cells using the DAPI channel. The *Lgr5* channel was thresholded and the mask was used to create a distance map. Then, the segmented cells were overlaid onto the distance map and thresholded images to determine their positivity for each marker and their distance from *Lgr5*.

#### Tumour marker gene FISH and IF quantification

ROIs around tissues were drawn and exported as TIFF files from scans using Slide Viewer (3DHistech). Images were then analysed using ImageJ/FIJI (NIH). Thresholding and Watershedding were used to segment cells using the DAPI channel. Each marker was thresholded to measure the area and intensity of the fluorescent signal and to classify positivity for each marker per cell.

### Patient-derived CRC organoid experimental methods

#### CRC organoid culture

Organoids were cultured and expanded in HISC medium comprising advanced DMEM/F12 (ADF12; Thermo Fisher Scientific), 2 mM GlutaMAX (Thermo Fisher Scientific), 10 mM HEPES (Thermo Fisher Scientific), 1 mM *N*-acetyl-l-cysteine (Sigma-Aldrich), 1:50 B27 supplement (Thermo Fisher Scientific), 1:100 N2 supplement (Thermo Fisher Scientific) and 100 μg ml^–1^ Primocin (InvivoGen), supplemented with growth factors and inhibitors, including 50 ng ml^–1^ EGF (Peprotech), 100 ng ml^–1^ Noggin (Peprotech), 500 nM A8301 (Sigma-Aldrich), 50 ng ml^–1^ FGF2 (Peprotech) and 100 ng ml^–1^ IGF-I (Peprotech). Where indicated, organoids were cultured in IGFF containing all base components of the medium but lacking EGF, Noggin, A8301, FGF2 and IGF. Medium was supplemented with 2 μg ml^–1^ doxycycline where indicated. Medium was refreshed every 2 days. Human organoid lines used in this study were verified by short tandem repeat analysis at the time of establishment and before each experiment. All organoid lines used in this study were routinely tested for mycoplasma contamination using a MycoAlert PLUS Detection kit (Lonza), and all tested negative.

For downstream applications, organoids were collected on day 7 by dissolving Matrigel with 2 mM EDTA in DPBS, followed by orbital agitation at 4 °C for 30–60 min. Organoids were then dissociated into single cells using TrypLE (Thermo Fisher Scientific) for 5–10 min at 37 °C. The reaction was quenched by adding 5 volumes of wash buffer (DMEM/F12 supplemented with 2 mM GlutaMAX, 10 mM HEPES, 100 U ml^–1^ penicillin–streptomycin (Thermo Fisher Scientific) and 5% FBS (Sigma-Aldrich)), and the cell suspension was filtered through a 40 μm cell strainer. For passaging, cells were embedded at 375 cells per μl Matrigel. For organoid formation and IC_50_ assays, cells were seeded at 50 cells per μl Matrigel. Matrigel domes were incubated at 37 °C for 30 min to solidify before adding culture medium. Lentiviral transduction was performed as previously described^42^, and antibiotic selection was initiated after the first passage post-transduction using 2 μg ml^–1^ puromycin, 15 μg ml^–1^ blasticidin and 400 μg ml^–1^ geneticin (Thermo Fisher Scientific).

#### Plasmids

For doxycycline-inducible knockdown of *ZFP36L2*, miR-30a-based shRNA sequences (RHS4430-200267269 and RHS4430-200276644, pGIPZ) were subcloned into the pTRIPZ lentiviral vector using XhoI and MluI restriction sites. The resulting constructs encoded the following inducible mature antisense sequences: 5′-TGCACAAGAAGTCGACATC-3′ and 5′-TGTTGAGCAGGGCTGTGCC-3′. Lentiviral particles were produced as previously described^78^ in HEK293T cells (American Type Culture Collection) using a non-targeting control vector (Addgene, 127696). For *ZFP36L2* knockout, the doxycycline-inducible Cas9 vector TLCV2 (Addgene, 87360) was cloned with either sgRNA1 (5′-ACCCTTAAGGAGCCGTCGGG-3′) or sgRNA2 (5′-TGCTGGCCGAGTGCCGTCGG-3′) targeting *ZFP36L2* to generate indel-mediated gene disruption. These constructs were delivered to organoids by lentiviral transduction. Transduced organoids were selected using puromycin and treated with 2 μg ml^–1^ doxycycline for 1 week before sorting eGFP^+^ cells. Sorted cells were seeded at 2,000 cells per 40 μl Matrigel dome and cultured in the continued presence of doxycycline. On day 10, individual organoid clones were picked, dissociated and expanded as single clones. *ZFP36L2* knockout was confirmed by western blot analysis. For inducible protein expression, the coding sequence of WT human *ZFP36L2* was PCR-amplified from organoid cDNA, in-frame fused to an eGFP or meGFP tag and cloned into AgeI-digested and NheI-digested TLCV2 vector (Addgene, 87360) via Gibson assembly using 20-bp homologous overlaps. A frameshift mutant (fsZFP36L2) was generated using a similar approach by dividing the *ZFP36L2* coding sequence into two fragments (nucleotides 1–429 and 433–1359) for Gibson assembly. A single-nucleotide deletion (guanine deletion at codon Gly143) was introduced on the primer through site-directed mutagenesis. Lentiviral particles carrying doxycycline-inducible WT ZFP36L2–eGFP, ZFP36L2–meGFP, fsZFP36L2–eGFP or eGFP-only control were produced for downstream experiments. To generate ADAR-fusion constructs for HyperTRIBE, the hyperactive catalytic domain of *Drosophila melanogaster* ADAR was PCR-amplified from a plasmid template (Addgene, 166969) and inserted in-frame into the doxycycline-inducible WT and fsZFP36L2 expression constructs. The resulting fusion constructs encoded ADAR-tagged ZFP36L2 or fsZFP36L2, followed by a self-cleaving T2A peptide linked to eGFP (Extended Data Fig. 11a). Lentiviral particles were generated and used to transduce organoids. For nuclear labelling during live-cell imaging, organoids were stably transduced with mCherry-tagged histone H2B in a pLenti6/V5-DEST backbone (Addgene, 89766). All the plasmid sequences are listed in Supplementary Table 4a.

#### Western blotting

Approximately 2 million organoid cells were recovered from Matrigel by disrupting domes in 2 mM EDTA in DPBS with orbital shaking at 4 °C for 30 min. Cells were washed, centrifuged (500*g*, 5 min, 4 °C) and lysed in 1× RIPA buffer (50 mM Tris-HCl, pH 8.0, 150 mM NaCl, 0.1% SDS, 0.5% sodium deoxycholate and 1% NP-40) supplemented with protease inhibitors (Roche, complete EDTA-free protease inhibitor cocktail) and phosphatase inhibitors (Sigma-Aldrich, 524636-1SET) and benzonase nuclease (2% v/v; Thermo Fisher Scientific, 70-664-3) for 30 min on ice. Lysates were sonicated using a Bioruptor (10 s on–10 s off for 10 cycles at 4 °C). The protein concentration was determined using a Pierce BCA Protein Assay kit (Thermo Fisher Scientific, 23227).

For immunoblotting, 50 μg of total protein per sample was separated by SDS–PAGE on Bis–Tris polyacrylamide gels (Thermo Fisher Scientific), transferred to methanol-activated 0.45 μm PVDF membranes (Millipore, IPFL00010) and blocked with 5% non-fat milk in TBST for 30 min at room temperature. Membranes were incubated overnight at 4 °C with the following primary antibodies: mouse anti-β-actin (1:2,000; Abcam, ab8226), anti-GAPDH (1:2,000; Abcam, ab181602) and anti-ZFP36L2 (1:200; Santa Cruz, clone A-3, sc-365908).

After washing, membranes were incubated with HRP-conjugated anti-mouse or anti-rabbit secondary antibodies (1:10,000; Cell Signaling Technology, 7076 for mouse and 7074 for rabbit). Signals were detected using an iBright CL1000 imaging system (Invitrogen) with SuperSignal West Femto Maximum Sensitivity substrate (Thermo Fisher Scientific).

#### Reverse transcription and qPCR

Total RNA was extracted from organoids using a RNeasy Mini kit (Qiagen) according to the manufacturer’s instructions. cDNA was synthesized from 2 μg of total RNA using a Transcriptor First-Strand cDNA Synthesis kit (Roche). qPCR was performed using TaqMan gene expression assay primers (*ZFP36L2*: Hs00272828_m1; *GAPDH*: Hs02758991_g1, Mm99999915_g1; *ACTB*: Hs01060665_g1; Thermo Fisher Scientific) on an ABI QuantStudio 7 Pro Real-Time PCR system (Applied Biosystems). Relative expression was computed using Design & Analysis software (v.2.6.0) using the ${2}^{-\Delta \Delta {C}_{t}}$ method and normalized to *GAPDH* or *ACTB* expression.

### CRC organoid dedifferentiation and scRNA-seq

OKG146P organoids expressing shZFP36L2 or shCtrl were cultured initially in HISC (with 2 μg ml^–1^ doxycycline) for 7 days, and live cells were collected for scRNA-seq. A portion of the same batch of organoids was then passaged into IGFF medium (with 2 μg ml^–1^ doxycycline) for 7 days to induce differentiation as previously described^10^. Organoids were then passaged into complete HISC medium (with 2 μg ml^–1^ doxycycline) for an additional 7 days to induce dedifferentiation into an ISC-like state, followed by scRNA-seq. Organoids continuously maintained in complete HISC medium (with 2 μg ml^–1^ doxycycline) for 7 days served as controls.

Organoids were dissociated into single cells as described above. For sample multiplexing, single-cell suspensions were incubated with TotalSeq hashtag antibodies (BioLegend, B0251, B0252, B0253) for 30 min on ice. Viable (DAPI-negative) cells were sorted using a 130 μm nozzle on a SH800S (Sony) cell sorter and collected into DPBS containing 0.04% BSA. Equal numbers of hashtag-labelled shZFP36L2 and control cells from each condition were pooled for scRNA-seq using a Chromium Single Cell 3′ (v.3.1) platform (10x Genomics) according to the manufacturer’s protocol. FACS-sorted cells were washed with DPBS + 0.04% BSA and resuspended at 700–1,300 cells per μl. Cell viability was above 90%, as determined by 0.2% Trypan Blue exclusion. Up to 10,000 cells per sample were targeted for droplet-based encapsulation and barcoding. After reverse transcription, emulsions were broken and cDNA was purified using Dynabeads MyOne SILANE, followed by PCR amplification. Libraries were sequenced on an Illumina NovaSeq S4 platform using the following read configuration: read 1, 28 cycles; i7 index, 8 cycles; read 2, 90 cycles.

#### Data processing, quality control and batch correction

Raw scRNA-seq data were pre-processed to remove ambient RNA contamination using CellBender (v.1.0.0). Doublets were identified and excluded using DoubletDetection (v.3.0). Cells with log-transformed library sizes deviating by more than 3 MADs below or 5 MADs above the median were considered outliers and removed. Additional quality control metrics, including total UMI counts, number of detected genes, mitochondrial gene fraction and ribosomal gene fraction, were computed per cell. Cells with fewer than 500 total counts, fewer than 400 detected genes, greater than 20% mitochondrial content or otherwise poor-quality metrics were excluded. Hashtag-related genes were removed to eliminate multiplexing artefacts.

#### Normalization and batch correction

After quality filtering, normalization was performed using Scran (v.3.20), and log-transformed expression values were generated. The dataset was aggregated for downstream analyses. Batch correction was performed using single-cell variational inference (scVI)^79^. A raw count matrix was input, and Seurat (v.3) was used to identify 5,000 HVGs. The scVI model was initialized with 100 latent dimensions, incorporating mitochondrial content as a covariate.

#### Dimensionality reduction and clustering

PCA was performed, retaining 280 principal components that explained 75% of the variance. Cells were clustered using the Leiden algorithm (resolution of 1.8), and UMAP embeddings were generated for visualization. Differential gene expression analysis was performed using the Wilcoxon rank-sum test.

#### Local variability analysis

To assess local variability in cell states, KNN entropy was computed by calculating the Shannon entropy of *ZFP36L2* expression in each cell’s 15-nearest neighbour graph. Entropy distributions across experimental groups (for example, shZFP36L2 versus shCtrl) were visualized using boxplots. Kernel density estimation plots were also used to visualize expression variability in low-dimensional space.

#### Differential abundance analysis

To evaluate shifts in cell population composition, MELD^80^ was used to partition the UMAP embedding into transcriptionally similar neighbourhoods. The method quantified relative differences in the abundance of these regions between conditions (for example, shZFP36L2 versus shCtrl).

#### Cell-state classification

Cell-state classification was performed using PhenoGraph^81^, which integrates graph-based clustering with phenotypic similarity. An affinity matrix was constructed based on nearest-neighbour relationships, and batch effects were corrected using Harmony^82^. Cell-state labels were assigned to organoid cells by propagating annotations from reference patient-derived cell states.

### Bulk RNA-seq

Total RNA was extracted from organoids with conditions specified in the figure legends using a RNeasy Mini kit (Qiagen) according to the manufacturer’s instructions. Three technical replates for each sample were prepared for all the experiments. After RiboGreen quantification and quality control by Agilent BioAnalyzer, 300 ng of total RNA with RIN values of 9.8–10 underwent polyA selection and TruSeq library preparation according to instructions provided by Illumina (TruSeq Stranded mRNA LT kit, RS-122-2102), with 8 cycles of PCR. Samples were barcoded and run on NovaSeq 6000 in a PE100 flowcell, using a NovaSeq 6000 S4 NovaSeq Reagent kit (200 Cycles) (Illumina). An average of 109 million paired reads was generated per sample. Ribosomal reads represented 0.4–0.7% of the total reads generated and the per cent of mRNA bases averaged 92%.

#### Bulk RNA-seq data analysis

Adapter sequences were trimmed using Cutadapt (v.4.8), and processed reads were aligned to the human reference genome (hg38) using STAR (v.2.7.11b). Gene-level read counts were quantified using HTSeq (v.2.0.5), and differential expression analysis was performed using DESeq2 (v.1.42.0).

#### HyperTRIBE data analysis

WT ZFP36L2 or fsZFP36L2 was expressed as a fusion with the hyperactive E488Q mutant catalytic domain of ADAR, which enables the detection of ZFP36L2-interacting mRNAs through capture of adenosine (A) to inosine (I, read as guanosine (G)) edits through RNA-seq^48^. The construct design is shown in Extended Data Fig. 11a. Following stable lentiviral integration, organoids were seeded as single cells, cultured in HISC medium for 3 days and then treated with doxycycline for 4 days before flow sorting for eGFP^+^ (ADAR fusion protein expressing) cells and RNA-seq. Day 5 organoids stably transduced with doxycycline-inducible ZFP36L2–ADAR or fsZFP36L2–ADAR constructs were treated with 2 μg ml^–1^ doxycycline for 2 days to induce transgene expression. eGFP^+^ live cells were isolated by flow cytometry, and total RNA was extracted using a RNeasy Mini kit (Qiagen). mRNA libraries were prepared and sequenced as described above. ADAR-seq data were processed as previously described^49^. In brief, adapter sequences were trimmed using Cutadapt (v.4.8), and reads were aligned to the hg38 genome and construct sequences using STAR (v.2.7.11b). RNA editing sites were identified using GATK HaplotypeCaller (v.3.8.1.0), with stringent filtering to retain only A-to-G substitutions on the forward strand and U-to-C substitutions on the reverse strand. Known variants annotated in dbSNP (human_9606_b144_GRCh38p2) were excluded. The editing frequency at each site was assessed using a Beta-binomial test, with multiple testing correction performed using the Benjamini–Hochberg method.

#### Gene decay rate comparison

PDO MSK107Li and OKG146Li organoids expressing shCtrl or shZFP36L2 were cultured as described above, supplemented with 2 μg ml^–1^ doxycycline. On day 5, organoids were treated with or without 5 μg ml^–1^ ActD for 5 h. Organoids were then collected, and total RNA was extracted using a RNeasy Mini kit (Qiagen). mRNA libraries were prepared and sequenced as described above.

Adapter sequences were trimmed using Cutadapt (v.4.8), and processed reads were aligned to the human reference genome (hg38) using STAR (v.2.7.11b). Gene-level read counts were quantified using HTSeq (v.2.0.5), and differential expression analysis was performed using DESeq2 (v.1.42.0) to compare the 5-h-treated versus untreated samples for shCtrl or shZFP36L2 separately to capture gene decay rates in each condition. To compare decay rates between shCtrl and shZFP36L2 experiments, gene-level read counts were normalized globally for both shCtrl and shZFP36L2 cells, with and without treatment. The decay rate dependency on ZFP36L2 for each gene was calculated using the following formula:

$$\mathrm{Gene}\times \mathrm{ZFP}36{\rm{L}}2\,\mathrm{dependency}={\log }_{2}\left[\frac{\mathrm{gene}\times \mathrm{mean}\,\mathrm{count}\,\mathrm{in}\,\mathrm{shZFP}36{\rm{L}}2(\mathrm{treated})/\mathrm{shZFP}36{\rm{L}}2(\mathrm{untreated})}{(\mathrm{gene}\times \mathrm{mean}\,\mathrm{count}\,\mathrm{in}\,\mathrm{shCtrl}(\mathrm{treated})/\mathrm{shCtrl}(\mathrm{untreated})}\right]$$

#### Thiol-linked alkylation for the metabolic sequencing of RNA

To measure RNA decay kinetics, we performed SLAM-seq^52^ in CRC organoids using a SLAM-seq Kinetics kit–Catabolic Kinetics Module (Lexogen) followed by library preparation with a QuantSeq 3′ mRNA-seq V2 Library Prep kit FWD with Unique Dual Indices (Lexogen). The SLAM-seq workflow is based on metabolic labelling of newly synthesized RNA with 4sU, followed by alkylation of 4sU-containing RNA and detection of T > C conversions after sequencing. For all steps from 4sU handling up until library preparation, samples and reagents were protected from white light and handled in the dark or wrapped in foil whenever possible, as recommended by the manufacturer because 4sU is light sensitive and can crosslink.

We first performed an organoid viability titration to determine a non-toxic working concentration of 4sU. shCtrl-expressing MSK107L organoids were dissociated and seeded in 96-well plates as 40 μl Matrigel domes containing 2,000 cells per well in HISC medium. On day 6, organoids were treated with a range of 4sU concentrations, with medium replaced every 2 h, and cell viability was measured after 13 h using CellTiter-Glo assays. The concentration causing 10% growth inhibition over the assay window was selected for the final experiment, as per the manufacturer’s recommendations. On the basis of this titration, 500 μM 4sU was used for all subsequent labelling experiments.

For the catabolic labelling experiment, organoids were labelled with 500 μM 4sU for 6 h, with fresh 4sU-containing medium supplied every 2 h to maintain labelling efficiency. At the end of labelling, one set of samples was collected immediately as the* t*_0_ time point. To measure mRNA decay kinetics, 4sU-containing medium was removed and organoids were washed by adding 3 ml warm PBS and incubating for 10 min to allow residual labelling medium to equilibrate out. The PBS was then replaced with HISC medium containing 50 mM uridine and samples were collected at 2 h, 5 h and 12 h. At each time point, the medium was removed completely and organoids were lysed directly in the culture well by adding 1 ml TRIzol and pipetting 15 times to fully dissolve the Matrigel domes. Lysates were transferred to pre-labelled 1.5 ml tubes, snap-frozen and stored at −80 °C until RNA extraction. Total RNA was subsequently isolated using a standard TRIzol-based extraction protocol.

Purified RNA was then subjected to chemical derivatization according to the SLAM-seq Kinetics kit–Catabolic Kinetics Module manufacturer’s instructions. In brief, 4sU-labelled RNA was alkylated with iodoacetamide under the recommended reaction conditions to modify incorporated 4sU residues before library preparation. Alkylated RNA was purified and used as input for sequencing library construction. SLAM-seq chemistry enables reverse transcriptase to incorporate G opposite alkylated 4sU during cDNA synthesis, which results in diagnostic T > C conversions in sequencing reads derived from newly labelled transcripts. Sequencing libraries were generated from total RNA using a QuantSeq 3′ mRNA-seq V2 Library Prep kit FWD with Unique Dual Indices according to the manufacturer’s instructions. This protocol uses oligo(dT)-primed first-strand synthesis, followed by RNA removal, random-primed second-strand synthesis, bead purification and PCR amplification to generate strand-specific Illumina-compatible libraries enriched for the 3′ ends of polyadenylated transcripts. Unique dual indices were introduced during PCR amplification. Libraries were prepared from total RNA without previous poly(A) enrichment or rRNA depletion, consistent with the QuantSeq workflow.

#### SLAM-seq data processing and half-life analysis

Raw SLAM-seq reads were processed using the nf-core/slamseq pipeline (v.1.0.0) implemented in Nextflow. Sequencing reads were trimmed for adapters and low-quality bases using Trim Galore, and processed reads were aligned using Slamdunk, which performs conversion-aware mapping, alignment filtering, multimapper recovery, SNP calling to mask genomic T > C variants and quantification of both total and T > C-converted reads. Read counts were first generated at the UTR level and then collapsed to the gene level. The pipeline also generated quality-control metrics summarizing nucleotide-conversion frequencies across reads, read positions and gene–UTR positions, together with trimming and alignment statistics compiled in a MultiQC report. Human organoid datasets were processed against the GRCh38 reference genome. Generation of 3′ UTR annotations and Nextflow pipeline configurations followed the settings described in a previous study^83^. For RNA half-life estimation, gene-level T > C conversion rates were background-corrected using matched unlabelled samples, with control samples corrected using unlabelled control samples and knockdown samples corrected using unlabelled knockdown samples. In organoid samples, residual 4sU retention in Matrigel resulted in delayed peak labelling, with many transcripts reaching maximal T > C signal at 2 h after chase initiation. Therefore, for the 5,888 genes that peaked at *t* = 2 h in control samples, *t*_2_ was considered the first effective decay time point, and half-lives were calculated by fitting a single-exponential decay model to the *t*_2_, *t*_5_ and *t*_12_ time points. RNA half-lives were calculated as *t*_1/2_ = ln(2)/*k*, where *k* is the fitted first-order decay constant. For control versus knockdown comparisons, only these 5,888 genes were considered, and genes were further filtered for good decay-model fit in the control condition (*R*^2^ exp > 0.6, where *R*^2^ exp is the coefficient of determination of the single-exponential decay model fit for each gene).

### Live-cell confocal microscopy

Confocal imaging was performed on stably transduced, doxycycline-inducible eGFP, fsZFP36L2–eGFP, ZFP36L2–eGFP and meGFP organoids seeded in a 1:1 mixture of culture medium and Matrigel on Ibidi glass-bottom chamber slides (80821). Imaging was carried out using a Nikon CSU-W1 SoRa spinning disk confocal microscope equipped with a ×60 oil-immersion objective, 1.49 NA, 300 ms exposure time, 5% laser power and 1 μm *z*-stack intervals. Variable settings, treatments or imaging time windows specific to each experiment are detailed in the figure legends. For nuclear–cytoplasmic shuttling experiments, organoids expressing eGFP-tagged constructs were co-transduced with a H2B–mCherry nuclear marker. Live-cell imaging was performed using the same microscope setup at ×60 magnification, with 300 ms exposure time, 5% laser power and continuous acquisition of 0.2 μm *z* stacks.

### Condensate tracking analysis

Time-lapse videos were first motion-corrected for sample drift using the Correct 3D Drift plugin in Fiji, with drift correction restricted to the *x–y* plane and based on the condensate channel to avoid instability in *z* axis estimation. Background subtraction was then performed on the condensate channel using a rolling-ball radius of 50 μm to reduce variability arising from elevated background fluorescence in some cells. Condensates were subsequently detected and tracked in 3D using the TrackMate plugin in Fiji (TrackMate 7)^84^. Spot detection was performed with the difference-of-Gaussians detector using a sigma of 1.25 μm. The quality threshold was manually adjusted for each video on the first frame to optimize condensate detection while minimizing false-positive assignments. Tracking was performed using the Simple LAP tracker, with a maximum linking distance of 4 μm between consecutive frames and a maximum gap-closing distance of 2 μm. Tracklets spanning fewer than two frames were excluded from further analyses. The remaining trajectories were overlaid on maximum-intensity projections of the drift-corrected videos. For visualization, tracks were displayed retrospectively for up to five preceding frames across all *z* planes.

### FRAP

FRAP experiments were performed using a Stellaris confocal imaging system (Leica) equipped with a ×63 1.4 NA oil-immersion objective. Stably transduced, doxycycline-inducible eGFP, fsZFP36L2–eGFP and WT ZFP36L2–eGFP organoid lines were seeded on Lab-Tek chamber slides (155409) 3 days before imaging, with doxycycline (2 μg ml^−1^) added 24 h before the experiment to induce construct expression. A ROI measuring 1.5 × 1.5 μm was photobleached at 35% laser power for 15 frames (0.6 s per frame), followed by acquisition of 30 frames (1 s per frame) to monitor fluorescence recovery. Fluorescence intensity in the ROI was quantified using Fiji (ImageJ), and the mobile fraction was calculated as:

$$\mathrm{Mobile}\,\mathrm{fraction}=\frac{F(\infty )-F(0)}{F(\mathrm{pre})-F(0)}$$

where *F*(*∞*) is the fluorescence intensity at steady state after recovery, *F*(0) is the intensity immediately after bleaching and *F*(pre) is the pre-bleach intensity.

### Statistics and reproducibility

No statistical method was used to predetermine sample sizes. The number of samples (*n*) is indicated in each figure panel, and statistical analyses are described in the corresponding figure legends. In vitro experiments were repeated a minimum of three times independently with similar results unless otherwise noted. All staining experiments were performed with a minimum of three biological replicates. Mice that died within 24 h of surgery were excluded from analysis as procedure-related complications; no other data were excluded.

### Reporting summary

Further information on research design is available in the Nature Portfolio Reporting Summary linked to this article.

## Online content

Any methods, additional references, Nature Portfolio reporting summaries, source data, extended data, supplementary information, acknowledgements, peer review information; details of author contributions and competing interests; and statements of data and code availability are available at https://doi.org/10.1038/s41586-026-10890-0.

## Supplementary information

Supplementary Information Supplementary Data Figs. 1–7.

Reporting Summary

Supplementary Table 1 Supplementary Table containing: **a** Gene list of tumour ISC-like gene module 29; **b** Gene sets used as reference for GSEA in Fig. 1i and Extended Data Fig. 1g; **c** Autocorrelation scores of genes correlated with ZFP36L2 in normal colon epithelial cells; **d** Autocorrelation scores of genes correlated with ZFP36L2 in CRC tumour epithelial cells; **e** GSEA of genes based on the autocorrelation scores with ZFP36L2 in normal colon epithelial cells; **f** GSEA of genes based on the autocorrelation scores with ZFP36L2 in CRC tumour epithelial cells.

Supplementary Table 2 Supplementary Table containing gene sets used for ssGSEA and gene sets used for scoring cell states (Moorman et al. 2025).

Supplementary Table 3 Supplementary Table containing gene expression counts in ZFP36L2 perturbation conditions and *ZFP36L2* mutation information for CRC tumours from patients in Fig. 3m,n.

Supplementary Table 4 A list of plasmid sequences used in this study and a list of primers and PCR programs used for genotyping.

Supplementary Table 5 Supplementary Table containing: **a** Profiling of ZFP36L2 target mRNAs edited by HyperTRIBE; **b** Profiling of ZFP36L2 dependent RNA decay by ActD treatment; **c** GO enrichment analysis of ZFP36L2 dependent decay genes shared by KG146Li and MSK107Li; **d** GO enrichment analysis of shared and individual ZFP36L2-bound mRNAs identified in OKG146N, OKG146P and OKG146Li organoids; **e** Half-lives of transcripts profiled by SLAM-seq in MSK107Li organoids; **f** GO enrichment analysis of transcripts with prolonged RNA half-lives after ZFP36L2 depletion relative to control, identified by SLAM-seq in MSK107Li organoids.

Supplementary Videos 1–3 **Supplementary Video** **1** shows live imaging of day 3 nuclear H2B–mCherry tagged WT ZFP36L2–eGFP, fsZFP36L2–eGFP and eGFP organoids (**1**.**a**.**1**. MSK107Li_eGFP; **1**.**a**.**2**. MSK107Li_fsZFP36L2-eGFP; **1**.**a**.**3**. MSK107Li_ZFP36L2-eGFP; **1**.**b**.**1**. OKG146P_eGFP; **1**.**b**.**2**. OKG146P_fsZFP36L2-eGFP; **1**.**b**.**3**. OKG146P_ZFP36L2-eGFP; **1**.**c**.**1**. OKG146PLi_eGFP; **1**.**c**.**2**. OKG146Li_fsZFP36L2-eGFP; **1.c.3**. OKG146Li_ZFP36L2-eGFP). **Supplementary Video 2** shows live imaging tracking ZFP36L2–eGFP condensate expression after dissociation to single-cell stage (**2**.**a**. MSK107Li_ZFP36L2-eGFP_1; **2**.**b**. MSK107Li_ZFP36L2-eGFP_2; **2**.**c**. MSK107Li_ZFP36L2-eGFP_3; **2**.**d**. MSK107Li_ZFP36L2-eGFP_4; **2**.**e**. MSK107Li_ZFP36L2-eGFP_5). **Supplementary Video 3** shows FRAP in WT ZFP36L2–eGFP, fsZFP36L2–eGFP and eGFP organoids (**3**.**a**.**1**. MSK107Li_eGFP; **3**.**a**.**2**. MSK107Li_fsZFP36L2-eGFP; **3**.**a**.**3**. MSK107Li_ZFP36L2-eGFP; **3**.**b**.**1**. OKG146P_eGFP; **3**.**b**.**2**. OKG146P_fsZFP36L2-eGFP; **3**.**b**.**3**. OKG146P_ZFP36L2-eGFP; **3.c**.**1**. OKG146PLi_eGFP; **3**.**c**.**2**. OKG146Li_fsZFP36L2-eGFP; **3**.**c**.**3**. OKG146Li_ZFP36L2-eGFP).

Peer Review File

## Source data

Source Data Figs. 2 and 3 and Extended Data Fig. 5

## Data Availability

All raw and processed sequencing data generated in this study have been deposited into the NCBI GEO database under superseries accession number GSE336781, which encompasses the following subseries: GSE336319, GSE336415, GSE336416, GSE336417 and GSE336564. CRC scRNA-seq data from patients were obtained from the HTAN Data Portal (http://humantumouratlas.org/publications/hta8_crc_moorman_2024). Mouse colon epithelial cell scRNA-seq data were obtained from the NCBI GEO under accession number GSE168448 and are further cited in the Methods. Source data are provided with this paper.

## Code availability

No custom code was generated in this study. All bioinformatic analyses were carried out using publicly available software and packages as described in the Methods and cited accordingly.
