# Supplementary material for: ZFP36L2 orchestrates stress-adaptive plasticity in regeneration and cancer
Source: Nature. 2026 Aug 5;657(8133):1053–63. doi: 10.1038/s41586-026-10890-0 (PMC13600930; doi:10.1038/s41586-026-10890-0)
Supplement: Supplementary file 1 [file 41586_2026_10890_MOESM1_ESM.pdf]

nature portfolio

https://doi.org/10.1038/s41586-026-10890-0

Supplementary information

ZFP36L2 orchestrates stress-adaptive plasticity in regeneration and cancer

In the format provided by the
authors and unedited

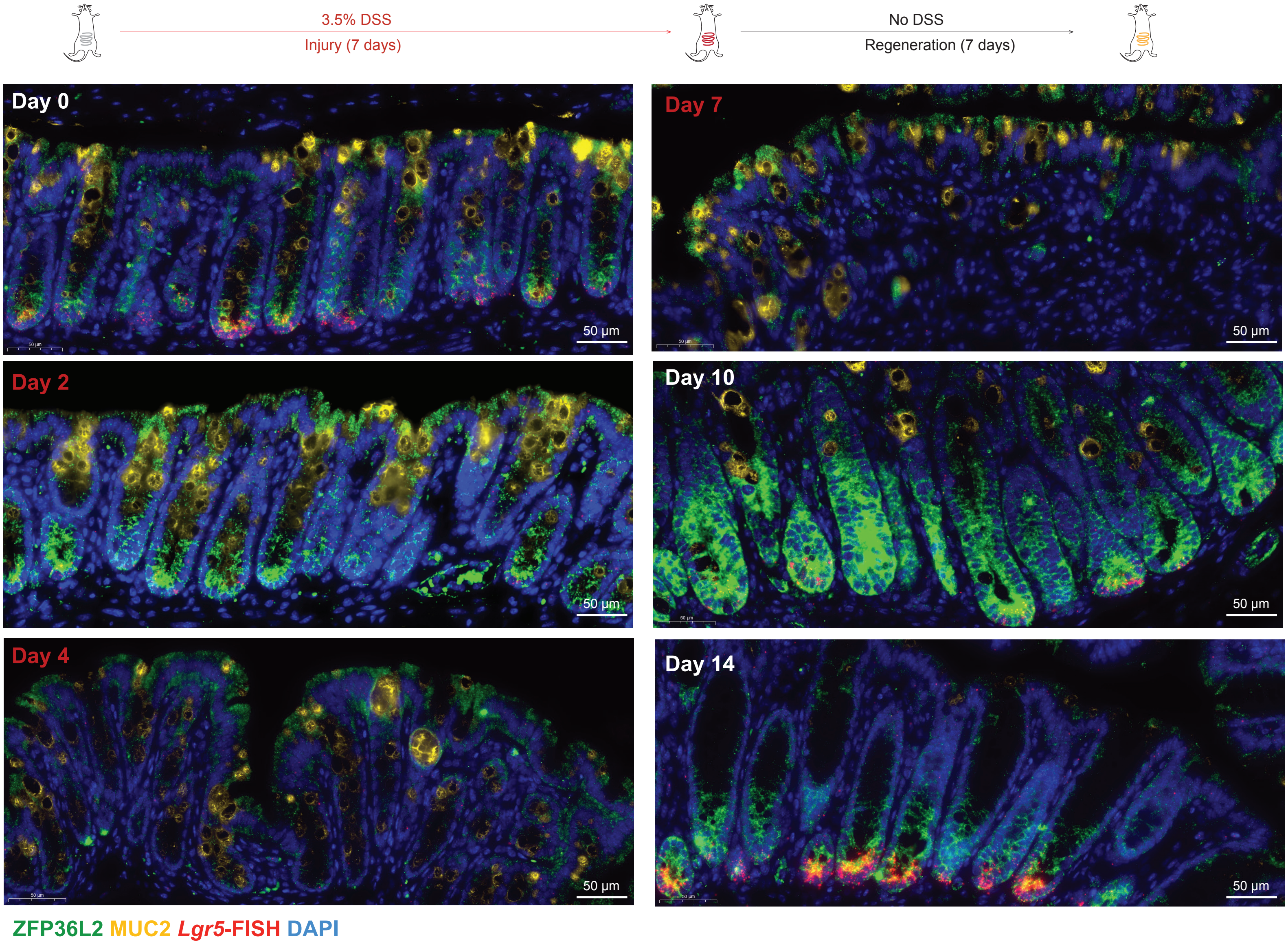

Supplementary data 1. Expanded field of view corresponding to Fig. 2c.

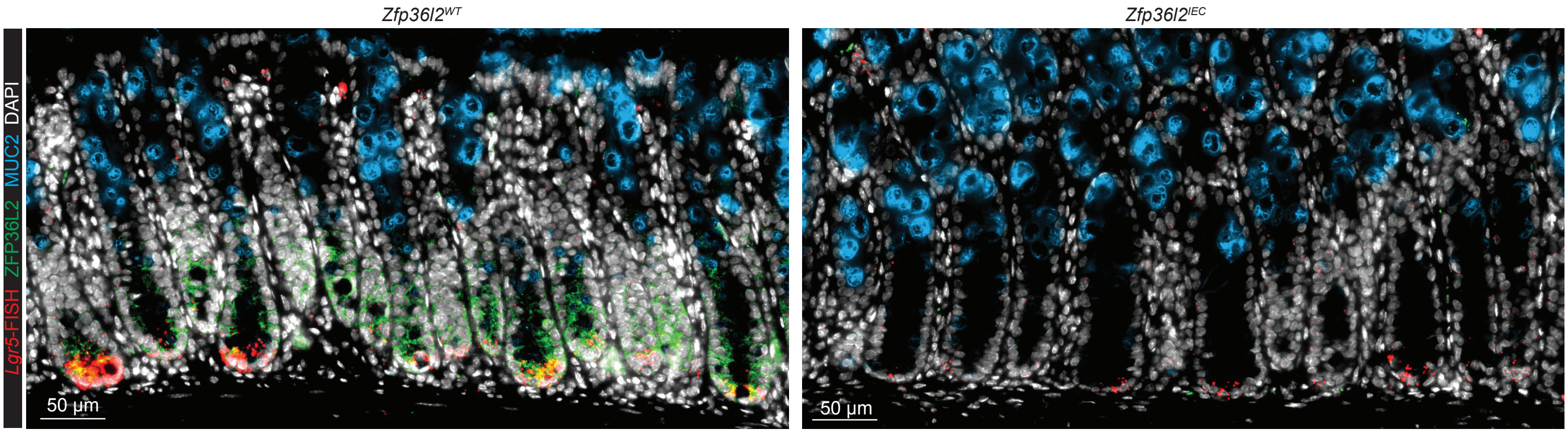

Supplementary data 2. Expanded field of view corresponding to Extended data fig3e.

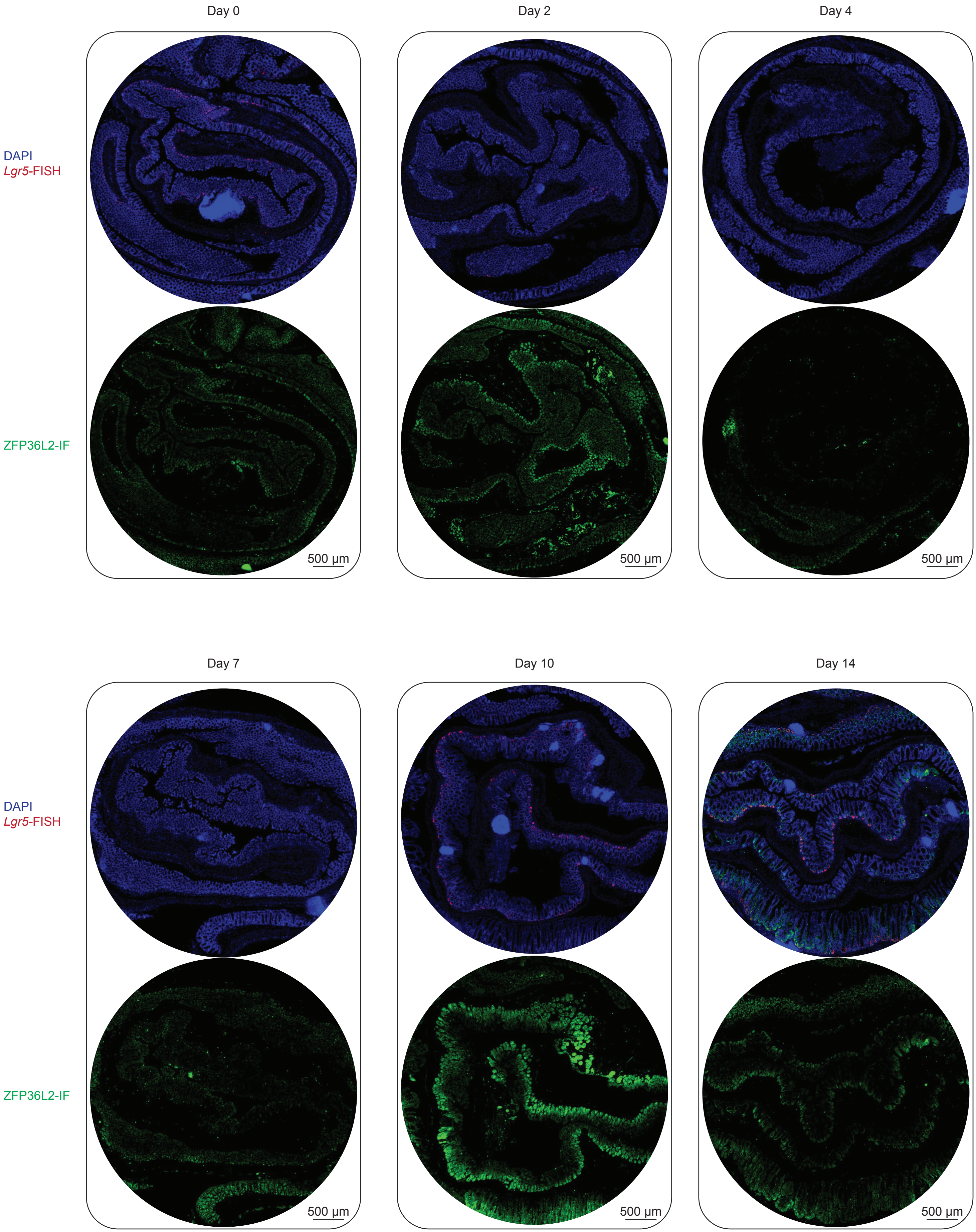

Supplementary data 3. Expanded field of view corresponding to Fig. 2c.

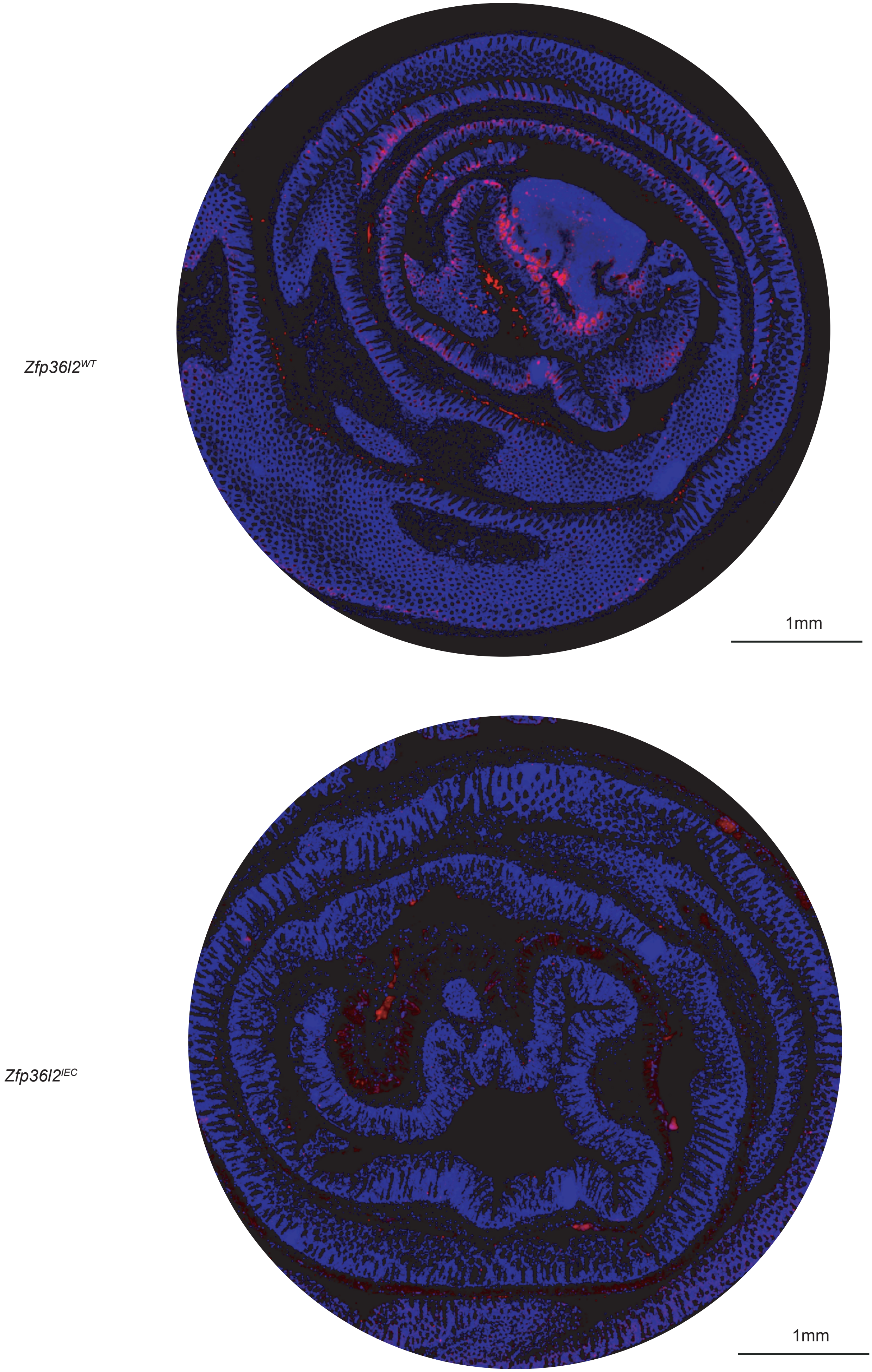

Supplementary data 4. Verification of efficient Zfp36l2 knockout in mouse colonic crypts.

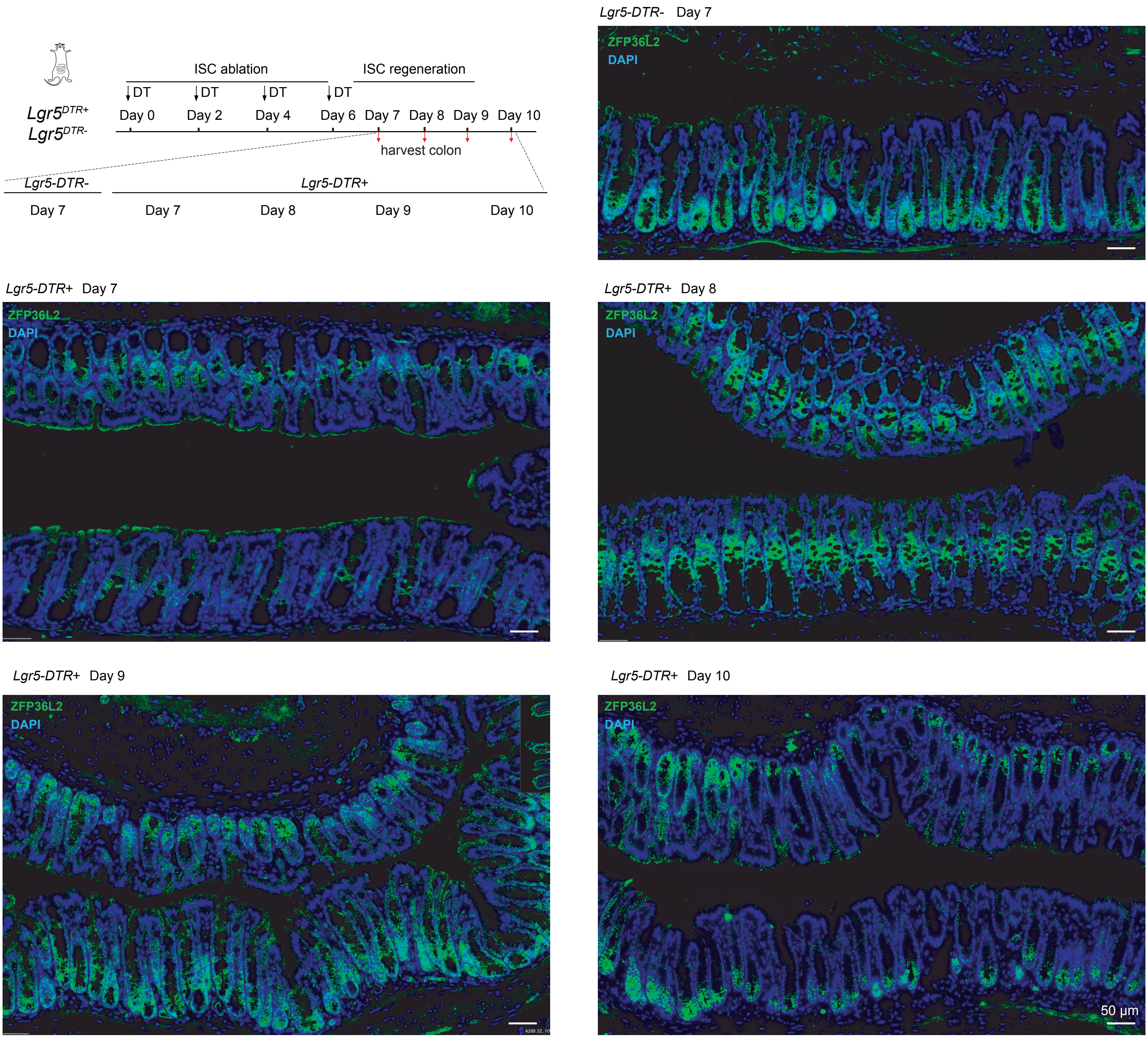

Supplementary data 5. Expanded field of view corresponding to Fig. 2f.

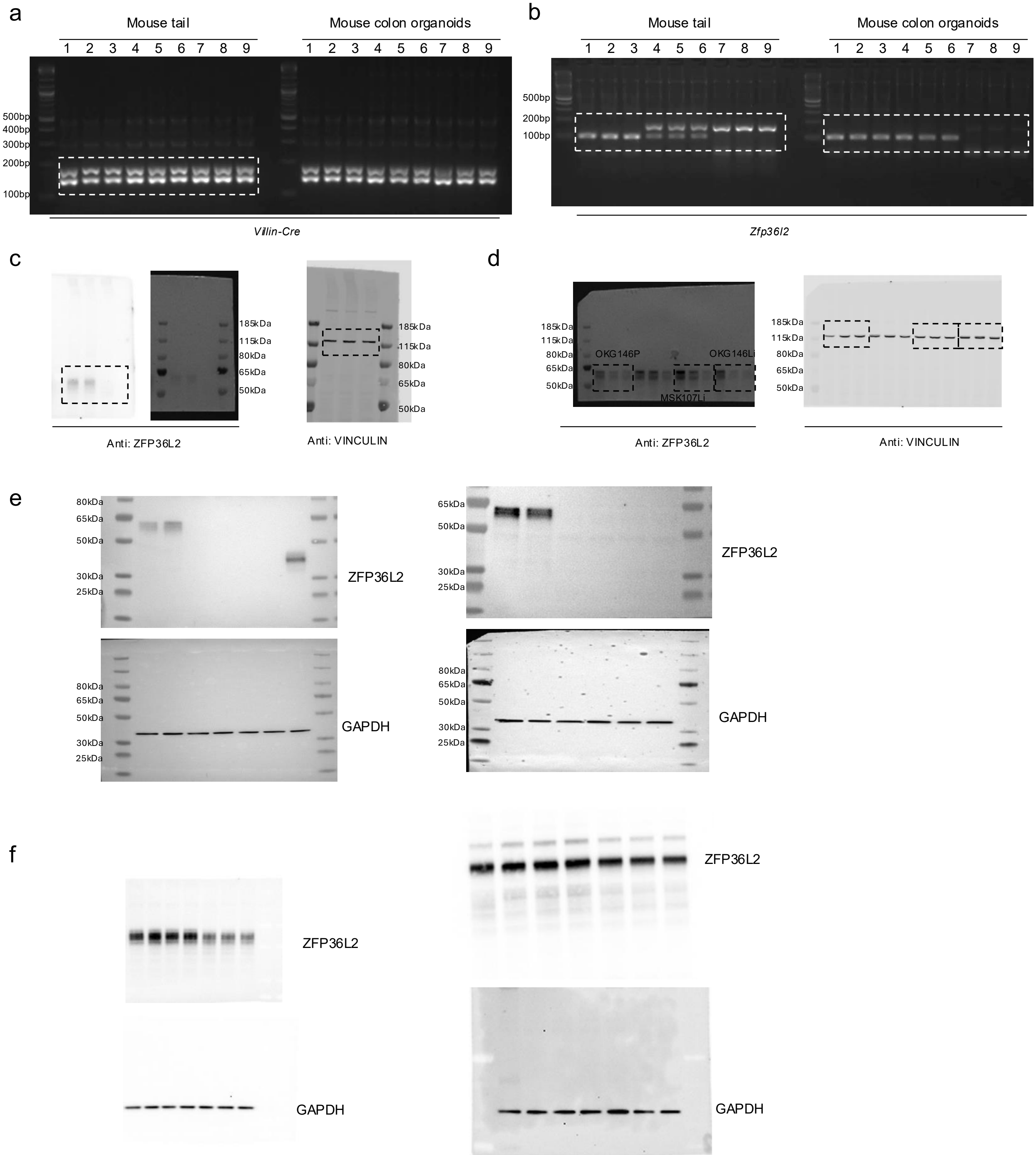

Supplementary data 6 | Source data for gel and immunoblot experiments.

a–b, Gel source data for Extended Data Fig. 3b. PCR control and target bands were loaded on the same gel. Dashed boxes indicate the regions cropped for the figure.

c, Immunoblot source data for Extended Data Fig. 3c. ZFP36L2 was detected using HRP substrate, and the blot was subsequently reprobed for VINCULIN using a fluorescence-conjugated secondary antibody. Dashed boxes indicate cropped regions; ladder overlay indicates band sizes.

d, Immunoblot source data for Extended Data Fig. 4d. ZFP36L2 was detected with HRP substrate, and the same blot was reprobed for VINCULIN using a fluorescence-conjugated secondary antibody. Dashed boxes indicate cropped regions; ladder overlay indicates band sizes.

e, Immunoblot source data for Extended data fig. 7a. ZFP36L2 and GAPDH were blotted on individual membranes. All blots were processed in parallel. Ladder overlays indicate band sizes.

f. Immunoblot source data for Extended data fig. 9i. ZFP36L2 and GAPDH were blotted on individual membranes. All blots were processed in parallel.

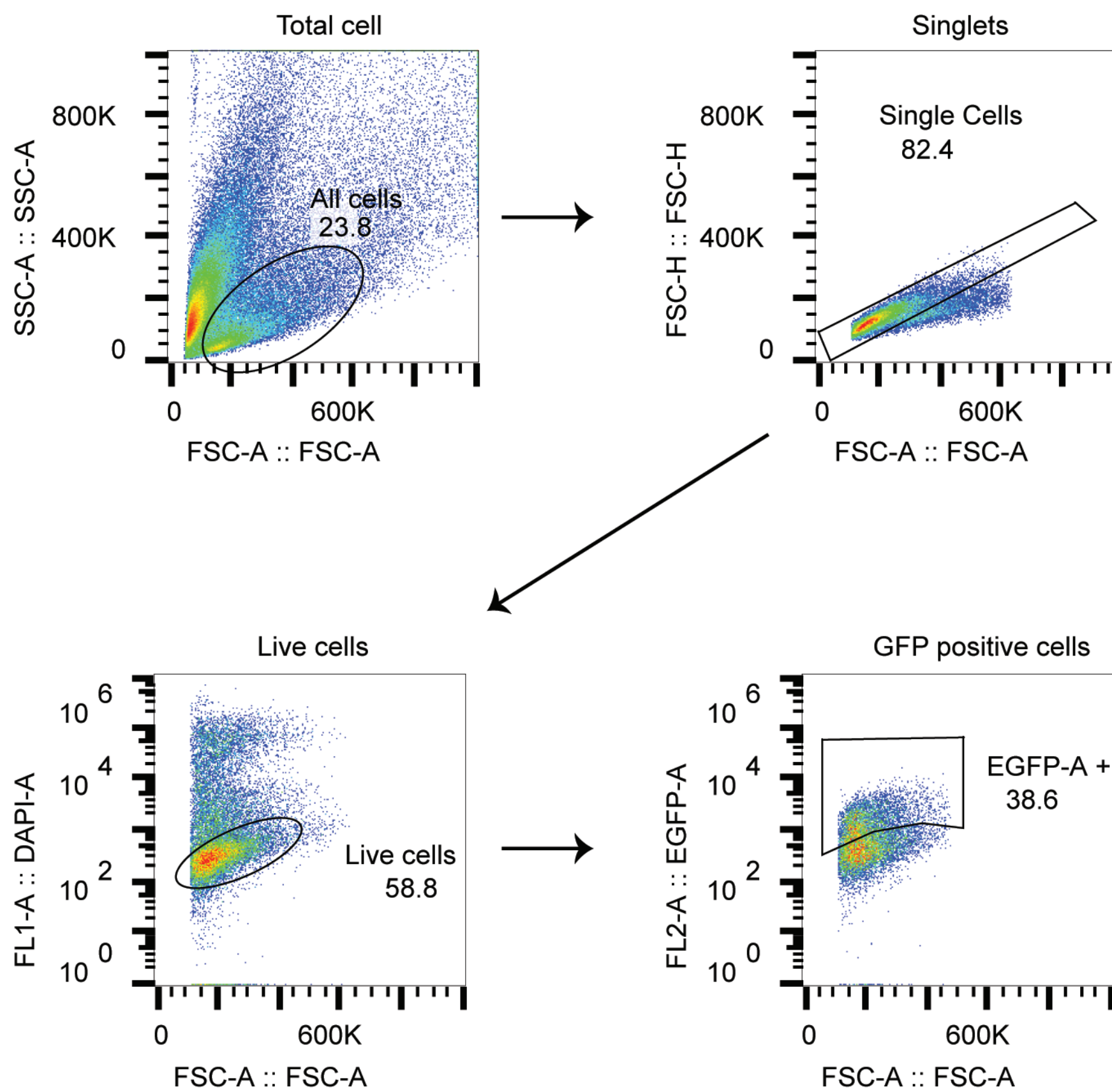

Supplementary data 7. Gating strategy for Extended Data Fig.3h and Extended Data Fig.4c,d,h.
