# Supplementary material for: ZFP36L2 orchestrates stress-adaptive plasticity in regeneration and cancer
Source: Nature. 2026 Aug 5;657(8133):1053–63. doi: 10.1038/s41586-026-10890-0 (PMC13600930; doi:10.1038/s41586-026-10890-0)
Supplement: Supplementary file 2 [file 41586_2026_10890_MOESM2_ESM.pdf]

nature portfolio

Corresponding author(s): Karuna Ganesh, MD, PhD

Last updated by author(s): Jun 28, 2026

Reporting Summary

Nature Portfolio wishes to improve the reproducibility of the work that we publish. This form provides structure for consistency and transparency in reporting. For further information on Nature Portfolio policies, see our Editorial Policies and the Editorial Policy Checklist.

Statistics

For all statistical analyses, confirm that the following items are present in the figure legend, table legend, main text, or Methods section.

n/a	Confirmed	
☐	☒	The exact sample size (n) for each experimental group/condition, given as a discrete number and unit of measurement
☐	☒	A statement on whether measurements were taken from distinct samples or whether the same sample was measured repeatedly
☐	☒	The statistical test(s) used AND whether they are one- or two-sided<br>*Only common tests should be described solely by name; describe more complex techniques in the Methods section.*
☐	☒	A description of all covariates tested
☐	☒	A description of any assumptions or corrections, such as tests of normality and adjustment for multiple comparisons
☐	☒	A full description of the statistical parameters including central tendency (e.g. means) or other basic estimates (e.g. regression coefficient) AND variation (e.g. standard deviation) or associated estimates of uncertainty (e.g. confidence intervals)
☐	☒	For null hypothesis testing, the test statistic (e.g. F, t, r) with confidence intervals, effect sizes, degrees of freedom and P value noted<br>*Give P values as exact values whenever suitable.*
☒	☐	For Bayesian analysis, information on the choice of priors and Markov chain Monte Carlo settings
☒	☐	For hierarchical and complex designs, identification of the appropriate level for tests and full reporting of outcomes
☒	☐	Estimates of effect sizes (e.g. Cohen's d, Pearson's r), indicating how they were calculated

Our web collection on statistics for biologists contains articles on many of the points above.

Software and code

Policy information about availability of computer code

Data collection	Data were collected using multiple platforms and instruments tailored to each experimental modality. Next-generation sequencing was performed on the Illumina NovaSeqX and NovaSeq 6000 platforms. Quantitative PCR data were acquired and analyzed using Design & Analysis Software v2.6.0. Western blot images were captured using the iBright CL1000 Imaging System. FRAP experiments were conducted on the Stellaris Confocal Imaging System (Leica). Brightfield images of organoid formation assays were acquired using Gen5 Image Prime v3.08. Bioluminescence imaging of tumour-bearing mice was performed using the IVIS Spectrum Xenogen System (Caliper Life Sciences). Flow cytometry and cell sorting were performed using the Sony Cell Sorter with Sony Cell Sorter Software v2.1.5. Fluorescence in situ hybridization (FISH) and immunofluorescence (IF) staining were performed on the Leica Bond RX automated stainer, and slides were scanned using a Pannoramic Scanner (3DHistech). Single-cell RNA sequencing was carried out using the Chromium Single Cell 3′ v3.1 platform (10x Genomics). Live-cell confocal microscopy was performed using a Nikon CSU-W1 SoRa Spinning Disk Confocal Microscope.
Data analysis	Data analysis was performed using a range of software tools specific to each experimental modality. Single-cell RNA-seq data were processed and analyzed using Scanpy (v1.9.1 or v1.9.8), Seurat (v3), Scran (v3.20), MAGIC (v3.0.0), GSEApy (v0.14.0), Hotspot (v0.9.1), scDblFinder (v1.16.0), CellBender (v1.0.0), DoubletDetection (v3.0), ForceAtlas2, scVI, Harmony, PhenoGraph, MELD, and UMAP. Visualization and plotting were carried out using Matplotlib (v3.6.0). Image quantification and analysis were performed using CaseViewer (v2.4.0.119028), ImageJ (v2.14.0/1.54f), and QuPath (v0.5.0). Condensate tracking in live-cell imaging data was performed using TrackMate (v7) within FIJI, with sample drift corrected using the Correct 3D Drift plugin. Flow cytometry data were analyzed using FlowJo (v10.9.0). Bulk RNA-seq data were processed using Cutadapt (v4.8), STAR (v2.7.11b), HTSeq (v2.0.5), and DESeq2 (v1.42.0); variant calling was performed using GATK HaplotypeCaller (v3.8.1.0). SLAM-seq data were processed using the nf-core/slamseq pipeline (v1.0.0) implemented in Nextflow, incorporating Trim Galore, Slamdunk, and MultiQC. Gene set enrichment analyses were conducted using GSEApy (v0.14.0) and ssGSEA. Whole-exome sequencing data were analyzed using the TEMPO pipeline in conjunction with OncoKB. Statistical and graph-based analyses

were performed in GraphPad Prism (v10.4.1).

For manuscripts utilizing custom algorithms or software that are central to the research but not yet described in published literature, software must be made available to editors and reviewers. We strongly encourage code deposition in a community repository (e.g. GitHub). See the Nature Portfolio guidelines for submitting code & software for further information.

Data

Policy information about availability of data

All manuscripts must include a data availability statement. This statement should provide the following information, where applicable:

- Accession codes, unique identifiers, or web links for publicly available datasets
- A description of any restrictions on data availability
- For clinical datasets or third party data, please ensure that the statement adheres to our policy

All raw and processed sequencing data generated in this study have been deposited in the NCBI Gene Expression Omnibus (GEO) under SuperSeries accession number GSE336781, which encompasses the following SubSeries: GSE336319, GSE336415, GSE336416, GSE336417, and GSE336564. No custom code was generated in this study. All bioinformatic analyses were carried out using publicly available software and packages as described in the Methods section and cited accordingly. All other data generated and/or analyzed during this study are included in this article and its supplementary files, including main figures, extended data figures, and supplementary information.

Research involving human participants, their data, or biological material

Policy information about studies with human participants or human data. See also policy information about sex, gender (identity/presentation), and sexual orientation and race, ethnicity and racism.

Reporting on sex and gender	Tumour tissues for organoid generation were randomly selected based on sample availability, without consideration of the patient's sex or gender.
Reporting on race, ethnicity, or other socially relevant groupings	Human organoids, patient sequencing data, and FFPE tissue samples were collected under institutional review board–approved protocols (MSK IRB #14-244 and #22-404) with prior written informed consent from all participants. The human organoid lines used in this study were previously described in Ganesh et al. (2020) and Moorman et al. (2025).
Population characteristics	This study incorporated previously reported scRNA-seq data from a cohort of 31 patients with MSS/pMMR metastatic colorectal cancer (16 male, 15 female). Time to each treatment event was calculated from the date of diagnosis to enable comparison across patients. At the time of surgery, 17/31 patients presented with multiple metastatic sites, with greater than 50% of tumour sites remaining following resection. Seventeen of 31 patients had early-onset colorectal cancer (age at diagnosis <50 years). Prior to surgery, 22/31 patients had received 5-fluorouracil-based chemotherapy. Clinical MSK-IMPACT targeted exon sequencing was performed on matched tumour/normal tissue from 27/31 patients, revealing mutations consistent with expected colorectal cancer driver alterations. Comprehensive clinical and genetic characteristics of all patients are described in Moorman et al. (2025). For the present study, 25 patients with paired primary tumour and liver metastasis samples were included in the scRNA-seq analyses.<br>For immunostaining analyses, archival FFPE tissue from patients harbouring somatic ZFP36L2 mutations was examined (3 tissue blocks from male, 9 tissue blocks from female; age at sample collection 33–75 years). All patients in this cohort had received 5-fluorouracil-based chemotherapy prior to surgery.
Recruitment	Patients undergoing synchronous colorectal resection and metastasectomy at Memorial Sloan Kettering Cancer Center (MSKCC) were identified through chart review. Individuals who had provided written informed consent under MSK IRB protocols #06-107, #12-245, #14-244, and #22-404 for biospecimen collection were included in the MSKCC biobank. FFPE tissue samples were obtained through approved requests to the MSKCC biobank.
Ethics oversight	The study protocols were approved by MSK IRB

Note that full information on the approval of the study protocol must also be provided in the manuscript.

Field-specific reporting

Please select the one below that is the best fit for your research. If you are not sure, read the appropriate sections before making your selection.

☒ Life sciences ☐ Behavioural & social sciences ☐ Ecological, evolutionary & environmental sciences

For a reference copy of the document with all sections, see nature.com/documents/nr-reporting-summary-flat.pdf

Life sciences study design

All studies must disclose on these points even when the disclosure is negative.

Sample size	No statistical methods were used to predetermine sample sizes. Sample sizes were chosen based on prior experience in our laboratory with similar experimental systems and reported variability in the literature, and are consistent with those reported in previous studies of similar

design (Ganesh et al., Nature Cancer, 2020; Moorman et al., Nature, 2025). For in vivo mouse experiments, group sizes of n=5–15 per condition were determined based on prior experiments using similar xenograft and colitis models, with animal numbers kept to the minimum necessary in accordance with the 3Rs principles. For bulk RNA-seq, four biological replicates were included. In vitro experiments were performed with a minimum of three biological replicates per condition. For patient tissue immunostaining, samples were selected based on availability of ZFP36L2 mutation status from MSK-IMPACT sequencing and adequate tissue quality for FFPE staining. Detailed sample sizes for each experiment are provided in the corresponding figure legends.

Data exclusions

Animals that died within 24 hours following surgery were presumed to have succumbed to procedure-related complications and were therefore excluded from further analysis.

Replication

All attempts at replication were successful. Multiple biologically independent experiments were performed whenever feasible, as detailed in the Methods and figure legends. For in vitro assay representative images, each experiment was successfully repeated at least three times under similar conditions. In vitro experiments included at least two distinct patient-derived organoid (PDO) lines. All staining experiments were conducted on a minimum of two independent biological samples per group to ensure reproducibility, and representative images were selected from experiments in which consistent results were obtained across replicates and coupled with quantification of all samples.

Randomization

For in vivo experiments involving treatments, animals were assigned to treatment groups such that each group included mice from the same litters to minimize genetic and environmental variability. For in vitro experiments, randomization was not required, as all samples were processed and analyzed equally.

Blinding

Blinding was implemented throughout the study where applicable. Mouse surgeries and subsequent imaging were performed by different investigators, each blinded to the groups by number coding. Tissue collection and staining were also carried out by separate individuals, blinded to group allocation. Imaging and quantification of stained samples were performed using software with identical settings across conditions to ensure unbiased analysis. In other experiments, investigators were blinded through the use of coded sample identifiers. For experiments where all samples were processed uniformly and objectively (e.g., bulk sequencing or flow cytometry), blinding was not deemed necessary.

Reporting for specific materials, systems and methods

We require information from authors about some types of materials, experimental systems and methods used in many studies. Here, indicate whether each material, system or method listed is relevant to your study. If you are not sure if a list item applies to your research, read the appropriate section before selecting a response.

Materials & experimental systems

n/a	Involved in the study	
☐	☒	Antibodies
☐	☒	Eukaryotic cell lines
☒	☐	Palaeontology and archaeology
☐	☒	Animals and other organisms
☒	☐	Clinical data
☒	☐	Dual use research of concern
☒	☐	Plants

Methods

n/a	Involved in the study	
☒	☐	ChIP-seq
☐	☒	Flow cytometry
☒	☐	MRI-based neuroimaging

Antibodies

Antibodies used

Antibodies used for IF:
anti-ZFP36L2 (1:200; Santa Cruz, clone A-3, #sc-365908)
MUC2 (rabbit, 0.0625µg/mL, Abcam, #ab973f86)
pan-CK (mouse, 0.125 µg/mL, Abcam, #ab8068)
KI67 (rabbit, 0.25µg/mL, Abcam, #ab15580)
CK20 (rabbit, 1µg/mL, Novus Biologicals, #NBP3-03605)
CK5 (rabbit, 0.016µg/mL, Cell Signaling Technology, #81817)
Chromogranin B (rabbit, 0.1µg/mL, Invitrogen, #PA5-52605)
DDX6 (rabbit, 0.517 mg/mL, Abcam, #ab307418)
G3BP1 (mouse, 1mg/mL, Abcam, #ab56574)
PABPC1 (rabbit, 1mg/mL, Abcam,#ab21060)

Primary antibodies used for Western Blot:
mouse anti-β-actin (1:2,000; Abcam, #ab8226)
anti-GAPDH (1:2,000; Abcam, #ab181602)
anti-ZFP36L2 (1:200; Santa Cruz, clone A-3, #sc-365908)

Secondary antibodies for Western Blot:
HRP-conjugated anti-mouse or anti-rabbit secondary antibodies (1:10,000; Cell Signaling Technology, #7076 and #7074)

Secondary antibodies for IF staining:
Leica Bond Post-Primary (rabbit anti-mouse linker) (Leica, DS9800) for staining human samples with mouse-derived antibodies.
Rabbit anti-mouse secondary antibody linker (Abcam, ab133469) for staining mouse samples with mouse-derived antibodies.
Leica Bond Polymer anti-rabbit HRP (Leica, DS9800)

Validation	Pre-validated antibodies were obtained from reputable commercial sources. All primary antibodies were tested in the relevant applications to ensure specific detection of target proteins without cross-reactivity to non-specific proteins. Antibodies used for immunofluorescence (IF), and western blotting (WB) were extensively validated by the suppliers (see catalog numbers and company information listed above). Additional in-house validation was performed by comparing expression patterns in internal positive and negative controls for each assay. For IF and WB of ZFP36L2, specificity was confirmed using knockdown samples, as shown in the figures. For other antibodies used in IF, negative controls included unstained samples and samples processed without primary antibody but with secondary antibody. For WB, specificity of other antibodies was confirmed by detecting bands at the expected molecular weight using protein ladders.

Eukaryotic cell lines

Policy information about cell lines and Sex and Gender in Research

Cell line source(s)	Organoid lines used in this study were previously established and characterized in our earlier publications (Ganesh et al., 2020; Moorman et al., 2025). HEK293T cells were obtained from the American Type Culture Collection (ATCC).
Authentication	Human organoid lines used in this study were short tandem repeat (STR) verified at the time of establishment and prior to each experiment. Mouse organoids were genotyped before each experiment to confirm genotype. HEK293T cells were obtained from the American Type Culture Collection (ATCC) and were not subjected to further authentication.
Mycoplasma contamination	All cell and organoid lines used in this study were routinely tested for mycoplasma contamination using the MycoAlert PLUS Detection Kit (Lonza), and all tested negative.
Commonly misidentified lines (See ICLAC register)	None

Animals and other research organisms

Policy information about studies involving animals; ARRIVE guidelines recommended for reporting animal research, and Sex and Gender in Research

Laboratory animals	The following mouse strains were used for colon regeneration experiments at 6–8 weeks of age:<br>C57BL/6J (strain 000664), Vil1-Cre (B6.Cg-Tg(Vil1-cre)1000Gum/J; stock 021504), Lgr5-EGFP-IRES-creERT2 (B6.129P2-Lgr5tm1(cre/ERT2)Cle/J; stock 008875), Zfp36l2fl/fl and Lgr5-DTR-eGFP mice.<br>For xenograft tumor evaluation experiments, NSG (NOD.Cg-PrkdcscidIl2rgtm1Wjl/SzJ; stock 005557)were used at 6 weeks of age. All mice were maintained at the MSKCC Research Animal Resource Center (RARC) under a 12-hour light/dark cycle at controlled temperature and humidity, with ad libitum access to standard diet and water. NSG mice were housed under specific pathogen-free (SPF) conditions. C57BL/6J (strain 000664) and other immunocompetent strains, including Vil1-Cre (stock 004586), Lgr5-EGFP-IRES-creERT2 (stock 008875), Zfp36l2fl/fl, and Lgr5-DTR-eGFP mice, were housed under standard barrier conditions.
Wild animals	This study did not involve wild animals.
Reporting on sex	Xenograft experiments were performed using female NSG mice. For colon regeneration experiments, both male and female mice were used, with equal representation of sexes, and animals were selected from the same litters to minimize variability.
Field-collected samples	No field collected samples were used in this study.
Ethics oversight	All animal experiments were conducted in accordance with protocols approved by the Institutional Animal Care and Use Committee (IACUC) of Memorial Sloan Kettering Cancer Center.

Note that full information on the approval of the study protocol must also be provided in the manuscript.

Plants

Seed stocks	*Report on the source of all seed stocks or other plant material used. If applicable, state the seed stock centre and catalogue number. If plant specimens were collected from the field, describe the collection location, date and sampling procedures.*
Novel plant genotypes	*Describe the methods by which all novel plant genotypes were produced. This includes those generated by transgenic approaches, gene editing, chemical/radiation-based mutagenesis and hybridization. For transgenic lines, describe the transformation method, the number of independent lines analyzed and the generation upon which experiments were performed. For gene-edited lines, describe the editor used, the endogenous sequence targeted for editing, the targeting guide RNA sequence (if applicable) and how the editor was applied.*
Authentication	*Describe any authentication procedures for each seed stock used or novel genotype generated. Describe any experiments used to assess the effect of a mutation and, where applicable, how potential secondary effects (e.g. second site T-DNA insertions, mosiacism, off-target gene editing) were examined.*

Flow Cytometry

Plots

Confirm that:

☒ The axis labels state the marker and fluorochrome used (e.g. CD4-FITC).

☒ The axis scales are clearly visible. Include numbers along axes only for bottom left plot of group (a 'group' is an analysis of identical markers).

☒ All plots are contour plots with outliers or pseudocolor plots.

☒ A numerical value for number of cells or percentage (with statistics) is provided.

Methodology

Sample preparation	For quantification of LGR5-GFP by crypt flow cytometry in mice colon, Colons were dissected, flushed with PBS to remove faecal content, opened longitudinally, and cut into approximately 1 cm segments. Tissue pieces were incubated in dissociation buffer (PBS supplemented with 8 mM EDTA, 0.5 mM DTT, and 10 U/mL DNase I (Roche, #04716728001)) at 4°C with gentle shaking for 60 minutes. Crypts were detached by vigorous shaking, pelleted by centrifugation, and further dissociated into single cells using TrypLE (Thermo Fisher Scientific). The resulting suspension was treated with DNase I at room temperature for 5 minutes, then passed through a 40 µm cell strainer to generate single-cell suspensions. Viable epithelial cells were identified by forward and side scatter gating and exclusion of DAPI-positive (4′,6-diamidino-2-phenylindole) dead cells. Lgr5-EGFP-IRES-creERT2−, Vil1-Cre+, Zfp36l2^WT^ mice were processed in parallel to serve as gating controls for GFP-negative populations. Two independent experimental batches were performed, and data were pooled for analysis.<br>For assessment of LGR5-GFP expression in organoids, organoids were recovered from Matrigel by incubation in 2 mM EDTA in DPBS followed by orbital agitation at 4°C. Organoids were then dissociated into single cells using TrypLE and passed through a 40 µm cell strainer. Viable single cells were identified by forward and side scatter gating and exclusion of DAPI-positive dead cells prior to flow cytometric quantification of GFP fluorescence.
Instrument	SH800S Sony Cell Sorter
Software	Soni Cell Sorter Software v2.1.5 were used for data collection and FlowJo were used for data analysis.
Cell population abundance	GFP-positive cells comprised approximately 3–5% of viable, single, DAPI-negative cells isolated from colonic crypts of Lgr5-EGFP-IRES-creERT2 mice. In the Lgr5-DTR-GFP organoid model, GFP-positive cells represented 20–40% of viable single cells.
Gating strategy	Quantification of LGR5-GFP by crypt flow cytometry:<br>Cells were first gated on forward scatter area (FSC-A) and side scatter area (SSC-A) to exclude debris. Doublets were excluded using sequential gating on FSC-H versus FSC-A and FSC-W versus FSC-A. Live cells were identified by gating DAPI-negative events using FSC-A versus DAPI. Unstained controls (no fluorophores and no DAPI) and GFP-positive/DAPI-negative controls were used to define and validate the gating thresholds for identifying GFP-positive populations.

☒ Tick this box to confirm that a figure exemplifying the gating strategy is provided in the Supplementary Information.
