# Supplementary material for: ZFP36L2 orchestrates stress-adaptive plasticity in regeneration and cancer
Source: Nature. 2026 Aug 5;657(8133):1053–63. doi: 10.1038/s41586-026-10890-0 (PMC13600930; doi:10.1038/s41586-026-10890-0)
Supplement: Supplementary file 3 [file 41586_2026_10890_MOESM9_ESM.pdf]

nature portfolio

Peer Review File

ZFP36L2 orchestrates stress-adaptive plasticity in regeneration and cancer

Corresponding Author: Dr Karuna Ganesh

Any redactions in this file are there to maintain patient confidentiality, the confidentiality of unpublished data, or to remove third-party material.

This file contains all reviewer reports in order by version, followed by all author rebuttals in order by version.

Version 0:

Reviewer comments:

Referee #1

(Remarks to the Author)
This study by Jiang et al investigates the mechanism underlying the de-differentiation of cells known to occur during intestinal regeneration injury that is also recapitulated during colorectal cancer metastasis. Although it has been clearly demonstrated in numerous studies that when Lgr5 positive cells are lost, differentiated cell types undergo a reprogramming process that leads to the replacement of Lgr5-positive cells, the trigger for this de-differentiation has not been identified. This study fills this gap by identifying a key molecule ZFP36L2 driving this process. Furthermore, the authors provide a link to CRC metastasis by showing ZFP36L2 knockdown significantly reduced metastatic burden and inhibits de-differentiation. Finally, the authors examine the mechanism of ZFP36L2 action and demonstrate the ability of ZFP36L2 to form RNA-dependent condensates and pinpoint a specific role in promoting mRNA decay of stress-associated mRNAs including FOSL2, JUND, ATF4. [redacted] his revealed a dual function for ZFP36L2 in terminating the stress response and promoting de-differentiation. Overall, this is an outstanding study both in the breadth in defining function spanning regeneration and tumour metastasis and the robust methodology that defines the underlying mechanisms.

The key findings from this study are that:
1. ZFP36L2 is highly associated with stemness in both the normal colon and CRC, is associated with the regenerative RevCSC signature and mutated in around 5-10% of CRCs.
2. ZFP36L2 is up-regulated during regeneration following injury in the DSS mouse model and knockout revealed a key functional role in regeneration.
3. Knockdown of ZFP36L2 in human CRC organoid models reduces metastatic burden and blocks the de-differentiation state.
3. ZFP36L2 binds and promotes the decay of mRNAs associated with stress response and therefore has a key role in terminating the stress response.
[redacted]

This is likely to be of considerable interest to researchers studying regeneration of the colonic epithelium following injury and mechanisms of cancer cell plasticity in colorectal cancer. The specific function of ZFP362 has not been defined previously. The results of this study will also have interest more broadly in other regenerative models and solid cancers where similar mechanisms may be observed.

Data and Methodology: The outstanding strengths of this study are the breadth of models utilising both mouse and human models of regeneration and tumour metastasis. The models encompass meticulous analysis of the [redacted] temporal patterns of expression of ZFP36L2 with other markers alongside functional studies in mouse and human models. For some of the conclusions suggested by the authors, lineage tracing should have been included. [redacted]

Use of statistics: The experiments are accompanied by detailed explanations in the figure legends of sample size and

statistical analyses. These were mostly robust with some exceptions outlined in the specific comments.

Discussion and Conclusions: The discussion is an accurate summary of the body of work with reference to the literature. The manuscript is very well written.

Specific Comments and suggested improvements

Line 69. Add Fig 1a

Figure 1B It is not clear which cell population was utilised to calculate mean expression. Could this be outlined or highlighted within the figure?

Line 85 The mutational profile of ZFP36L1 is provided but does this associate with clinical outcome or molecular sub-types of CRC?

Line 108 The conclusions from this section would be more convincing if lineage tracing from ZFP36L2 cells was conducted.

Figure 2b There is a significant number of cells expressing ZFP36L1 during homeostasis. Can the authors comment on a potential role here?

Figure 2cInjury-induced expression appears to start in the differentiated cells at the top of the crypts (day 2 to 7 Fig2c and then appear in the TA at day 10. Can the authors comment on this?

Line 29 Should be IF and RNA FISH

Line 133 Figure 2e Is there any change in the Goblet cell population?

Line 149-150 Regarding assessment of de-differentiation capacity, did the authors verify that the differentiated organoids lacked LGR5+ cells?

Line 184 “O” should be added to KG
Line 191 Bioluminescence Imaging (BLI)

Figure 2e The data would be more convincing if quantitative data on the cell types present could be provided.

Figure 2f Error bars should be included on the graph
Figure 2g, j, k Error bars are not clearly visible even though they are defined in the legend
Figure 3C, H Error bars are not visible, 3C include the organoid identity
Figure 3K The schematic diagram is not easy to interpret and could be made clearer.
Line 192 Further explanation of 3i should be included.
Figure 4F Error bars should be visible

Referee #2

(Remarks to the Author)
Many observations over the past decade or so advance the idea that colorectal cancer (CRC) cells are “plastic” in nature, mimicking physiologic cycles of differentiation and dedifferentiation in normal intestinal stem cells (ISCs). Among associated molecular processes, activation of a YAP/TAZ signal-related transcriptional program is seen both during tissue regeneration after ISC injury and in intestinal tumors. Several groups have recognized and studied this “oncofetal” program during intestinal tumor initiation, progression, and metastasis. However, its function and significance remain unclear and the literature on the topic is often imprecise. The present study “sought to identify the molecular switch controlling stress-induced dedifferentiation into an LGR5+ ISC state, a conserved process that is essential for both epithelial regeneration and the seeding and outgrowth of canonical metastases” (lines 73-75). Here, “canonical” distinguishes colonocytic from squamous or neuroendocrine “non-canonical” differentiation, as defined in the group’s recent study of biospecimen trios from normal colon and primary and metastatic CRCs.

The authors identify ZFP36L2 as a gene enriched in “tumor ISC-like cells”, mutated in 5-10% of CRCs, and ectopically expressed and required during mouse crypt epithelial regeneration (Figs. 1 and 2). Loss of ZFP36L2 in organoids derived from metastatic CRCs impairs their ability to seed liver deposits from distant graft sites but does not affect growth of those metastases, and RNA expression in ZFP36L2-deficient CRC organoids is interpreted to reflect excessive “non-canonical” differentiation (Fig. 3). immunofluorescence shows that ZFP36L2, which has multiple intrinsically disordered regions (IDRs), forms phase-separated RNA-dependent cytoplasmic condensates, more so in single isolated cells than in organoids (Fig. 4). Like other such factors, ZFP36L2 is associated with mRNA decay [redacted]

The manuscript is clearly written and thoughtfully assembled. The problem is that it tackles the entire gamut of possible ZFP36L2 activity in cells without solidifying any of at least half a dozen disparate conclusions. Many of these represent promising leads that would elicit enthusiasm if they were individually well proven and together generated a coherent model. Instead, the reader is left with a large number of dots that don't connect into a plausible arc and don't fulfill the goal to identify the molecular switch controlling stress-induced dedifferentiation. The large volume of data doesn't support the conclusion that ZFP36L2 is that elusive molecular switch.

In essence, this manuscript has two separate components that don't join in a conclusive or satisfying way. The first piece (Figs. 1-3) needs stronger evidence to implicate ZFP36L2 in epithelial regeneration or "stress-induced cancer cell dedifferentiation." The second piece (Figs. 4-5) needs stronger experimental support for ZFP36L2's dual cytoplasmic and nuclear functions and deeper insights into what the disparate findings might mean. Finally, to support the authors' claims (which are somewhat murky), the two components would need to be connected with greater rigor and plausibility.

Major comments (corresponding roughly to numbered Figures)

1. ZFP36L2 expression, "tumor ISC-like" cells, and link to AP-1 factors. ZFP36L2 seems to be expressed in many crypt epithelial (except the most mature) and almost all tumor cells. It is not ISC-restricted (Figs. 1b, 1e, Extended Data Fig. 1b-c, Fig. 2a-b) and expression in "tumor ISC-like" cells is lower than in "normal" ISC or TA cells (Fig. 1b), though Extended Data Figs. 1a and 1d (difficult to understand) mask this point. How are tumor "ISC-like cells" defined? A "tumor ISC-like" signature is about equally auto-correlated with ZFP36L2 in normal (with a better FDR) than in CRC epithelium (Fig. 1i, Extended Data Fig. 1g). ZFP36L2 expression might seem even less selective if "tumor ISC-like" cells were defined differently. Tumor cells in the top and bottom deciles of ZFP36L2 expression are almost equal in their "CSC signatures" (Extended Data Fig. 2). What does it mean, then, to note that "ZFP36L2 is the second most mutated tumor ISC-associated gene"?

If ZFP36L2 is relatively enriched in stem and TA cells, then its association with an "AP-1 gene score" (Fig. 1j) is expected and may be trivial. The "revCSC signature" is amplified in nearly all ZFP36L2-high cells, but the "injury repair signature", L1CAM, and EMP1 are elevated in small fractions. Are the latter 3 elements increased in overlapping or distinct cells, and what is the significance? Most important, how do the authors reconcile widely elevated AP-1 gene scores (Fig. 1j) with the small fraction of "revCSC" and "injury repair" expressing cells seen only within the ZFP36L2-high decile.

In summary, Fig. 1 and Extended Data Figs. 1 and 2 seem intended to defend interest in ZFP36L2 and build a connection to AP-1 complexes, but the data are murky and don't support the implied conclusions. Broad expression in primary tumors and apparent diminution in metastases doesn't shed useful light (Fig. 1e – how many samples were examined? how representative are the areas shown?) and statements like "Although ZFP36L2 was most broadly expressed in tumour ISC-like cells, …." (line 96) add to the confusion. Mutation in 5-10% of CRCs is arguably sufficient justification to study ZFP36L2 functions. Instead of laying the foundation for a potentially interesting study, the convoluted implications of Fig. 1 and Extended Data Figs. 1 and 2 muddy the waters. The leap from ZFP36L2 to AP-1 is tenuous.

2. Technical problems with experiments on ZFP36L2 requirement in intestinal regeneration.

(a) DSS colitis occurs in patches. To establish a "spatiotemporal sequence" of ZFP36L2 expression in this setting (Fig. 2c), the authors should demonstrate that the inferred changes occur in affected areas (the first 2 images are not so different from the control in Fig. 2b). Ideally, differences between affected and unaffected regions should be assessed/quantified in tissue "Swiss rolls". If ZFP36L2's role is in regeneration, can the authors explain why weight loss is more severe during the injury phase? If mutant epithelium is more sensitive to DSS damage, might recovery be delayed simply as a proportional effect? What happened to mutant body weight after 14 days? Did 10-15 mutant mice lose exactly the same fraction of body weight between 5 and 8 days (these are the only data points lacking a range)?

(b) Villin-Cre expression is patchy in the colon (Ref. 56), especially the distal colon, where DSS damage tends to be highest. To confirm gene loss, the authors should genotype the recombined allele in colonic epithelium (as shown for unrecombined tail DNA, Extended Data Fig. 3b) and show IF images for ZFP36L2 in tissue "Swiss rolls" from IEC-KO and control colons.

(c) Lgr5+ ISCs are selectively ablated in Lgr5-DTR mice (this can be confirmed using the GFP marker that is included in the Lgr5-DTR allele). Can the authors explain why all ZFP36L2+ cells are missing on Day 7 (Extended Data Fig. 3a)? Images from Days 8-10 seem to fall in the range of variation expected from different control images in the study and the large void spaces should be explained. To support the conclusions, larger areas of tissue should be shown, with accurate quantification of cell positions.

(d) The reduction of Lgr5+ ISCs in ZFP36L2-KO mice is intriguing and should be fleshed out. Can the authors explain and demonstrate it more persuasively? Fig. 2e displays only a few crypts and the experiment with Lgr5-Gfp quantitation (Extended Data Fig. 3e-g) is fraught because the Lgr5-Gfp-Cre allele has mosaic expression and few colonic crypts are labeled. Flow cytometry cannot distinguish between fewer labeled crypts (this number varies between mice) and fewer Lgr5+ cells per crypt. As indicated above, the authors should show larger areas (or "Swiss rolls") so that the range of intrinsic crypt-to-crypt variation can be assessed. Findings in mutant intestines should be quantified and interpreted in that context. Are stem cell numbers also reduced in the small intestine (where Lgr5 marker expression is more robust and can be supplemented with in situ hybridization for Olfm4).

(e) The dedifferentiation assay in organoids is not widely accepted as standard and there are many explanations for the findings (Fig. 2h-k), such as inherent epithelial defects unrelated to dedifferentiation. Increased sensitivity to immune

damage could underlie DSS pathology but is an improbable explanation for findings in Lgr5-DTR mice. If the different in vivo studies are done carefully (as outlined above), perhaps adding irradiation as another trigger for dedifferentiation, the organoid assay adds little to the study. The concern about organoid dedifferentiation extends to its application to support ideas on CRC dedifferentiation (Fig. 3l-n).

(f) The authors did use two different dox-inducible shRNAs in the study, which is nice but does not strictly exclude off-target effects. Rescue of at least one shRNA in some assay(s) would increase confidence in the authors' conclusions.

(g) Please check the graphs in Figs. 3f and 3i. Captions indicate that they refer to the same data, but the numbers at week 13 don't match (<1000 for shCtl in Fig. 3f but >1000 in Fig. 3i, <100 for shL2 in Fig. 3f but >100 in Fig. 3i).

In summary, considerable additional rigor and explanation are required to support the conclusion that ZFP36L2 "mediates colonic epithelial regeneration" (line 108).

3. The manuscript stretches the idea that "CRC metastases progressively adopt a non-canonical differentiation trajectory" (line 226). Evidence for this "trajectory" in shZFP36L2 organoids is weak. The significance of changes in ssGSEA scores, e.g., 0.4 to 0.43 or 0.37 to 0.39 (Fig. 3j), is unclear and the increase in CHGB immunostaining (Fig. 3p) is not impressive.

The display of small representative sections from ZFP36L2 wild-type and mutant CRC metastases (Fig. 3q-r) poses the same problem for evaluation as noted above. IF images don't seem to match "representative" numbers graphed to the right. To assess their claims, the authors should provide large tissue areas as supplementary files. Also, please explain the meaning of negative values on the y-axes in Figs. 3n and 3r. Considering what is represented (expression or % positive cells), negative numbers are not intuitive and likely reflect very small values in both cases.

Cancers, especially metastases, express many genes aberrantly. It is unclear if "non-canonical" differentiation (which includes CHGB, a product of native ISC progeny) reflects something as specific as the authors assume and is not just the tip of a broadly aberrant iceberg. Extended Data Fig. 5 is not especially informative. A clearer definition and persuasive demonstration of ZFP36L2-mediated differences would help.

4. To the best of my [limited] understanding, the point that the RNA-binding protein ZFP36L2 participates, along with RNA molecules, in phase-separated cytoplasmic condensates appears valid. The videos from live cell imaging are clear, though I'm unsure what exactly the authors mean by "dynamic" condensates. Cells are moving while being imaged and many of the IF puncta look similar across frames, likely reflecting cell movement. Perhaps this is why the authors refer to "dynamic, mobile condensates" but the significance of those descriptors is unclear. More importantly, the observation that condensates are more abundant in single cells than in organoids does not merit the conclusion that ZFP36L2 plays "a role in tumour cell adaptation" (line 289). Even if the short period of peak condensate formation coincides with "dedifferentiation," that is an association at best, and the authors don't consider the innumerable alternative reasons. Adding to the confusion, static (Fig. 4b) and live (Suppl. Videos) organoid imaging show many more condensates per cell than the graph in Fig. 4f indicates (1-2 per cell). In summary, the link between condensate formation and dedifferentiation is very weak.

5. Like other claims above, the one that "These data support a role for ZFP36L2 in mediating the termination of the stress response through degradation of stress-associated mRNAs" (lines 315-316) is a big stretch. Huge numbers of transcripts undergo natural decay, and the authors identify putative ZFP36L2 targets using an arbitrary parameter of log(2) 0.5; most "targets" bunch near this cut-off, and a more conservative cut-off would yield few targets (Extended Data Fig. 8d). In Fig. 5c, that cut-off is further reduced to log(2) 0.25, without explanation. At these levels, the risk of noise is substantially elevated and many replicate experiments would be required to distinguish consistent signals from background noise. There simply isn't enough support for the fanciful model illustrated in Extended Data Fig. 8e.

[redacted]

[redacted]

Typos: Line 172 should say "MSK125P" and not "OKG125P", line 184 should say "OKG146Li" and not "KG146Li".

Referee #3

(Remarks to the Author)
In this study by Jiang and colleagues, the authors investigate the role of ZFP36L2 in intestinal differentiation and stress responses. They use various mouse models and organoids models to show that ZFP is highly expressed in intestinal stem cells, upregulated during stress and that its expression induces a stem cell fate. They then go into the molecular mechanism

of ZFP function. They first show that ZFP forms biomolecular condensates, and that such condensates are most prominently observed after stress. They than identify mRNA targets of ZFP and show that such targets include many stress related genes, for which the mRNAs are degraded in a ZFP-dependent manner. [redacted]

Overall, this is a well-written and highly impressive study with an enormous amount of data, which span from mouse models, to sequencing, live-imaging [redacted]. Moreover, the study spans in vivo cancer biology to detailed cellular and molecular mechanisms, which I found highly impressive. Overall, the conclusions are well supported by the data, and the data is well explained. The molecular mechanisms are not very well resolved though, especially the role of ZFP condensates in ZFP function.

Specific points

While overall the manuscript is well written, I became progressively more lost during the manuscript on the role of stress in the study. In the initial parts of the manuscript, the focus was mainly on ZFP as a stem cell maintenance factor, while in the later parts, ZFP is presented mostly as a regulator of stress, which I found confusing. Are these two different functions, or are they two manifestations of the same function? It would be nice if the reader can be taken along more in the thinking throughout the manuscript in this regard. For example, ZFP is highly expressed in unstressed ISC's, why? Is this related to stress somehow, or unrelated?

ZFP36L2 mutations are frequently observed in cancer and the authors suggest that such mutations are likely loss-of-function mutations. In this study the authors use ZFP36L2 knockouts or knockdowns to eliminate the protein entirely. It would be good to know if the mutations indeed are complete loss of function alleles, or whether a truncated protein is expressed. Can they perform a western blot on the N-terminal domain from cancer samples to see if the N-terminus is expressed or not? Alternatively, but less good, they could perform a qPCR to see if the mRNA is lost due to nonsense mediated mRNA decay. If the N-terminus is expressed, it could be nice to see if it acts as a dominant negative in ZFP function.

Figure 4E. The authors tag ZFP with GFP, but I didn't see which GFP variant it is. A recent study illustrated the importance of the fluorescent protein for condensate formation (doi.org/10.1101/2024.10.04.616694). Can the authors confirm their results with a fluorescent protein that does not perturb condensate formation (or are they already using one)?

In figure 4 the authors show that ZFP forms condensates upon a variety of cell stress triggers. At the same time, ZFP levels (at least endogenous levels, unclear for exogenous levels) change. It is well known that condensate formation is highly dependent on protein levels of condensate inducing proteins, so it is possible that condensate formation is simply a consequence of altered ZFP protein levels. To what extent is the formation of ZFP condensates dependent on ZFP protein levels changes (both endogenous and exogenous) during single cell plating or other stressor? Does ZFP form more condensates under stressed conditions, even at similar total expression levels (including endogenous levels)?

Hypertribe was performed in CRC cancer organoids instead of in normal organoids. I'm wondering whether the targets identified by these experiments all represent the mRNA targets during normal physiology, or whether a subset of the mRNA targets are cancer specific? Can some of these be validated in non-cancer setting?

Figure 5C. The authors measure mRNA half-life using actinomycin D. Transcriptional inhibition in this assay likely causes a substantial amount of stress, and ZFP is highly stress responsive, so I don't think this is a good assay. Much better is to use SLAM-seq (or perhaps a PCR-based variation as validation).

I find it quite hard to see how good the overlap is in fig 5C compared to what would be expected by random chance. ZFP binding is seen for both mRNAs that are upregulated and downregulated mRNAs. Can the authors provide a more quantitative metric for this overlap and enrichment?

To understand the role of ZFP condensates a bit better, it could be nice to examine whether mRNAs that are degraded by ZFP are enriched in ZFP condensates? And if so, during which phase of stress or repair. This would link condensate formation to ZFP-dependent mRNA decay of its mRNA targets. In this context, it could also be nice to examine if any mRNA decay factors (or P-body markers) co-localize with ZFP condensates, and whether the protein interactors of ZFP are localized to ZFP condensates (since the authors are claiming that condensates induce protein-protein interactions, see below).

I'm not entirely convinced that the ZFP condensates are really moving into the nucleus. It seems a bit hard to imagine how a condensate of that size could fit in it's entirely through a nuclear pore. Moreover, the condensate in the examples only spends 1 or 2 frames co-localizing with the nuclear H2B signal and it is unclear to me why the condensate would go in and then immediately go out again. Rather than moving into the nucleus and immediately out of the nucleus again, could the condensate be just above or below the nucleus, such that they appear co-localized even in a single Z-slice? Do the authors see condensates going into the nuclear and that moving around the nucleus for prolonged periods of time? Also, it is unclear how reliable an Imaris rendering is, do the authors fully understand the computational corrections that are performed to create this images? I think raw data should be used to show convincing condensate localization to the nucleus.

[redacted] in the first paragraph of the discussion they claim to have shown that ZFP condensates induce protein-protein interactions. But in fact, they do not show that condensates have any role in mediating protein-protein interactions, just that ZFP might stimulate protein-protein interactions. The authors could consider to try to use the recently published condensate kill switch (Zhang et al., 2025 Nature) if they wish to make a statement about the

importance of ZFP condensates for all the reported phenotypes, in addition to showing co-localization of target mRNAs and proteins to condensates

Referee #4

(Remarks to the Author)
I co-reviewed this manuscript with one of the reviewers who provided the listed reports.

Version 1:

Reviewer comments:

Referee #1

(Remarks to the Author)

The authors have provided further clarity in the presentation of their data in the revised manuscript in response to the suggestions of the reviewers. A highly comprehensive point by point response to all the queries has been provided. The authors have also conducted numerous additional experiments to either reinforce, add quantitative data or provide further insight to their study. Numerous amendments to the text, Figures and Figure legends have been provided. The authors have adequately addressed the reviewer's comments and provide a highly original and significant manuscript supported by quality data and robust discussion.

Referee #2

(Remarks to the Author)
My original review raised concerns about this study in 5 broad categories, and the authors have addressed each of these concerns in considerable detail. My detailed review follows. Briefly, my concerns in categories 2, 4, and parts of 5 are thoroughly addressed for the most part, though additional small wording changes would improve the manuscript further. My persistent concerns in categories 1 and 3 can be addressed by changes to the text and/or figures without additional experiments. [redacted]

1. Excerpts from my original review include “ZFP36L2 seems to be expressed in many crypt epithelial (except the most mature) and almost all tumor cells”; “In summary, Fig. 1 and Extended Data Figs. 1 and 2 seem intended to defend interest in ZFP36L2 and build a connection to AP-1 complexes, but the data are murky and don’t support the implied conclusions”; and “Mutation in 5-10% of CRCs is arguably sufficient justification to study ZFP36L2 functions”. As these remarks indicate, I understand that these data do not address mechanism and simply set the stage to investigate ZFP36L2 functions.

The authors agree that “these figures were not designed to establish mechanistic conclusions, but rather to elaborate hypotheses” (rebuttal, p. 20 bottom). Data in Fig. 1 and Extended Data Fig. 1c show ~2-fold range in ZFP36L2 mRNA between normal human intestinal stem (ISCs) and terminally differentiated cells. In mouse colonic crypts, ZFP36L2 is expressed in most (all?) TA cells (Fig. 2b) and the idea that this is because they “exist in a poised, injury-responsive state” makes little sense. The leap from ZFP36L2 expression to a “regenerative” cell state (especially in normal epithelium) or to “metastasis seeding markers L1CAM and EMP1” (Extended Data Fig. 2; P values are less useful here than observing the shape of violin plots) remains tenuous. These are conceptual weaknesses of the study but not fatal flaws. In my opinion, originally and now, the presence of ZFP36L2 mutations in up to 10% of colorectal cancers (CRCs) adequately justifies studying its functions.

2. Technical problems with experiments on ZFP36L2 requirement in intestinal regeneration. All my concerns are addressed thoroughly.
(a) DSS colitis usually occurs in patches. The authors clarify that their protocol with 3.5% DSS induces uniform damage. Moreover, the authors now support their arguments with another injury model (Lgr5-DTR mice).
One remaining small point. If the authors agree that “the first two images in Fig. 2c appear similar to control,” then why claim that “ZFP36L2 expression was markedly increased by day 2 …” (line 117)? Even Suppl. Data 1 and 3 don’t support that claim. Moreover, the claim doesn’t help or hurt the authors’ central arguments.
(b) Villin-Cre expression is patchy in the colon. The authors have addressed this concern with appropriate care.
(c) Lgr5+ ISCs are selectively ablated in Lgr5-DTR mice. Why, then, are all ZFP36L2+ cells missing on Day 7 (Extended Data Fig. 3a)? The authors clarify nicely, show larger areas of tissue, and quantify cell positions.
(d) The reduction of Lgr5+ ISCs in ZFP36L2-KO mice is intriguing and should be fleshed out. Can the authors explain and demonstrate it more persuasively? The authors have addressed this question very well.
(e) The dedifferentiation assay in organoids is not widely accepted as standard and there are many explanations for the findings (Fig. 2h-k), such as inherent epithelial defects unrelated to dedifferentiation. Addressed, as also noted above, with

an independent in vivo model of ISC ablation.
(f) The authors did use two different dox-inducible shRNAs in the study, which is nice but does not strictly exclude off-target effects. cDNA rescue of shRNA-treated organoids was not successful but the authors do a nice job with independent CRISPR/Cas9 deletion of Zfp36l2.
(g) Captions indicate that Figs. 3f and 3i refer to the same data, but the numbers at week 13 don't match. The authors have cleared the misunderstanding.

3. The manuscript stretches the idea that "CRC metastases progressively adopt a non-canonical differentiation trajectory" (line 226). … IF images don't seem to match "representative" numbers graphed to the right. The authors should provide large tissue areas as supplementary files. Also, please explain the meaning of negative values on the y-axes … likely reflect very small values in both cases. … It is unclear if "non-canonical" differentiation (which includes CHGB, a product of native ISC progeny) reflects something as specific as the authors assume and is not just the tip of a broadly aberrant iceberg.

I accept that CHGB and CK5 mark "non-canonical" differentiation, as the group's previous work defined. Neither the rebuttal nor the revised manuscript address my concern about significance of ssGSEA score changes from 0.89 to 0.88 or from 0.36 to 0.38 (Fig. 3j), which could easily represent noise. CHGB+ cell fractions (25th to 75th quartile) in ZFP36L2-MUT metastases were <0.1% to 1% and in half the cases, CK5+ cell fractions were <0.01% (Fig. 3q-r). These numbers don't make a strong case for the thrust of Fig. 3, which is that absence of ZFP36L2 favors "non-canonical differentiation". This conclusion should be toned down or revised to match the data.

4-5. The point that the RNA-binding protein ZFP36L2 participates, along with RNA molecules, in phase-separated cytoplasmic condensates appears valid, and videos from live cell imaging are clear. … but the link between condensate formation and dedifferentiation is weak. … Some data are noisy [redacted]

The authors' use of drift correction and quantitative tracking adds the desired level of rigor to describe the condensates as "dynamic". The temporal association of ZFP36L2 condensates with dedifferentiation is strengthened. Cut-off thresholds are made consistent, SLAM-seq is used to corroborate findings. [redacted] These changes improve the study, as do new data that localize putative ZFP36L2 target mRNAs in ZFP36L2-containing condensates (Fig. 5g-h).

[redacted]

Referee #3

(Remarks to the Author)
In the revised manuscript the authors have addressed all my questions and concerns very nicely and I support publication of this excellent study. I have one small comments on the newly added experiments and one on the text. I don't think the experiment is absolutely essential, but it would be nice to add.
Fig. 5g,h It would be nice to see a control mRNA that is not associated with ZFP36L2 in these experiments.

I was a bit confused by the explanation of these experiments (lines 386-390): co-localizaiton of target mRNAs is observed strongly in the unstressed condition, but then severely reduced during stress. The authors state that stress induces ZFP36L2 expression, which binds to target mRNAs and induces their degradation. However, if this were true, would one not expect the target mRNAs to enrich to ZFP36L2 foci during stress? Are the authors thinking that the co-localization during stress is reduced, because the target mRNAs are degraded by ZFP36L2 foci during stress? This text could be clarified a bit.

Reviewed by Marvin Tanenbaum

Referee #4

(Remarks to the Author)
I co-reviewed this manuscript with one of the reviewers who provided the listed reports.

Version 2:

Reviewer comments:

Referee #2

(Remarks to the Author)
The authors have accepted the recommendation to remove the speculative and under-developed pieces of the story previously presented in Fig. 6. The revised manuscript also includes additional corrections and clarification. No further revision is necessary. The authors' extensive characterization of ZFP36L2 will make a nice contribution to the field.

Referee #3

(Remarks to the Author)
All my comments have been adequately addressed

Open Access This Peer Review File is licensed under a Creative Commons Attribution 4.0 International License, which permits use, sharing, adaptation, distribution and reproduction in any medium or format, as long as you give appropriate credit to the original author(s) and the source, provide a link to the Creative Commons license, and indicate if changes were made.
In cases where reviewers are anonymous, credit should be given to 'Anonymous Referee' and the source.
The images or other third party material in this Peer Review File are included in the article's Creative Commons license, unless indicated otherwise in a credit line to the material. If material is not included in the article's Creative Commons license and your intended use is not permitted by statutory regulation or exceeds the permitted use, you will need to obtain permission directly from the copyright holder.
To view a copy of this license, visit https://creativecommons.org/licenses/by/4.0/

Response to Reviewers' Comments

We would like to thank the editor and reviewers for their supportive and constructive comments, and for highlighting points that were unclear in our manuscript. The extensive new experiments and revisions that we have undertaken in response have greatly improved the paper.

We emphasize that this study identifies ZFP36L2 as a critical molecular switch driving the de-differentiation of cells during intestinal regeneration—a process that is recapitulated during colorectal cancer (CRC) metastasis. We demonstrate for the first time that ZFP36L2 forms RNA-dependent condensates and promotes the decay of stress-associated mRNAs [redacted] to reprogram differentiated cells into a stem cell state. This dual function allows ZFP36L2 to terminate the stress response and facilitate the regenerative plasticity required for metastatic seeding.

In response to the predominant and overlapping reviewer concerns, we now:

- Supplement our *in vivo* DSS colitis and *ex vivo* organoid experiments with an orthogonal **Lgr5-DTR** mouse model to definitively demonstrate that ZFP36L2 is functionally required for dedifferentiation into an ISC state upon the loss of LGR5+ cells.
- Provide HyperTRIBE profiling of ZFP36L2-bound mRNAs in **human normal colon organoids**, establishing that its role in stress sensing and regenerative buffering is conserved between normal and tumor epithelial cells.
- Utilize **SLAM-seq** (thiol-linked alkylation for metabolic sequencing) to directly measure transcript-specific half-lives, confirming ZFP36L2's role in promoting the turnover of stress-responsive transcripts.
- Incorporate **CRISPR-Cas9** loss-of-function validation in multiple patient-derived organoid lines to definitively rule out shRNA off-target effects regarding regenerative plasticity.
- Quantify **epithelial lineage markers** (MUC2, LGR5) across large tissue areas and whole Swiss rolls to provide a rigorous assessment of crypt architecture and stem cell populations.
- Enhanced our **live-cell imaging** with drift correction and puncta tracking to demonstrate that ZFP36L2 forms bona fide dynamic, mobile condensates that undergo fusion and fission.
- Perform multiplex mRNA FISH/ZFP36L2 IF to visualize dynamics of **target mRNA colocalization in ZFP36L2 puncta** during organoid regeneration, providing orthogonal imaging based validation of the role of ZFP36L2 in terminating stress responses.
- Analyze a large clinical whole-genome sequencing and transcriptome cohort (n=1,063) to show that *ZFP36L2* mutations associate with **inferior relapse-free survival** in specific transcriptional subtypes of CRC.
- Perform additional experiments to probe the function of ZFP36L2 puncta, including testing **monomeric EGFP fusions** and testing the **GFP nanobody condensate killswitch** system.

In addition, in response to the reviewers' valuable feedback, we have **edited the text** to clarify:

- the relationship between the stress response, intestinal stem cell maintenance in both homeostasis and stress conditions, [redacted]
- [redacted]

We provide a detailed point-by-point response below. Excerpted text from the manuscript appears indented, with new text highlighted in red, and figure callouts refer to figure numbers in the revision, unless otherwise specified. New figure panels are listed below, other figure changes are indicated with red boxes in the marked up version of the manuscript.

New figure panels (numbered as in revision)

Fig. 2 g,h – *ZFP36L2* ko abrogates Lgr5+ ISC restoration after *Lgr5*-DTR-GFP ablation *in vivo*.
Fig. 4e – Drift corrected tracking of ZFP36L2 condensate motion.
Fig. 4j – Condensate formation by EGFP, ZFP36L2-EGFP and ZFP36L2-mEGFP.
Fig. 5e,f – SLAMseq in MSK107Li shCtrl and sh*ZFP36L2* organoids.
Fig. 5g,h – Colocalization of ZFP36L2 with target mRNAs.
[redacted]
Extended Data Fig. 3f – Quantification of ZFP36L2, *Lgr5* and MUC2 in the mouse colon.
Extended Data Fig. 4a-d – *ZFP36L2* ko abrogates Lgr5+ ISC restoration in organoids.
Extended Data Fig. 4h,i – Quantification of *Lgr5*-GFP depletion during dedifferentiation assay.
Extended Data Fig. 7a-d – *ZFP36L2* CRISPR knockout inhibits CRC dedifferentiation.
Extended Data Fig. 8i – ZFP36L2 protein levels during organoid regeneration and stress.
Extended Data Fig. 10b-d – HyperTRIBE in normal colon organoids.
Extended Data Fig. 11c – Comparison of ActDseq and SLAMseq ZFP36L2 mRNA targets.
Extended Data Fig. 12 d, e – Colocalization of ZFP36L2 with stress granule/ P-body markers.

Reviewer #1

This study by Jiang et al investigates the mechanism underlying the de-differentiation of cells known to occur during intestinal regeneration injury that is also recapitulated during colorectal cancer metastasis. Although it has been clearly demonstrated in numerous studies that when Lgr5 positive cells are lost, differentiated cell types undergo a reprogramming process that leads to the replacement of Lgr5-positive cells, the trigger for this de-differentiation has not been identified. This study fills this gap by identifying a key molecule ZFP36L2 driving this process. Furthermore, the authors provide a link to CRC metastasis by showing ZFP36L2 knockdown significantly reduced metastatic burden and inhibits de-differentiation. Finally, the authors examine the mechanism of ZFP36L2 action and demonstrate the ability of ZFP36L2 to form RNA-dependent condensates and pinpoint a specific role in promoting mRNA decay of stress-associated mRNAs including FOSL2, JUND, ATF4. [redacted]. This revealed a dual function for ZFP36L2 in terminating the stress response and promoting de-differentiation. Overall, this is an outstanding study both in the breadth in defining function spanning regeneration and tumour metastasis and the robust methodology that defines the underlying mechanisms.

The key findings from this study are that:

1. ZFP36L2 is highly associated with stemness in both the normal colon and CRC, is associated with the regenerative RevCSC signature and mutated in around 5-10% of CRCs.

2. ZFP36L2 is up-regulated during regeneration following injury in the DSS mouse model and knockout revealed a key functional role in regeneration.

3. Knockdown of ZFP36L2 in human CRC organoid models reduces metastatic burden and blocks the de-differentiation state.

4. ZFP36L2 binds and promotes the decay of mRNAs associated with stress response and therefore has a key role in terminating the stress response.

[redacted]

This is likely to be of considerable interest to researchers studying regeneration of the colonic epithelium following injury and mechanisms of cancer cell plasticity in colorectal cancer. The specific function of ZFP362 has not been defined previously. The results of this study will also have interest more broadly in other regenerative models and solid cancers where similar mechanisms may be observed.

Data and Methodology: The outstanding strengths of this study are the breadth of models utilising both mouse and human models of regeneration and tumour metastasis. The models encompass meticulous analysis of the [redacted] temporal patterns of expression of ZFP36L2 with other markers alongside functional studies in mouse and human models. For some of the conclusions suggested by the authors, lineage tracing should have been included. [redacted]

Use of statistics: The experiments are accompanied by detailed explanations in the figure legends of sample size and statistical analyses. These were mostly robust with some exceptions outlined in the specific comments.

Discussion and Conclusions: The discussion is an accurate summary of the body of work with reference to the literature. The manuscript is very well written.

We thank the reviewer for their thoughtful and highly positive evaluation of our study, and for highlighting the broad significance of our findings for intestinal regeneration and colorectal cancer plasticity. We are encouraged that the referee finds our identification of ZFP36L2 as a trigger for injury-induced de-differentiation, and our mechanistic framework linking RNA-dependent condensates, stress-mRNA decay, [redacted], to be robust and compelling. We address their specific comments in line below:

Specific Comments and suggested improvements

R1.1. Line 69. Add Fig 1a

We have now added the figure citation (**Fig. 1a**) at this location (revised manuscript, line 69).

R1.2. Figure 1B It is not clear which cell population was utilised to calculate mean expression. Could this be outlined or highlighted within the figure?

The mean expression indicated by the bar plots in Fig. 1b was calculated within the **human normal crypt intestinal stem cell (ISC) population**. We have now explicitly indicated the cell population used directly in the figure and clarified this in the figure legend (revised Fig. 1b; legend).

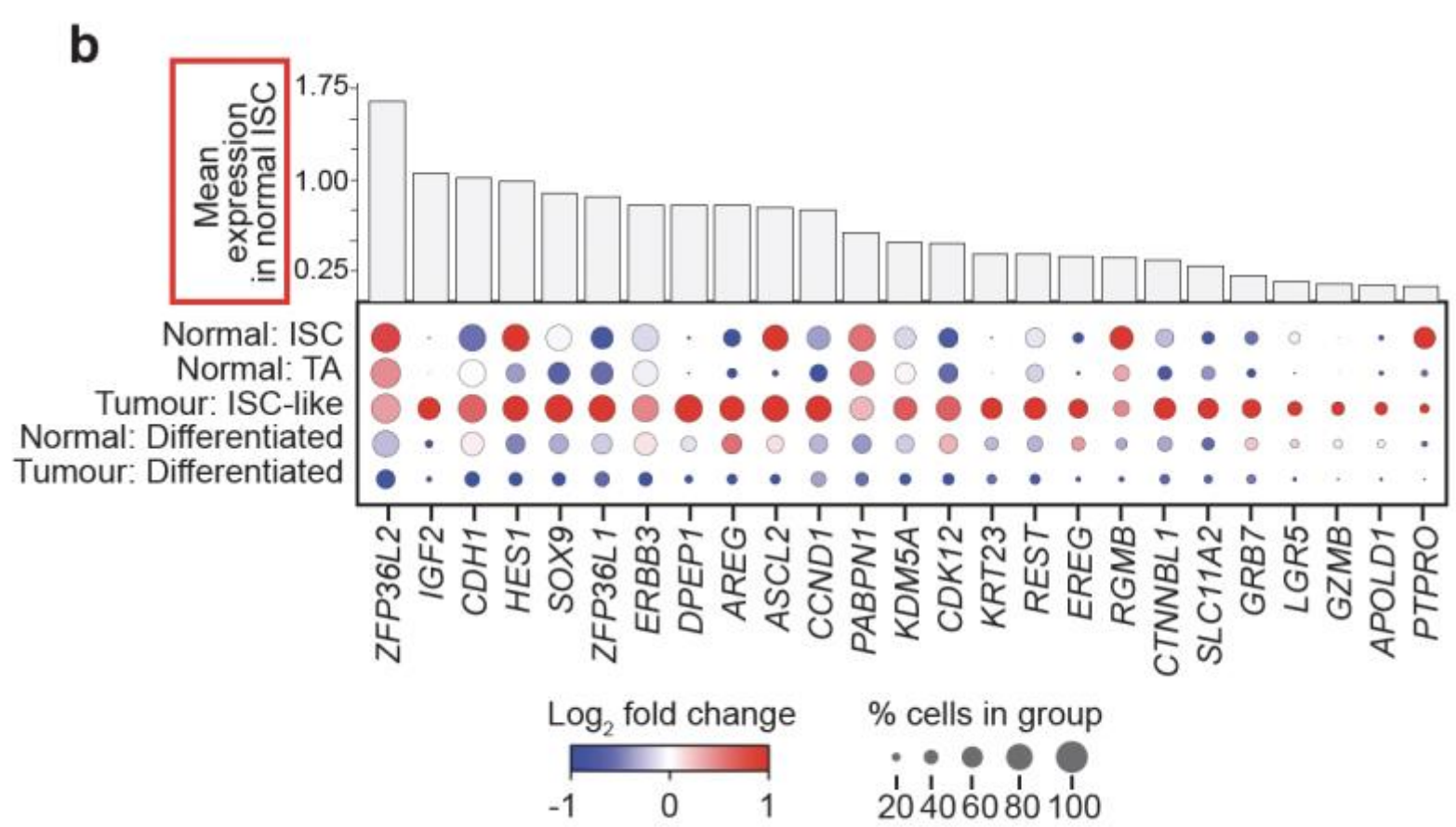

Fig. 1b. Dot plot showing expression of tumour ISC-like module genes in matched normal colon (Normal) and colorectal cancer (CRC; Tumour) epithelial cells from 25 patients. The tumour ISC-like module comprises a covarying set of 54 genes identified using Hotspot analysis of local gene–gene covariance across epithelial cells from primary and metastatic CRC samples[1] (n=28 patients). Dot size represents the percentage of cells within each epithelial subtype (rows) expressing each gene (columns) of top ranking genes. Coloured scale indicates the $\log_2$ fold change in average gene expression across cell types. Bar plots display mean expression of each gene across normal colon ISC populations. Genes were filtered for statistical significance using the Wilcoxon rank-sum test (ISC versus all other epithelial subtypes) with FDR correction (FDR<0.05). ISC: intestinal stem cell; TA: transit amplifying.

R1.3. Line 85 The mutational profile of ZFP36L2 is provided but does this associate with clinical outcome or molecular sub-types of CRC?

To address this question, we sought to analyze human CRC datasets with both ZFP36L2 mutation information and clinical outcome annotation and/or bulk RNAseq data to enable us to calculate consenus molecular subtypes (CMS). ZFP36L2 mutations are not detected as a part of standard clinical tumor gene panel sequencing assays such as MSK-IMPACT or Foundation One, and large-scale outcome-linked CRC sequencing datasets with ZFP36L2 mutation calls have only recently become available. In small outcome-annotated CRC whole exon sequencing cohorts from TCGA (n=570) and MSKCC (n=267), we did not detect a significant association between ZFP36L2 mutation and survival in either MSI-H or MSS CRC (**Fig. R1a**,**b**). On the other hand, in a larger WGS+transcriptome cohort (Nunes et al., *Nature*, 2024. n=1,063)[2], ZFP36L2 is highlighted as a previously unrecognized mutational event in CRC, enriched in hypermutated CMS1 tumors, but also present across all non-hypermutated MSS subtypes. ZFP36L2 mutations associate with inferior relapse-free survival (RFS) in specific transcriptional subtypes (subtype-stratified Cox models), even though mutation frequency itself is not necessarily enriched in those subtypes (**Fig. R1c**). Notably, this adverse association was subtype-specific (most evident in CMS4/CRPS4) rather than uniformly detected across the overall cohort (**Fig. R1d**). Our prior work showed that non-canonical differentiated transcriptional modules correspond to CMS4 and are associated with poor clinical outcomes[1], consistent with our observations in the present manuscript showing that loss-of-function ZFP36L2 mutations promote non-canonical differentiation. While larger cohorts of clinical CRC tumor sequencing data with stage matching and clinical outcome annotation will be required to robustly characterize the association of ZFP36L2 mutation with clinical outcomes, the data from Nunes et al., *Nature*, 2024 support a model in which ZFP36L2 loss is associated with poor-prognosis tumour states.

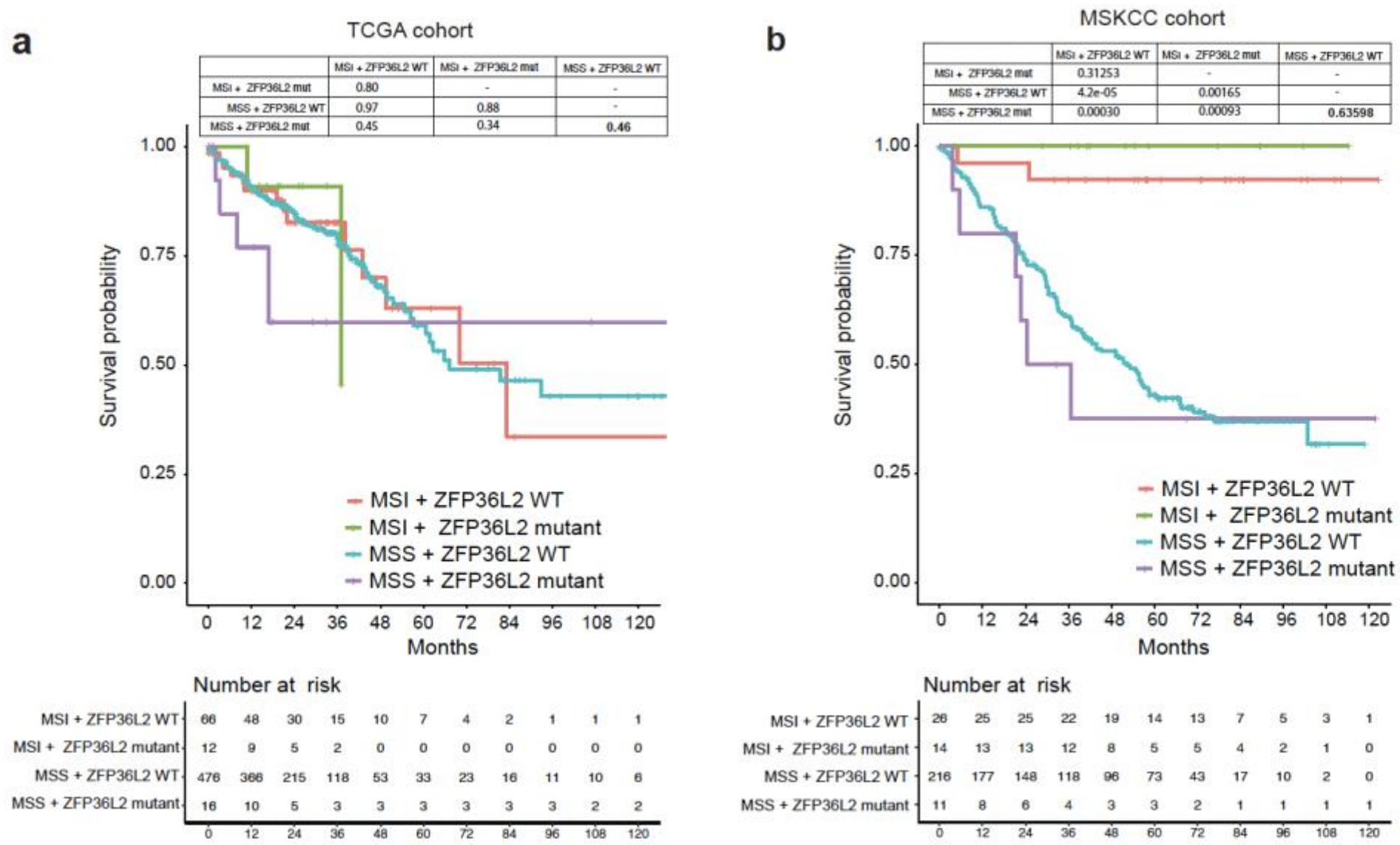

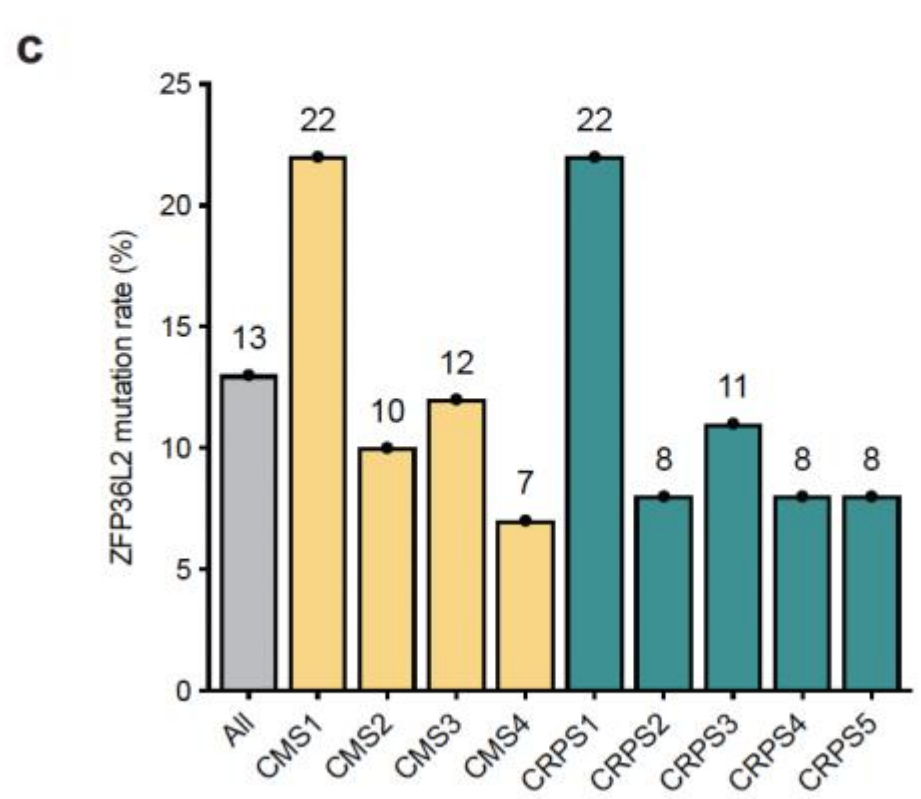

d

Subtype-stratified association of ZFP36L2 mutations with relapse-free survival (RFS) in non-hypermutated CRC

Tumour type	Gene	HR (univariable)	HR (multivariable)
nHM CRPS2	ZFP36L2	2.141 (1.025-4.471, p=0.0428)	2.278 (1.063-4.883, p=0.0342)
nHM CRPS4	ZFP36L2	3.857 (1.367-10.882, p=0.0108)	3.672 (1.173-11.494, p=0.0255)
nHM CMS4	ZFP36L2	3.261 (1.485-7.164, p=0.0032)	3.237 (1.404-7.462, p=0.0058)

Fig. R1 | Association of ZFP36L2 with clinical outcome and molecular sub-types of CRC. a,b, Kaplan–Meier analyses of survival probability in the TCGA cohort (**a**) and the MSKCC cohort (**b**), with patients stratified by microsatellite instability status (MSI or MSS) and ZFP36L2 mutation status. **c**, Prevalence of ZFP36L2 mutations in distinct consensus molecular subtypes (CMS) and CRC intrinsic prognostic subtypes (CRPS). Data were adapted from Nunes, Li, Wu et al., Nature, 2024[2]. **d**, Univariable and multivariable hazard ratios for the association between ZFP36L2 mutations and relapse-free survival in subtype-stratified non-hypermutated colorectal cancer. Data were adapted from Nunes, Li, Wu et al., Nature, 2024[2].

R1.4. Line 108 The conclusions from this section would be more convincing if lineage tracing from ZFP36L2 cells was conducted.

We appreciate this comment from the reviewer. In our initial submission we generated a mouse model in which we deleted Zfp36L2 in the murine intestinal epithelium (*Villin*^{Cre}/*Zfp36L2*$^{fl/fl}$) (**Fig. 2**, **Extended Data Fig. 3**). By tracking the recovery of the mouse intestinal epithelium in Zfp36l2 wt and knockout animals following DSS colitis injury *in vivo*, as well as through tracking of

dedifferentiation into an Lgr5+ ISC state and functional organoid regeneration from *Zfp36l2^IEC^*, *Zfp36l2^HET^* and *Zfp36l2^WT^* colon epithelial cells, we concluded that "ZFP36L2 mediates colon epithelial regeneration through injury-induced dedifferentiation into an ISC state." While the knockout data support this claim, lineage tracing would expand the scope of our study from assessing the cellular function of ZFP36L2, the focus of the manuscript, to ask whether the Zfsp36l2-expressing cell state, potentially with features beyond Zfp36L2 expression, is the cell state of origin for intestinal epithelial regeneration. Testing this new hypothesis would require generating novel genetically engineered mouse models and multi-generational breeding to establish Zfp36l2 knockout in this context, necessitating extended timelines, and is beyond the scope of the current study.

Nevertheless, we appreciate the reviewer's suggestion to more rigorously assess whether Zfp36l2 is **functionally required** for de-differentiation into an ISC state upon the loss of Lgr5+ cells. To directly address this question, we have now supplemented our *in vivo* DSS colitis (**Fig. 2d**) and *ex vivo* organoid formation experiments (**Extended Data Fig. 4g-l**) with an orthogonal *in vivo* LGR5+ ISC ablation approach. We generated $Lgr5^{DTR\text{-}GFP+}/Zfp36L2^{IEC}$ and $Lgr5^{DTR\text{-}GFP+}/Zfp36L2^{WT}$ mice, administered diphtheria toxin to selectively ablate Lgr5+ ISCs, and assayed the restoration of Lgr5 expression through dedifferentiation of Lgr5- cells in Zfp36L2 WT vs knockout contexts both *in vivo* and in *ex vivo* organoids (**Fig. R2**). Consistent with our prior observations, we find that loss of Zfp36l2 impaired recovery of Lgr5+ crypt base intestinal stem cells following the ablation of Lgr5+ cells. Our findings now across two independent *in vivo* mouse models of Lgr5+ ISC loss (DSS colitis and Lgr5-DTR) and in *ex vivo* organoid dedifferentiation and regeneration assays, definitively support our conclusion that Zfp36l2 is critical for injury-induced de-differentiation. These new data and quantifications have been added to the revised manuscript (**Fig. 2g,h; Extended Data Fig. 4b-f;** Results, page 5, lines 143-161; Methods, page 65, lines 1432-1460).

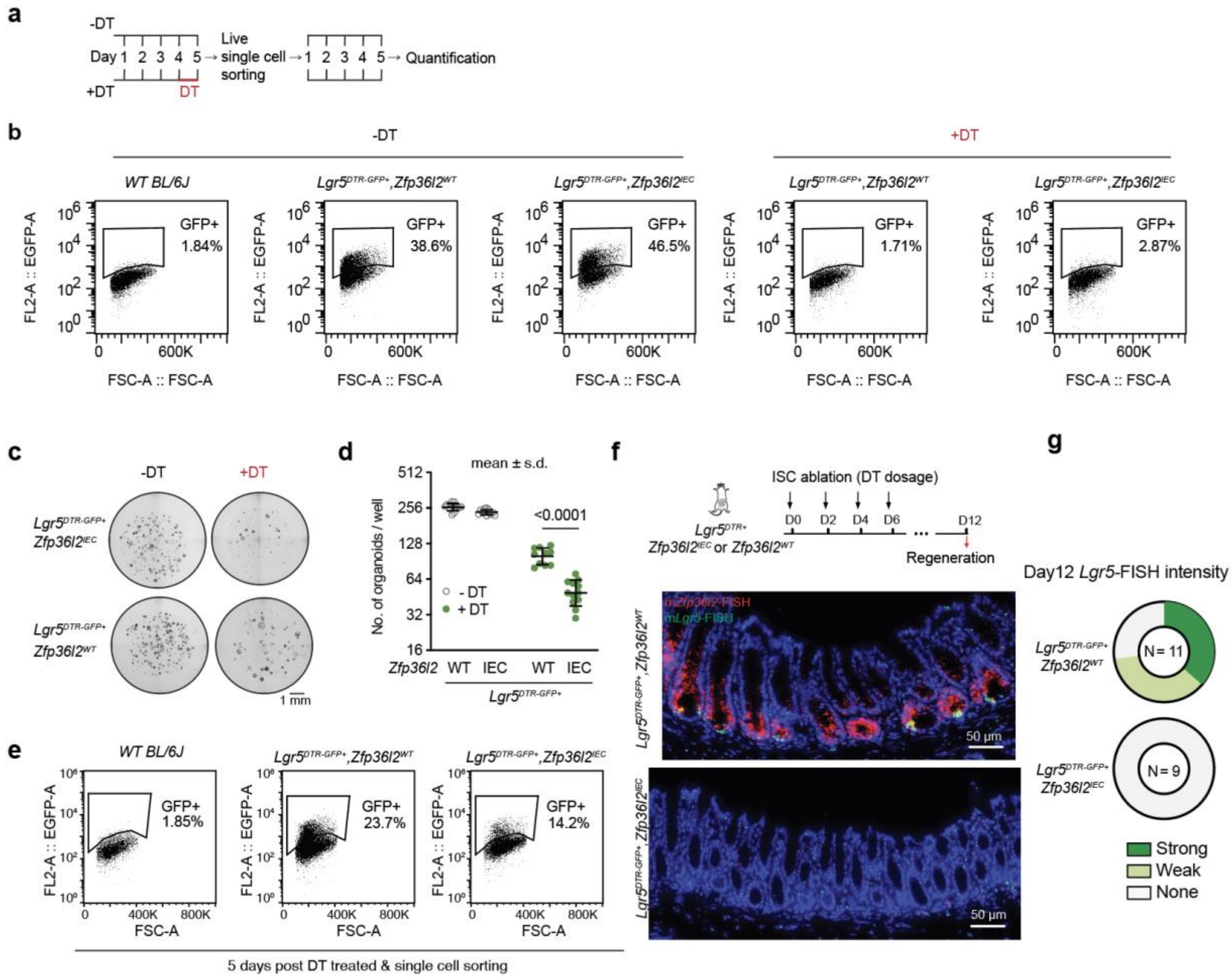

Fig. R2 (Fig. 2g,h;Extended Data Fig. 4b-f) | *Zfp36l2* is required for dedifferentiation into ISC cells. a, Schematic of LGR5-DTR ablation and organoid dedifferentiation assay. Colon organoids were derived from 6–8-week-old *Lgr5*$^{DTR-GFP-}$, *Zfp36l2*^{WT}; *Lgr5*$^{DTR-GFP+}$, *Zfp36l2*^{WT} and *Lgr5*$^{DTR-GFP+}$, *Zfp36l2*^{IEC} mice, and maintained in mouse intestinal stem cell medium (mWRENAFI). After 4 days in culture, diphtheria toxin (DT; 0.6 µg/mL) was added to the +DT group for 24 h. DT-treated and untreated organoids were then dissociated, live cells were harvested by sorting, and single cells were reseeded at low density (2,000 cells per 40 µL Matrigel per well) in mWRENAFI to induce dedifferentiation. Regenerated organoids (organoids per well) were quantified 5 days after reseeding. **b**, Representative flow cytometry density plots showing the frequency of LGR5-DTR-GFP positive cells in organoids with or without 24 h DT treatment, measured at day 5 (the time point used for sorting in the dedifferentiation assay). **c**, Representative brightfield images of regenerated organoids in individual wells 5 days after reseeding of live single cells in mWRENAFI. n = 3 mice per genotype; 3–4 wells per mouse. Scale bar, 1 mm. **d**, Quantification of regenerated organoids per well from (**c**). Data are mean ± s.d. Two-tailed Mann–Whitney *U* test. **e**, Representative flow cytometry density plots showing regeneration of LGR5-DTR-GFP positive cells following reseeding of live single cells from the DT treated group. **f**, Schematic of *in vivo* DT administration to ablate LGR5-DTR-GFP+ colon ISCs and assess ISC regeneration. Colon Swiss rolls were collected at day 12 after the first DT dose (top). Dual mRNA FISH for *mLgr5* and *mZfp36l2* was performed on day-12 Swiss-roll sections to evaluate ISC regeneration (bottom). **g**, Quantification of the number of mice showing regeneration of *Lgr5*-FISH signal

at day 12 in the experiment shown in Fig. 2g. Strong indicates complete regeneration; Weak indicates patchy recovery of *Lgr5*-FISH signal; None indicates no recovery of *Lgr5*-FISH signal.

R1.5. Figure 2b There is a significant number of cells expressing ZFP36L2 during homeostasis. Can the authors comment on a potential role here?

We thank the Reviewer for drawing attention to this important point, also raised by Reviewer 3. During homeostasis, ZFP36L2 expression is primarily observed in the ISC and transit-amplifying (TA) compartments (**Fig. 2a,b**). As we showed in the original submission, $Villin^{CRE}Zfp36L2^{fl/fl}$ animals contain fewer Lgr5+ ISCs in their intestinal epithelia, suggesting that Zfp36L2 plays a role in maintaining the ISC pool under homeostatic conditions (**Extended Data Fig. 3e-i**). Importantly, intestinal epithelial Zfp36l2 knockout animals did not exhibit overt defects in baseline crypt architecture or body weight, and crypt-derived organoids lacking ZFP36L2 showed no gross impairment in growth or maintenance under homeostatic conditions (**Extended Data Fig. 3b,d**). These data suggest that ZFP36L2 is not critically required for intestinal epithelial maintenance during homeostasis, at least under the conditions tested.

To further define the cellular function of ZFP36L2 in the normal colon as prompted by the reviewer, we have now performed ZFP36L2 HyperTRIBE in human normal colon organoids (**Fig. R3; Extended Data Fig. 10b-d**). In normal colon organoids, ZFP36L2 target sites were predominantly enriched in 3'UTRs and associated with AU-rich/UAUUUA motifs (**Fig. R3a; Extended Data Fig. 10b**), consistent with ZFP36L2 RNA targets in tumor organoids (**Fig. 5a; Extended Data Fig. 10b**). Gene ontology analysis of ZFP36L2-bound transcripts in normal organoids continued to highlight stress-response–associated programs, and comparison of ZFP36L2-bound transcripts in normal (OKG146N) vs. tumor (OKG146P/Li) organoids from the same patient revealed large overlap (**Fig. R3b; Extended Data Fig. 10c,d**). Analysis of the genes that were differentially bound in tumor vs. normal revealed that the differences could be largely explained by context-dependent expression of a slightly different subset of transcripts belonging to similar gene programs, rather than a change in binding specificity or cellular function *per se* (i.e., targets detected preferentially in normal were often low/absent in tumor, and vice versa) **(Fig. R3c; Extended Data Fig. 10c)**. Overall, the consistency in ZFP36L2 mRNA binding in both normal and tumor intestinal epithelial cells further reinforces our model that ZFP36L2's principal role is engaged upon stress/injury to terminate stress signaling and facilitate regenerative de-differentiation. Our data support a functional link between the stress response, dedifferentiation and stem cell maintenance. a function that is active, in the normal intestinal epithelium to maintain the ISC population, but is not essential for epithelial function in homeostatic conditions. Instead, the function of ZFP36L2 in stress-responsive ISC pool replenishment becomes critical for epithelial regeneration following injury, and is co-opted to drive tumor regeneration during tumor progression and metastasis.

Regarding the question of why ZFP36L2 is highly expressed in unstressed ISCs, we note that intestinal stem cells exist in a poised, injury-responsive state and are highly sensitive to environmental fluctuations including levels of metabolites, cytokines, growth factors derived from the stromal, immune and microbial cell populations of the gut[3–9]. Basal steady-state ZFP36L2 expression in ISCs may therefore reflect a preparedness to buffer transient stress-associated transcriptional programs and maintain stem cell identity. Emerging studies suggest that intestinal

progenitor populations and more differentiated cells, including isthmus / TA-adjacent progenitors and enteroendocrine cells[10,11,12], can functionally replenish ISC pools and display dedifferentiation capacity even under homeostatic steady-state conditions.

We have revised the manuscript to clarify this conceptual link between stress response and maintenance of the ISC pool, and to guide the reader more explicitly through this reasoning (Page 12, lines 418-422). We thank the reviewer for this insightful line of questioning that has prompted us to further strengthen the conceptual framework and narrative of our manuscript.

> Intriguingly, comparison of ZFP36L2 HyperTRIBE targets in normal colon organoids (OKG146N) with those in the tumour organoids from the same patient (OKG146P, OKG146Li) identified broadly similar targets in tumour and normal (**Extended Data Fig. 10c,d**) suggestive of a relationship between epithelial stress response and ongoing dedifferentiation in the maintenance of the ISC pool even in homeostatic conditions, consistent with recent reports in mouse models[10,11]

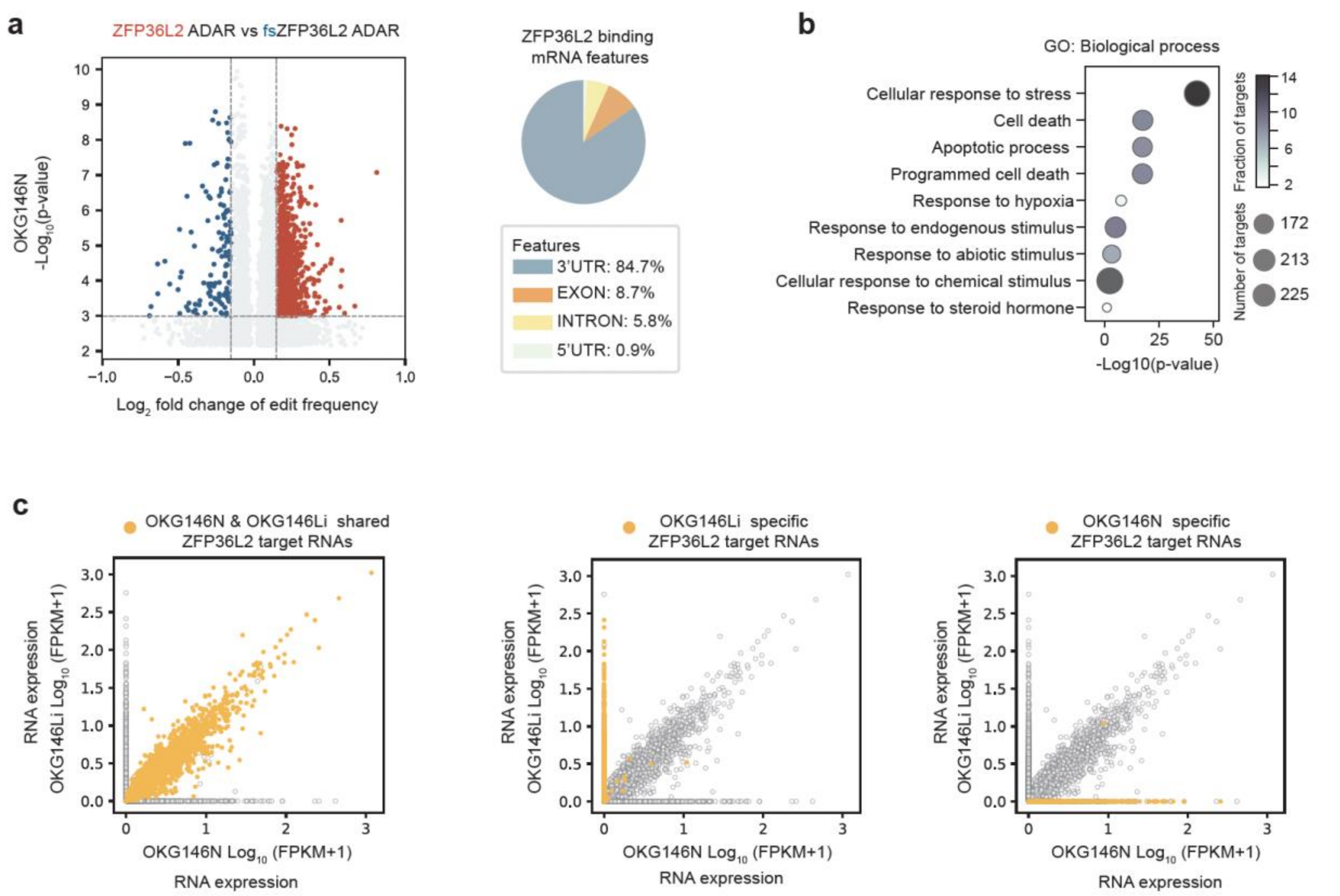

Fig. R3 (Extended Data Fig. 10b-d) | HyperTRIBE profiling of ZFP36L2-bound mRNAs in human normal colon epithelial organoids. a, Volcano plot showing ZFP36L2-bound mRNAs identified by ZFP36L2-ADAR-mediated RNA editing in OKG146N organoids, comparing wild-type ZFP36L2 with frameshift mutant ZFP36L2 (fsZFP36L2). Pie chart showing the genomic distribution of ZFP36L2-specific ADAR editing sites, defined as sites with $-\log_{10}(P\text{-value}) > 3$and $\log_2$fold change in editing frequency > 0.1(wild-type versus fsZFP36L2). **b,** Gene Ontology (GO) enrichment analysis of shared ZFP36L2-bound mRNAs identified in OKG146N, OKG146P and OKG146Li organoids (Revision Table 1b), using GO Biological Process gene sets. The dot plot shows representative GO terms from Fig. 5d. The x axis indicates the $-\log_{10}(P\text{-value})$of enrichment, dot size indicates the number of target genes in each pathway, and

colour indicates the fraction of genes enriched in each term. **c,** Scatter plots showing RNA expression in OKG146N and OKG146Li organoids, plotted as $\log_{10}(\mathrm{FPKM}+1)$. Shared ZFP36L2-bound RNAs, as well as OKG146Li-specific and OKG146N-specific ZFP36L2 targets, are highlighted in yellow.

R1.6. Figure 2c Injury-induced expression appears to start in the differentiated cells at the top of the crypts (day 2 to 7 Fig2c and then appear in the TA at day 10. Can the authors comment on this?

As we showed in the original submission, we observed that "during the regeneration phase, Zfp36l2 was re-expressed first in the crypt middle (TA compartment) and crypt base (ISC compartment) by day 10, preceding restoration of crypt base *Lgr5* expression by day 14." This is consistent with prior observations from multiple labs that a variety of differentiated cell types can serve as cells of origin for injury-induced dedifferentiation into crypt base Lgr5+ ISC cells and eventual restoration of homeostatic differentiation[3,12–26]. Our observation that Zfp36L2 is expressed in a broad swathe of TA and more differentiated cells in the mid-upper crypt regions suggests that Zfp36L2 could function in any of these cells to trigger stress-induced dedifferentiation into the Lgr5+ ISC state. We did observe some non-specific staining at the crypt top, associated with likely residual mucus that can trap antibody, and do not include this in our quantifications which are based on DAPI+ nuclei. We have replaced the Figure panel from day 2 for increased clarity (**Fig. 2c**). We now include larger sections of the tissue (**Supplementary Data 1**) and the entire colon swiss rolls in (**Supplementary Data 3)** for further clarity.

c

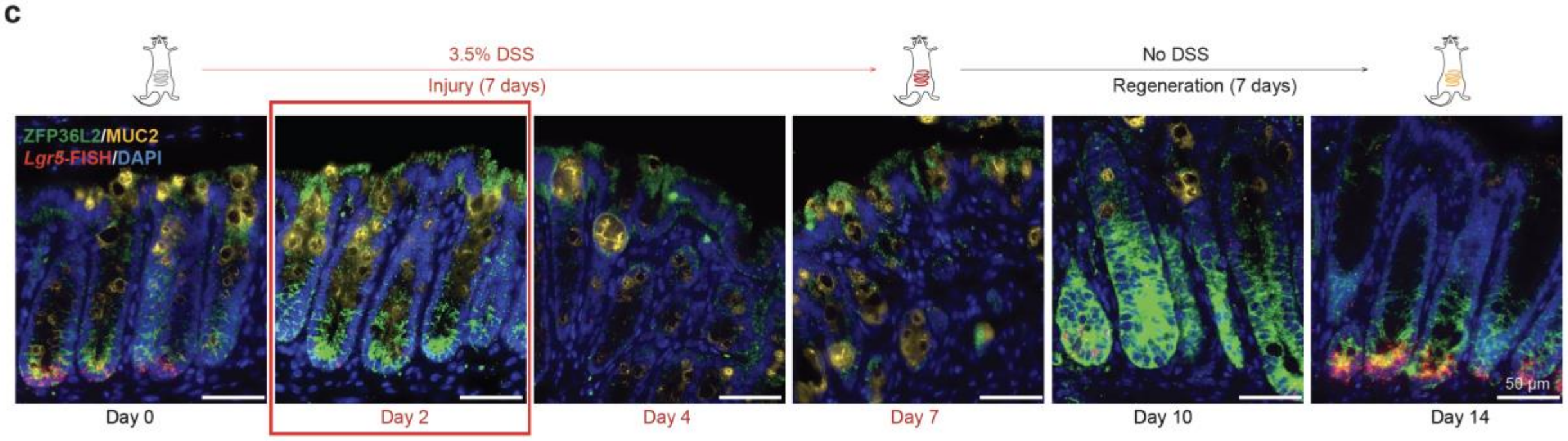

Fig. 2c. Representative images of *Lgr5*-FISH, ZFP36L2-IF, and MUC2-IF in colon tissue harvested at the indicated timepoints from mice provided 3.5% dextran sodium sulfate (DSS) in drinking water for 7 days followed by recovery for 7 days with DSS-free water. n=3 mice per time point.

R1.7. Line 129 Should be IF and RNA FISH

Corrected as suggested (revised manuscript, line 128).

R1.8. Line 133 Figure 2e Is there any change in the Goblet cell population?

To address this question, we have now quantified the goblet cell population by MUC2 staining in $Zfp36L2^{WT}$ and $Zfp36l2^{IEC}$ knockout mice, and did not detect a statistically significant difference between groups. We have added the quantification and corresponding statistical analysis to the revised manuscript (**Extended Data Fig. 3f**).

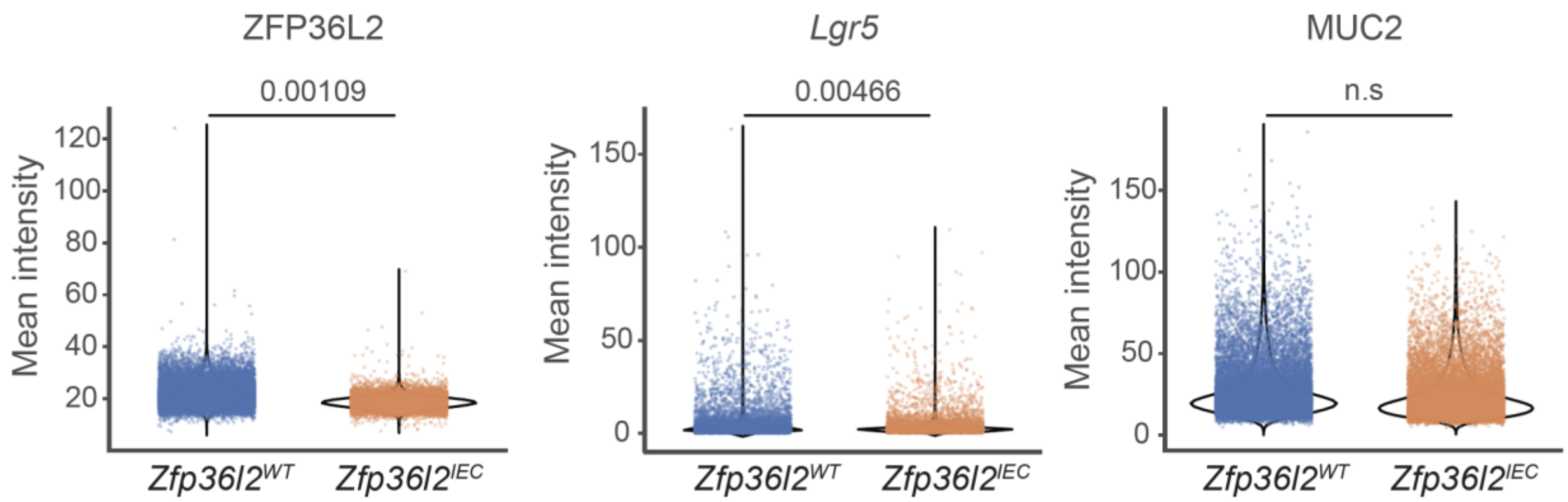

Extended Data Fig. 3f | Quantification of ZFP36L2, Lgr5 and MUC2 expression in mouse colonic crypts. Quantification of ZFP36L2 and MUC2 mean fluorescence intensity by immunofluorescence, and Lgr5 RNA abundance by RNA FISH, in colonic crypt cells from Swiss rolls of *Zfp36l2*^{WT} and *Zfp36l2*^{IEC} mice. Violin plots with individual cells overlaid as dots are shown. Each dot represents one cell. Swiss roll regions containing U-shaped crypts were quantified from 3 mice per group. Two-tailed Mann–Whitney *U* test. Data correspond to the representative images in **Extended Data Fig. 3e and Supplementary Data 2**.

R1.9. Line 149-150 Regarding assessment of de-differentiation capacity, did the authors verify that the differentiated organoids lacked LGR5+ cells?

We thank the reviewer for raising this important point. To directly verify depletion of LGR5+ cells under the differentiation conditions used throughout this study (3 days of organoid culture in Wnt/R-Spondin containing media, followed by Wnt-R-spondin withdrawal for 3 days), we repeated the differentiation protocol using *Lgr5*–DTR-GFP reporter organoids and quantified *Lgr5*-GFP+ cells by flow cytometry after 3 days of culture in differentiation media (**Extended Data Fig. 4h,i**).. We observed that differentiated organoids contained negligible *Lgr5*–GFP+ cells (1.4% vs original 13.3%), confirming effective loss of the LGR5+ ISC population prior to the de-differentiation assays. We have included these important control experiments in the revised manuscript.

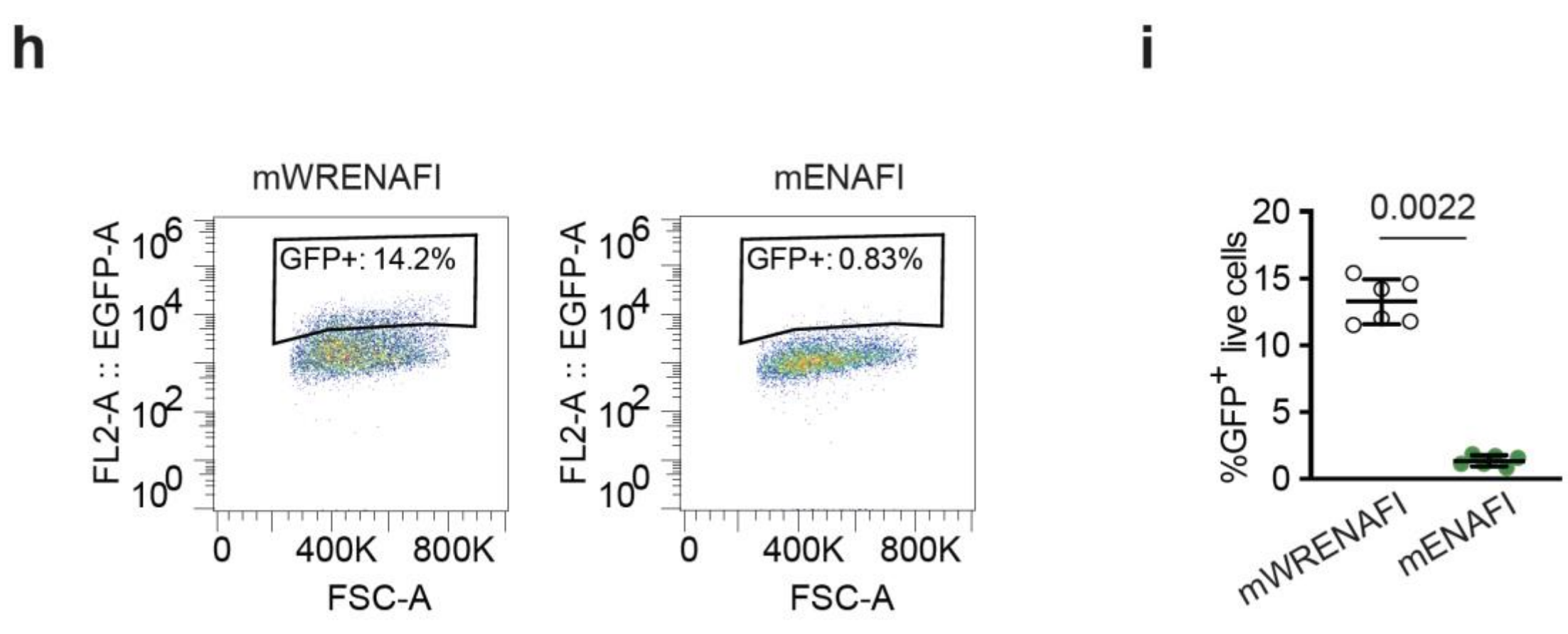

Extended Data fig. 4h,i: **LGR5-positive cells are absent following Wnt and R-spondin withdrawal. h**, Representative flow cytometry density plots showing the frequency of Lgr5-GFP+ cells after 5 days of culture in mWRENAFI (left), or after 3 days in mWRENAFI followed by 2 days in mENAFI medium lacking Wnt and R-spondin (right). **i**, Quantification of *Lgr5*-GFP+ cells from (**h**). n = 2 mice; 3 technical replicates per mouse. Data are mean ± s.d. Two-tailed Mann–Whitney *U* test.

R1.10. Line 184 “O” should be added to KG

Corrected as suggested (revised manuscript, line 216).

R1.11. Line 191 Bioluminescence Imaging (BLI)

Corrected as suggested (revised manuscript, line 220).

R1.12. Figure 2e The data would be more convincing if quantitative data on the cell types present could be provided.

We thank the Reviewer for this feedback. Accordingly, we have now added quantitative analyses of the relevant epithelial cell types (**Extended Data Fig. 3f**). Specifically, we quantified goblet cells by MUC2 IF, ZFP36L2 by IF and ISCs by *mLgr5* RNA FISH in *Zfp36L2*^{WT} and *Zfp36l2*^{IEC} knockout mice. ZFP36L2+ cells and *Lgr5*+ ISCs were significantly reduced in the *Zfp36l2*^{IEC} knockout mice, while we did not detect a statistically significant difference in the MUC2+ goblet cell population.

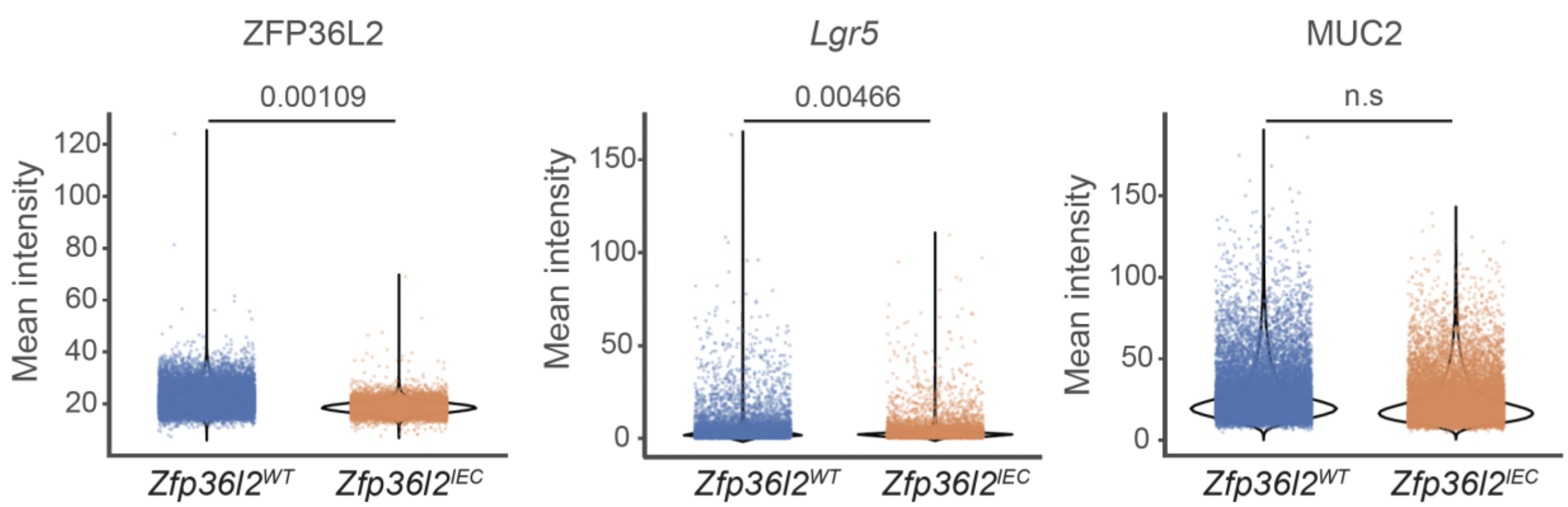

Extended Data Fig. 3f | Quantification of ZFP36L2, Lgr5 and MUC2 expression in mouse colonic crypts. a, Quantification of ZFP36L2 and MUC2 mean fluorescence intensity by immunofluorescence, and Lgr5 RNA abundance by RNA FISH, in colonic crypt cells from Swiss rolls of *Zfp36l2*^{WT} and *Zfp36l2*^{IEC} mice. Violin plots with individual cells overlaid as dots are shown. Each dot represents one cell. Swiss roll regions containing U-shaped crypts were quantified from 3 mice per group. Two-tailed Mann–Whitney *U* test. Data correspond to the representative images in **Extended Data Fig. 3c**.

R1.13. Figure 2f Error bars should be included on the graph

We thank the Reviewer for this suggestion. Error bars were previously displayed as a shaded region with dashed boundaries; to improve clarity, we have now made the error bars more explicit and clarified the legend (**Fig. 2d**).

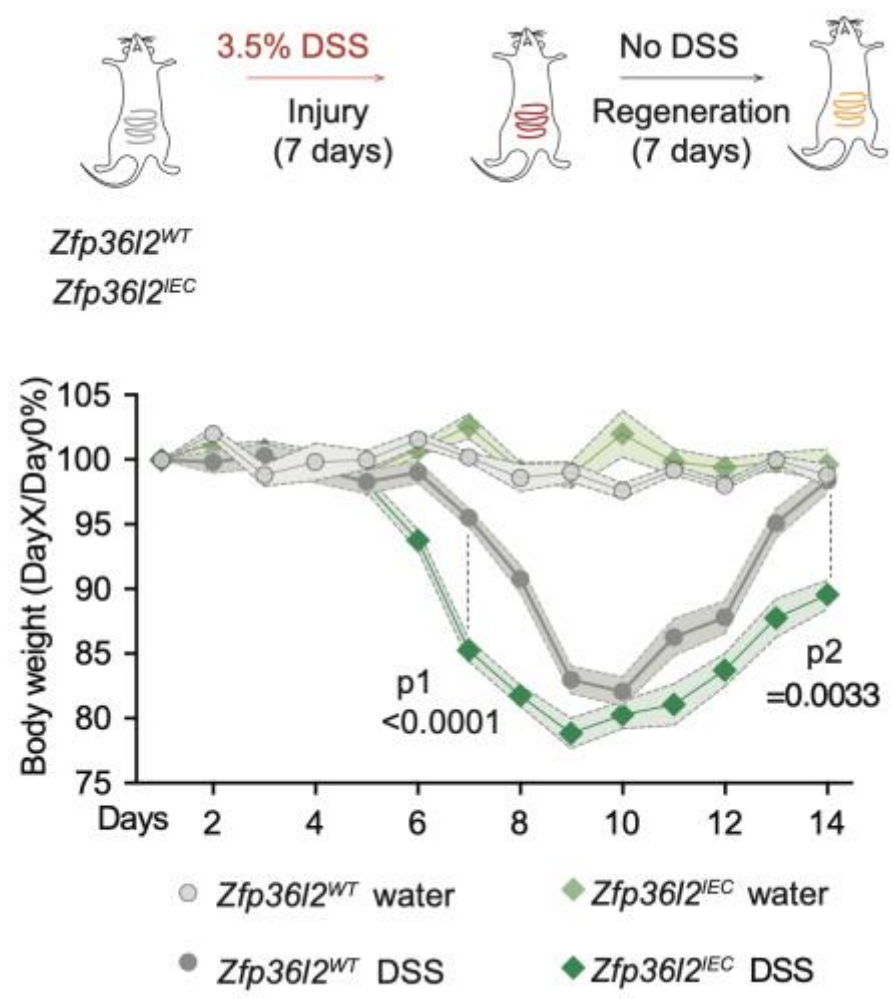

Fig. 2d | (Top) Schematic of experimental design of DSS-induced colitis and regeneration. *Zfp36l2*^{WT} and *Zfp36l2*^{IEC} mice were provided with 3.5% DSS in drinking water for 7 days followed by recovery for 7 days with DSS-free water. (Bottom) Daily body weight measurements throughout the experiment. n=5 mice for water controls; n=15 mice for DSS-treated groups. Data are shown as mean (solid line; dots denote individual time points) ± s.e.m. (shaded area; dashed lines indicate the s.e.m. boundaries); two-tailed Mann-Whitney *U* tests at day 7 and day 14.

R1.14. Figure 2g, j, k Error bars are not clearly visible even though they are defined in the legend

Corrected as suggested. (Revised **Fig. 2e; Extended Fig. 4k,l**).

R1.15. Figure 3C, H Error bars are not visible, 3C include the organoid identity.

Corrected as suggested (Revised **Fig. 3c,h**).

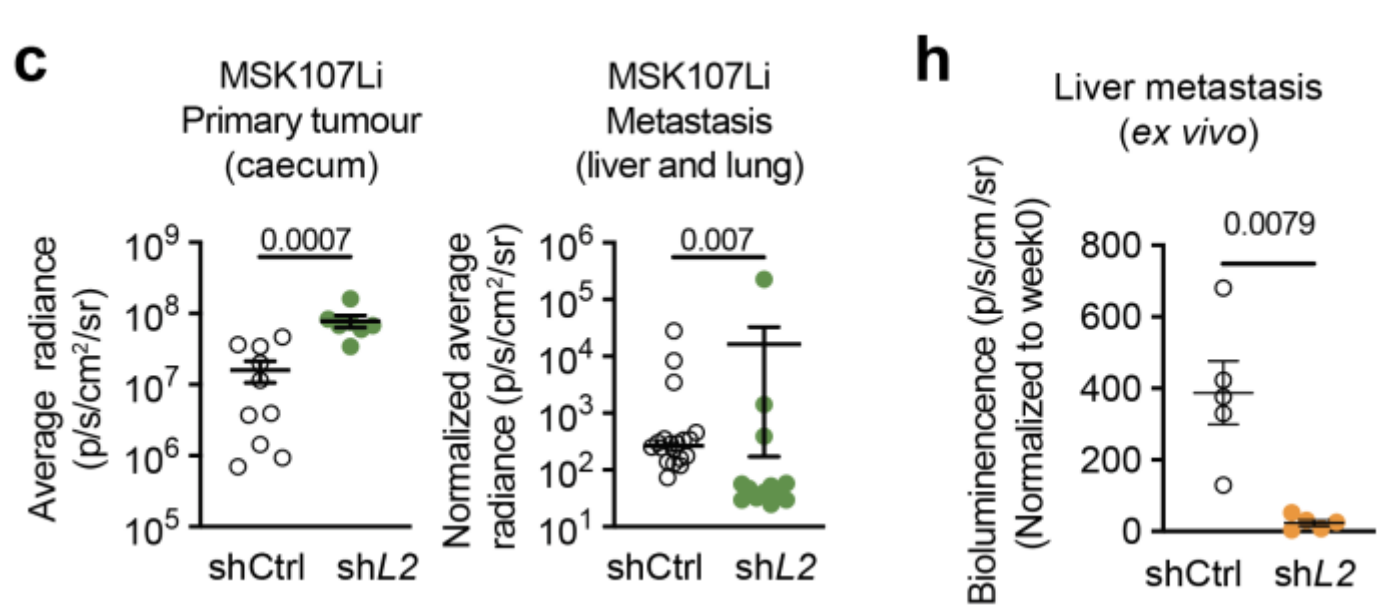

R1.16. Figure 3K The schematic diagram is not easy to interpret and could be made clearer.

We thank the reviewer for this feedback, and have accordingly replaced **Fig. 3k** with a redesigned schematic that more clearly depicts the workflow/model and improves readability.

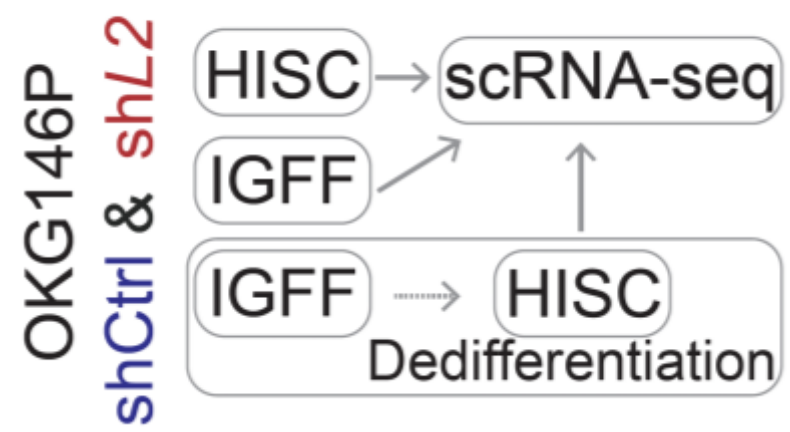

Fig. 3k | Schematic of CRC organoid dedifferentiation experimental design. Organoids were cultured in human intestinal stem cell (HISC) media for 7 days, and live cells were collected and sorted for single-cell RNA sequencing (scRNA-seq) using the 10x Genomics platform. A portion of the same batch of organoids was passaged into intestinal growth factor-free (IGFF) medium for 7 days to induce differentiation as shown in Moorman *et al.*[1] and then passaged back into HISC media for another 7 days to induce dedifferentiation into an ISC-like state, followed by scRNA-seq.

R1.17. Line 192 Further explanation of 3i should be included.

We appreciate the Reviewer's comment seeking to increase the clarity of our description of this critical experiment. Accordingly, we have expanded the text to clarify the early post-inoculation kinetics in **Fig. 3i** (progressive BLI decline in shZFP36L2 during the first ~2 weeks, followed by outgrowth of the surviving/seeded population with growth kinetics comparable to controls), supporting an effect on early metastatic seeding/survival. This explanation has been added to the revised manuscript (Page 7, lines 210-218).

> Notably, the first two weeks following intrasplenic injection are a period of high attrition—disseminating cancer cells extravasating from liver sinusoids and infiltrating into the hepatic parenchyma must overcome immune, biochemical, and growth factor stressors to survive, with only a small fraction of injected cells overcoming these hurdles to reinitiate tumour growth[27]. During this early seeding period, shCtrl organoids displayed a slow but steady increase in BLI signal. In contrast, sh*ZFP36L2* organoids showed a sustained decrease in BLI signal from immediately after injection through week 2, consistent with a profound defect in early metastatic seeding and reinitiation of growth in the liver. Beginning around week 2, the BLI signal in the sh*ZFP36L2* cohort increased, suggesting that the rare cells that successfully seeded were able to subsequently regrow with kinetics similar to controls, ultimately giving rise to macroscopic tumour nodules (**Fig. 3i**).

R1.18. Figure 4F Error bars should be visible

We have made the error bars clearly visible as suggested (Revised **Fig. 4g, Extended Data Fig. 8d,f**).

Reviewer 2

Many observations over the past decade or so advance the idea that colorectal cancer (CRC) cells are “plastic” in nature, mimicking physiologic cycles of differentiation and dedifferentiation in normal intestinal stem cells (ISCs). Among associated molecular processes, activation of a YAP/TAZ signal-related transcriptional program is seen both during tissue regeneration after ISC injury and in intestinal tumors. Several groups have recognized and studied this “oncofetal” program during intestinal tumor initiation, progression, and metastasis. However, its function and significance remain unclear and the literature on the topic is often imprecise. The present study “sought to identify the molecular switch controlling stress-induced dedifferentiation into an LGR5+ ISC state, a conserved process that is essential for both epithelial regeneration and the seeding and outgrowth of canonical metastases” (lines 73-75). Here, “canonical” distinguishes colonocytic from squamous or neuroendocrine “non-canonical” differentiation, as defined in the group’s recent study of biospecimen trios from normal colon and primary and metastatic CRCs. The authors identify ZFP36L2 as a gene enriched in “tumor ISC-like cells”, mutated in 5-10% of CRCs, and ectopically expressed and required during mouse crypt epithelial regeneration (Figs. 1 and 2). Loss of ZFP36L2 in organoids derived from metastatic CRCs impairs their ability to seed liver deposits from distant graft sites but does not affect growth of those metastases, and RNA expression in ZFP36L2-deficient CRC organoids is interpreted to reflect excessive “non-canonical” differentiation (Fig. 3). immunofluorescence shows that ZFP36L2, which has multiple intrinsically disordered regions (IDRs), forms phase-separated RNA-dependent cytoplasmic condensates, more so in single isolated cells than in organoids (Fig. 4). Like other such factors, ZFP36L2 is associated with mRNA decay, but decayed transcripts were unrelated to colonic differentiation, so the authors propose additional unrelated functions and found that transcription factors represented ~40% of co-immunoprecipitated proteins. [redacted]

The manuscript is clearly written and thoughtfully assembled. The problem is that it tackles the entire gamut of possible ZFP36L2 activity in cells without solidifying any of at least half a dozen disparate conclusions. Many of these represent promising leads that would elicit enthusiasm if they were individually well proven and together generated a coherent model. Instead, the reader is left with a large number of dots that don’t connect into a plausible arc and don’t fulfill the goal to identify the molecular switch controlling stress-induced dedifferentiation. The large volume of data doesn’t support the conclusion that ZFP36L2 is that elusive molecular switch.

In essence, this manuscript has two separate components that don’t join in a conclusive or satisfying way. The first piece (Figs. 1-3) needs stronger evidence to implicate ZFP36L2 in epithelial regeneration or “stress-induced cancer cell dedifferentiation.” The second piece (Figs. 4-5) needs stronger experimental support [redacted] and deeper insights into what the disparate findings might mean. Finally, to support the authors’ claims (which are somewhat murky), the two components would need to be connected with greater rigor and plausibility.

We thank the reviewer for their thoughtful feedback and address their specific comments below:

Major comments (corresponding roughly to numbered Figures)

R2.1. ZFP36L2 expression, "tumor ISC-like" cells, and link to AP-1 factors. ZFP36L2 seems to be expressed in many crypt epithelial (except the most mature) and almost all tumor cells. It is not ISC-restricted (Figs. 1b, 1e, Extended Data Fig. 1b-c, Fig. 2a-b) and expression in "tumor ISC-like" cells is lower than in "normal" ISC or TA cells (Fig. 1b), though Extended Data Figs. 1a and 1d (difficult to understand) mask this point. How are tumor "ISC-like cells" defined? A "tumor ISC-like" signature is about equally auto-correlated with ZFP36L2 in normal (with a better FDR) than in CRC epithelium (Fig. 1i, Extended Data Fig. 1g). ZFP36L2 expression might seem even less selective if "tumor ISC-like" cells were defined differently. Tumor cells in the top and bottom deciles of ZFP36L2 expression are almost equal in their "CSC signatures" (Extended Data Fig. 2). What does it mean, then, to note that "ZFP36L2 is the second most mutated tumor ISC-associated gene"?

R2.1.1: We thank the reviewer for prompting us to clarify these points. The "Tumour ISC-like" cells were defined transcriptionally based on enrichment of an ISC-related gene program derived from tumor-intrinsic gene modules, rather than by single markers or presumed functional equivalence to normal intestinal stem cells. Specifically, in Moorman et al., *Nature*, 2025, we used an unbiased computational approach (Hotspot) to identify modules of co-regulated gene expression recurrently expressed across scRNAseq data from human primary and metastatic CRC epithelial cells from 31 patients[1]. One of these tumour-derived covariant gene modules showed strong correspondence to well-characterized normal intestinal stem and transit-amplifying programs relative to differentiated epithelial lineages, and we therefore termed them "tumour ISC-like", since they are ISC-like, but derived from the tumour. We prioritized human CRC–derived signatures because tumor-intrinsic covariant gene modules capture stem-like transcriptional states specific to human tumors, which are not fully conserved in mouse small intestine, which has historically been the main data source for many ISC-related gene programs and functional studies in the field. As the Reviewer points out and as we show extensively throughout this manuscript, by definition the "tumor ISC-like" program shares many features, including ZFP36L2 expression, with normal colon ISCs. For clarity and consistency, we retained the "tumor ISC-like" nomenclature in this manuscript. We appreciate that this nomenclature could cause confusion, and have now clarified this in the text (Page 3, Line 79).

> …we first focused on the set of genes co-expressed in ISC-like cells in a cohort of same-patient trios of normal, primary and metastatic CRC, identified through Hotspot gene module analysis of fresh tissue tumour single-cell RNAseq (scRNA-seq; termed "tumour ISC-like")[14]

R2.1.2 : Regarding the expression of ZFP36L2 in cells based on their "CSC signatures", several recent papers in the field have adopted different gene sets to define what are phenotypically largely consistent populations. Our aim was to show the consistency of the enrichment of ZFP36L2 in ISC-like cells and in a subset of cells expressing regenerative stem cell programs, regardless of defining gene sets. As described in the legend, the gene signatures employed in **Extended Data Fig. 2** are derived from prior work by Tape and colleagues (Qin et.al., Cell, 2023), who demonstrated that primary CRC tumors comprise three distinct populations, including a proliferative CSC population (proCSC) enriched in primary CRC, a regenerative or revival CSC (revCSC) population similar to the regenerative injury-repair and fetal states identified in Moorman et al., and a third undesignated CSC population (CSC) that lacked the distinguishing markers of

either the proCSC or revCSC populations, which was also enriched in tumours (although to a lesser extent than proCSCs), but was the dominant CSC population in WT colon epithelia[28]. Consistent with this framework, ZFP36L2 expression is selectively elevated in revCSCs, which are associated with injury repair (**Extended Data Fig. 2b**, bottom panel) and cellular plasticity, and in and the tumor enriched proCSCs and is more uniformly enriched in homeostatic CSC populations.

We agree with the reviewer that ZFP36L2 expression is not restricted to a single discrete cell state. As shown in **Fig. 1**, ZFP36L2 displays a graded expression pattern across epithelial lineages, with highest expression in ISC-like cells and transit-amplifying (TA) populations in normal tissue and revCSCs in tumor cells. Our central argument is therefore not that ZFP36L2 marks a static ISC or CSC state, but that it functions in transitional, injury-associated populations that facilitate switching from differentiated epithelial states, through a revCSC intermediate, toward an ISC-like program. This interpretation is verified and fleshed out in subsequent figures demonstrating preferential association of ZFP36L2 with regenerative and injury-linked transcriptional programs. Overall, the purpose of this introductory section is to survey the genetic and transcriptional association of ZFP36L2 mutations and expression with known CRC and ISC biology, with the expression of ZFP36L2 in the ISC and RevCSC populations motivating the subsequent in-depth functional and mechanistic studies on the role of ZFP36L2 in this transitioning cell population in both regenerating epithelia and in tumor regeneration.

R2.2. If ZFP36L2 is relatively enriched in stem and TA cells, then its association with an "AP-1 gene score" (Fig. 1j) is expected and may be trivial.

We respectfully disagree that the association between ZFP36L2 expression and AP-1 gene scores is expected or trivial based solely on enrichment of ZFP36L2 in stem or transit-amplifying (TA) compartments. In colorectal cancer, ISC-like identity is not intrinsically coupled to AP-1 activity. Recent single cell multiomic analyses in human CRC (Ogden et al., *Cell Genomics*, 2025) demonstrate that canonical stem/TA and stem-NOTUM states are predominantly regulated by WNT-associated transcriptional programs and display minimal AP-1 activation. In contrast, AP-1 activity is selectively enriched in regenerative and inflammatory tumor cell states that are distinct from stem/TA cells[29]. These findings suggest that AP-1 activation reflects injury-associated plasticity rather than stemness. Consistently, another study (Mzoughi et al., *Nature Genetics*, 2025) also recently identified distinct Wnt-driven Lgr5+ CSC, and AP-1/YAP-driven oncofetal programs in mouse models of CRC, and found that the AP-1 state, but not the CSC state, was required for tumor regeneration after FOLFIRI chemotherapy[30]. Consistent with this framework, our data show that ZFP36L2 associates with AP-1 gene scores in a subset of tumor cells in a regenerative or transitional state, rather than uniformly across all ISC-like or TA cells. Thus, the observed ZFP36L2–AP-1 association is not a trivial consequence of stem cell identity, but instead reflects engagement of an injury-linked transcriptional program that is distinct from canonical ISC/TA states. Importantly, the dual association of ZFP36L2 with both ISC-related programs and AP-1–linked regenerative signatures emerged from unsupervised analyses, rather than from an *a priori* assumption of pathway coupling. Cells with high ZFP36L2 expression show strong enrichment of revCSC–like programs[28], consistent with a transitional, injury-responsive state[28,31]. It was this convergence—association with ISC programs, selective coupling to AP-1 activity, and

enrichment in revCSCs/injury repair states—that motivated our hypothesis that ZFP36L2 functions during injury-induced dedifferentiation rather than in steady-state ISCs.

R2.3. The "revCSC signature" is amplified in nearly all ZFP36L2-high cells, but the "injury repair signature", L1CAM, and EMP1 are elevated in small fractions. Are the latter 3 elements increased in overlapping or distinct cells, and what is the significance? Most important, how do the authors reconcile widely elevated AP-1 gene scores (Fig. 1j) with the small fraction of "revCSC" and "injury repair" expressing cells seen only within the ZFP36L2-high decile.

We appreciate this important question regarding the relationship between revCSC programs, injury-repair markers, and AP-1 activity within ZFP36L2-high cells. The injury repair signature, L1CAM, and EMP1 are not independent elements, but are strongly correlated and have been shown to co-occur within regenerative tumor cell populations, as demonstrated in Moorman et al., *Nature*, 2025 and reproduced below (**Fig. R6**) for reference. L1CAM and EMP1 and components of and individual markers of the injury repair population and have been shown in independent studies to mark a tumor regenerative, metastasis-initiating cell state[32,33]. We now find that cancer cells highly expressing the Injury Repair signature (and its components L1CAM and EMP1) are also highly expressing ZFP36L2 (**Extended Data Fig. 2**), even though ZFP36L2 is more highly expressed overall in the Tumor ISC-like population (**Extended Data Fig. 1D**). These data suggest that ZFP36L2 might mark a subset of Injury Repair state cels that are transitioning into an ISC-like state.

While mechanistically addressing the relationship between AP-1 transcription factor activity and ZFP36L2 is not the central focus of this study, we interpret these signatures in a dynamic rather than static framework. Our model is as follows: ZFP36L2 expression spans transitional epithelial states, in a subset of cells expressing the injury-repair program associated with AP-1 activation and tumor regeneration. A subset of these cells subsequently downregulates injury-repair markers as they dedifferentiate toward an ISC-like state and re-establish homeostasis. As a consequence, AP-1 gene scores can be elevated across a broader population of ZFP36L2-high cells, whereas overt injury-repair markers (including L1CAM and EMP1) are confined to a narrower temporal window within this trajectory. Thus, we speculate that the widespread AP-1 activity and the smaller fraction of injury-repair or revCSC-expressing cells reflects the temporal dynamics of epithelial regeneration rather than mutually exclusive cell states. ZFP36L2 and AP-1 span these transitions, consistent with a model in which ZFP36L2 marks injury-induced dedifferentiation and regenerative plasticity rather than a single fixed cell identity.

Critically, we are cautious not to draw broad conclusions from these observations – as elaborated below, we simply report our observation of the association of ZFP36L2 expression with AP-1, L1CAM/EMP1-expressing injury repair states and revCSC signatures as overlapping programs established through multiple independent studies to be associated with injury induced transitional states required for tumor regeneration[1,26,28–30,32,33]. The association of ZFP36L2 expression with both these regenerative states and the Lgr5+ CSC state implicates ZFP36L2 as a putative mediator of the transition between these states. It is this hypothesis that we examine in great detail using multiple *in vivo* and organoid-based models of epithelial regeneration and metastasis in the remainder of this manuscript.

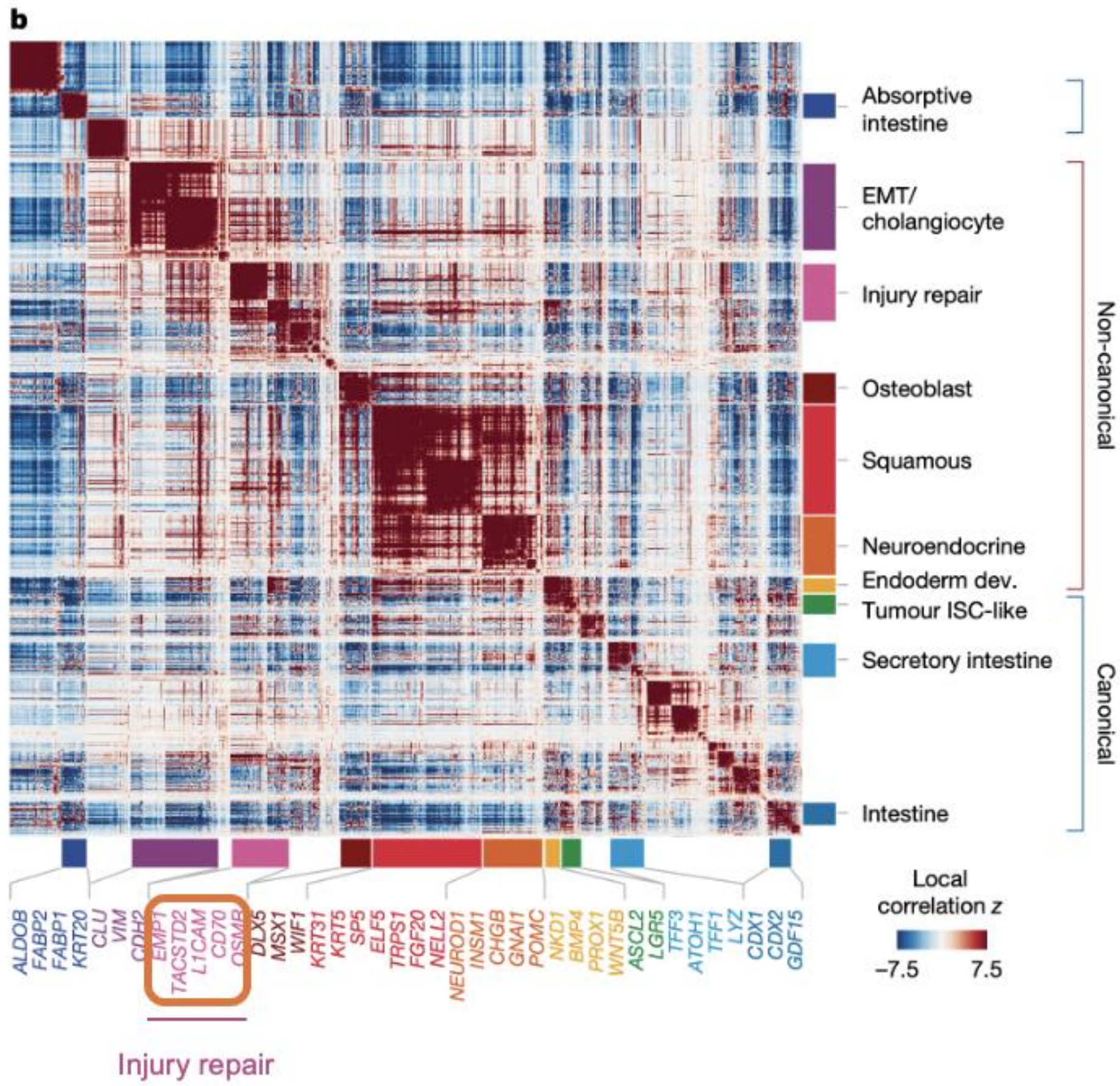

Fig. R4 | *L1CAM* and *EMP1* are co-expressed as part of an Injury Repair module of covarying genes in human CRC. Figure reproduced from Moorman et al., Nature, 2025[1]. Hotspot modules in all CRC tumour cells. The heat map comprises 2,003 highly variable genes with significant autocorrelation (FDR < 0.01), grouped into 4 canonical and 6 non-canonical CRC-derived gene modules. Across CRC cells, EMP1 and L1CAM are co-expressed in the same covarying group of genes as part of the Injury Repair module.

R2.4. In summary, Fig. 1 and Extended Data Figs. 1 and 2 seem intended to defend interest in ZFP36L2 and build a connection to AP-1 complexes, but the data are murky and don't support the implied conclusions. Broad expression in primary tumors and apparent diminution in metastases doesn't shed useful light (Fig. 1e – how many samples were examined? how representative are the areas shown?) and statements like "Although ZFP36L2 was most broadly expressed in tumour ISC-like cells, …." (line 96) add to the confusion. Mutation in 5-10% of CRCs is arguably sufficient justification to study ZFP36L2 functions. Instead of laying the foundation for a potentially interesting study, the convoluted implications of Fig. 1 and Extended Data Figs. 1 and 2 muddy the waters. The leap from ZFP36L2 to AP-1 is tenuous.

We appreciate the reviewer's careful reading and would like to clarify the intent and interpretation of **Fig. 1** and **Extended Data Figs. 1** and **2**. These figures were not designed to establish mechanistic conclusions, but rather to elaborate the hypotheses that motivated the extensive and rigorous functional analyses presented later in the manuscript. As stated in the manuscript, our initial observation was that ZFP36L2 expression is strongly associated with an ISC-related gene

program in human CRC epithelium. We then performed unbiased, genome-wide correlation analyses to identify transcriptional programs covarying with ZFP36L2 expression, which revealed enrichment of AP-1–associated genes as the top genes co-varying with ZFP36L2. Given the extensive literature linking AP-1 activity to injury response and epithelial regeneration, we considered the convergence of ZFP36L2 with both ISC-related and AP-1–linked programs to be biologically intriguing. Importantly, we do not draw functional conclusions from these correlative analyses; instead, they serve as the basis for hypotheses that are subsequently tested using multiple in vivo and organoid-based regeneration models. Regarding ZFP36L2 expression in primary tumors and metastases, our quantitative analyses are based on single-cell RNA-seq data from 25 paired primary and metastatic CRC samples. These data show that metastatic tumors can lose ZFP36L2 expression and that ZFP36L2-low cells are also depleted of ISC-related signatures (**Extended Data Fig. 1a**). Patient tissue staining further validates that the co-localization of ZFP36L2 with ISC markers such as LGR5 is observable at the spatial level and is not an artifact of scRNA-seq analysis. While mutation frequency alone justifies studying ZFP36L2 in CRC, our analysis of co-expression of ZFP36L2 with distinct cell state-defining gene expression programs places ZFP36L2 within a functional framework relevant to epithelial plasticity and regeneration. **Fig. 1** and **Extended Data Figs. 1** and **2** thus provide the conceptual foundation for the extensive functional validation that follows.

R2.5a Technical problems with experiments on ZFP36L2 requirement in intestinal regeneration. (a) DSS colitis occurs in patches. To establish a "spatiotemporal sequence" of ZFP36L2 expression in this setting (Fig. 2c), the authors should demonstrate that the inferred changes occur in affected areas (the first 2 images are not so different from the control in Fig. 2b). Ideally, differences between affected and unaffected regions should be assessed/quantified in tissue "Swiss rolls". If ZFP36L2's role is in regeneration, can the authors explain why weight loss is more severe during the injury phase? If mutant epithelium is more sensitive to DSS damage, might recovery be delayed simply as a proportional effect? What happened to mutant body weight after 14 days? Did 10-15 mutant mice lose exactly the same fraction of body weight between 5 and 8 days (these are the only data points lacking a range)?

We thank the Reviewer for this thoughtful feedback and agree that DSS colitis can occur in a patchy manner depending on experimental conditions. Below we clarify our experimental observations, spatial analyses, and interpretation of ZFP36L2 function during injury and regeneration.

1. Spatial pattern of injury and ZFP36L2 expression in our DSS model: In our experiments, we did not observe a patchy pattern of injury in the distal colon. Under the DSS conditions used in this study, the distal colon uniformly exhibited loss of *Lgr5* signal, disruption of crypt architecture, followed by coordinated crypt regeneration. Consistent with this, the changes in ZFP36L2 expression shown in Fig. 2c reflect a global response across the distal colon rather than focal lesions. To address the reviewer's concern regarding spatial representation, we have now provided: a. Larger field-of-view images corresponding to Fig. 2c (**Supplementary Data 1**), and b) Whole Swiss-roll sections of the distal colon (**Supplementary Data 3**), which confirm that the temporal changes in ZFP36L2 expression occur broadly across affected regions.

2. Interpretation of the early time points (first two images in **Fig. 2c**): We agree that the first two images in Fig. 2c appear similar to control. This is expected, as these mice had been exposed to

DSS water for only 2 days, a time point at which overt crypt architectural disruption has not yet occurred. Importantly, however, *Lgr5* signal is already markedly lost at this early stage, despite preserved crypt morphology. This dissociation suggests that stem cell identity is disrupted prior to gross tissue damage, consistent with a stress-responsive phase preceding visible colitis.

3. Spatiotemporal relationship between ZFP36L2 induction and regeneration: As shown in Fig. 2c: At day 2 of DSS exposure, *Lgr5* expression is lost, coinciding with early induction of ZFP36L2. During the regeneration phase (day 10), Zfp36l2 expression is strikingly elevated, concomitant with restoration of *Lgr5*-positive cells and crypt structure. With continued recovery, ZFP36L2 expression returns to its baseline homeostatic pattern, paralleling tissue repair. Based on this temporal sequence, we interpret ZFP36L2 induction as part of a stress-termination and regenerative buffering response, rather than a marker of injury severity per se. Failure to properly resolve stress responses may therefore contribute to impaired survival.

4. Clarifying the relationship between injury severity, weight loss, and regeneration: During DSS injury, ongoing epithelial destruction is countered by attempts at regeneration, and the weight loss during days 1-7 while the mice are on DSS reflects a complex balance of epithelial injury and regeneration, while the weight recovery on days days 8-14 reflects regeneration in the absence of ongoing injury. The Reviewer raises the important possibility that increased weight loss in mutant mice could reflect heightened DSS sensitivity rather than a defect in regeneration. In our original manuscript, we further clarified the role of *Zfp36l2* in dedifferentiation using an *ex vivo* organoid regeneration assay (**Extended Data Fig. 4g-l**).To more definitively disentangle injury from regeneration *in vivo*, based on the Reviewer's feedback, we have now employed an orthogonal, injury-independent mouse ISC regeneration model: Using **Lgr5-DTR**–mediated ablation of Lgr5+ ISCs in $Zfp36l2^{WT}$ and $Zfp36l2^{IEC}$ knockout mice (**Revision Data 2**), we find that *Zfp36l2*-deficient epithelium exhibited impaired restoration of Lgr5-positive cells, both *in vivo* and in organoid assays. These data support a direct role for *Zfp36l2* in regeneration, independent of DSS-induced epithelial toxicity.

5. Body weight beyond day 14: We did not extend body-weight analysis beyond day 14, as body weight at later stages is not a sensitive readout of epithelial regeneration. The colon can eventually regain barrier integrity through multiple mechanisms, including: a) dedifferentiation-driven ISC regeneration, and b) expansion, fission, and migration of adjacent intact crypts[34,35]. Thus, given sufficient recovery time, body weight may normalize even when early regenerative efficiency is compromised. Our focus therefore remained on early regenerative dynamics and stem cell restoration, which are more directly linked to ZFP36L2 function.

6. Clarification of body-weight error bars (days 5–8): We apologize for the confusion regarding the apparent lack of variability at days 5 and 8. The error bars were present but extremely narrow, resulting in limited visibility. In the revised figure, we have enhanced error-bar contrast, and displayed them using dashed lines to clearly indicate variability across animals (**Fig.2d**).

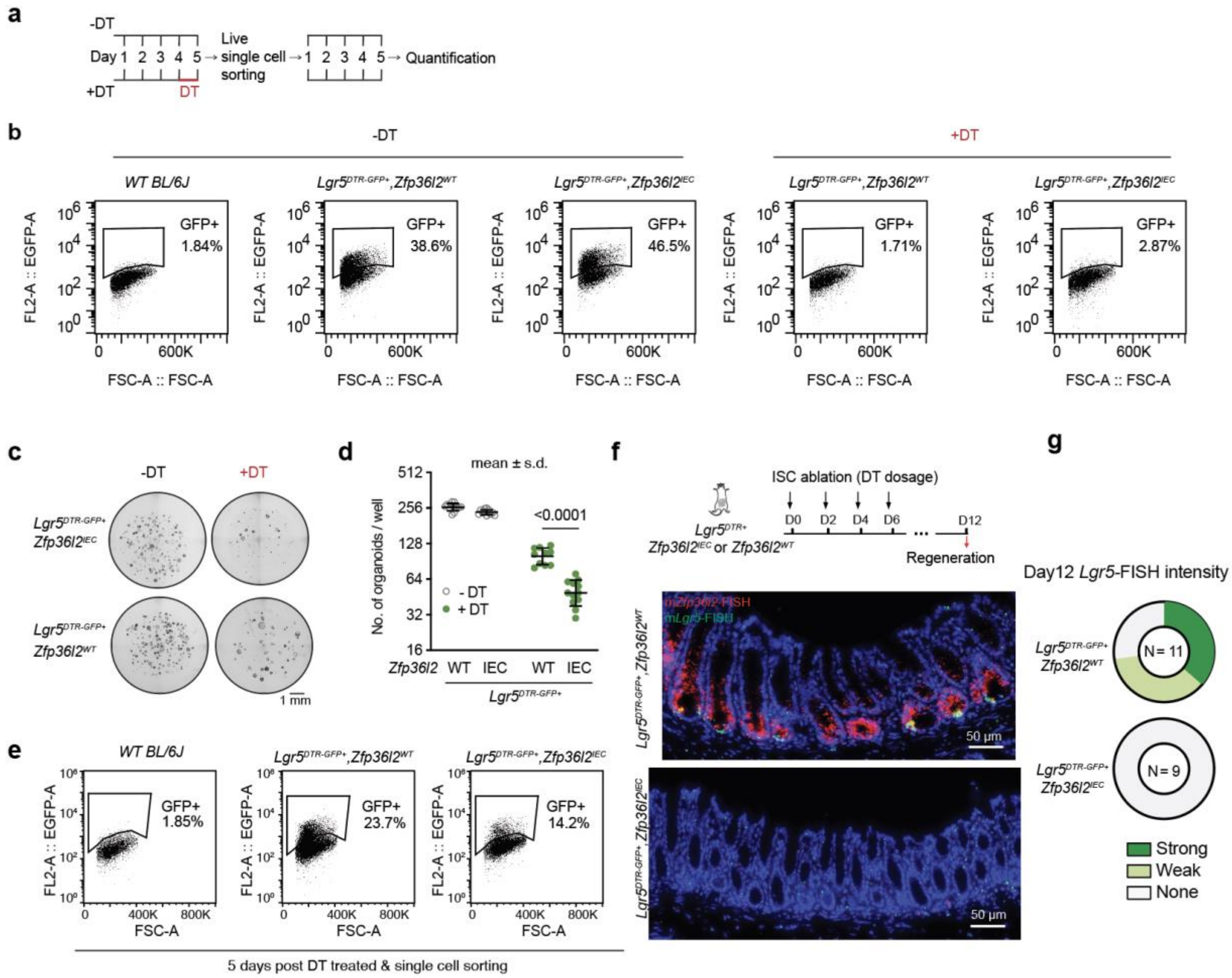

Fig. R2 (Fig. 2g,h;Extended Data Fig. 4b-f) | *Zfp36l2* is required for dedifferentiation into ISC cells. a, Schematic of LGR5-DTR ablation and organoid dedifferentiation assay. Colon organoids were derived from 6–8-week-old *Lgr5*$^{DTR\text{-}GFP-}$, *Zfp36l2*^{WT}; *Lgr5*$^{DTR\text{-}GFP+}$, *Zfp36l2*^{WT} and *Lgr5*$^{DTR\text{-}GFP+}$, *Zfp36l2*^{IEC} mice, and maintained in mouse intestinal stem cell medium (mWRENAFI). After 4 days in culture, diphtheria toxin (DT; 0.6 μg/mL) was added to the +DT group for 24 h. DT-treated and untreated organoids were then dissociated, live cells were harvested by sorting, and single cells were reseeded at low density (2,000 cells per 40 μL Matrigel per well) in mWRENAFI to induce dedifferentiation. Regenerated organoids (organoids per well) were quantified 5 days after reseeding. **b**, Representative flow cytometry density plots showing the frequency of LGR5-DTR-GFP positive cells in organoids with or without 24 h DT treatment, measured at day 5 (the time point used for sorting in the dedifferentiation assay). **c**, Representative brightfield images of regenerated organoids in individual wells 5 days after reseeding of live single cells in mWRENAFI. n = 3 mice per genotype; 3–4 wells per mouse. Scale bar, 1 mm. **d**, Quantification of regenerated organoids per well from (**c**). Data are mean ± s.d. Two-tailed Mann–Whitney *U* test. **e**, Representative flow cytometry density plots showing regeneration of LGR5-DTR-GFP positive cells following reseeding of live single cells from the DT treated group. **f**, Schematic of *in vivo* DT administration to ablate LGR5-DTR-GFP+ colon ISCs and assess ISC regeneration. Colon Swiss rolls were collected at day 12 after the first DT dose (top). Dual mRNA FISH for *mLgr5* and *mZfp36l2* was performed on day-12 Swiss-roll sections to evaluate ISC regeneration (bottom). **g**, Quantification of the number of mice showing regeneration of *Lgr5*-FISH signal

at day 12 in the experiment shown in Fig. 2g. Strong indicates complete regeneration; Weak indicates patchy recovery of *Lgr5*-FISH signal; None indicates no recovery of *Lgr5*-FISH signal.

R2.5b Villin-Cre expression is patchy in the colon (Ref. 56), especially the distal colon, where DSS damage tends to be highest. To confirm gene loss, the authors should genotype the recombined allele in colonic epithelium (as shown for unrecombined tail DNA, Extended Data Fig. 3b) and show IF images for ZFP36L2 in tissue "Swiss rolls" from IEC-KO and control colons.

R2.2.b: Although Villin-Cre expression has been reported to be patchy in the colon depending on mouse microbiomes and other conditions in specific animal facilities, in our experimental conditions in our animal facility, we observed efficient Villin-Cre dependent loss of Zfp36l2 in our IEC-KO mice. As suggested by the reviewer, to directly confirm efficient epithelial recombination, we performed PCR-based genotyping of the recombined allele using DNA isolated from colonic crypt epithelium, which demonstrates robust recombination in the colonic epithelium from IEC-KO mice (**Fig. R5a**). In addition, in accordance with the Reviewer's suggestion we also included immunofluorescence staining for ZFP36L2 on colon tissue Swiss rolls from both IEC-KO and control mice (**Fig. R5b**). These analyses show efficient and widespread loss of ZFP36L2 throughout the colonic epithelium, rather than patchy deletion. Together, these data confirm effective and uniform epithelial-specific deletion of *Zfp36l2* in the colon. These data are now included in the revised **Extended Data Fig. 3a *and* Supplementary data 4**

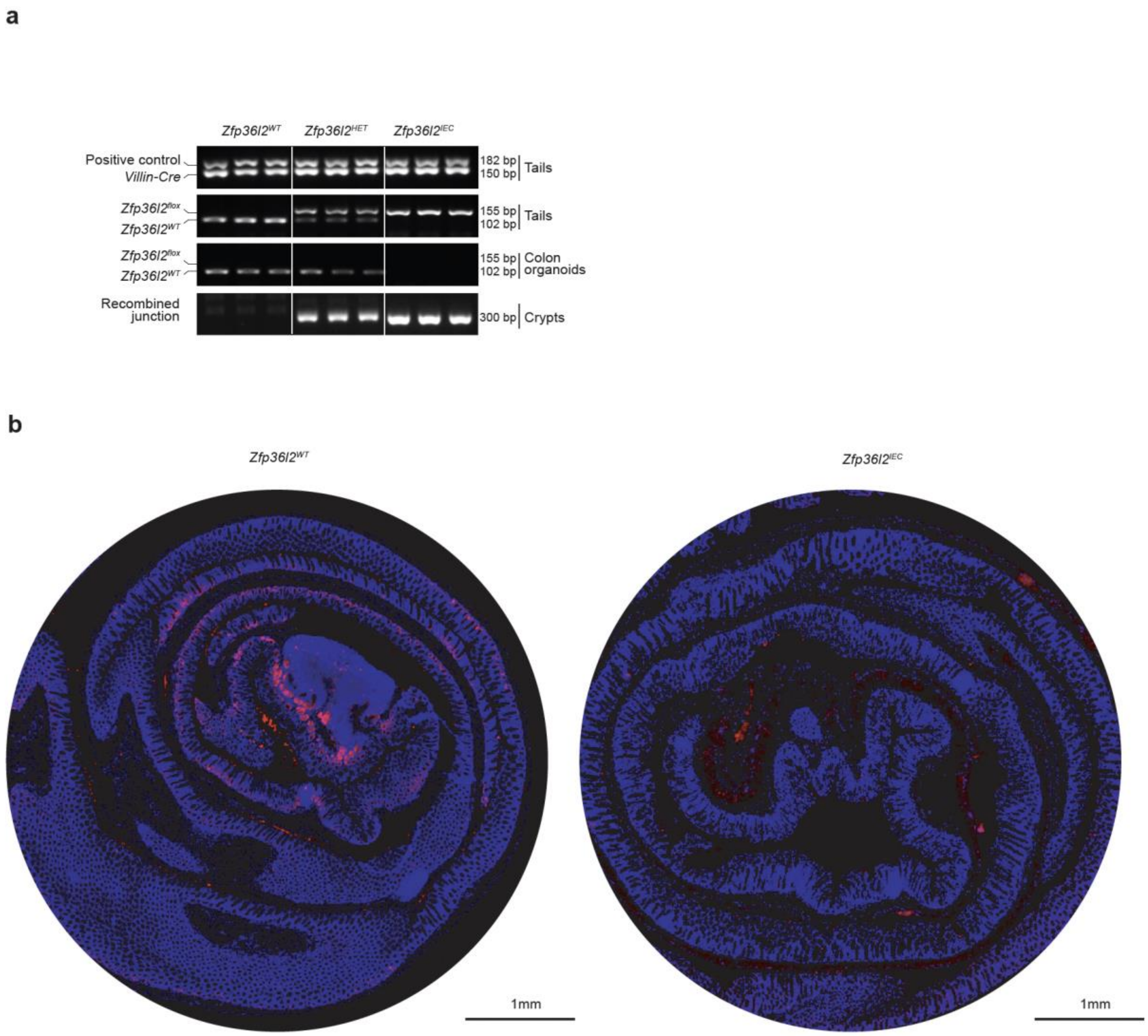

Fig. R5 (Extended Data Fig. 3a, Supplementary Data 4) | Verification of efficient *Zfp36l2* knockout in mouse colonic crypts. a, Complete genotyping panel for *Zfp36l2* floxed mice. In addition to the original genotyping panel, the recombined junction region was amplified by PCR to confirm efficient excision of the loxP-flanked region by Villin-Cre in mouse colonic crypts. **b,** Swiss roll view of ZFP36L2 immunofluorescence showing efficient loss of ZFP36L2 in the colons of *Zfp36l2*^{IEC} mice, with *Zfp36l2*^{WT} mice as controls.

R2.5c. Lgr5+ ISCs are selectively ablated in Lgr5-DTR mice (this can be confirmed using the GFP marker that is included in the Lgr5-DTR allele). Can the authors explain why all ZFP36L2+ cells are missing on Day 7 (Extended Data Fig. 3a)? Images from Days 8-10 seem to fall in the range of variation expected from different control images in the study and the large void spaces should be explained. To support the conclusions, larger areas of tissue should be shown, with accurate quantification of cell positions.

We apologize for the lack of clarity of the representative images selected in our original submission. ZFP36L2+ cells are present in the mid-crypt region (Lgr5 negative transit amplifying zone) on Day 7. We have now replaced the Day 7 image with a section that shows this more clearly (**Fig. 2f**)

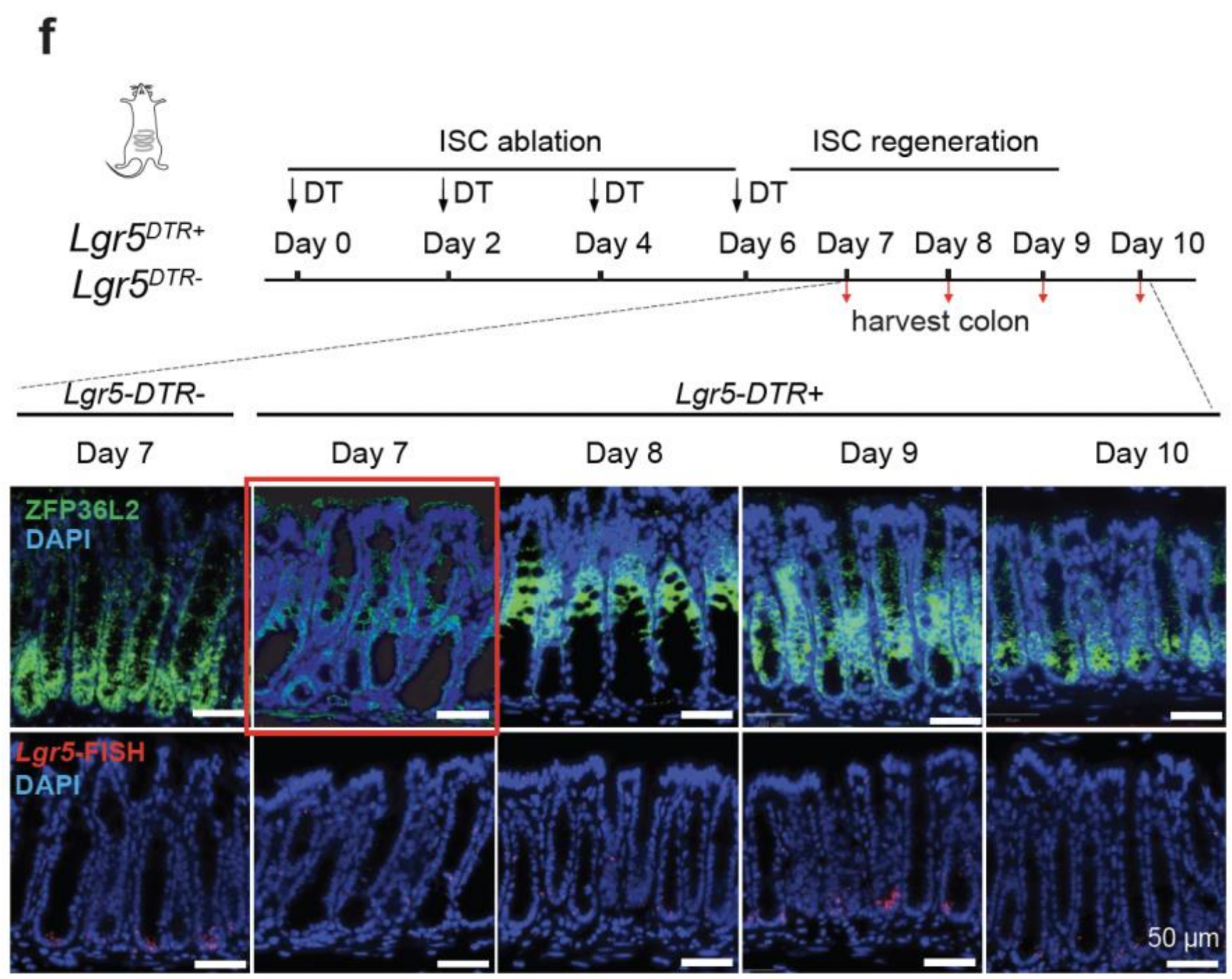

Fig. 2f | *LGR5-DTR* ablation. (Top) Schematic of experimental design indicating the intraperitoneal (IP) injection schedule of diphtheria toxin (DT; 0.05 µg per gram of body weight per dose) and timing of euthanasia and colon tissue harvest. (Bottom) Representative images (of 2 mice) of colon sections stained with *LGR5*-FISH or ZFP36L2-IF at indicated time points. *Lgr5-DTR*-negative mice treated with four doses of DT over 7 days served as controls to demonstrate specificity of DT-mediated LGR5+ cell ablation in *Lgr5-DTR*-positive mice.

As recommended by the reviewer, we now also include larger sections of swiss rolls generated from Lgr5-DTR- and Lgr5-DTR+ mice at time time point (**Supplementary Data 5**), and have accurately quantified cell positions and ZFP36L2 IF intensity in each region of the crypt over time after Lgr5-DTR ablation (**Extended Data Fig. 4a**). We appreciate the reviewer's suggestions that have enabled us to more rigorously demonstrate the expression dynamics of ZFP36L2 in different regions of colon crypts following Lgr5-DTR ablation.

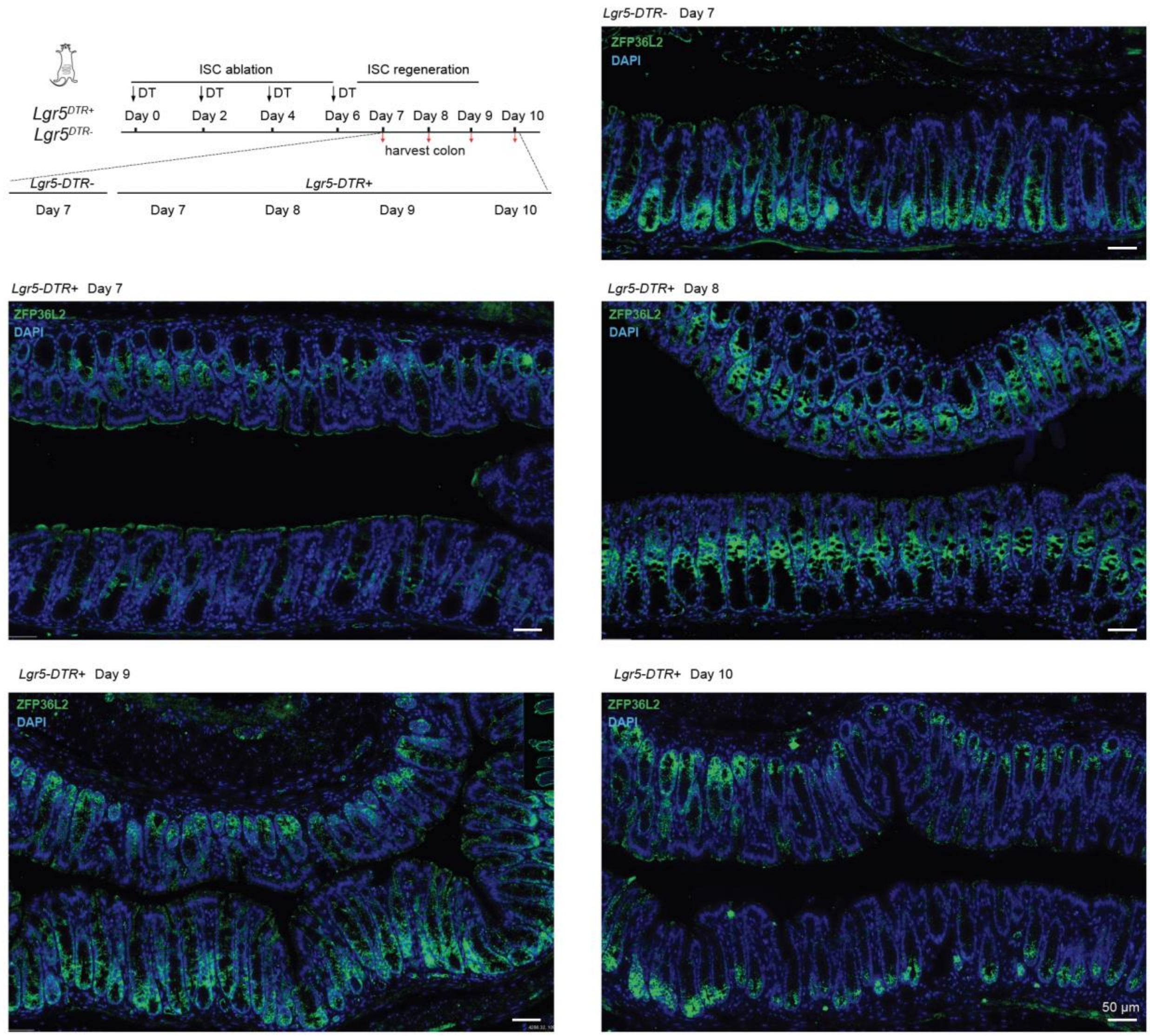

Supplementary Data 5. Larger areas of colon sections shown in Fig. 2f. *LGR5-DTR* ablation. (Top left). Schematic of experimental design indicating the intraperitoneal (IP) injection schedule of diphtheria toxin (DT; 0.05 µg per gram of body weight per dose) and timing of euthanasia and colon tissue harvest. Representative images of colon sections stained with *LGR5*-FISH or ZFP36L2-IF at indicated time points. *Lgr5-DTR*-negative mice treated with four doses of DT over 7 days served as controls to demonstrate specificity of DT-mediated LGR5+ cell ablation in *Lgr5-DTR*-positive mice.

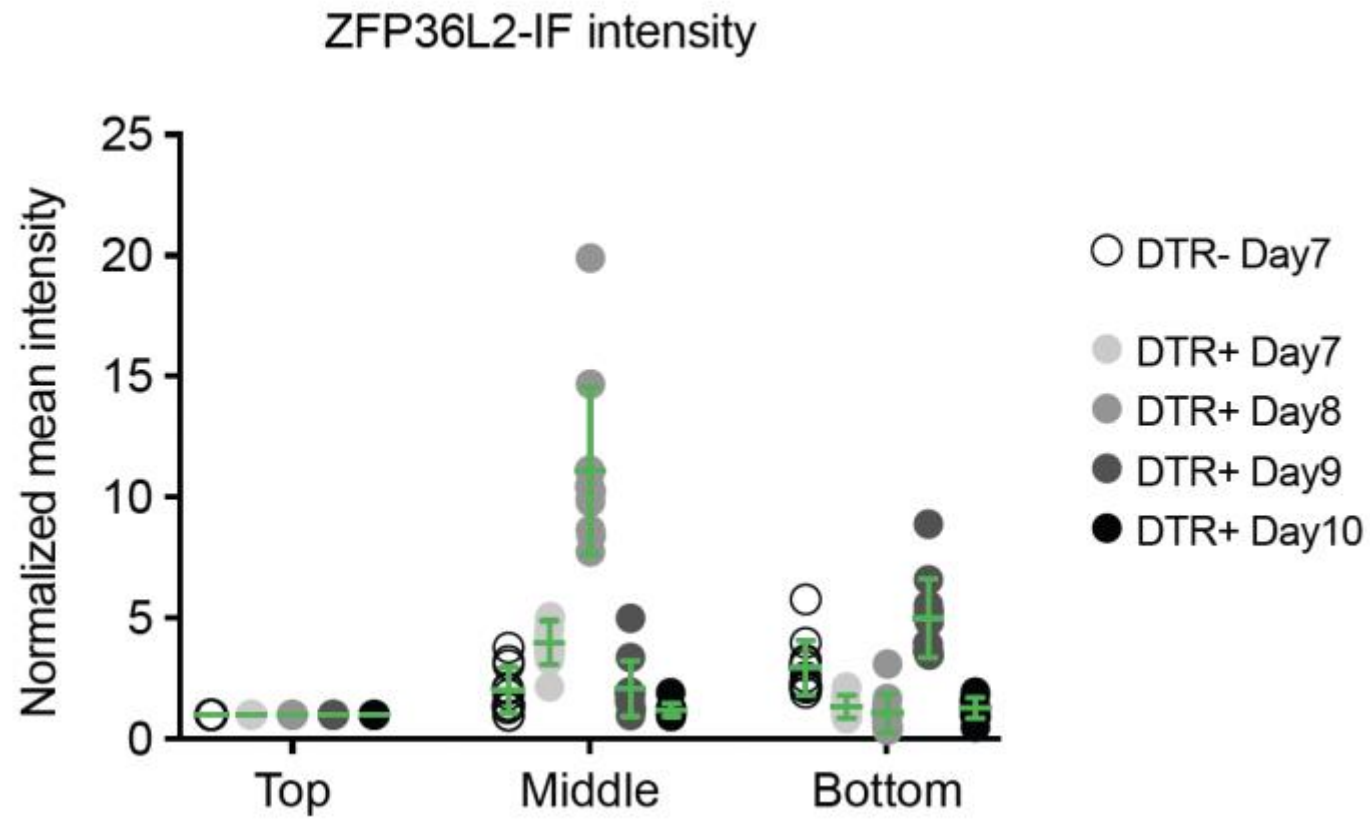

Extended Data Fig. 4a. Quantification of ZFP36L2-IF intensity dynamics shown in Fig. 2f. For each crypt region, cells were divided into the top, middle, and bottom thirds along the crypt axis. Top, top third; Middle, middle third; Bottom, bottom third. n = 11 regions. Mean ZFP36L2-IF intensity for each region was normalized to the local top one-third signal. Each dot represents one region; green bars indicate mean ± s.d.

R2.5d. The reduction of Lgr5+ ISCs in ZFP36L2-KO mice is intriguing and should be fleshed out. Can the authors explain and demonstrate it more persuasively? Fig. 2e displays only a few crypts and the experiment with Lgr5-Gfp quantitation (Extended Data Fig. 3e-g) is fraught because the Lgr5-Gfp-Cre allele has mosaic expression and few colonic crypts are labeled. Flow cytometry cannot distinguish between fewer labeled crypts (this number varies between mice) and fewer Lgr5+ cells per crypt. As indicated above, the authors should show larger areas (or "Swiss rolls") so that the range of intrinsic crypt-to-crypt variation can be assessed. Findings in mutant intestines should be quantified and interpreted in that context. Are stem cell numbers also reduced in the small intestine (where Lgr5 marker expression is more robust and can be supplemented with in situ hybridization for Olfm4).

We agree with the Reviewer that the reduction of *Lgr5*$^{+}$ ISCs in ZFP36L2-KO mice is an intriguing observation. Regarding the robustness of the demonstration of *Zfp36L2* knockout and association with *Lgr5* expression, as the Reviewer points out, the *Lgr5-GFP-Cre* allele is notoriously mosaic in expression. This was why we did not use *Lgr5-GFP-Cre* driver to drive recombination of the Zfp36L2$^{fl/fl}$ allele and instead used the *Lgr5-GFP-Cre* allele solely for robust flow cytometry-based quantification of *Lgr5*-expression (**Extended Data Fig. 3g-i**). We used the *Villin-Cre*, which is more uniformly expressed across the colon to drive *Zfp36L2* knockout. As per the Reviewer's suggestion, we now include images spanning larger tissue areas (**Fig. R9a**), to capture intrinsic crypt-to-crypt variability, together with quantification of *Lgr5*$^{+}$ cells across entire Swiss roll sections from *Zfp36l2*^{WT} and *Zfp36l2*^{IEC} mice (**Fig. R9b**). These analyses further confirm a consistent reduction in *Lgr5* signal in Villin-Cre driven *Zfp36l2* knockout colons across large tissue areas. These data are now included in the revised **Extended Data Fig. 3f *and* Supplementary Data 2.**

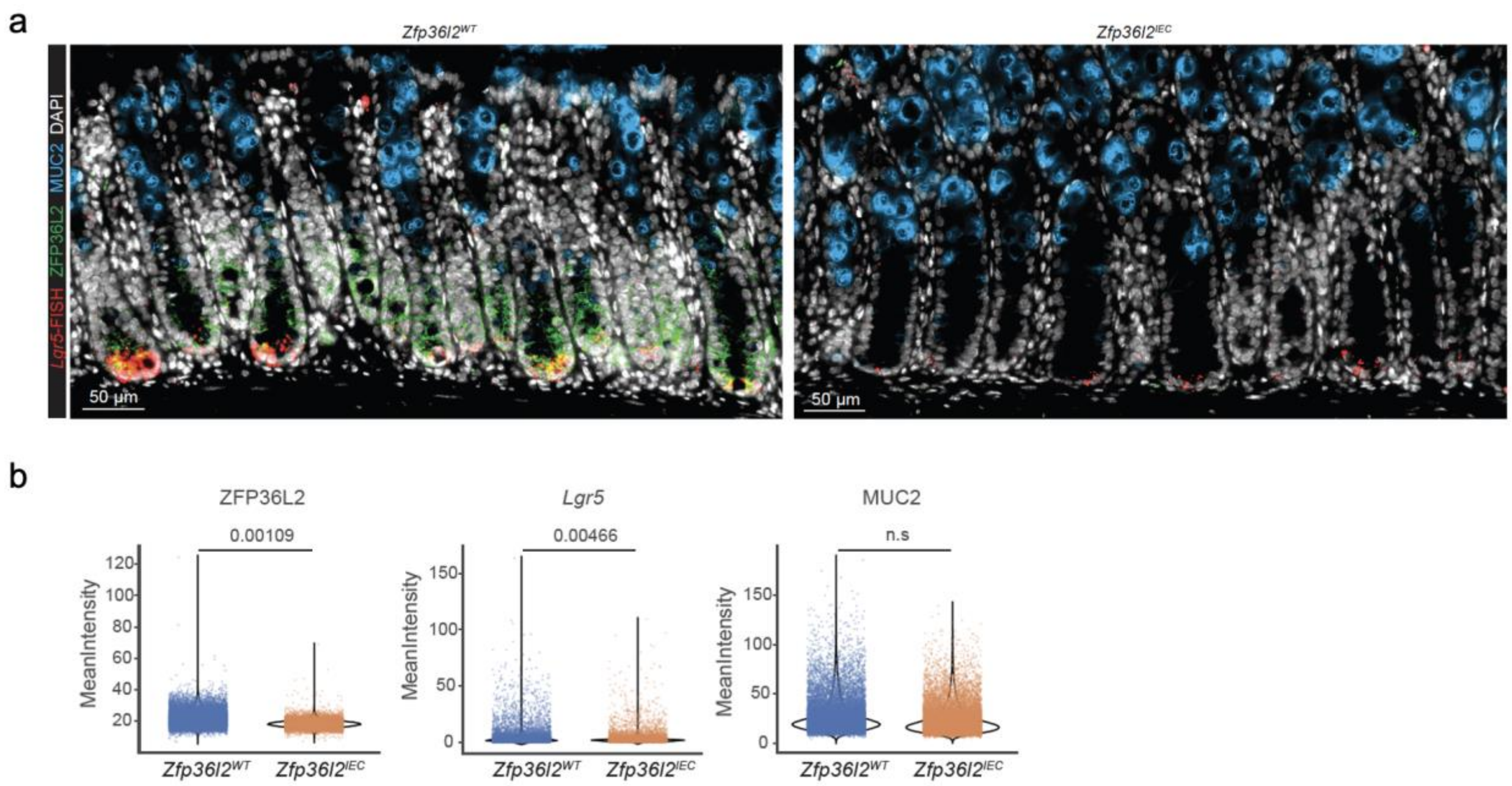

Fig. R6 (Extended Data Fig. 3f, Supplementary Data 2) | Reduced *Lgr5* signal in colons from Villin-Cre–driven *Zfp36l2* knockout mice. a. Larger region of tissue shown in **Extended Data Fig. 3e.** Representative images of *Lgr5*-FISH, ZFP36L2-IF and MUC2-IF of colon tissue from 6-week-old *Zfp36l2*^{WT} and *Zfp36l2*^{IEC} mice. **b**, Quantification of (**a**) ZFP36L2 and MUC2 mean fluorescence intensity by immunofluorescence, and *Lgr5* RNA abundance by RNA FISH, in colonic crypt cells from Swiss rolls of *Zfp36l2*^{WT} and *Zfp36l2*^{IEC} mice. Violin plots with individual cells overlaid as dots are shown. Each dot represents one cell. Swiss roll regions containing U-shaped crypts were quantified from 3 mice per group. Two-tailed Mann–Whitney *U* test.

We did not examine stem cell numbers in the small intestine. DSS-induced colitis predominantly affects the colon, and the central biological questions addressed in this study—epithelial regeneration following injury and colorectal cancer–associated plasticity—are specific to colonic epithelium. Analysis of small intestinal stem cells would therefore fall outside the scope of the present work.

R2.5e. The dedifferentiation assay in organoids is not widely accepted as standard and there are many explanations for the findings (Fig. 2h-k), such as inherent epithelial defects unrelated to dedifferentiation. Increased sensitivity to immune damage could underlie DSS pathology but is an improbable explanation for findings in Lgr5-DTR mice. If the different in vivo studies are done carefully (as outlined above), perhaps adding irradiation as another trigger for dedifferentiation, the organoid assay adds little to the study. The concern about organoid dedifferentiation extends to its application to support ideas on CRC dedifferentiation (Fig. 3l-n).

R2.2.e: We appreciate the Reviewer's comment regarding the interpretation of organoid-based dedifferentiation assays. To ensure that our organoid system was rigorously controlled, we quantitatively validated the culture conditions used in these experiments. Withdrawal of WNT and R-spondin led to robust differentiation and loss of *Lgr5*+ ISCs (**Extended data Fig. 4h,i**), confirming that the assay effectively drives exit from the stem cell state before regenerative

reprogramming is assessed. We therefore believe that the dedifferentiation assays presented in the original manuscript were performed carefully and interpreted conservatively.

To more directly assess the function of ZFP36L2 in dedifferentiation, to complement our *in vivo* DSS colitis (**Fig. 2d,e**) and *ex vivo* organoid formation experiments (**Extended data Fig. 4g-l**), we have now performed a new set of experiments in an orthogonal models system developed specifically to address this question. We generated *$Lgr5^{DTR\text{-}GFP+}$, $Zfp36L2^{IEC}$* and *$Lgr5^{DTR\text{-}GFP+}$, $Zfp36L2^{WT}$* mice, administered diphtheria toxin to selectively ablate LGR5+ ISCs, and assayed the restoration of LGR5 expression through dedifferentiation of LGR5- cells in *Zfp36L2* WT vs knockout contexts both *in vivo* and in *ex vivo* organoids. The $Lgr5^{DTR\text{-}GFP+}$ mouse model has been used widely in the field to specifically study this question of dedifferentiation and LGR5+ ISC replenishment following crypt base LGR5+ ISC ablation[18,20].

Consistent with our prior observations, we find that loss of Zfp36l2 impaired recovery of LGR5+ crypt base intestinal stem cells following the ablation of LGR5+ cells (**Fig. R2**). Our findings now across two independent *in vivo* mouse models of LGR5+ ISC loss (DSS colitis and *$Lgr5^{DTR\text{-}GFP+}$*) and in *ex vivo* organoid dedifferentiation and regeneration assays, definitively support our conclusion that *Zfp36l2* is critical for injury-induced de-differentiation. These new data and quantifications have been added to the revised manuscript (**Fig. 2g,h; Extended Data Fig. 4b-f;** Results, page 5, lines 143-161; Methods, page 65, lines 1432-1460).

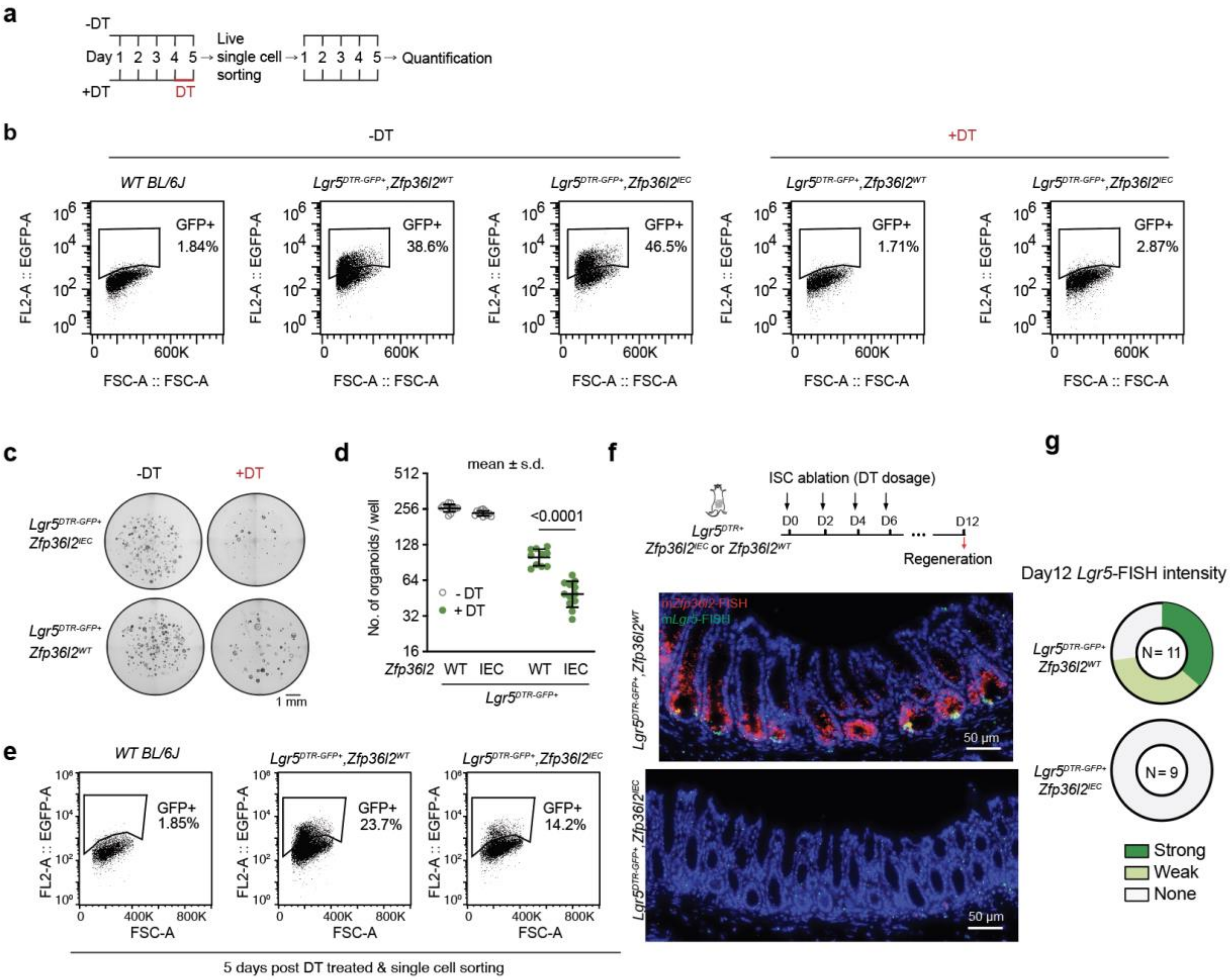

Fig. R2 (Fig. 2g,h;Extended Data Fig. 4b-f) | *Zfp36l2* is required for dedifferentiation into ISC cells. a, Schematic of LGR5-DTR ablation and organoid dedifferentiation assay. Colon organoids were derived from 6–8-week-old *Lgr5*$^{DTR\text{-}GFP-}$*, Zfp36l2*^{WT}*; Lgr5*$^{DTR\text{-}GFP+}$*, Zfp36l2*^{WT} and *Lgr5*$^{DTR\text{-}GFP+}$*, Zfp36l2*^{IEC} mice, and maintained in mouse intestinal stem cell medium (mWRENAFI). After 4 days in culture, diphtheria toxin (DT; 0.6 µg/mL) was added to the +DT group for 24 h. DT-treated and untreated organoids were then dissociated, live cells were harvested by sorting, and single cells were reseeded at low density (2,000 cells per 40 µL Matrigel per well) in mWRENAFI to induce dedifferentiation. Regenerated organoids (organoids per well) were quantified 5 days after reseeding. **b**, Representative flow cytometry density plots showing the frequency of LGR5-DTR-GFP positive cells in organoids with or without 24 h DT treatment, measured at day 5 (the time point used for sorting in the dedifferentiation assay). **c**, Representative brightfield images of regenerated organoids in individual wells 5 days after reseeding of live single cells in mWRENAFI. n = 3 mice per genotype; 3–4 wells per mouse. Scale bar, 1 mm. **d**, Quantification of regenerated organoids per well from (**c**). Data are mean ± s.d. Two-tailed Mann–Whitney *U* test. **e**, Representative flow cytometry density plots showing regeneration of LGR5-DTR-GFP positive cells following reseeding of live single cells from the DT treated group. **f**, Schematic of *in vivo* DT administration to ablate LGR5-DTR-GFP+ colon ISCs and assess ISC regeneration. Colon Swiss rolls were collected at day 12 after the first DT dose (top). Dual mRNA FISH for *mLgr5* and *mZfp36l2* was performed on day-12 Swiss-roll sections to evaluate ISC regeneration (bottom). **g**, Quantification of the number of mice showing regeneration of *Lgr5*-FISH signal

at day 12 in the experiment shown in Fig. 2g. Strong indicates complete regeneration; Weak indicates patchy recovery of *Lgr5*-FISH signal; None indicates no recovery of *Lgr5*-FISH signal.

R2.5f. The authors did use two different dox-inducible shRNAs in the study, which is nice but does not strictly exclude off-target effects. Rescue of at least one shRNA in some assay(s) would increase confidence in the authors' conclusions.

We appreciate the Reviewer's comment regarding the possibility of off-target effects associated with shRNA-based knockdown. While we used two independent doxycycline-inducible shRNAs targeting *ZFP36L2* and repeated experiments with multiple independent patient-derived organoid models, we agree that rescue experiments could further increase confidence in specificity. However, cDNA rescue of ZFP36L2 expression markedly suppresses proliferation while inducing injury-associated markers such as L1CAM (**Fig. R7**), making cDNA-based rescue difficult to interpret, as it introduces additional overexpression-related biological effects that confound straightforward phenotypic reversal.

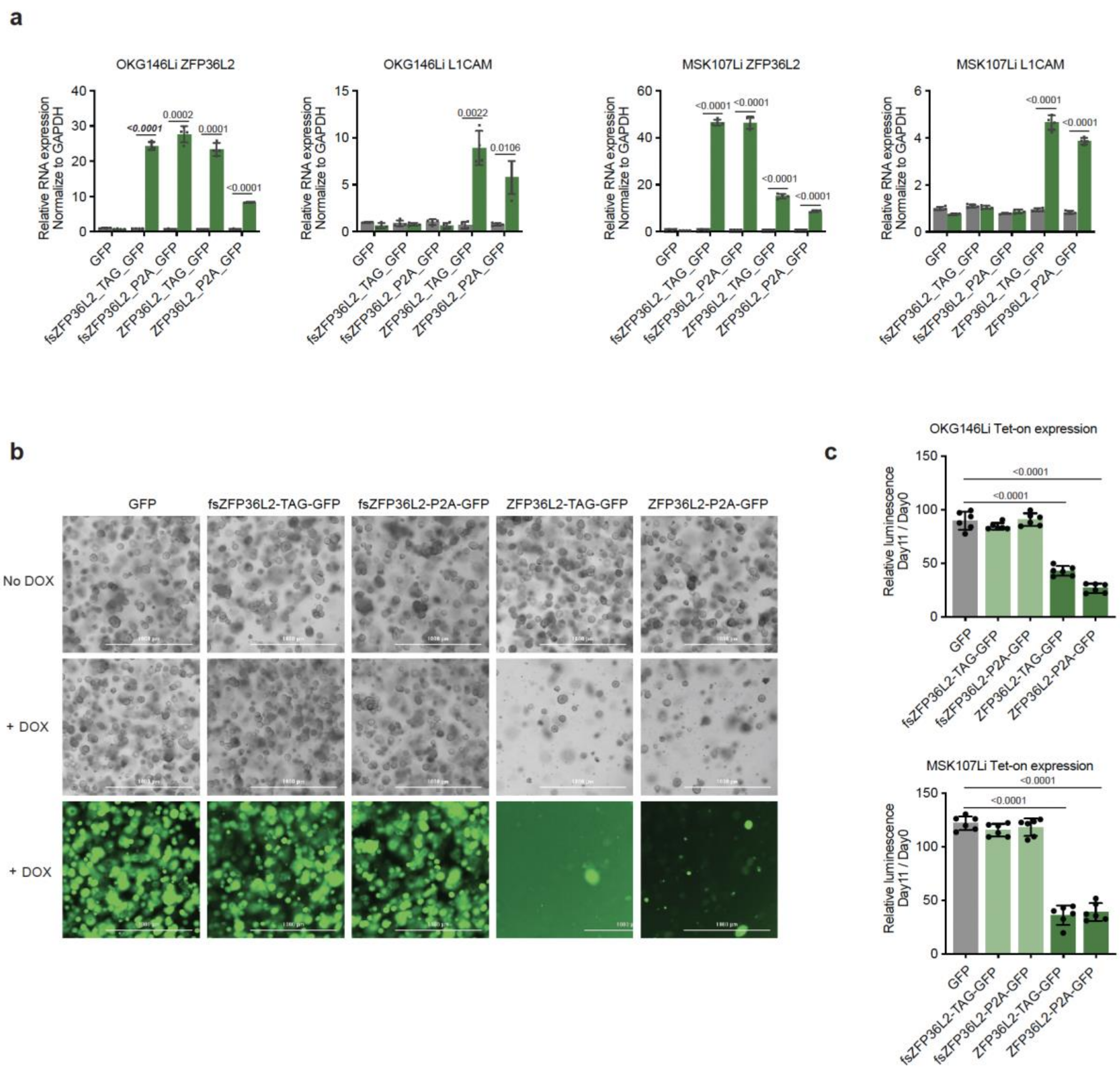

Fig. R7 | Overexpression of *ZFP36L2* traps organoids in a low-cycling injury-repair state. **a**, qPCR analysis of *ZFP36L2* and *L1CAM* expression, normalized to *GAPDH*, in OKG146Li and MSK107Li organoids expressing doxycycline-inducible Tet-on constructs encoding GFP, GFP-tagged ZFP36L2, GFP-tagged frameshift ZFP36L2 (fsZFP36L2), or P2A-GFP versions of ZFP36L2 and fsZFP36L2. Data are mean ± s.d. Two-tailed Mann–Whitney *U* test. **b**, Representative bright-field and GFP images of MSK107Li organoids with indicated construct at day 8. **c**, Cell viability measured by CellTiter-Glo in organoids grown in HISC medium with 2 μg/mL at day 11 and normalized to day 0. Data are mean ± s.d. Two-tailed Mann–Whitney *U* test.

To definitively exclude shRNA off-target artifacts, we employed an independent CRISPR–Cas9–based loss-of-function strategy to knockout *ZFP36L2* using two distinct guide RNAs in two patient-derived CRC organoid lines MSK107Li and OKG146Li (**Extended Data Fig. 7**). ZFP36L2. Consistent with our observations with shRNA-mediated *ZFP36L2* knockdown, CRISPR-Cas9 *ZFP36L2* knockout decreased dedifferentiation and organoid regeneration. Bulk RNAseq similarly revealed decreased ISC-associated gene signatures, and increased expression of canonical and non-canonical differentiation programs in ZFP36L2 knockout organoids in comparison with *ZFP36L2* WT controls.

Together, the concordance between shRNA knockdown with two independent shRNAs, CRISPR knockout with two independent sgRNAs, with both CRISPR knockout and shRNA knockdown performed in multiple patient-derived organoid lines, with data from both transcriptional profiling and functional regeneration assays strongly argues against off-target effects and provides further robust genetic validation of ZFP36L2's role in regulating regenerative plasticity. This comprehensive dataset far exceeds the typical standards of evidence rigor used to support functional claims, and underscores the consistency and robustness of our findings regarding ZFP36L2 function. These new data have been added to the revised manuscript (**Extended Data Fig. 7;** Results, page 6 lines 241-252; Methods, page 70, lines 1584-1591).

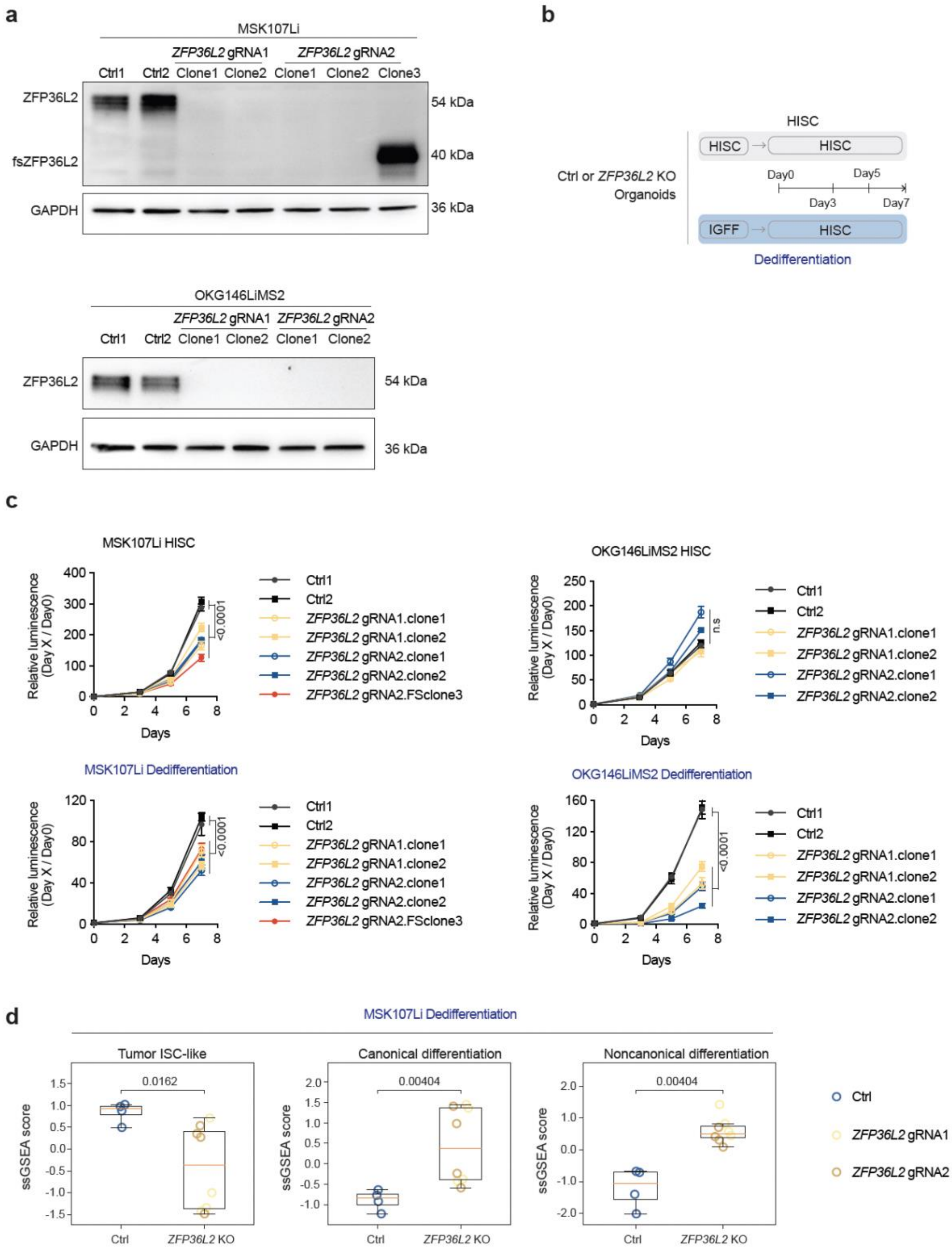
a
MSK107Li
ZFP36L2 gRNA1
ZFP36L2 gRNA2
Ctrl1
Ctrl2
Clone1
Clone2
Clone1
Clone2
Clone3
ZFP36L2
fsZFP36L2
GAPDH
54 kDa
40 kDa
36 kDa
OKG146LiMS2
ZFP36L2 gRNA1
ZFP36L2 gRNA2
Ctrl1
Ctrl2
Clone1
Clone2
Clone1
Clone2
ZFP36L2
GAPDH
54 kDa
36 kDa
b
HISC
HISC
HISC
Ctrl or ZFP36L2 KO Organoids
Day0
Day5
Day3
Day7
IGFF
HISC
Dedifferentiation
c
MSK107Li HISC
Relative luminescence (Day X / Day0)
Days
<0.0001
Ctrl1
Ctrl2
ZFP36L2 gRNA1.clone1
ZFP36L2 gRNA1.clone2
ZFP36L2 gRNA2.clone1
ZFP36L2 gRNA2.clone2
ZFP36L2 gRNA2.FSclone3
OKG146LiMS2 HISC
Relative luminescence (Day X / Day0)
Days
n.s
Ctrl1
Ctrl2
ZFP36L2 gRNA1.clone1
ZFP36L2 gRNA1.clone2
ZFP36L2 gRNA2.clone1
ZFP36L2 gRNA2.clone2
MSK107Li Dedifferentiation
Relative luminescence (Day X / Day0)
Days
<0.0001
Ctrl1
Ctrl2
ZFP36L2 gRNA1.clone1
ZFP36L2 gRNA1.clone2
ZFP36L2 gRNA2.clone1
ZFP36L2 gRNA2.clone2
ZFP36L2 gRNA2.FSclone3
OKG146LiMS2 Dedifferentiation
Relative luminescence (Day X / Day0)
Days
<0.0001
Ctrl1
Ctrl2
ZFP36L2 gRNA1.clone1
ZFP36L2 gRNA1.clone2
ZFP36L2 gRNA2.clone1
ZFP36L2 gRNA2.clone2
d
MSK107Li Dedifferentiation
Tumor ISC-like
0.0162
ssGSEA score
Ctrl
ZFP36L2 KO
Canonical differentiation
0.00404
ssGSEA score
Ctrl
ZFP36L2 KO
Noncanonical differentiation
0.00404
ssGSEA score
Ctrl
ZFP36L2 KO
Ctrl
ZFP36L2 gRNA1
ZFP36L2 gRNA2

Extended Data Fig. 7 | *ZFP36L2* knockout inhibits CRC dedifferentiation into an ISC state. a, Western blot validation of ZFP36L2 protein knockout/frameshift indel truncating mutation in MSK107Li and OKG146LiMS2 organoids. Two individual clones for each of two gRNAs targeting the N-terminal region of *ZFP36L2* were analyzed. Organoids cultured in HISC medium for 7 days were harvested for western blotting. **b-d**, Organoid dedifferentiation assay **b**, Schematic of organoid dedifferentiation assay. Organoids were either continuously cultured in HISC medium (HISC group) or cultured in IGFF medium for 13 days, then dissociated into single cells and passaged into HISC medium (Dedifferentiation group), and analyzed at the indicated time points. **c**, Organoid growth measured by CellTiter-Glo viability assays at the indicated time points and normalized to day 0 immediately after plating as single cells (1K cells/ 20 uL Matrigel/well). Data are mean ± s.d. n = 6 replicates/group. Two-tailed Mann–Whitney *U* tests between control and knockout groups. **d**, ssGSEA scores for the indicated gene sets from bulk RNA sequencing of MSK107Li control and *ZFP36L2* knockout organoid lines. Two independent clones from each gRNA were pooled. Organoid on day 7 of dedifferentiation were harvested for bulk RNA sequencing. Gene sets were the same as those used in **Fig. 3j** and **Supplementary Table 2a**. Box plots show the interquartile range (25th to 75th percentiles), horizontal line: median; whiskers: minimum and maximum values. Two-tailed Mann–Whitney *U* tests.

R2.5g. Please check the graphs in Figs. 3f and 3i. Captions indicate that they refer to the same data, but the numbers at week 13 don't match (<1000 for shCtrl in Fig. 3f but >1000 in Fig. 3i, <100 for shL2 in Fig. 3f but >100 in Fig. 3i). In summary, considerable additional rigor and explanation are required to support the conclusion that ZFP36L2 "mediates colonic epithelial regeneration" (line 108).

We thank the Reviewer for pointing out this inconsistency. Figures 3f and 3i are derived from the same experimental cohort but represent different measurements. Figure 3i shows longitudinal bioluminescence imaging (BLI) signals acquired from live mice *in vivo*, whereas Figure 3f quantifies liver metastatic burden measured *ex vivo* after liver dissection at the experimental endpoint. Because *in vivo* BLI and *ex vivo* measurements differ in sensitivity, tissue attenuation, and signal normalization, their absolute values are not directly comparable. We have revised the figure labels to clarify the distinction between these two readouts and to avoid confusion. (**Revised Fig. 3 f,i**)

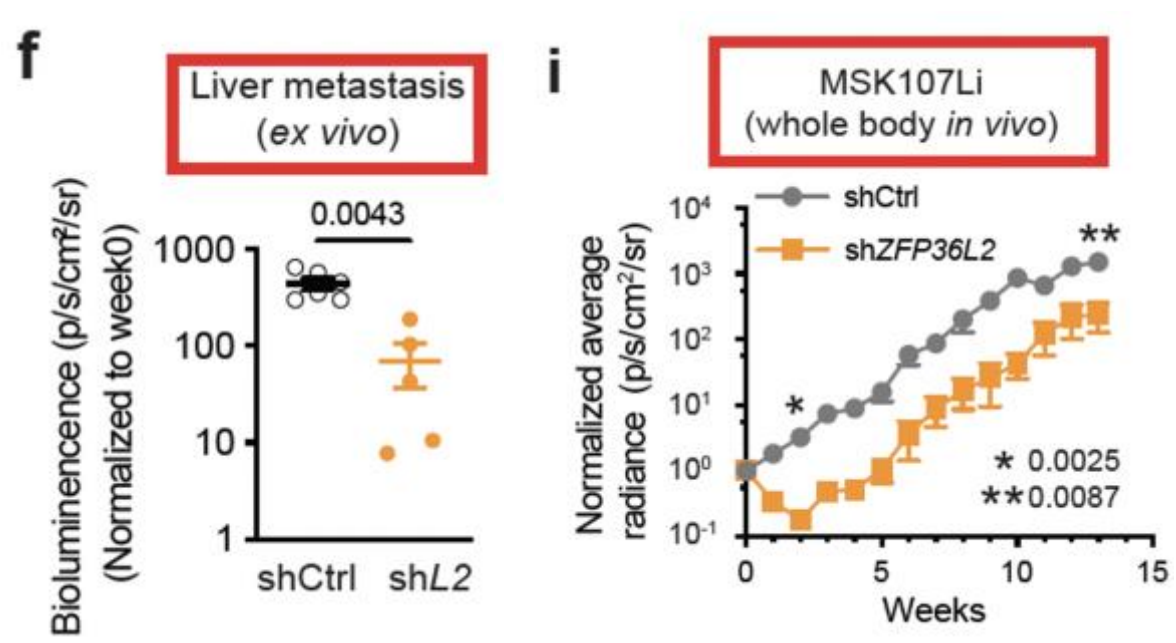

R2.5h. The manuscript stretches the idea that "CRC metastases progressively adopt a non-canonical differentiation trajectory" (line 226). Evidence for this "trajectory" in shZFP36L2 organoids is weak. The significance of changes in ssGSEA scores, e.g., 0.4 to 0.43 or 0.37 to 0.39 (Fig. 3j), is unclear and the increase in CHGB immunostaining (Fig. 3p) is not impressive. The display of small representative sections from ZFP36L2 wild-type and mutant CRC

metastases (Fig. 3q-r) poses the same problem for evaluation as noted above. IF images don't seem to match "representative" numbers graphed to the right. To assess their claims, the authors should provide large tissue areas as supplementary files.

We appreciate the reviewer's comment regarding the magnitude of the observed changes and the interpretation of a non-canonical differentiation trajectory.

(1) ZFP36L2 knockdown and knockout organoid models from multiple patient donors, and in multiple experimental conditions and assays converge on non-canonical differentiation. The ssGSEA analyses in Fig. 3j were performed on bulk RNA-seq from organoids cultured in human intestinal stem cell (HISC) media, which we have previously shown contains growth factors that strongly promote Wnt and EGF signaling and inhibit TGF-beta signaling, and induce CRC cells to retain ISC-like programs, thus reducing differences between conditions[1]. Despite the strong growth factor rich media-driven convergence in gene expression, we find that all shZFP36L2 organoid lines reproducibly exhibit a shift toward differentiation programs. Notably, similar trends are observed in our scRNA-seq datasets, where *ZFP36L2* knockdown cells exhibit reduced ISC signatures and elevated differentiation markers; importantly, these differences are further amplified under dedifferentiation conditions (**Fig. 3k-n; Extended Data Fig. 6**). This context-dependent amplification of the difference in ISC and differentiation program expression between shCtrl and ZFP36L2 knockdown CRC organoids provides strong support for a role of ZFP36L2 in regulating dedifferentiation dynamics. Further, in new data generated during this revision, we generated ZFP36L2 knockout organoids and performed bulk RNA-seq under dedifferentiation culture conditions (**Extended Data Fig.7**). ZFP36L2-knockout organoids displayed markedly constrained re-acquisition of ISC programs and enhanced canonical and non-canonical differentiation signatures compared to controls.

(2) Patient CRC metastases and PDO xenograft models *in vivo* provide strong evidence for non-canonical differentiation upon ZFP36L2 loss of function. Nevertheless, we agree that *ex vivo* organoid conditions cannot fully recapitulate the *in vivo* tumor microenvironment required for full-blown differentiation, since organoid conditions by definition are designed to promote convergence into stem-like states[1]. This was the rationale for carefully examining tissues from both sh*ZFP36L2*/sh*Ctrl* human organoid liver xenografts (**Fig. 3p**) as well as from ZFP36L2 mutant human metastatic tumors (**Fig. 3q,r**). In both cases, especially and crucially in the *ZFP36L2* mutant human tumor context where metastatic tumors grow out over time under the selective pressures imposed by tumor *ZFP36L2* mutation, we find striking and consistent evidence for non-canonical gene expression in ZFP36L2-deficient metastatic tumors growing in the liver microenvironment. We respectfully disagree that the increase in CHGB immunostaining (**Fig. 3p**) is biologically trivial. In control tumors generated from shCtrl organoids, CHGB-positive cells are nearly absent, with no appreciable clusters. In contrast, sh*ZFP36L2* consistently exhibit CHGB-positive cells across nearly all samples. The apparent modest percentage values reflect quantification across whole liver sections, where the substantial total tumor cell number dilutes the relative frequency of positive cells. Despite this conservative quantification strategy, the difference between control and knockdown tumors remains statistically robust.

Notably, the increase in CHGB+ and CK5+ non-canonical neuroendocrine and squamous cells respectively, with concomitant decrease in LGR5+ ISCs was even more striking in *ZFP36L2* mutant patient CRC metastases in comparison to sh*ZFP36L2* PDO xenograft metastases,

suggesting strong selective pressure for non-canonical differentiation during metastatic outgrowth in the context of complete mutation-driven tumor loss of *ZFP36L2* function in patients.

Also, please explain the meaning of negative values on the y-axes in Figs. 3n and 3r. Considering what is represented (expression or % positive cells), negative numbers are not intuitive and likely reflect very small values in both cases.

The y-axes represent % positive cells/ROI. Negative numbers represent small percentages (i.e. 10^{-3} = 0.001%). We have also provided larger-area images as supplementary files (**Supplementary Data 6**) to allow assessment of overall tissue context.

Together, our comprehensive and orthogonal data from multiple models and most importantly, clinical metastases, spanning (1) bulk and (2) single cell RNA sequencing from 4 patient-derived organoid lines with shRNA-mediated *ZFP36L2* knockdown, (3) bulk RNAseq from CRISPR *ZFP36L2* knockout organoids, and (4) multiplexed immunostaining of organoid-derived sh*ZFP36L2* orthotopic liver xenografts *in vivo* and (5) clinical *ZFP36L2* mutant CRC liver metastases, together present a robust and consistent evidence base for the enrichment of non-canonical programs in *ZFP36L2*-deficient CRC metastases.

R2.6 Cancers, especially metastases, express many genes aberrantly. It is unclear if "non-canonical" differentiation (which includes CHGB, a product of native ISC progeny) reflects something as specific as the authors assume and is not just the tip of a broadly aberrant iceberg. Extended Data Fig. 5 is not especially informative. A clearer definition and persuasive demonstration of ZFP36L2-mediated differences would help.

We appreciate the reviewer's request for a clearer definition of "non-canonical" differentiation and a more persuasive demonstration of ZFP36L2-mediated differences.

In our manuscript, we use the term non-canonical differentiation in the specific sense we initially defined in Moorman et al., 2025, where colorectal cancer progression follows a conserved trajectory: canonical intestinal states → tumour ISC-like states → fetal progenitor intermediate → divergent non-canonical lineages, including squamous-like and neuroendocrine-like programs. Importantly, these non-canonical modules were derived from unsupervised Hotspot analysis of patient tumour epithelial cells and represent CRC-intrinsic transcriptional programs that are enriched in the transition from primary tumours to metastases, crucially are associated with poor clinical outcomes[1]. Thus, "non-canonical" does not simply denote aberrant or random gene expression, and is not based on assumptions, but refers to defined gene modules (e.g., squamous and neuroendocrine programs) identified through unbiased computational analysis and rigorously established to reproducibly emerge along a conserved axis of plasticity during metastatic progression across multiple patient cohorts[1]. CHGB, as discussed by the Reviewer, is part of the tumor-specific neuroendocrine module (which is distinct from the native enteroendocrine program and lacks expression of canonical intestinal genes such as CDX2) within this defined non-canonical spectrum. Therefore, its expression reflects entry into a transcriptionally coherent lineage program rather than stochastic dysregulation. Consistent with this framework, we observed that ZFP36L2 perturbation shifts organoids along this canonical-to-non-canonical axis rather than inducing broad, unstructured gene expression changes.

As we mentioned above, with CRISPR-mediated *ZFP36L2* knockout and under defined dedifferentiation conditions, bulk RNA-seq revealed a more pronounced decrease in expression of canonical ISC programs and increased differentiation toward both canonical and non-canonical states (**Extended Data Fig. 7**).These transcriptional shifts were concordant with reduced regenerative efficiency *ex vivo*, reinforcing that *ZFP36L2* regulates plastic state transitions rather than merely affecting steady-state proliferation. The gene expression matrices in all *ZFP36L2* perturbation conditions are provided (**Supplementary Table 3b**) for further interrogation of *ZFP36L2*-dependent transcriptional reprogramming.

Taken together, non-canonical differentiation in our study is not interpreted as a vague marker of aberrancy, but as movement along a defined and conserved plasticity trajectory rigorously established in patient metastases[1]. Our data support a role for *ZFP36L2* in regulating positioning along this trajectory.

R2.7 To the best of my [limited] understanding, the point that the RNA-binding protein ZFP36L2 participates, along with RNA molecules, in phase-separated cytoplasmic condensates appears valid. The videos from live cell imaging are clear, though I'm unsure what exactly the authors mean by "dynamic" condensates. Cells are moving while being imaged and many of the IF puncta look similar across frames, likely reflecting cell movement. Perhaps this is why the authors refer to "dynamic, mobile condensates" but the significance of those descriptors is unclear. More importantly, the observation that condensates are more abundant in single cells than in organoids does not merit the conclusion that ZFP36L2 plays "a role in tumour cell adaptation" (line 289). Even if the short period of peak condensate formation coincides with "dedifferentiation," that is an association at best, and the authors don't consider the innumerable alternative reasons. Adding to the confusion, static (Fig. 4b) and live (Suppl. Videos) organoid imaging show many more condensates per cell than the graph in Fig. 4f indicates (1-2 per cell). In summary, the link between condensate formation and dedifferentiation is very weak.

We thank the reviewer for carefully evaluating the condensate imaging data and for raising important points regarding interpretation.

First, by "dynamic" condensates we specifically refer to their active intracellular behavior, including directional movement, fusion, and fission events. To address the reviewer's concern that apparent motion might simply reflect whole-cell displacement during imaging, we have now performed image drift correction and quantitative puncta tracking on live cell microscopy of three organoid lines (MSK107Li, OKG146P, OKG146Li) (**Fig. 4e; Supplementary videos 6**). These analyses demonstrate that individual condensates move independently and in distinct directions within the same cell, confirming that their motion is not attributable to global cellular movement. In addition, new live-imaging videos directly show fusion (**Supplementary Video 7**) and dissolving (**Supplementary Video 8**) events over time, further supporting the conclusion that these are dynamic phase-separated structures rather than static aggregates. These new data have been added to the revised manuscript (**Fig. 4e**). Methodological details of image drift correction and condesate tracking analysis are provided (Methods, lines 1863-1874).

Second, we agree with the reviewer that the temporal coincidence between peak condensate formation and injury-induced dedifferentiation establishes correlation rather than causation. To

avoid confusion, we remove the speculation (line 289, original submission) “highlighting a role in tumor cell adaptation”. We have rephrased the conclusion of this section accordingly.

> Revised Manuscript Line 338: Thus, ZFP36L2 forms dynamic, RNA-dependent biomolecular condensates in tumour tissues and organoids that are stress inducible and highly responsive to environmental cues.

Regarding the apparent discrepancy between images and quantification: the live imaging quantification (**Fig. 4f**) was performed on a single Z-plane of ZFP36L2-GFP–expressing organoids to enable consistent dynamic tracking. In contrast, Fig. 4b shows immunofluorescent staining of endogenous ZFP36L2 in fixed samples, and the supplementary videos display Z-projected whole-mount imaging of organoids. These represent different imaging modalities and sampling strategies. We have now added further detail to the legend to clarify this point.

Together, our data and analysis are consistent with the norms in the field and rigorously demonstrate that ZFP36L2 forms bona fide dynamic condensates in living cells and that their formation is temporally associated with injury-induced state transitions.

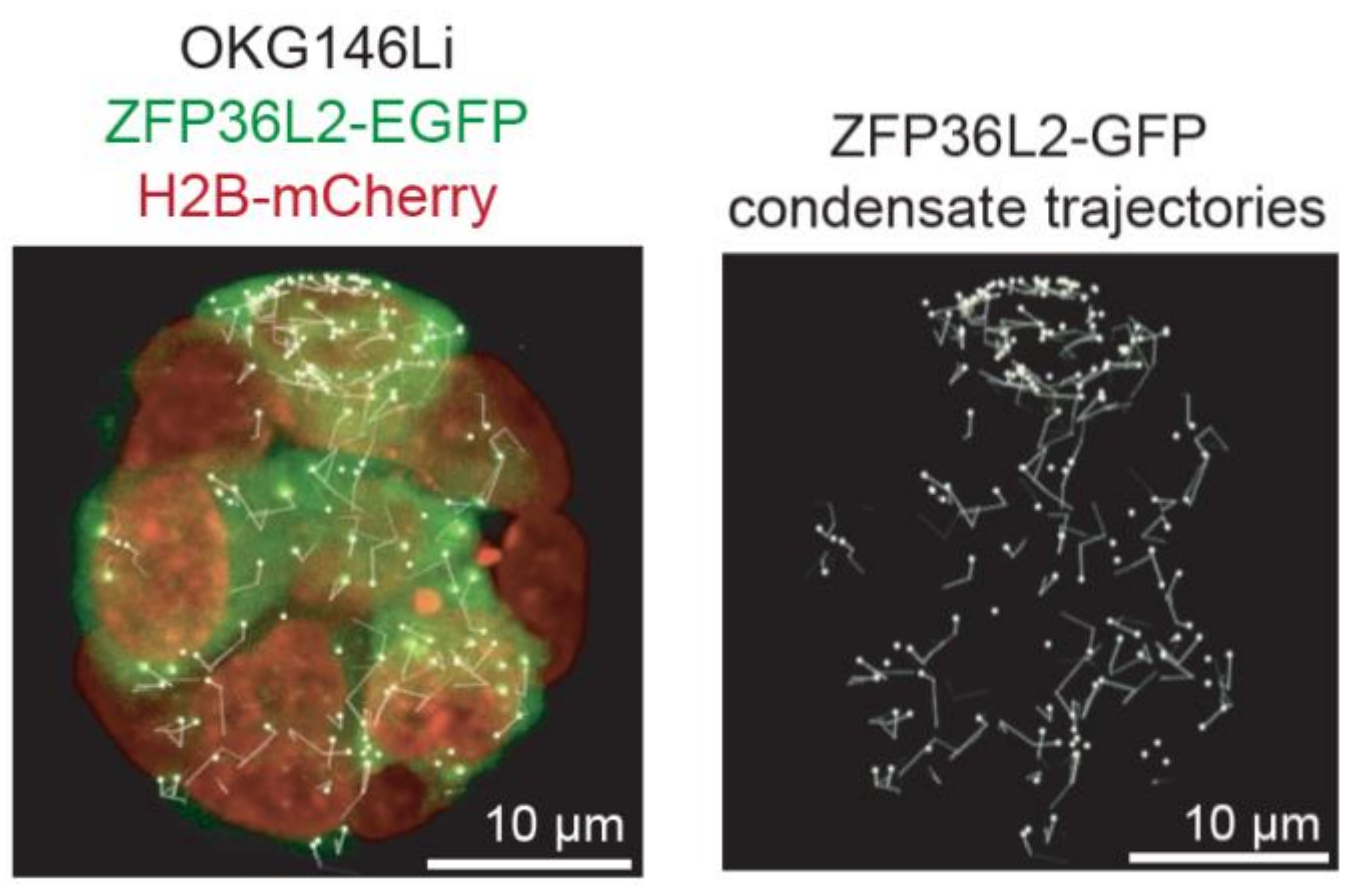

Fig. 4e | Representative images of maximum-intensity projection of a stabilized 3D time-lapse movie of OKG146Li organoids expressing ZFP36L2–EGFP and H2B–mCherry, showing TrackMate-based detection and tracking of ZFP36L2 condensates. Left, condensate positions and trajectories overlaid on the original image. Right, corresponding trajectory rendering shown separately for clarity.

R2.8. Like other claims above, the one that “These data support a role for ZFP36L2 in mediating the termination of the stress response through degradation of stress-associated mRNAs” (lines 315-316) is a big stretch. Huge numbers of transcripts undergo natural decay, and the authors identify putative ZFP36L2 targets using an arbitrary parameter of log(2) 0.5; most “targets” bunch near this cut-off, and a more conservative cut-off would yield few targets (Extended Data Fig. 8d). In Fig. 5c, that cut-off is further reduced to log(2) 0.25, without explanation. At these levels, the risk of noise is substantially elevated and many replicate experiments would be required to distinguish consistent signals from background noise. There simply isn’t enough support for the fanciful model illustrated in Extended Data Fig. 8e.

We thank the reviewer for the careful evaluation of our RNA decay analyses.

(1) We agree that threshold selection must be clearly justified. In the revised manuscript, we have standardized the cutoff in Fig. 5c to $\log_2 FC \geq 0.5$. The prior use of 0.25 was intended for visualization purposes but may have introduced confusion; we have corrected this to maintain a consistent and more conservative threshold throughout (**Fig. 5c**). Importantly, the enrichment analysis shown in Fig. 5d does not depend on an arbitrary fold-change cutoff. The ORA was performed using <u>all</u> statistically significant upregulated genes shared between two independent biological lines (without a fold-change cutoff), each with three technical replicates. This design minimizes random noise and focuses on reproducible ZFP36L2-dependent effects rather than isolated transcript fluctuations.

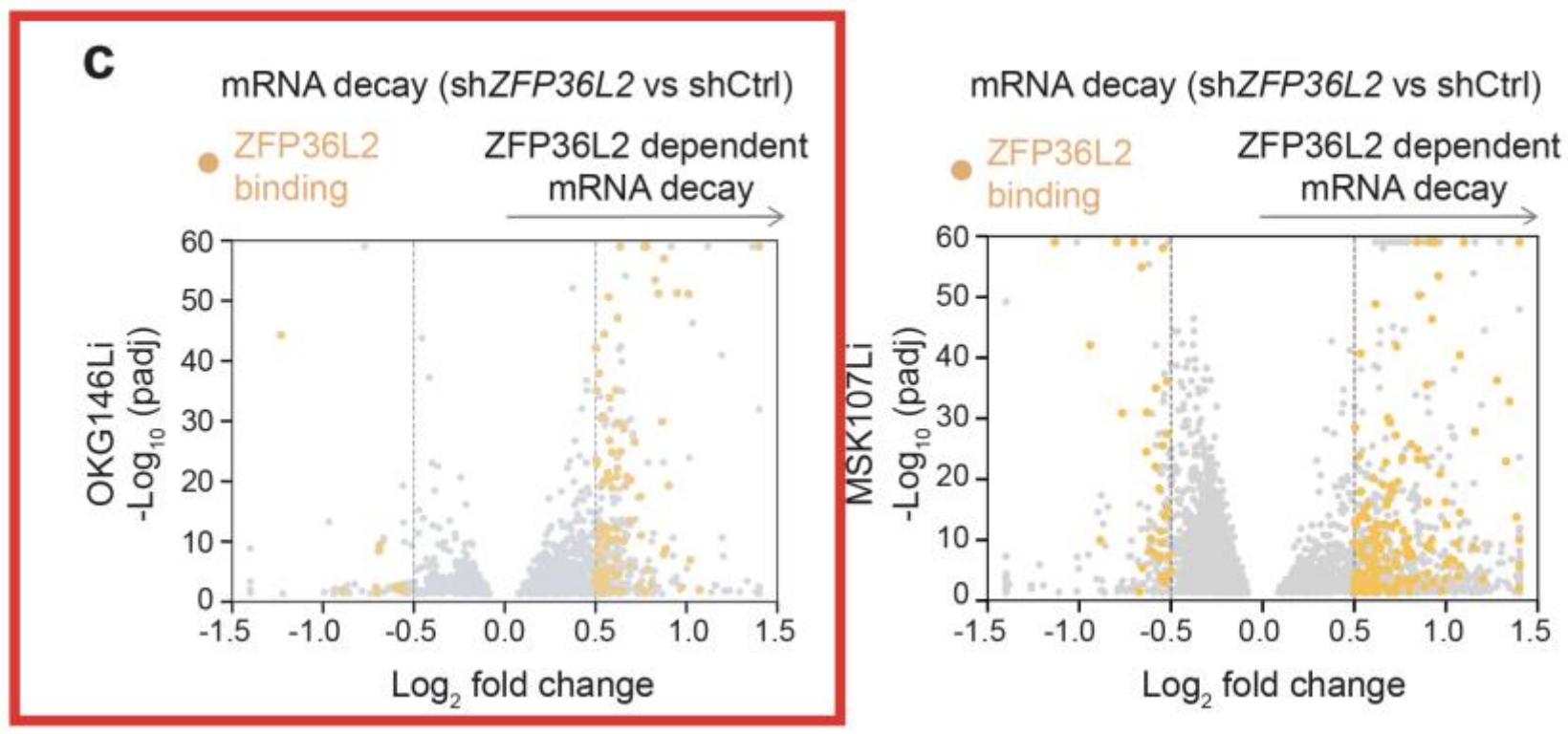

Fig. 5c | Identification of mRNAs that are both bound by ZFP36L2 (HyperTRIBE) and undergo ZFP36L2-dependent mRNA decay (Actinomycin-D (ActD) RNAseq) in OKG146Li (left) and MSK107Li (right). Volcano plots show differential mRNA decay in sh*ZFP36L2* vs shCtrl. Organoids were treated with or without 5 μg/mL ActD for 5 hours and then subjected to RNAseq. Transcriptomes were compared between ActD-treated and untreated conditions to determine mRNA decay rates for shCtrl and sh*ZFP36L2* (see **Methods** for details). ZFP36L2 binding genes shared by two organoid lines (MSK107Li, OKG146Li), identified by hyperTRIBE are highlighted as yellow dots.

(2) We agree with the Reviewer that a large number of cellular transcripts undergo natural decay. This is precisely why we controlled our Actinomycin-D pulse chase assays with shCtrl and sh*ZFP36L2* organoids harvested for RNAseq at the same time points after Actinomycin-D exposure. This experimental design enables us to identify the subset of transcripts whose abundance increases uniquely in the sh*ZFP36L2* condition (i.e. transcripts that undergo decay in a ZFP36L2-dependent manner) (**Extended Data Fig. 11a,b, Supplementary Table 5b**).

(3) To further strengthen the mechanistic basis of our conclusions, we have now incorporated an orthogonal experimental approach to study ZFP36L2-dependent mRNA kinetics using thiol(SH)-linked alkylation for the metabolic sequencing of RNA (SLAM-seq)[36] (**Fig. R8; Fig. 5e,f; Extended Data Fig.11c**), in which newly synthesized RNAs are metabolically labeled and transcript-specific half-lives are directly measured in a time-resolved manner. Consistent with our ActD RNA-seq findings, *ZFP36L2* knockdown increased the half-life of a subset of mRNAs (**Fig. R8a**). Notably, stress-associated GO terms identified using ActD RNA-seq are also significantly enriched among SLAM-seq–validated decay targets (**Fig. R8b**), providing additional independent evidence for a functional role for ZFP36L2 in promoting turnover of stress-responsive transcripts.

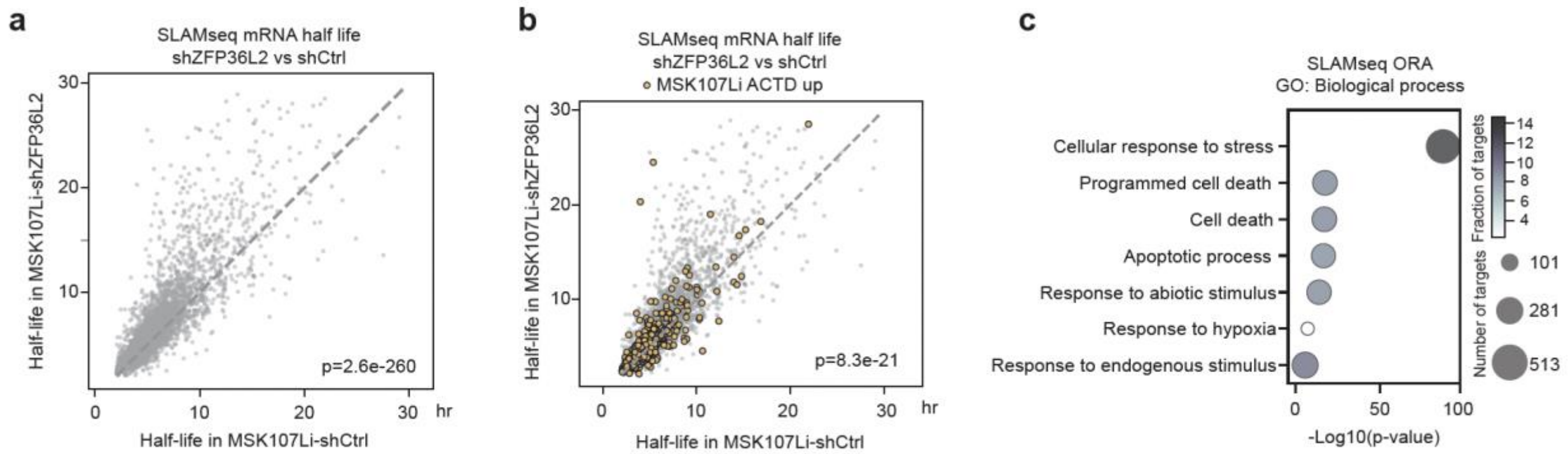

Fig. R8 (Fig. 5e,f; Extended Data Fig.11c) | SLAM-seq identifies ZFP36L2-dependent decay targets enriched for cellular stress-response pathways. a, Scatter plot showing RNA half-lives measured by SLAM-seq in MSK107Li shCtrl and sh*ZFP36L2* organoids. Each dot represents one transcript. The dashed line represents identical half-life between conditions. Transcripts above the diagonal exhibited longer half-lives in sh*ZFP36L2* organoids, consistent with decreased decay following *ZFP36L2* knockdown. Statistical significance was assessed by one-sided Wilcoxon tests. **b,** Transcripts identified as significantly stabilized in the Actinomycin-D decay assay in MSK107Li organoids, highlighted on the SLAM-seq half-life plot, establishing concordance between ActD decay and SLAM-seq assays in identifying ZFP36L2-dependent decay targets. Statistical significance was assessed by one-sided Wilcoxon tests. **c,** GO over-representation analysis of transcripts with significantly increased half-life in sh*ZFP36L2* relative to shCtrl organoids, using GO Biological Process gene sets. The x axis shows the −log10(P−value) of enrichment, dot size indicates the number of target transcripts in each category, and colour indicates the fraction of targets in each term. The same GO terms as in **Fig. 5d** are shown.

Taken together with the half-life measurements, our data establish a coherent regulatory relationship: stress conditions upregulate ZFP36L2 (**Fig. 2c,f; Fig. 3n; Fig. 4f,g; Extended Data Fig. 8g-i**), and ZFP36L2 in turn contributes to attenuation of stress-associated gene expression through post-transcriptional RNA decay (**Fig. 5c-h, Extended Data Fig. 11**), summarized in the model (Extended Data Fig. 11d). While our data identify the central role of ZFP36L2 in the regulation of the stress response, we agree that additional mechanistic details await discovery, and will be the subject of future work in our laboratory and others.

These new data have been added to the revised manuscript (**Fig. 5e,f; Extended Data Fig.11c**). We have added this discussion and clarified the text/legend accordingly (Results, lines 366-371; Methods, lines 1724-1778).

[redacted]

[redacted]

[redacted]

[redacted]

[redacted]

[redacted]

[redacted]

[redacted]

[redacted]

[redacted]

[redacted]

[redacted]

[redacted]

[redacted]

[redacted]

R2.11. Typos: Line 172 should say “MSK125P” and not “OKG125P”, line 184 should say “OKG146Li” and not “KG146Li”.

We have corrected these typos in the revised manuscript.

Reviewer #3

In this study by Jiang and colleagues, the authors investigate the role of ZFP36L2 in intestinal differentiation and stress responses. They use various mouse models and organoids models to show that ZFP is highly expressed in intestinal stem cells, upregulated during stress and that its expression induces a stem cell fate. They then go into the molecular mechanism of ZFP function. They first show that ZFP forms biomolecular condensates, and that such condensates are most prominently observed after stress. They than identify mRNA targets of ZFP and show that such targets include many stress related genes, for which the mRNAs are degraded in a ZFP-dependent manner. [redacted]

Overall, this is a well-written and highly impressive study with an enormous amount of data, which span from mouse models, to sequencing, live-imaging [redacted]. Moreover, the study spans in vivo cancer biology to detailed cellular and molecular mechanisms, which I found highly impressive. Overall, the conclusions are well supported by the data, and the data is well explained. The molecular mechanisms are not very well resolved though, especially the role of ZFP condensates in ZFP function.

We thank the Reviewer for their enthusiastic and highly positive feedback. We appreciate their comment that the overall the conclusions are well supported by the data, and address their specific questions in line below:

R3.1. Specific points While overall the manuscript is well written, I became progressively more lost during the manuscript on the role of stress in the study. In the initial parts of the manuscript, the focus was mainly on ZFP as a stem cell maintenance factor, while in the later parts, ZFP is presented mostly as a regulator of stress, which I found confusing. Are these two different functions, or are they two manifestations of the same function? It would be nice if the reader can be taken along more in the thinking throughout the manuscript in this regard. For example, ZFP is highly expressed in unstressed ISC's, why? Is this related to stress somehow, or unrelated?

R3.5. Hypertribe was performed in CRC cancer organoids instead of in normal organoids. I'm wondering whether the targets identified by these experiments all represent the mRNA targets during normal physiology, or whether a subset of the mRNA targets are cancer specific? Can some of these be validated in non-cancer setting?

We appreciate the reviewer's thoughtful comment regarding the conceptual integration of ZFP36L2's role in stress regulation and stem cell maintenance, and ZFP36L2 targets in normal organoids. Given the overlap between these questions, we answer them together here. We agree that this connection could have been more clearly articulated in the manuscript.

Our data indicate that these are not two unrelated functions, but rather two manifestations of a unified regulatory program. Our HyperTRIBE and RNA decay analyses unexpectedly revealed that ZFP36L2 preferentially associates with and promotes degradation of stress-associated transcripts. Concurrently, we observed that stress stimuli induce ZFP36L2 expression and promote formation of ZFP36L2-containing condensates. These findings led us to define an initial function of ZFP36L2 in attenuating stress-responsive gene expression. [redacted]

[redacted] Based on these data, we propose the following integrated model: stress induces ZFP36L2 expression. ZFP36L2 first attenuates stress-associated transcripts at the post-transcriptional level. Elevated ZFP36L2 levels then contribute to remodeling of transcriptional [redacted] states, [redacted] thereby facilitating dedifferentiation or maintenance of ISC-like states. In this framework, stress regulation and dedifferentiation represent temporally coordinated aspects of a single adaptive response driving the continual replenishment of the ISC pool, rather than independent functions.

Given the scope of the current study, we chose to focus on the role of ZFP36L2 in injury-induced dedifferentiation and regeneration, where its functional requirement is most evident, however, as the Reviewer correctly points out, "ZFP is highly expressed in unstressed ISCs" during intestinal homeostasis (**Fig. 2a,b).** To further delineate the cellular function of ZFP36L2 in the normal colon epithelium, we have now performed HyperTRIBE for wtZFP36L2 vs. fsZFP36L2 in human normal colon organoids (**Fig. R3; Extended Data Fig. 10b-d**). In normal colon organoids, ZFP36L2 target sites were predominantly enriched in 3'UTRs and associated with AU-rich/UAUUUA motifs (**Fig. R3a**), consistent with ZFP36L2 RNA targets in tumor organoids (**Fig. 5a,b**). Importantly, Gene Ontology analysis of ZFP36L2-bound transcripts in normal organoids continued to highlight stress-response–associated programs (**Fig. R3b**), and comparison of ZFP36L2-bound transcripts in normal (OKG146N) vs.tumor (OKG146Li) organoids from the same patient revealed large overlap. Analysis of the genes that were differentially bound in tumor vs. normal revealed that the differences could be largely explained by context-dependent expression of a slightly different subset of transcripts belonging to similar gene programs, rather than a change in binding specificity or cellular function *per se* (i.e., targets detected preferentially in normal were often low/absent in tumor, and vice versa (**Fig. R3c**; **Supplementary Table 5d**).

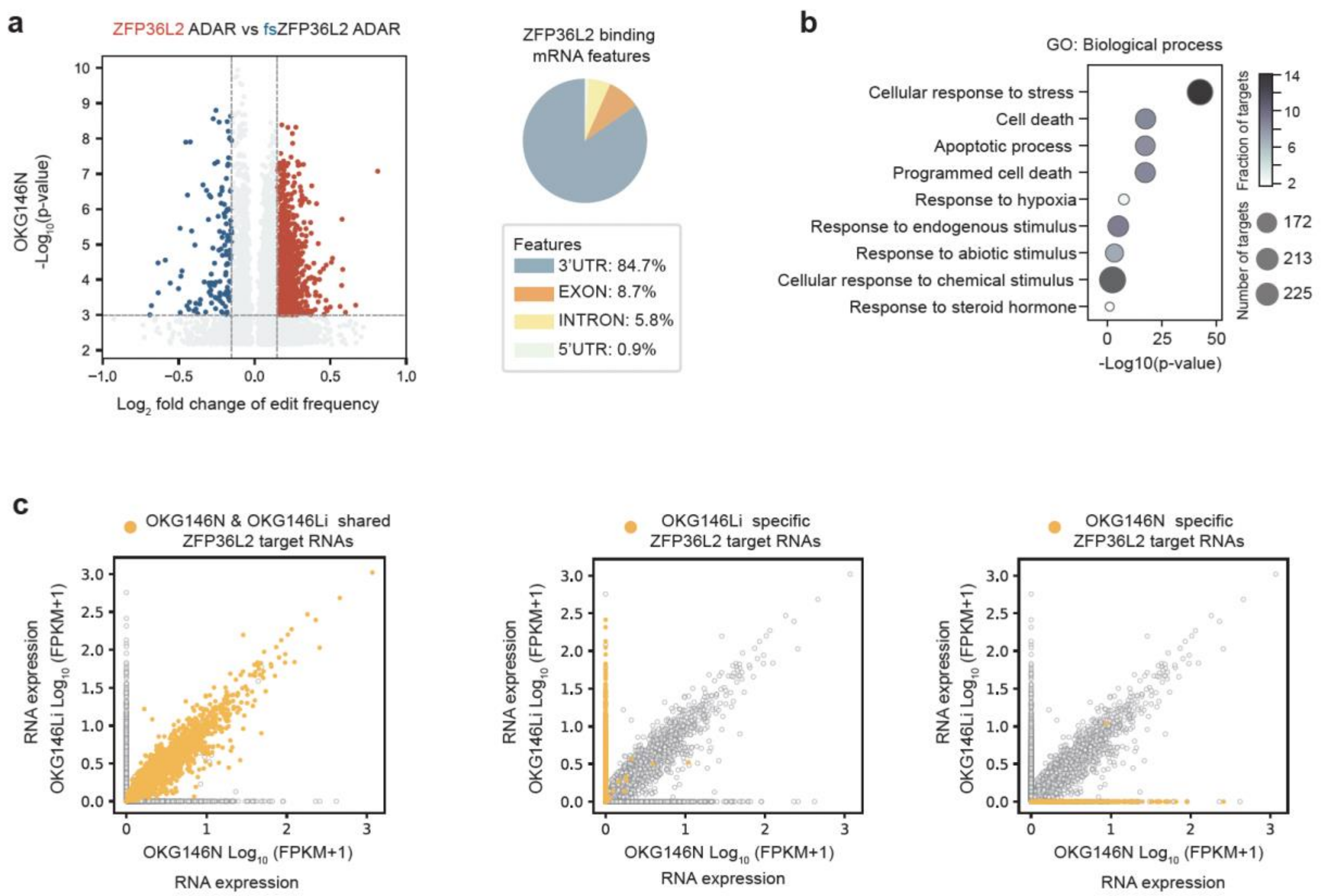

Fig. R3 (Extended Data Fig. 10b-d) | HyperTRIBE profiling of ZFP36L2-bound mRNAs in human normal colon epithelial organoids. a, Volcano plot showing ZFP36L2-bound mRNAs identified by ZFP36L2-ADAR-mediated RNA editing in OKG146N organoids, comparing wild-type ZFP36L2 with frameshift mutant ZFP36L2 (fsZFP36L2). Pie chart showing the genomic distribution of ZFP36L2-specific ADAR editing sites, defined as sites with $-\log_{10}(P\text{-value}) > 3$and $\log_2$fold change in editing frequency > 0.1(wild-type versus fsZFP36L2). **b,** Gene Ontology (GO) enrichment analysis of shared ZFP36L2-bound mRNAs identified in OKG146N, OKG146P and OKG146Li organoids (Revision Table 1b), using GO Biological Process gene sets. The dot plot shows representative GO terms from Fig. 5d. The x axis indicates the $-\log_{10}(P\text{-value})$of enrichment, dot size indicates the number of target genes in each pathway, and colour indicates the fraction of genes enriched in each term. **c,** Scatter plots showing RNA expression in OKG146N and OKG146Li organoids, plotted as $\log_{10}(\mathrm{FPKM}+1)$. Shared ZFP36L2-bound RNAs, as well as OKG146Li-specific and OKG146N-specific ZFP36L2 targets, are highlighted in yellow.

Overall, the consistency in ZFP36L2 mRNA binding in both normal and tumor intestinal epithelial cells further reinforces our model that ZFP36L2 plays a conserved role in epithelial stress sensing, to terminate stress signaling and facilitate regenerative dedifferentiation. Our work identifies that this stress responsive function is active in the normal intestinal epithelium and correlated with the abundance of the ISC population, but is not essential for epithelial function in homeostatic conditions. Instead, the function of ZFP36L2 in stress-responsive ISC pool replenishment becomes critical for epithelial regeneration following injury, and is co-opted to drive tumor regeneration during tumor progression and metastasis.

Regarding the reviewer's question of why ZFP36L2 is highly expressed in unstressed ISCs, we note that intestinal stem cells exist in a poised, injury-responsive state and are highly sensitive to environmental fluctuations including levels of metabolites, cytokines, growth factors derived from the stromal, immune and microbial cell populations of the gut[3–8]. Basal ZFP36L2 expression in ISCs may therefore reflect a preparedness to buffer transient stress-associated transcriptional programs and maintain stem cell identity. Emerging studies[10,11] suggest that intestinal progenitor populations, including isthmus / TA-adjacent progenitors, can functionally replenish ISC pools and display dedifferentiation capacity even under homeostatic steady-state conditions.

We have revised the manuscript to clarify this conceptual link between stress response and maintenance of the ISC pool, and to guide the reader more explicitly through this reasoning. We thank the Reviewer for this insightful line of questioning that has prompted us to further strengthen the conceptual framework and narrative of our study. These new data have been added to the revised manuscript (**Extended Data Fig.10, b-d**).

We have added this discussion and clarified the text/legend accordingly (Discussion, lines 490-499).

R3.2. ZFP36L2 mutations are frequently observed in cancer and the authors suggest that such mutations are likely loss-of-function mutations. In this study the authors use ZFP36L2 knockouts or knockdowns to eliminate the protein entirely. It would be good to know if the mutations indeed are complete loss of function alleles, or whether a truncated protein is expressed. Can they perform a western blot on the N-terminal domain from cancer samples to see if the N-terminus is expressed or not? Alternatively, but less good, they could perform a qPCR to see if the mRNA is lost due to nonsense mediated mRNA decay. If the N-terminus is expressed, it could be nice to see if it acts as a dominant negative in ZFP function.

We appreciate the reviewer's important question regarding whether cancer-associated ZFP36L2 mutations represent complete loss-of-function alleles or produce truncated proteins with residual or dominant-negative activity.

(1) The antibody we used to stain endogenous ZFP36L2 (sc-365908) recognizes amino acids 241-269 of ZFP36L2 (i.e. C-terminal of the zinc fingers (aa 154-180 and 192-217) and should not recognize the N-terminal domain retained in the common clinical frameshift deletion mutants that truncate the protein at aa107 or 144 . Our immunostaining of FFPE tumor tissue from CRC tumors harboring ZFP36L2 frameshift-indel mutations (**Fig. 1f**) shows lack of protein staining, but could potentially miss retained expression of the N-terminal fragment. Unfortunately, we do not have fresh patient tumor samples suitable for Western blot or qPCR analysis of endogenous mutant proteins, and lack an N-terminal recognizing ZFP36L2 antibody that could be used to assess expression.

(2) Directly addressing possible residual or dominant-negative activity of fsZFP36L2, we lentivirally expressed a frameshift ZFP36L2 mutation that recapitulates a recurrent hotspot mutation identified in cancer (G144Afs*43/5rFS*329, **Fig. R10**). This frameshift mutant (fsZFP36L2) is expressed at detectable levels (**Fig. R10a**). However, in contrast to wt ZFP36L2, which induces a slow-cycling state (**Fig. R10b,c**) and induces expression of the injury repair marker L1CAM (**Fig. R10a**), the fsZFP36L2 mutant fails to induce these phenotypes. GFP and

fsZFP36L2 both fail to form condensates and exhibit identical FRAP dynamics (**Fig. 4**, **Extended Data Fig. 8, 9**) with GFP FRAP and is phenotypically indistinguishable from control GFP expressing organoids. These observations argue against a dominant negative phenotype of fsZFP36L2.

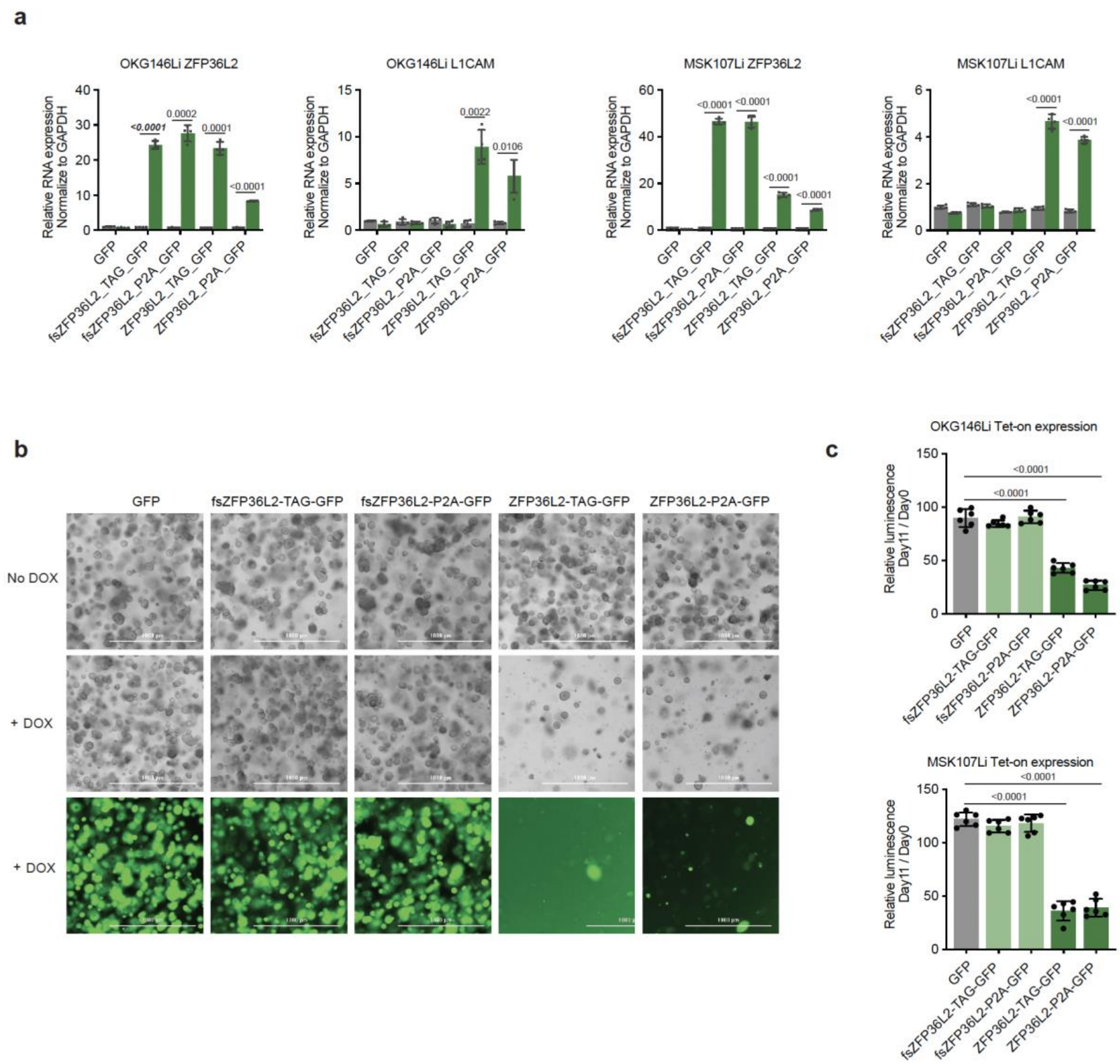

Fig. R7 | Overexpression of *ZFP36L2* traps organoids in a low-cycling injury-repair state. **a**, qPCR analysis of *ZFP36L2* and *L1CAM* expression, normalized to *GAPDH*, in OKG146Li and MSK107Li organoids expressing doxycycline-inducible Tet-on constructs encoding GFP, GFP-tagged ZFP36L2, GFP-tagged frameshift ZFP36L2 (fsZFP36L2), or P2A-GFP versions of ZFP36L2 and fsZFP36L2. Data are mean ± s.d. Two-tailed Mann–Whitney *U* test. **b**,Representative bright-field and GFP images of MSK107Li organoids with indicated construct at day 8. **c**, Cell viability measured by CellTiter-Glo in organoids grown in HISC medium with 2 μg/mL at day 11 and normalized to day 0. Data are mean ± s.d. Two-tailed Mann–Whitney *U* test.

(3) Further, during CRISPR–Cas9-mediated knockout of *ZFP36L2* in organoids, we fortuitously generated a frameshift mutation that preserves the N-terminal region of the protein and lacks

expression of full length wt *ZFP36L2* (**Extended Data Fig. 7**). Importantly, this frameshift clone behaves indistinguishably from complete knockout clones in both organoid growth assays under stem cell–supporting conditions (HISC) and under dedifferentiation-inducing conditions (HISC→IGFF→HISC) (**Extended Data Fig. 7**). While we cannot entirely exclude novel functions of the N-terminus, these results argue against dominant-negative activity of the N-terminal fragment and instead support the interpretation that these mutations disrupt ZFP36L2 function rather than producing an active truncated protein. In future work, development of an antibody recognizing the N-terminus of ZFP36L2 will be necessary to enable specific assessment of the expression of the N-terminal fragment in *fsZFP36L2* mutant tumors. These new data have been added to the revised manuscript (**Extended Data Fig. 7**).

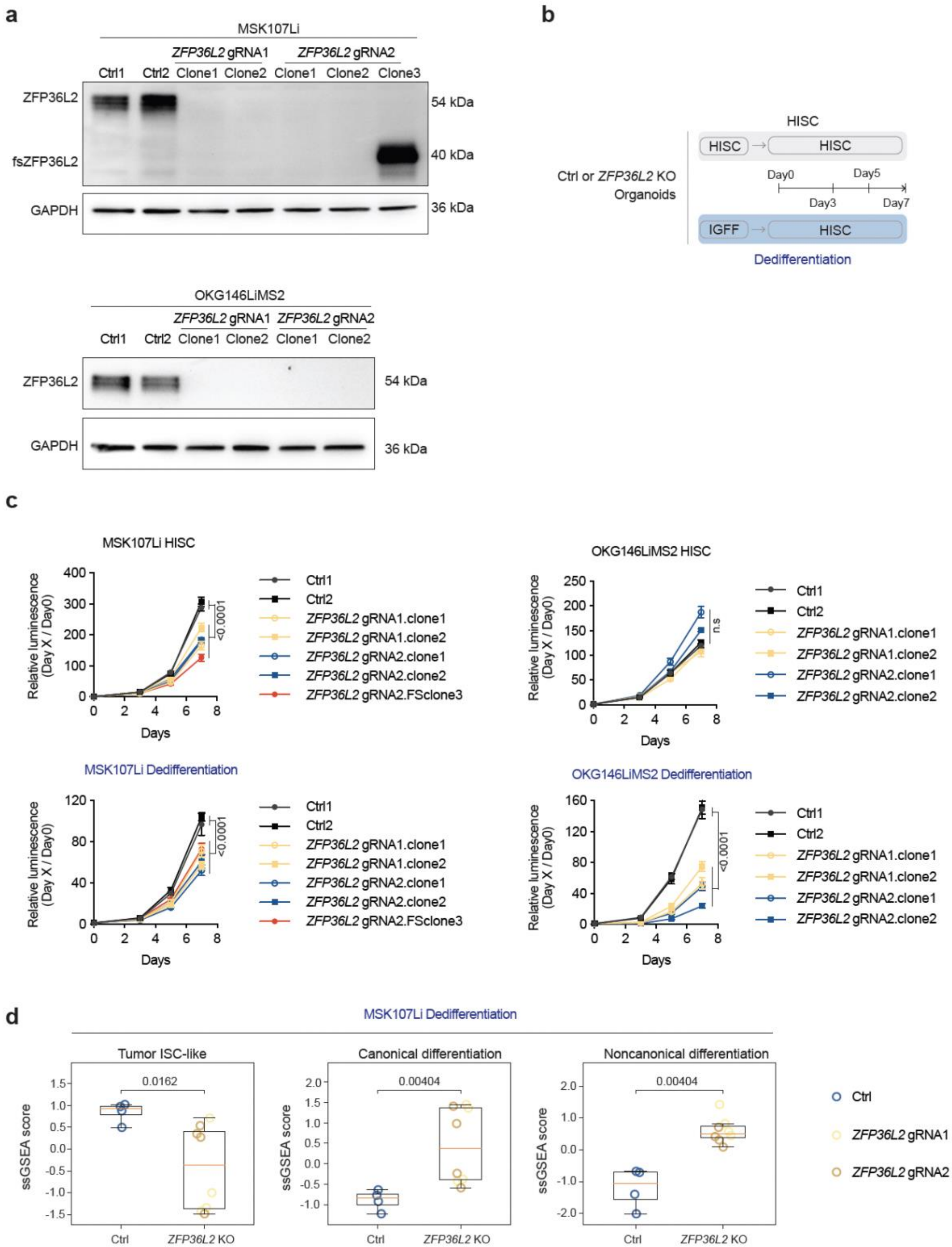

a
MSK107Li
ZFP36L2 gRNA1
ZFP36L2 gRNA2
Ctrl1
Ctrl2
Clone1
Clone2
Clone1
Clone2
Clone3
ZFP36L2
fsZFP36L2
GAPDH
54 kDa
40 kDa
36 kDa
OKG146LiMS2
b
HISC
Ctrl or ZFP36L2 KO Organoids
Day0
Day3
Day5
Day7
IGFF
Dedifferentiation
c
MSK107Li HISC
OKG146LiMS2 HISC
MSK107Li Dedifferentiation
OKG146LiMS2 Dedifferentiation
Relative luminescence (Day X / Day0)
Days
<0.0001
n.s
ZFP36L2 gRNA1.clone1
ZFP36L2 gRNA1.clone2
ZFP36L2 gRNA2.clone1
ZFP36L2 gRNA2.clone2
ZFP36L2 gRNA2.FSclone3
d
MSK107Li Dedifferentiation
Tumor ISC-like
Canonical differentiation
Noncanonical differentiation
ssGSEA score
0.0162
0.00404
0.00404
Ctrl
ZFP36L2 KO
ZFP36L2 gRNA1
ZFP36L2 gRNA2

Extended Data Fig. 7 | *ZFP36L2* knockout inhibits CRC dedifferentiation into an ISC state. a, Western blot validation of ZFP36L2 protein knockout/frameshift indel truncating mutation in MSK107Li and OKG146LiMS2 organoids. Two individual clones for each of two gRNAs targeting the N-terminal region of *ZFP36L2* were analyzed. Organoids cultured in HISC medium for 7 days were harvested for western blotting. **b-d**, Organoid dedifferentiation assay **b**, Schematic of organoid dedifferentiation assay. Organoids were either continuously cultured in HISC medium (HISC group) or cultured in IGFF medium for 13 days, then dissociated into single cells and passaged into HISC medium (Dedifferentiation group), and analyzed at the indicated time points. **c**, Organoid growth measured by CellTiter-Glo viability assays at the indicated time points and normalized to day 0 immediately after plating as single cells (1K cells/ 20 uL Matrigel/well). Data are mean ± s.d. n = 6 replicates/group. Two-tailed Mann–Whitney *U* tests between control and knockout groups. **d**, ssGSEA scores for the indicated gene sets from bulk RNA sequencing of MSK107Li control and *ZFP36L2* knockout organoid lines. Two independent clones from each gRNA were pooled. Organoid on day 7 of dedifferentiation were harvested for bulk RNA sequencing. Gene sets were the same as those used in **Fig. 3j** and **Supplementary Table 2a**. Box plots show the interquartile range (25th to 75th percentiles), horizontal line: median; whiskers: minimum and maximum values. Two-tailed Mann–Whitney *U* tests.

R3.3. Figure 4E. The authors tag ZFP with GFP, but I didn't see which GFP variant it is. A recent study illustrated the importance of the fluorescent protein for condensate formation (doi.org/10.1101/2024.10.04.616694). Can the authors confirm their results with a fluorescent protein that does not perturb condensate formation (or are they already using one)?

We thank the reviewer for raising this important point regarding potential fluorescent protein–driven artifacts in condensate formation. The suggested paper by et al evaluates the role of fluorescent proteins on the formation of a variety of condensates, and concludes that protein of interest, specific condensates and fluorescent protein choice all can contribute to the extent of condensate perturbation, if any. EGFP was shown to not alter stress granule condensate dynamics[42]. In our original submission, we used EGFP to tag ZFP36L2 at the C-terminus. as one of the more appropriate GFP versions for minimizing oligomerization artifacts.

Nevertheless, to rigorously exclude the possibility that condensate formation was influenced by GFP multimerization, we reconstructed the fusion protein using monomeric EGFP (mEGFP), which was also shown to have minimal condensation perturbation[42]. Live-cell imaging of ZFP36L2–mEGFP demonstrated condensate formation with morphology comparable to those observed with the original EGFP construct (**Fig. 4j**). These results with two independent fluorescent proteins, both verified to have low condensate perturbation propensity, further support the conclusion that condensate formation is not driven by GFP-induced oligomerization, but reflects an intrinsic property of ZFP36L2. The new data and representative imaging have been added to the revised manuscript (**Fig. 4j).** We appreciate the Reviewer's helpful suggestion, which has strengthened the rigour of this analysis.

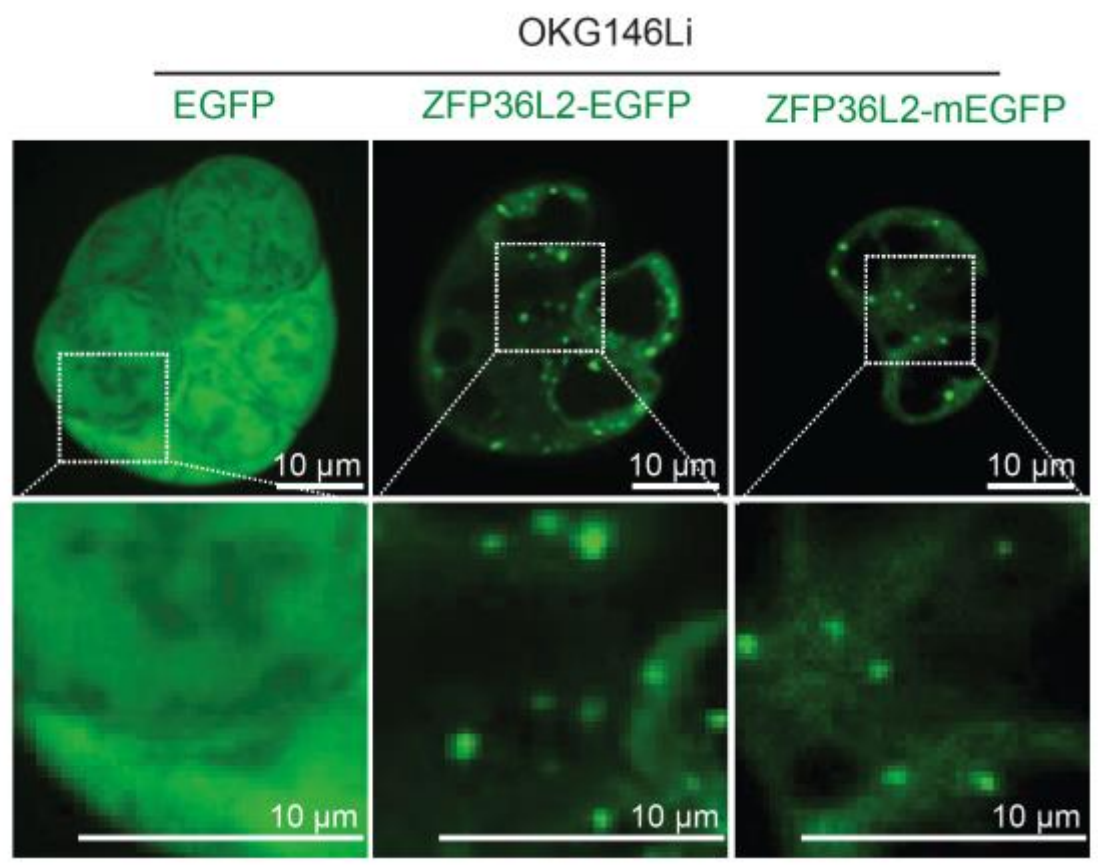

Fig. 4j | Monomeric EGFP-tagged ZFP36L2 forms condensates comparably to EGFP-tagged ZFP36L2. Live-cell imaging of OKG146Li organoids expressing doxycycline-inducible EGFP, ZFP36L2–EGFP or ZFP36L2–mEGFP. Day3 organoids were treated with 2 µg/ml doxycycline for 24 h before imaging. Insets are shown at higher magnification below. ZFP36L2–mEGFP exhibited condensate formation comparable to that of ZFP36L2–EGFP.

R3.4. In figure 4 the authors show that ZFP forms condensates upon a variety of cell stress triggers. At the same time, ZFP levels (at least endogenous levels, unclear for exogenous levels) change. It is well known that condensate formation is highly dependent on protein levels of condensate inducing proteins, so it is possible that condensate formation is simply a consequence of altered ZFP protein levels. To what extent is the formation of ZFP condensates dependent on ZFP protein levels changes (both endogenous and exogenous) during single cell plating or other stressor? Does ZFP form more condensates under stressed conditions, even at similar total expression levels (including endogenous levels)?

We thank the reviewer for raising this important point. We have now performed new experiments to address the question of whether stress-induced ZFP36L2 condensate formation reflects altered protein abundance. As requested, we performed these analyses for both endogenous and exogenous ZFP36L2-GFP, under the same timepoints after single cell seeding used for live cell imaging (**Fig. 4f-g; Extended Data Fig. 8c-f**): i.e., Day 1 (24 hours after seeding), Day 3 and Day 7; treatment with irinotecan for 1 hours, and growth factor starvation by culture in IGFF media for the indicated number of days (**Extended Data Fig. 8g,h**).

(1) For both endogenous and exogenous ZFP36L2, Western blotting revealed that protein levels were elevated on day 1, and further increase on day 3 after single cell seeding. The dynamics of ZFP36L2 expression broadly correlate with the timing of ZFP36L2 condensate formation during early organoid regeneration, although peak condensation occurs on day 1, preceding peak protein levels on day 3 (**Extended Data Fig. 8i**) These data suggest that increased condensate abundance at early time points may partly reflect increased protein abundance.

(2) However, during acute stress treatments (2-hour irinotecan exposure or growth factor starvation), which robustly induce ZFP36L2 condensate formation (**Extended Data Fig. 8i**), total ZFP36L2 protein levels did not increase (Extended Data Fig. 8i). Together, these data suggest

that ZFP36L2 condensate formation is controlled in a multi-step manner. ZFP6L2 protein levels and condensation increase early during organoid regeneration, providing a baseline level of control, but other forms of stress, including chemotherapy and growth factor starvation, can additionally trigger ZFP36L2 condensation independent of ZFP36L2 protein levels. The molecular mechanisms that regulate rapid stress-induced condensate assembly (e.g. through RNA availability or post-translational modification ZFP36L2 or its interaction partners) are of great interest and are the subject of ongoing work in the lab. In future work, delineating tumor-specific mechanisms of ZFP36L2 condensate assembly could provide avenues for therapeutically targeting ZFP36L2-dependent stress adaptive dedifferentiation and tumor regeneration. We have added the new data (**Extended Data Fig. 8i**) and description in the text (Results, lines 316-323).

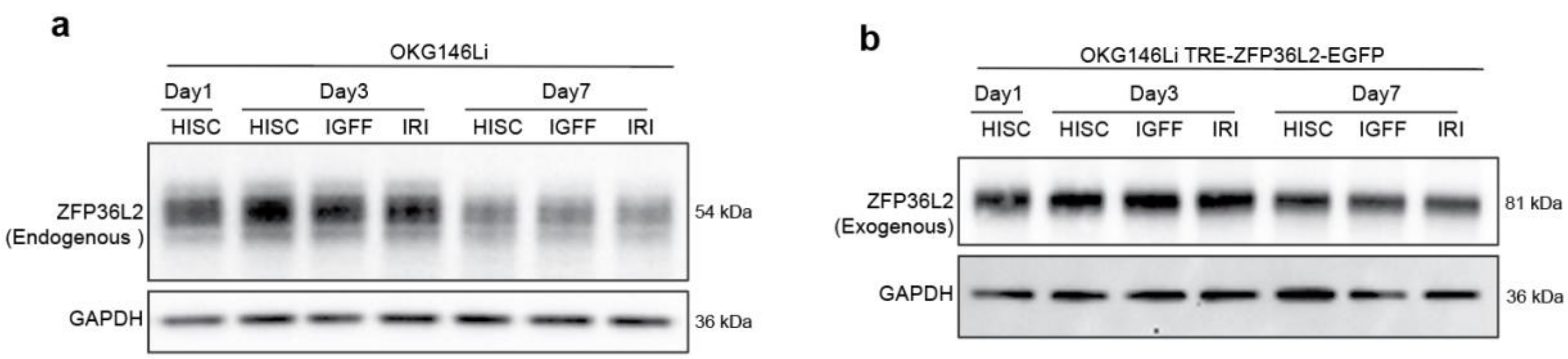

Extended Data Fig. 8i | Distinct contributions of ZFP36L2 protein levels and stressors to condensate formation. a, Western blot analysis of endogenous ZFP36L2 in OKG146Li organoids harvested at day 1, day 3 and day 7 after single-cell plating. Organoids were also exposed to the same conditions used for live-cell imaging shown in **Extended Data Fig 8g**, including 2 h 500 nM irinotecan treatment or growth factor withdrawal in IGFF medium at day 3 or day 7 before collection. **b**, Western blot analysis of exogenous ZFP36L2–EGFP in OKG146Li organoids harvested at the same time points and under the same treatment conditions as in a. Expression of exogenous ZFP36L2–EGFP was induced with doxycycline 24 h before collection.

> Revised manuscript lines 316-323: The level of ZFP36L2 protein increased on days 1-3 of organoid regeneration, and then declined to steady state by day 7 (**Extended Data Fig. 8i)**, while peak condensation occured on day 1, preceding peak protein levels on day 3 (**Fig.4f-g**). However, at each regeneration timepoint, growth factor withdrawal or irinotecan treatment induced condensate formation (**Extended Data Fig. 8g-h**) without changing overall ZFP36L2 protein levels (**Extended Data Fig. 8i**). These observations suggest that while ZFP36L2 protein levels can be one determinant of condensate formation, cellular stresses can rapidly nucleate condensation through protein level-independent mechanisms, potentially through activation of stress signalling pathways or modulation of levels of ZFP36L2-interacting macromolecules.

R3.6. Figure 5C. The authors measure mRNA half-life using actinomycin D. Transcriptional inhibition in this assay likely causes a substantial amount of stress, and ZFP is highly stress responsive, so I don't think this is a good assay. Much better is to use SLAM-seq (or perhaps a PCR-based variation as validation).

R3.6. We thank the Reviewer for this suggestion. To address this concern directly, we performed SLAM-seq as an independent and less stress-inducing approach to measure transcript half-lives without disrupting cellular transcription in the MSK107Li organoids (**Fig. R8**). Following 4sU labeling, we tracked transcripts genome-wide and calculated mRNA half-lives in control versus ZFP36L2 knockdown organoids. Consistent with the Actinomycin-D decay assay, *ZFP36L2* knockdown significantly increased the half-life of transcripts associated with stress-response gene programs. Importantly, transcripts identified as ZFP36L2-dependent decay targets in SLAM-seq experiment were also identified as undergoing ZFP36L2-dependent decay in the Actinomycin-D experiment, thus controlling for and excluding Actinomycin-D induced stress as an experimental confounder. The concordance between the results of the mechanistically distinct SLAMseq and Actinomycin-D mRNA decay experiments, together with the new target mRNA/ZFP36L2 condensate colocalization data shown in **Fig. R9 below** further strengthen and validate the robustness of our conclusions regarding the role of ZFP36L2 in binding and degrading stress response-related transcripts.

These new data have been added to the revised manuscript (**Fig. 5e,f; Extended Data Fig.11c**). We have added this discussion and clarified the text/legend accordingly (Results, lines 366-371; Methods, lines 1724-1778).

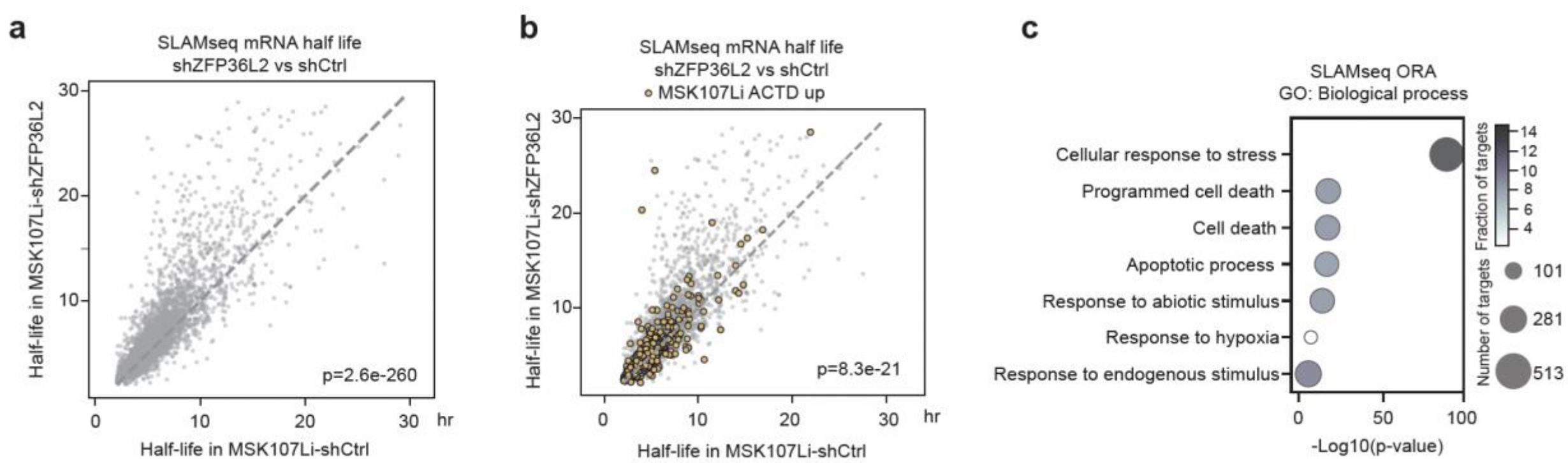

Fig. R8 (Fig. 5e,f; Extended Data Fig.11c) | SLAM-seq identifies ZFP36L2-dependent decay targets enriched for cellular stress-response pathways. a, Scatter plot showing RNA half-lives measured by SLAM-seq in MSK107Li shCtrl and sh*ZFP36L2* organoids. Each dot represents one transcript. The dashed line represents identical half-life between conditions. Transcripts above the diagonal exhibited longer half-lives in sh*ZFP36L2* organoids, consistent with decreased decay following *ZFP36L2* knockdown. Statistical significance was assessed by one-sided Wilcoxon tests. **b,** Transcripts identified as significantly stabilized in the Actinomycin-D decay assay in MSK107Li organoids, highlighted on the SLAM-seq half-life plot, establishing concordance between ActD decay and SLAM-seq assays in identifying ZFP36L2-dependent decay targets. Statistical significance was assessed by one-sided Wilcoxon tests. **c,** GO over-representation analysis of transcripts with significantly increased half-life in sh*ZFP36L2* relative to shCtrl organoids, using GO Biological Process gene sets. The x axis shows the −log10(P−value) of enrichment, dot size indicates the number of target transcripts in each category, and colour indicates the fraction of targets in each term. The same GO terms as in **Fig. 5d** are shown.

R3.7. I find it quite hard to see how good the overlap is in Fig 5C compared to what would be expected by random chance. ZFP binding is seen for both mRNAs that are upregulated and

downregulated mRNAs. Can the authors provide a more quantitative metric for this overlap and enrichment?

To quantitatively assess the enrichment of ZFP36L2-mRNA binding over what would be expected by random chance, we performed an over-representation analysis using all protein-coding genes as the background to test whether ADAR/HyperTRIBE-associated (ZFP36L2-bound) genes are enriched among transcripts showing increased stability upon ZFP36L2 knockdown (i.e., positive decay differences). This analysis revealed a strong and highly significant enrichment in both models: MSK107Li (Fisher's exact test, odds ratio = 2.21, $p = 2.4 \times 10^{-35}$) and OKG146Li (Fisher's exact test, odds ratio = 2.47, $p = 9.2 \times 10^{-75}$). We have added these statistics to the revised legend for **Fig. 5c** (Line 1115-1118)**.**

R3.8. To understand the role of ZFP condensates a bit better, it could be nice to examine whether mRNAs that are degraded by ZFP are enriched in ZFP condensates? And if so, during which phase of stress or repair. This would link condensate formation to ZFP-dependent mRNA decay of its mRNA targets.

To strengthen the connection between ZFP36L2 condensate formation with its mRNA targets, as per the reviewer's suggestion we performed ZFP36L2 IF combined with RNA FISH in OKG146LiMS2 organoids for three stress-associated transcripts identified as ZFP36L2-interacting/decay target mRNAs in our datasets — *GDF15, GADD45A*, and *HILPDA* (**Fig. R9**; **Fig. 5g,h**). We were readily able to detect colocalization of a subset of each of these mRNAs with ZFP36L2 condensates (**Fig. R9a**).

We then quantified mRNA FISH signal within ZFP36L2 puncta/condensate regions across distinct phases of stress and recovery (**Fig. R9b**). Under baseline stem-cell conditions (growth in HISC medium for 7 days), these transcripts showed detectable colocalization with ZFP36L2 puncta. Upon acute short-term (1 hour) exposure to growth factor withdrawal (IGFF medium) or irinotecan —conditions that we have shown to induce rapid ZFP36L2 condensate formation (**Extended Data Fig. 8g,h**)— the percentage of ZFP36L2 condensates colocalizing with mRNA FISH signal was significantly decreased, with this reduction becoming even more pronounced following long-term culture in IGFF media for 10 days. Importantly, passaging organoids that had undergone long-term IGFF culture back into growth factor rich HISC medium, thus alleviating mitogen deprivation-associated stress and inducing dedifferentiation (see **Fig. 3k-n**), induced recovery of the ZFP36L2/mRNA colocalization (**Fig. R9b**).

Together, these data spatiotemporally link ZFP36L2 condensate dynamics with its stress-response mRNA targets: colocalization is detectable at baseline, decreases during acute stress, reaches a minimum under prolonged growth factor stress conditions, and is reversible upon reintroduction of growth factors and is temporally coupled with dedifferentiation into an Lgr5+. ISC state. We have added these new IF–FISH experiments, quantifications, and revised the text accordingly (**Fig. 5g,h;** Results, lines 371-386**).** We thank the reviewer for this excellent suggestion that has strengthened the link between condensate formation. And ZFP-mediated mRNA decay.

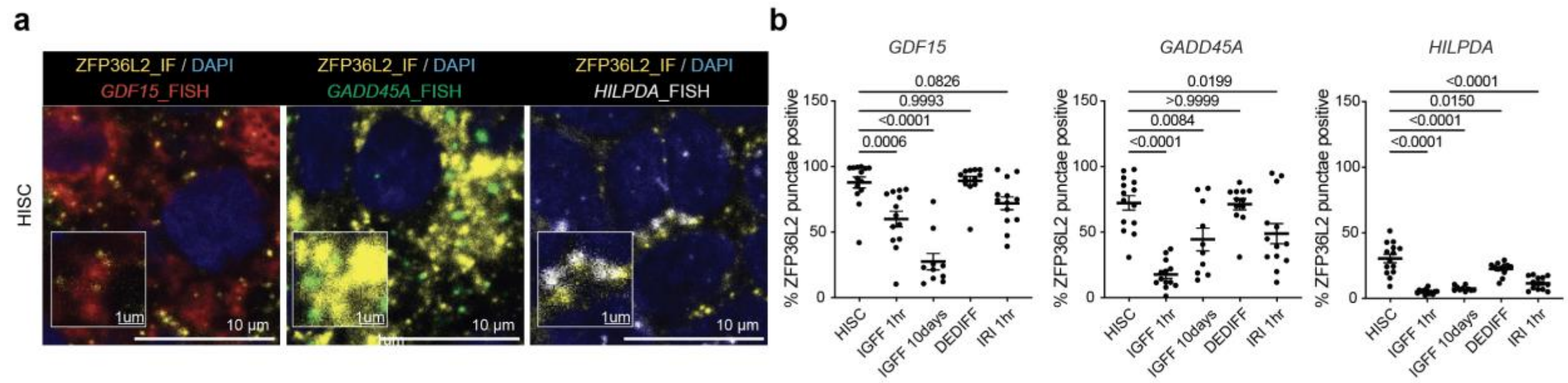

Fig. R9 (Fig. 5g,h) | Colocalization of ZFP36L2 condensates with its target mRNAs. a, Representative images showing colocalization of ZFP36L2 protein with the indicated target RNAs, assessed by ZFP36L2-IF together with RNA FISH for *GDF15, GADD45A, and HILPDA* in OKG146Li-MS2 organoids cultured in HISC medium. **b**, Quantification of the percentage of ZFP36l2 puncta in OKG146Li-MS2 organoids that are also positive for the indicated RNA FISH signal. Prior to fixation, staining and imaging, organoids were cultured in HISC (HISC) media for 7 days, cultured in IGFF media for 10 days prior to switching to HISC media for 7 days to induce dedifferentiation (Dediff), or cultured in HISC media for 7 days prior to switching to IGFF (IGFF 1 hr) or HISC containing 500 μmol irinotecan (IRI 1 hr) for 1 h. Each dot represents one organoid. n = 10-14 organoids per condition. Data are mean ± s.e.m. Two-tailed Mann–Whitney *U* test.

R3.8. It could also be nice to examine if any mRNA decay factors (or P-body markers) co-localize with ZFP condensates, [redacted]

We thank the Referee for this helpful suggestion. Our ZFP36L2 IP-MS data shows that ZFP36L2 pulls down some RNA-decay components also associated with P bodies, but does not pull down classical stress granule components or ribosomal factors (**Extended Data Fig. 12c**, **Supplementary Table 6a**). To validate these protein:protein interactions spatially in the context of ZFP36L2 condensates, we performed co-immunofluorescence (co-IF) for endogenous ZFP36L2 with DDX6, a canonical P-body marker/RNA decay factor required for deadenylation-dependent mRNA decay[43] (**Fig. R10; Extended Data Fig. 12**). We find that around 16% of DDX6 colocalizes with ZFP36L2 condensates, consistent with a context-dependent association between ZFP36L2 condensates and P-body–associated mRNA decay components (**Fig. R10a**). To ascertain whether ZFP36L2 condensates overlap with stress granules, we also examined colocalization of ZFP36L2 with canonical stress granule markers G3BP1 and PABPC1[43]. Under the same conditions where we observed ZFP36L2 condensate and P body formation, we failed to observe the formation of G3BP1/PABPC1 stress granules (**Fig. R10b**), suggesting that ZFP36L2 condensates are distinct from stress granules.

We have added these new data (**Extended Data Fig. 12d,e**), and revised the text accordingly (Results, lines 416-419).

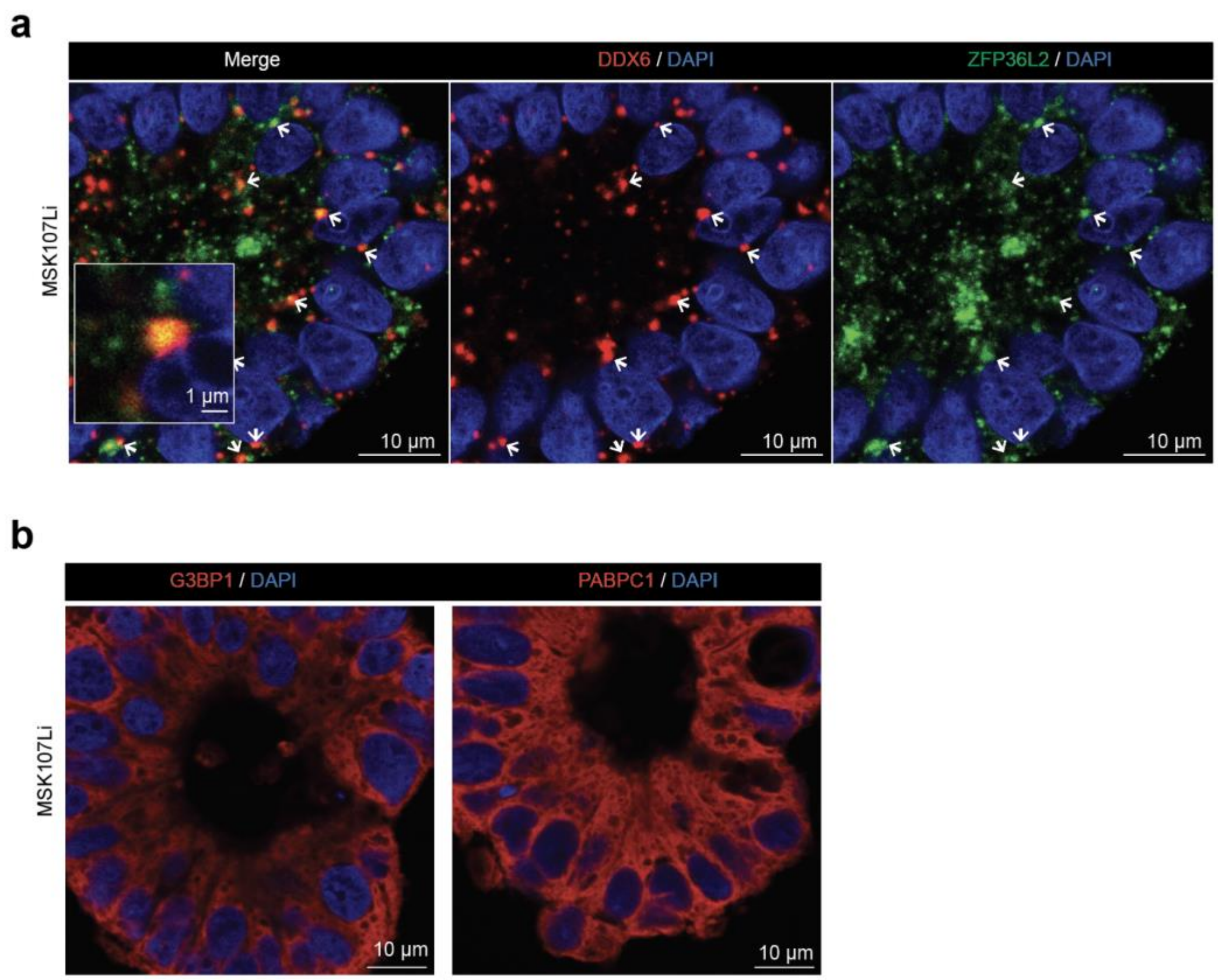

Fig. R10 (Extended Data Fig. 12d,e) | ZFP36L2 puncta partially colocalize with the P-body marker DDX6. a, Representative images of dual immunofluorescence staining for P-body marker DDX6 and ZFP36L2 in MSK107Li organoids 1 h after growth factor withdrawal, showing partial colocalization between DDX6 and ZFP36L2 puncta. Arrows indicate representative colocalized puncta. Inset shows a higher-magnification view. **b**, Representative images of stress granule markers G3BP1 and PABPC1 immunostaining in MSK107Li organoids 1 h after growth factor withdrawal. G3BP1 and PABPC1 failed to form condensates under these conditions.

[redacted]

[redacted]

[redacted]

[redacted]

[redacted]

[redacted]

[redacted]

[redacted]

[redacted]

[redacted]

[redacted]

R3.10. [redacted]

In our original manuscript, we were careful not to claim to have demonstrated specific functions of ZFP36L2 condensates, as opposed to showing separately the functions of the ZFP36L2 protein, and that ZFP36L2 forms condensates. However, we appreciate the reviewer's feedback that our unclear wording in the original manuscript. Because we did not directly disentangle the role of ZFP36L2 from ZFP36L2 condensates, we have revised the Results and Discussion to more explicitly avoid implying that condensates are required [redacted] We now state more clearly and conservatively in the Discussion that ZFP36L2 forms RNA-dependent condensates [redacted], while the requirement of condensate material properties for these interactions remains to be established.

> Revised manuscript lines 499-502: While this study shows that ZFP36L2 can form [redacted] condensates, the extent to which [redacted] functions of ZFP36L2 are mediated by condensate formation *per se*, as opposed to condensate independent functions of ZFP36L2, remain unresolved.

We agree with the reviewer that it is experimentally challenging to selectively perturb condensate material properties without altering other functions of ZFP36L2. We thank the reviewer for their excellent suggestion to try the recently published condensate "kill switch" micropeptide system (Zhang et al., Nature, 2025)[44]. In this system, GFP fusion proteins are targeted by an anti-GFP nanobody fused with two copies of a 17 amino acid, hydrophobic and aromatic residue-rich micropeptide derived from HMGB1 (the "killswitch"), which was reported to reduce protein mobility within targeted condensates, and thus alter either condensate formation (by light microscopy) or dynamics (by FRAP) of some nuclear and fusion oncoproteins. The authors of this study proposed that this GFP-targeted killswitch system could be used broadly as a strategy to separate condensate-dependent functions from those of the target protein *per se*.

Accordingly, we engineered OKG146Li organoids expressing either ZFP36L2-GFP or GFP to co-express either control anti-GFP nanobody (GFP-nb) or anti-GFP nanobody-2xKillSwitch (GFP-nb-2xKS) (**Fig. R10a**). In our system, however, recruitment of 2× killswitch to ZFP36L2–GFP condensates did not measurably impair condensate formation or dynamics (**Fig. R10a**). FRAP analysis showed that ZFP36L2–GFP condensates remained highly dynamic in the presence of 2× killswitch, with recovery behavior comparable to both a nanobody control and our prior ZFP36L2–GFP measurements (**Fig. R10b**). Thus, under our experimental conditions, the

killswitch approach did not provide a means to selectively “harden” ZFP36L2 condensates, limiting its utility for testing whether condensate material properties are required for protein–protein interactions or downstream phenotypes.

Consistent with this interpretation, the 2× killswitch did not rescue the reduced growth phenotype associated with high ZFP36L2 expression (**Fig. R10c**). Moreover, we observed that 2× killswitch could modestly accelerate organoid growth in both ZFP36L2–GFP and GFP-only contexts ((**Fig. R10c**), suggesting context-independent effects that complicate functional interpretation in our system. Zhang et al., primarily tested the killswitch (itself derived from a nucleolar protein HMGB1) against nuclear condensates, while ZFP36L2 condensates are largely cytoplasmic; it is possible that differences in the subcellular context including the local chemical environment of these condensates may contribute to the lack of killswitch efficacy here, but discerning this is beyond the scope of the current study. Given these outcomes, we did not pursue killswitch-based perturbations further.

Instead, to enable rigorous tests of condensate requirement, a structure–function (separation-of-function) approach—for example, systematic mutagenesis to identify variants that disrupt condensate properties while preserving RNA binding/other core activities—may be required. In the revised manuscript, we therefore tone down statements regarding condensates mediating protein–protein interactions and frame this question as an important direction for future work. We are grateful to the reviewer for prompting us to clarify this important point.

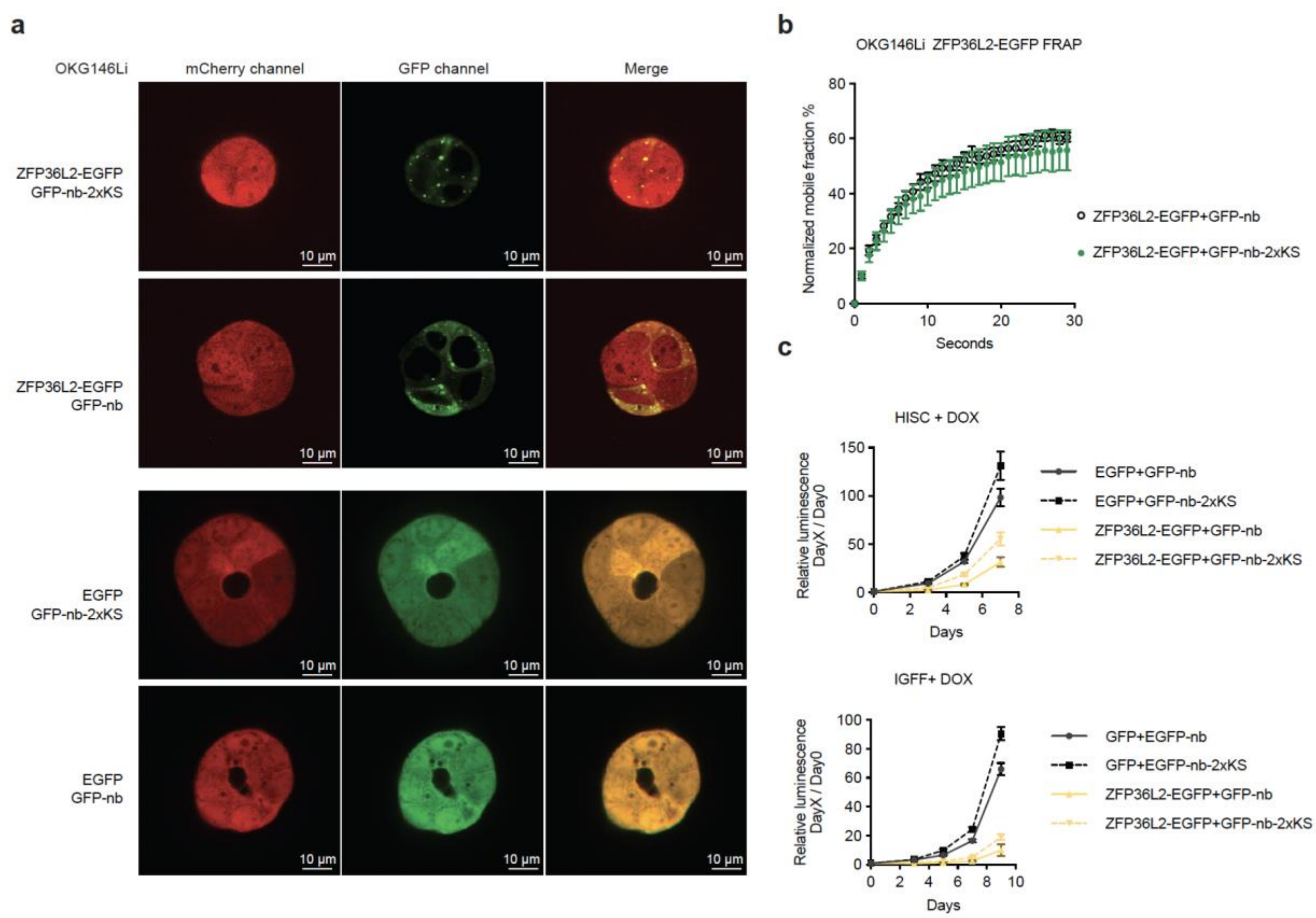

Fig. R10 | The GFP nanobody-based killswitch system does not perturb ZFP36L2 condensates. a,

Representative images of OKG146Li organoids expressing doxycycline-inducible ZFP36L2–EGFP or EGFP together with GFP-nb-2xKS or GFP-nb. All constructs were introduced by lentiviral integration. Expression was induced in day 3 organoids with doxycycline for 24 h before imaging. **b**, FRAP analysis of ZFP36L2–EGFP condensates in organoids expressing GFP-nb or GFP-nb-2xKS. n=9 bleached condensates. Data are mean ± s.e.m. **c**, Growth curves of OKG146Li organoids expressing the indicated construct combinations. Growth was measured by CellTiter-Glo at the indicated time points and normalized to day 0 immediately after plating. Organoids were cultured in HISC or IGFF medium with 2 µg /mL doxycycline.

Reviewer #4

I co-reviewed this manuscript with one of the reviewers who provided the listed reports.

References

1. Moorman, A. *et al.* Progressive plasticity during colorectal cancer metastasis. *Nature* **637**, 947–954 (2025).
2. Nunes, L. *et al.* Prognostic genome and transcriptome signatures in colorectal cancers. *Nature* **633**, 137–146 (2024).
3. Hageman, J. H. *et al.* Intestinal Regeneration: Regulation by the Microenvironment. *Developmental Cell* **54**, 435–446 (2020).
4. Choi, J. & Augenlicht, L. H. Intestinal stem cells: guardians of homeostasis in health and aging amid environmental challenges. *Exp Mol Med* **56**, 495–500 (2024).
5. Kaiko, G. E. *et al.* The Colonic Crypt Protects Stem Cells from Microbiota-Derived Metabolites. *Cell* **165**, 1708–1720 (2016).
6. Biton, M. *et al.* T Helper Cell Cytokines Modulate Intestinal Stem Cell Renewal and Differentiation. *Cell* **175**, 1307-1320.e22 (2018).
7. Lindemans, C. A. *et al.* Interleukin-22 promotes intestinal-stem-cell-mediated epithelial regeneration. *Nature* **528**, 560–564 (2015).
8. Goto, N. *et al.* Lymphatics and fibroblasts support intestinal stem cells in homeostasis and injury. *Cell Stem Cell* **29**, 1246-1261.e6 (2022).
9. Islam, M. *et al.* Unbiased recording of clonal potency reveals species-specific regulation of mammalian intestine. *bioRxiv* 2025.06.10.658707 (2025) doi:10.1101/2025.06.10.658707.
10. Malagola, E. *et al.* Isthmus progenitor cells contribute to homeostatic cellular turnover and support regeneration following intestinal injury. *Cell* **187**, 3056-3071.e17 (2024).
11. Capdevila, C. *et al.* Time-resolved fate mapping identifies the intestinal upper crypt zone as an origin of Lgr5+ crypt base columnar cells. *Cell* **187**, 3039-3055.e14 (2024).
12. Yan, K. S. *et al.* Intestinal Enteroendocrine Lineage Cells Possess Homeostatic and Injury-Inducible Stem Cell Activity. *Cell Stem Cell* **21**, 78-90.e6 (2017).
13. van Es, J. H. *et al.* Dll1+ secretory progenitor cells revert to stem cells upon crypt damage. *Nat Cell Biol* **14**, 1099–1104 (2012).
14. Buczacki, S. J. A. *et al.* Intestinal label-retaining cells are secretory precursors expressing Lgr5. *Nature* **495**, 65–69 (2013).

15. Tetteh, P. W. *et al.* Replacement of Lost *Lgr5*-Positive Stem Cells through Plasticity of Their Enterocyte-Lineage Daughters. *Cell Stem Cell* **18**, 203–213 (2016).
16. Yu, S. *et al.* Paneth Cell Multipotency Induced by Notch Activation following Injury. *Cell Stem Cell* **23**, 46-59.e5 (2018).
17. Castillo-Azofeifa, D. *et al.* Atoh1+ secretory progenitors possess renewal capacity independent of Lgr5+ cells during colonic regeneration. *EMBO J* **38**, e99984 (2019).
18. Murata, K. *et al.* Ascl2-Dependent Cell Dedifferentiation Drives Regeneration of Ablated Intestinal Stem Cells. *Cell Stem Cell* **26**, 377-390.e6 (2020).
19. Viragova, S., Li, D. & Klein, O. D. Activation of fetal-like molecular programs during regeneration in the intestine and beyond. *Cell Stem Cell* **31**, 949–960 (2024).
20. Tian, H. *et al.* A reserve stem cell population in small intestine renders Lgr5-positive cells dispensable. *Nature* **478**, 255–259 (2011).
21. de Sousa e Melo, F. & de Sauvage, F. J. Cellular Plasticity in Intestinal Homeostasis and Disease. *Cell Stem Cell* **24**, 54–64 (2019).
22. Higa, T. *et al.* Spatiotemporal reprogramming of differentiated cells underlies regeneration and neoplasia in the intestinal epithelium. *Nat Commun* **13**, 1500 (2022).
23. Jadhav, U. *et al.* Dynamic Reorganization of Chromatin Accessibility Signatures during Dedifferentiation of Secretory Precursors into Lgr5+ Intestinal Stem Cells. *Cell Stem Cell* **21**, 65-77.e5 (2017).
24. Jones, J. C. *et al.* Cellular Plasticity of Defa4Cre-Expressing Paneth Cells in Response to Notch Activation and Intestinal Injury. *Cell Mol Gastroenterol Hepatol* **7**, 533–554 (2019).
25. Huang, L. *et al.* Tuft cells act as regenerative stem cells in the human intestine. *Nature* **634**, 929–935 (2024).
26. Ayyaz, A. *et al.* Single-cell transcriptomes of the regenerating intestine reveal a revival stem cell. *Nature* **569**, 121–125 (2019).
27. Gerstberger, S., Jiang, Q. & Ganesh, K. Metastasis. *Cell* **186**, 1564–1579 (2023).
28. Qin, X. *et al.* An oncogenic phenoscape of colonic stem cell polarization. *Cell* **186**, 5554-5568.e18 (2023).
29. Ogden, S. *et al.* Phenotypic heterogeneity and plasticity in colorectal cancer metastasis. *Cell Genom* 100881 (2025) doi:10.1016/j.xgen.2025.100881.
30. Mzoughi, S. *et al.* Oncofetal reprogramming drives phenotypic plasticity in WNT-dependent colorectal cancer. *Nat Genet* **57**, 402–412 (2025).
31. Ayyaz, A. *et al.* Single-cell transcriptomes of the regenerating intestine reveal a revival stem cell. *Nature* **569**, 121–125 (2019).
32. Ganesh, K. *et al.* L1CAM defines the regenerative origin of metastasis-initiating cells in colorectal cancer. *Nat Cancer* **1**, 28–45 (2020).
33. Cañellas-Socias, A. *et al.* Metastatic recurrence in colorectal cancer arises from residual EMP1+ cells. *Nature* 1–11 (2022) doi:10.1038/s41586-022-05402-9.
34. Park, H. S., Goodlad, R. A. & Wright, N. A. Crypt fission in the small intestine and colon. A mechanism for the emergence of G6PD locus-mutated crypts after treatment with mutagens. *Am J Pathol* **147**, 1416–1427 (1995).

35. Miyoshi, H., Ajima, R., Luo, C. T., Yamaguchi, T. P. & Stappenbeck, T. S. Wnt5a potentiates TGF-β signaling to promote colonic crypt regeneration after tissue injury. *Science* **338**, 108–113 (2012).

36. Herzog, V. A. *et al.* Thiol-linked alkylation of RNA to assess expression dynamics. *Nat Methods* **14**, 1198–1204 (2017).

37. Bestehorn, A. *et al.* Cytoplasmic mRNA decay controlling inflammatory gene expression is determined by pre-mRNA fate decision. *Molecular Cell* **85**, 742-755.e9 (2025).

38. Yang, L. *et al.* Tissue-location-specific transcription programs drive tumor dependencies in colon cancer. *Nat Commun* **15**, 1384 (2024).

39. Stringer, E. J. *et al.* Cdx2 determines the fate of postnatal intestinal endoderm. *Development* **139**, 465–474 (2012).

40. Gao, N., White, P. & Kaestner, K. H. Establishment of Intestinal Identity and Epithelial-Mesenchymal Signaling by Cdx2. *Developmental Cell* **16**, 588–599 (2009).

41. Kumar, N. *et al.* The lineage-specific transcription factor CDX2 navigates dynamic chromatin to control distinct stages of intestine development. *Development* **146**, dev172189 (2019).

42. Dörner, K. *et al.* Fluorescent protein and peptide tags alter condensate formation and dynamics in vivo and in vitro. *EMBO Rep* **27**, 89–121 (2026).

43. Riggs, C. L., Kedersha, N., Ivanov, P. & Anderson, P. Mammalian stress granules and P bodies at a glance. *J Cell Sci* **133**, jcs242487 (2020).

44. Zhang, Y. *et al.* Probing condensate microenvironments with a micropeptide killswitch. *Nature* **643**, 1107–1116 (2025).

Response to Reviewers

We thank the Editor and the Reviewers for their thoughtful and constructive feedback on our revised manuscript. We are encouraged that the reviewers found the additional data and clarifications provided in the first revision to be comprehensive and impactful.

[redacted] By focusing the manuscript on the robust functional and biochemical evidence for ZFP36L2's role in stress-adaptive plasticity and cytoplasmic mRNA decay (Figures 1–5), we believe the core message of the study is now more cohesive and strongly supported.

Below, we address the remaining specific concerns, with a detailed emphasis on the Figure 3 data.

Referees' comments:

Referee #1 (Remarks to the Author):

The authors have provided further clarity in the presentation of their data in the revised manuscript in response to the suggestions of the reviewers. A highly comprehensive point by point response to all the queries has been provided. The authors have also conducted numerous additional experiments to either reinforce, add quantitative data or provide further insight to their study. Numerous amendments to the text, Figures and Figure legends have been provided. **The authors have adequately addressed the reviewer's comments and provide a highly original and significant manuscript supported by quality data and robust discussion.**

We thank the reviewer for their positive assessment and for recognizing the significance of our findings.

Referee #2

My original review raised concerns about this study in 5 broad categories, and the authors have addressed each of these concerns in considerable detail. My detailed review follows. Briefly, **my concerns in categories 2, 4, and parts of 5 are thoroughly addressed for the most part, though additional small wording changes would improve the manuscript further. My persistent concerns in categories 1 and 3 can be addressed by changes to the text and/or figures without additional experiments.** [redacted]

1. Excerpts from my original review include “ZFP36L2 seems to be expressed in many crypt epithelial (except the most mature) and almost all tumour cells”; “In summary, Fig. 1 and Extended Data Figs. 1 and 2 seem intended to defend interest in ZFP36L2 and build a connection to AP-1 complexes, but the data are murky and don’t support the implied conclusions”; and “Mutation in 5-10% of CRCs is arguably sufficient justification to study ZFP36L2 functions”. As these remarks indicate, I understand that these data do not address mechanism and simply set the stage to investigate ZFP36L2 functions.

The authors agree that “these figures were not designed to establish mechanistic conclusions, but rather to elaborate hypotheses” (rebuttal, p. 20 bottom). Data in Fig. 1 and Extended Data Fig. 1c show ~2-fold range in ZFP36L2 mRNA between normal human intestinal stem (ISCs) and terminally differentiated cells. In mouse colonic crypts, ZFP36L2 is expressed in most (all?) TA cells (Fig. 2b) and the idea that this is because they “exist in a poised, injury-responsive state” makes little sense. The leap from ZFP36L2 expression to a “regenerative” cell state (especially in normal epithelium) or to “metastasis seeding markers L1CAM and EMP1” (Extended Data Fig. 2; P values are less useful here than observing the shape of violin plots) remains tenuous. These are conceptual weaknesses of the study but not fatal flaws. In my opinion, originally and now, the presence of ZFP36L2 mutations in up to 10% of colorectal cancers (CRCs) adequately justifies studying its functions.

We appreciate the reviewer's perspective but respectfully disagree with the Reviewer's interpretation that the link between ZFP36L2 expression, ISC/TA states and regenerative programs remains tenuous.

We agree that the presence of ZFP36L2 mutations in up to 10% of CRCs is an important initial rationale for investigating this gene. However, the motivation for the current study was not based on mutation frequency alone, but also on unbiased analyses of human CRC and matched normal epithelial scRNA-seq datasets, which consistently associated ZFP36L2 with ISC/TA-related and injury/regeneration-linked transcriptional programs. Indeed, these observations were not intended to establish mechanism by themselves, but they provided a biologically informative framework (which is important) for the functional experiments that follow.

First, the association between ZFP36L2 and ISC/TA or AP-1-associated programs was not imposed by our model, nor was it inferred from selected marker genes. These relationships emerged from unbiased, gene-expression-based analyses across large human CRC datasets. In particular, ZFP36L2 expression covaried with tumour-intrinsic ISC-like gene modules and with AP-1-associated transcriptional programs. We consider this convergence **biologically meaningful** because ISC-like programs and AP-1-linked injury/regenerative programs represent distinct but functionally connected states during epithelial repair and tumour regeneration. Thus, the observation that ZFP36L2 is associated with both programs was not a trivial consequence of broad crypt expression, but a novel finding that helped explain the phenotypes later observed in regeneration, dedifferentiation and metastasis assays.

ZFP36L2 is consistently enriched in ISC-like and TA populations in both human and mouse colon epithelium. We agree that ZFP36L2 is expressed in TA cells in normal human and mouse colonic crypts. Our interpretation is not that every TA cell is actively regenerative or metastatic, but rather that proliferative crypt cells, including TA cells, are among the epithelial compartments competent to respond to injury and participate in regeneration. In this context, ZFP36L2 expression in ISC/TA populations is consistent with a role in enabling epithelial state transitions under stress, rather than marking a fixed "regenerative cell state" at homeostasis. This distinction is important: our central claim is not that ZFP36L2 expression alone defines a regenerative state, but that ZFP36L2 function becomes critical when epithelial cells are challenged to regenerate, dedifferentiate or seed metastases. This interpretation is directly supported by the subsequent in vivo and organoid-based functional experiments.

Regarding L1CAM and EMP1, we agree with the Reviewer that these markers are expressed in only a small fraction of tumour cells. However, this rarity is precisely why violin plots can visually underrepresent the relationship: the large number of marker-negative cells compresses the distribution, making the positive population difficult to appreciate from the overall violin shape. When cells positive for L1CAM, EMP1 or the injury-repair signature are examined directly, the vast majority are ZFP36L2-high. Thus, **although L1CAM/EMP1-positive cells represent a small subset of tumour cells, they are strongly enriched within the ZFP36L2-high compartment.**

Together, these data do not claim that ZFP36L2 expression alone establishes a regenerative or metastatic cell identity. Rather, they show that ZFP36L2 is positioned at the intersection of CRC genetics, ISC/TA-associated epithelial programs and injury-linked AP-1/regenerative states. This unbiased transcriptional association is important since it directly leads to the hypothesis that ZFP36L2 regulates epithelial plasticity, which we then test experimentally throughout the remainder of the manuscript.

We agree with the Reviewer that the statement that the barrier epithelium of the gut "exists in a poised, injury-responsive state" is somewhat speculative, and have removed this phrasing from the discussion.

2. Technical problems with experiments on ZFP36L2 requirement in intestinal regeneration. **All my concerns are addressed thoroughly.**

(a) DSS colitis usually occurs in patches. The authors clarify that their protocol with 3.5% DSS induces uniform damage. Moreover, the authors now support their arguments with another injury model (Lgr5-DTR mice).

One remaining small point. If the authors agree that "the first two images in Fig. 2c appear similar to control," then why claim that "ZFP36L2 expression was markedly increased by day 2 …" (line 117)? Even Suppl. Data 1 and 3 don't support that claim. Moreover, the claim doesn't help or hurt the authors' central arguments.

We appreciate that our additional data have addressed the reviewer's concerns thoroughly. Our statement that "the first two images in Fig. 2c appear similar to control" in the previous response to reviewers was in relation to overall crypt architecture, not specifically to ZFP36L2 expression. As the reviewer correctly points out, a claim of the extent of ZFP36L2 induction on day 2 does not help or hurt the manuscript's central arguments. We were simply reporting our observation, as

shown below from Figure 2c and Supplementary Data 1 and 3, that ZFP36L2 expression is increased in the crypts on day 2. Since the term "markedly increased" is indeed subjective, we will tone this down and simply report that "ZFP36L2 expression increased by day 2" (line 131), and can also remove this as per the Reviewer and Editor's guidance.

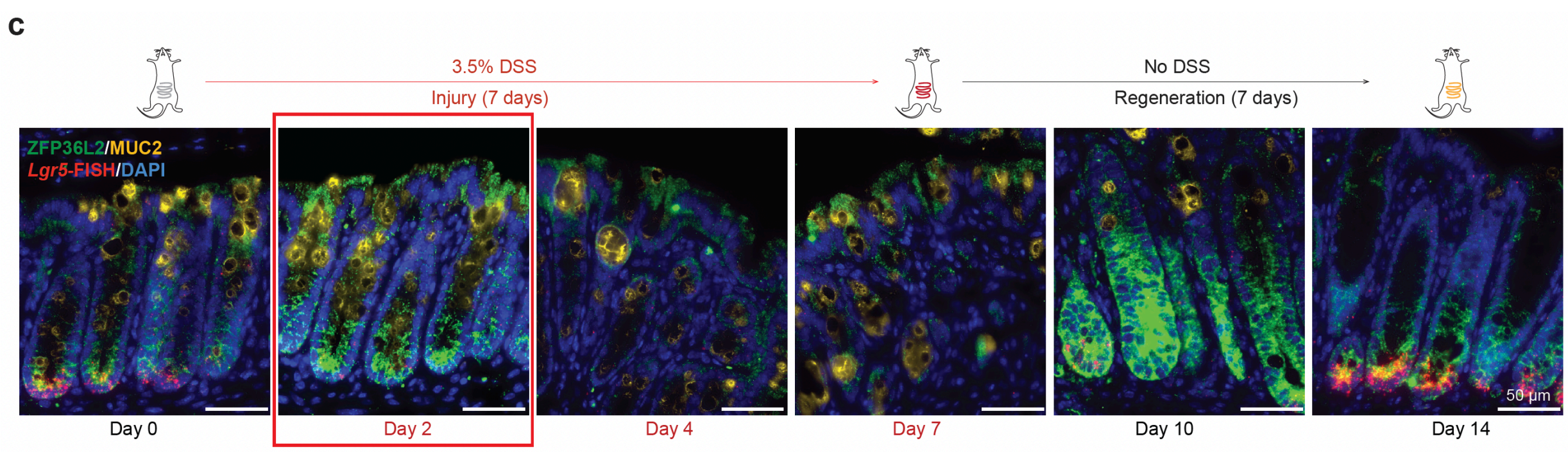

Fig. 2c. Representative images of *Lgr5*-FISH, ZFP36L2-IF, and MUC2-IF in colon tissue harvested at the indicated timepoints from mice provided 3.5% dextran sodium sulfate (DSS) in drinking water for 7 days followed by recovery for 7 days with DSS-free water. n=3 mice per time point.

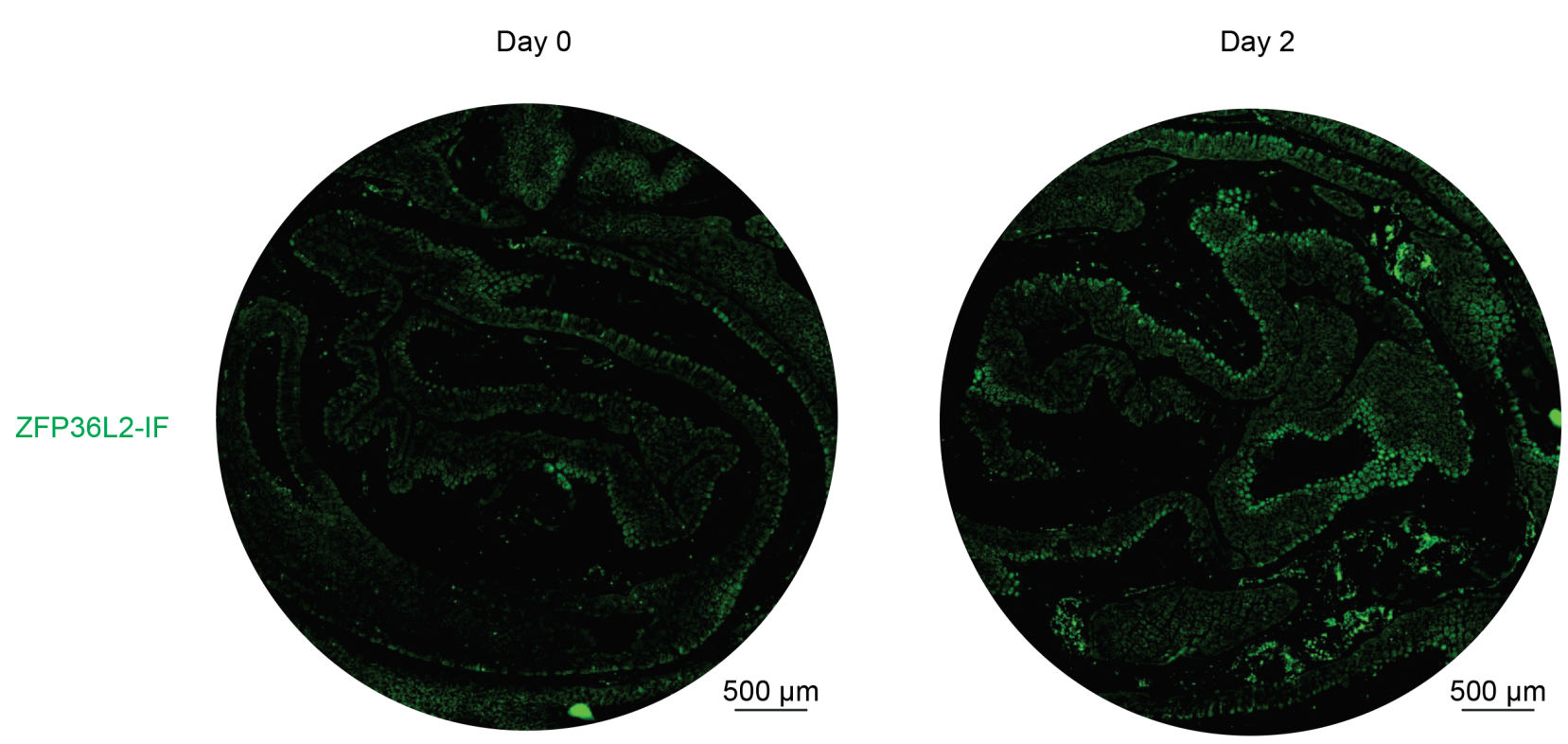

Supplementary Data 3. Expanded field of view of full colon swiss roll corresponding to Fig. 2c.

3. The manuscript stretches the idea that "CRC metastases progressively adopt a non-canonical differentiation trajectory" (line 226). … IF images don't seem to match "representative" numbers graphed to the right. The authors should provide large tissue areas as supplementary files. Also, please explain the meaning of negative values on the y-axes … likely reflect very small values in both cases. … It is unclear if "non-canonical" differentiation (which includes CHGB, a product of native ISC progeny) reflects something as specific as the authors assume and is not just the tip of a broadly aberrant iceberg.

I accept that CHGB and CK5 mark “non-canonical” differentiation, as the group’s previous work defined. Neither the rebuttal nor the revised manuscript address my concern about significance of ssGSEA score changes from 0.89 to 0.88 or from 0.36 to 0.38 (Fig. 3j), which could easily represent noise. CHGB+ cell fractions (25th to 75th quartile) in ZFP36L2-MUT metastases were <0.1% to 1% and in half the cases, CK5+ cell fractions were <0.01% (Fig. 3q-r). These numbers don’t make a strong case for the thrust of Fig. 3, which is that absence of ZFP36L2 favors “non-canonical differentiation”. This conclusion should be toned down or revised to match the data.

We appreciate the reviewer’s rigorous scrutiny of these data. We have addressed these concerns with a more robust, data-driven justification and revised text.

1. **ssGSEA analysis in Fig. 3j**: We agree that the absolute magnitude of the score changes is modest and that these data alone should not be overinterpreted. As we explained in our original Response to Reviewers, the data in Fig. 3j are bulk RNAseq generated from shControl or shZFP36L2 organoids cultured in HISC medium, a growth-factor-rich condition that strongly promotes ISC-like programs and therefore compresses transcriptional differences between conditions (see Moorman et al, 2025). While we agree that the absolute numerical shifts in ssGSEA scores appear small, these scores represent a rank-based enrichment across the entire transcriptomic profile of the sample. However, because of these limitations, we agree with the reviewer that this HISC medium organoid bulk RNAseq dataset in Fig. 3j should be replaced with more robust data. We therefore now remove Figure 3j and replace it with the RNAseq data generated from CRISPR-mediated ZFP36L2 knockout organoids under dedifferentiation conditions (**Extended Data Fig 7d, now incorporated into Fig.3j**), which better recapitulate the post-stress regenerative states of metastatic tumors *in vivo*. We now also break this down into individual non-canonical gene programs, osteoblast, squamous and neuroendocrine, and observe a consistent upregulation of these heterogeneous non-canonical programs in these organoids. We also now include volcano plots indicating the specific genes associate with these divergent non-canonical programs that are upregulated in the ZFP36L2 knockout condition. Our interpretation is that these scores indicate a *directional shift* in the population-wide transcriptional state toward plasticity rather than a wholesale lineage conversion. Importantly, the direction of this shift was reproducible across patient-derived organoid models and was further supported by orthogonal datasets, including scRNA-seq, CRISPR-mediated ZFP36L2 knockout under dedifferentiation conditions, PDO xenograft tumours, and ZFP36L2-mutant patient CRC liver metastases.

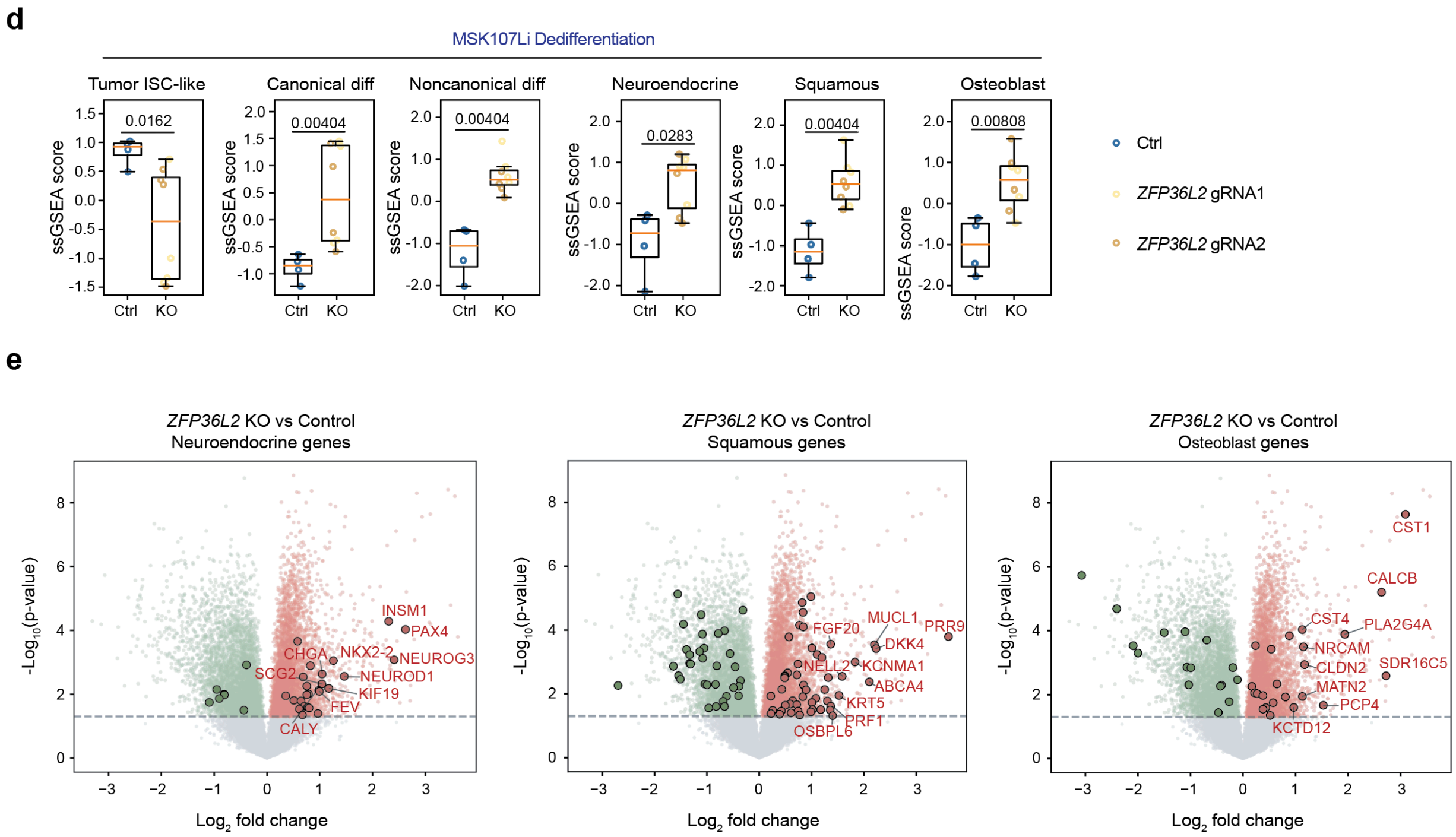

Extended Data Fig.7 d, ssGSEA scores for the indicated gene sets from bulk RNA sequencing of MSK107Li control and ZFP36L2 knockout organoid lines. Two independent clones from each gRNA were pooled. Organoid on day 7 of dedifferentiation were harvested for bulk RNA sequencing. Gene sets were the same as those used in Fig. 3l and Supplementary Table 2a, b. Box plots show the interquartile range (25th to 75th percentiles), horizontal line: median; whiskers: minimum and maximum values. Two-tailed Mann–Whitney U tests. e, Volcano plots showing differential gene expression from the bulk RNA-seq data shown in d. Genes from the indicated gene sets from Supplementary Table 2b are highlighted. The top ten upregulated genes from each gene set are labelled.

2. **Divergent plasticity into non-overlapping non-canonical states:** The Reviewer correctly points out that "CHGB+ cell fractions (25th to 75th quartile) in ZFP36L2-MUT metastases were <0.1% to 1% and in half the cases, CK5+ cell fractions were <0.01% (Fig. 3q-r)." The non-canonical states induced as a consequence of ZFP36L2 loss are themselves **heterogeneous**, encompassing squamous-like, neuroendocrine-like, osteoblast-like and potentially additional programs. The CHGB (neuroendocrine) and CK5 (squamous) staining shown are examples of non-canonical programs, but these do not

necessarily represent the full complement of non-canonical states in a given tumor or across patients. These programs emerge in non-overlapping cell clusters that are spatially heterogeneous and often appear in focal, patchy regions rather than being uniformly distributed across the tumour. Further, as we have described previously (Moorman et al., 2025), in any individual patient one or more of these divergent programs can dominate. Thus it is expected that within an individual tumor or region of tumor, one non-canonical program can be expressed while another is not expressed, i.e. a population of tumor cells could express CK5 but not CHGB, or neither marker but may express other non-canonical programs. This explains the low fraction of CK5+ cells in half the metastases. The overall conclusion from the patient data is that the ZFP36L2 mutation is correlated with a conserved decreased in the LGR5+ ISC state and non-canonical plasticity into a diversity of states, not that one specific non-canonical state is enriched. The representative images were selected to show the morphology and staining pattern of positive regions, whereas the quantification was performed **across whole tumour tissue sections across all ZFP36L2-mutant tissue rather than selectively over marker-positive regions**. We have also included larger fields of view showing the focal nature of the staining in Supplementary Data 6. To clarify this important point, we now include additional large field-of-view immunostaining showing serial sections from the same region of individual patient metastases, which show that within individual patient metastases, ZFP36L2 mutation leads to loss of LGR5-FISH staining, but is associated with expression of *either* CK5 or CHGB in parts of the tumor, but not both. These data are now incorporated into the manuscript as **Extended Data Fig. 8.**

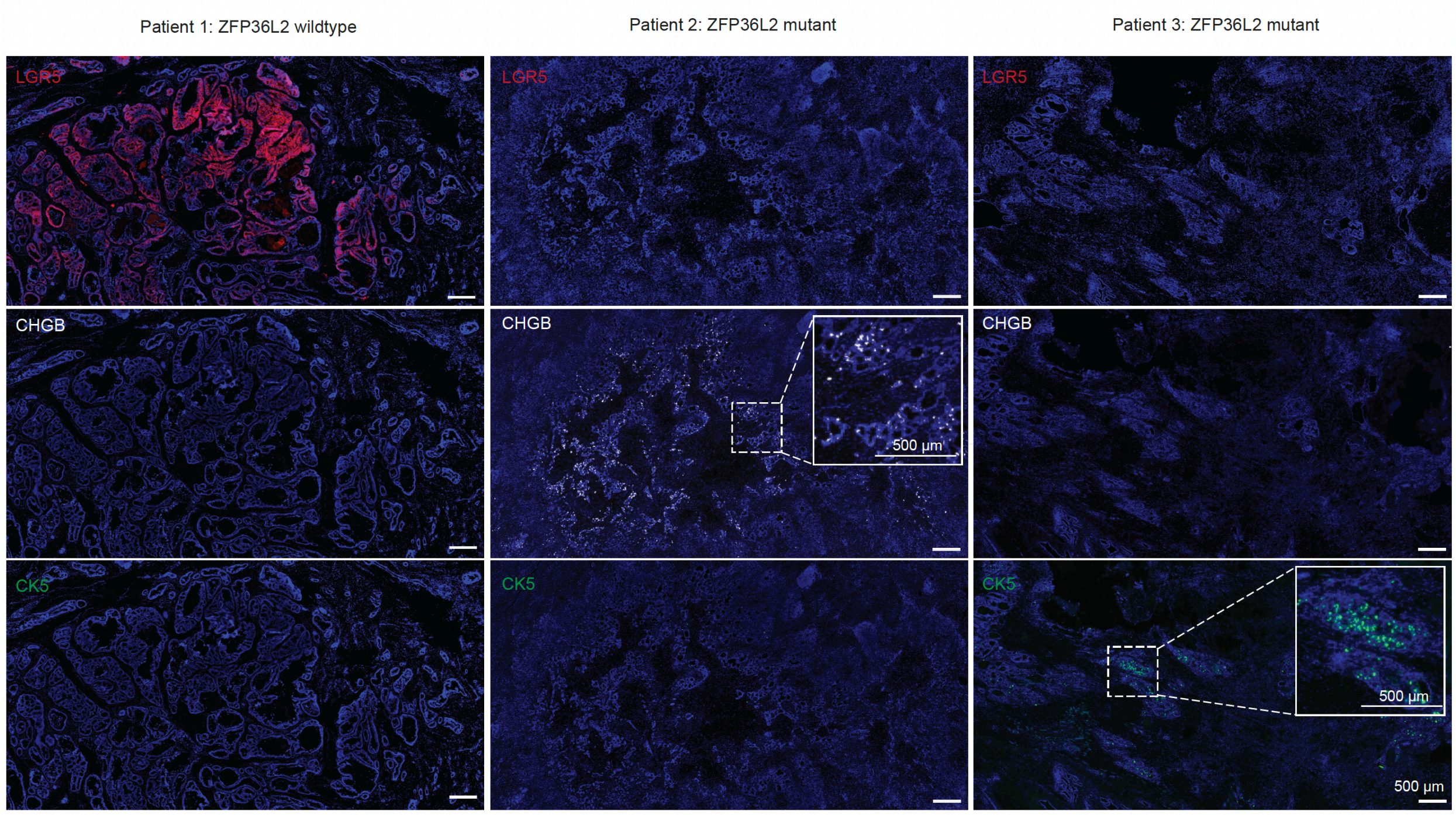

Extended Data Fig. 8 | Non-canonical differentiation in patient CRC metastases with loss of function ZFP36L2 mutations.
Representative images of LGR5-FISH or IF staining for CK5 (noncanonical squamous differentiation marker) or CHGB (noncanonical neuroendocrine differentiation marker) in patient CRC metastases with wildtype (WT) or mutant (MUT) ZFP36L2, showing loss of LGR5-FISH staining and divergent gain of CHGB or CK5 staining in focal areas of metastases from different patients.

We have also clarified the text as follows (lines 314-322):

> Within individual *ZFP36L2* mutant tumours and across patients, clusters of cells expressed either CK5 or CHGB, but not both markers (**Extended Data Fig. 8**), consistent with the loss of ZFP36L2 promoting a shift towards a more plastic state permissive for divergent differentiation into non-canonical states.

3. **Heterogeneity of non-canonical states:** We further note that CHGB and CK5 are only two markers of non-canonical differentiation and <u>do not capture the full diversity of non-canonical tumour cell states.</u> <u>**At the broader transcriptional level**</u>, human CRC epithelial scRNA-seq analyses show that ZFP36L2 expression is relatively low in osteoblast, squamous and neuroendocrine non-canonical programs, and that ZFP36L2 expression is

negatively correlated with osteoblast and neuroendocrine gene programs (*Extended Data Fig. 1d,g*). These analyses support the interpretation that ZFP36L2 expression is inversely associated with non-canonical differentiation programs at the tumour epithelial cell-state level.

Extended Data Fig. 1

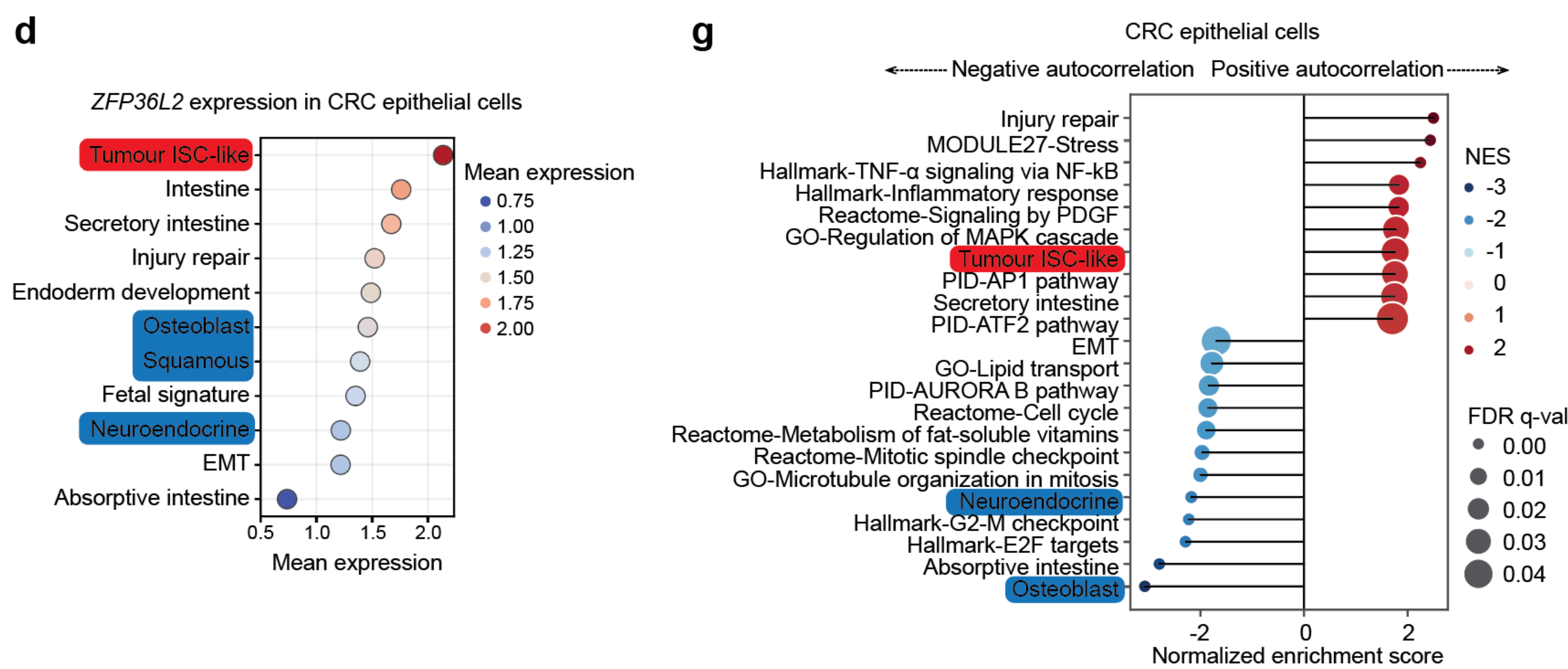

Extended Data Fig. 1d,g, Gene set enrichment analysis (GSEA) of genes ranked by autocorrelation with *ZFP36L2*, identifying pathways enriched in normal colon epithelial cells (left) and CRC epithelial cells (right) FDR<0.05. Full genesets and references are provided in **Supplementary Table 1b**.

The manuscript states that ZFP36L2 loss is associated with increased permissiveness toward spatially heterogeneous non-canonical differentiation programs, which we believe is rigorously supported by convergent evidence from ssGSEA, scRNA-seq, ZFP36L2 knockout organoids, PDO xenografts and ZFP36L2-mutant patient metastases.

4-5. The point that the RNA-binding protein ZFP36L2 participates, along with RNA molecules, in phase-separated cytoplasmic condensates appears valid, and videos from live cell imaging are clear. … but the link between condensate formation and dedifferentiation is weak. … Some data are noisy …[redacted].

The authors' use of drift correction and quantitative tracking adds the desired level of rigor to describe the condensates as "dynamic". The temporal association of ZFP36L2 condensates with

dedifferentiation is strengthened. Cut-off thresholds are made consistent, SLAM-seq is used to corroborate findings, [redacted] These changes improve the study, as do new data that localize putative ZFP36L2 target mRNAs in ZFP36L2-containing condensates (Fig. 5g-h).

We thank their reviewer for their feedback and positive appraisal of our revisions, which have improved the rigour and robustness of this section of the manuscript.

[redacted]

[redacted] We agree that the core strength of the study in its current form lies in our discovery of the role of ZFP36L2 as a regulator of stress-adaptive mRNA decay through condensate formation.

We have retained the co-immunostaining of ZFP36L2 with P body and stress granule markers (**Extended Data Figure 12 d,e**) and moved these data and the associated text to **Extended Data Figure 10**. We believe this will be of interest to the condensate field to characterize the relationship between ZFP36L2 granules and other known stress-related condensates.

Referee #3 (Remarks to the Author):

In the revised manuscript the authors have addressed all my questions and concerns very nicely and I support publication of this excellent study. I have one small comments on the newly added experiments and one on the text. I don't think the experiment is absolutely essential, but it would be nice to add.

Fig. 5g,h It would be nice to see a control mRNA that is not associated with ZFP36L2 in these experiments. I was a bit confused by the explanation of these experiments (lines 386-390): **co-localization of target mRNAs is observed strongly in the unstressed condition, but then**

severely reduced during stress. The authors state that stress induces ZFP36L2 expression, which binds to target mRNAs and induces their degradation. However, if this were true, would one not expect the target mRNAs to enrich to ZFP36L2 foci during stress? Are the authors thinking that the co-localization during stress is reduced, because the target mRNAs are degraded by ZFP36L2 foci during stress? This text could be clarified a bit.

Reviewed by Marvin Tanenbaum

We thank the Reviewer for the very positive assessment of our revised manuscript and for the helpful suggestions regarding the newly added RNA-FISH experiments.

We agree that inclusion of a control mRNA not associated with ZFP36L2 would be informative and could be done.

Regarding the colocalization of ZFP36L2 during stress, the reviewer's interpretation is exactly what we propose. At baseline in HISC conditions, ZFP36L2 can be seen interacting with target mRNAs (*GDF15*, *GADD45A*, *HILPDA*) without immediate large-scale degradation. Upon stress induction, ZFP36L2 expression increases and condensate formation is enhanced, leading to the rapid sequestration and subsequent degradation of these mRNAs. The reduction in co-localization during peak stress likely reflects the kinetics of active degradation—the targets are being cleared as quickly as they are recruited.

In the manuscript, we had quantified target mRNA colocalization per ZFP36L2 punctum, which we agree could be interpreted in either way as above. To distinguish between mRNA distribution vs abundance, we now also add images and quantification of the overall intensity of staining of ZFP36L2 IF and target mRNA per punctum (**Fig. 5i,j**).

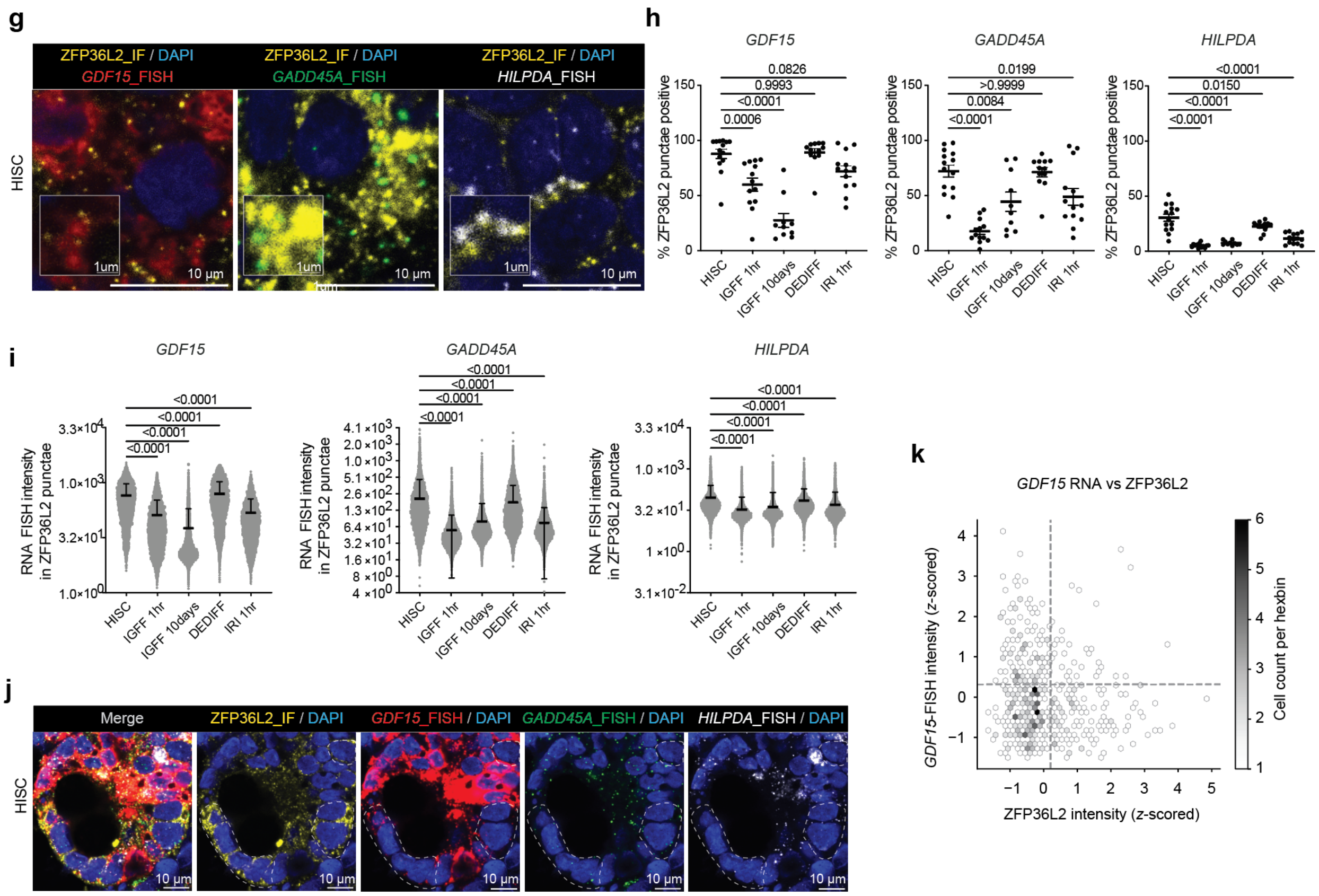

Fig. 5g-k. g, Representative images showing colocalization of ZFP36L2 protein with the indicated target RNAs, assessed by ZFP36L2-IF together with RNA FISH for *GDF15*, *GADD45A*, and HILPDA in OKG146Li-MS2 organoids cultured in HISC medium. **h-i**, Quantification of the percentage of ZFP36L2 puncta positive for the indicated RNA FISH signal (**h**) and target mRNA FISH intensity within ZFP36L2 puncta (**i**) in OKG146Li-MS2 organoids. Prior to fixation, staining and imaging, organoids were cultured in HISC (HISC) media for 7 days, cultured in IGFF media for 10 days prior to switching to HISC media for 7 days to induce dedifferentiation (Dediff), or cultured in HISC media for 7 days prior to switching to IGFF (IGFF 1 hr) or HISC containing 500 μmol irinotecan (IRI 1 hr) for 1 h. Each dot represents one organoid. n = 10-14 organoids per condition. Data are mean ± s.e.m. Two-tailed Mann–Whitney *U* test. **j-k**, Representative images (**j**) and quantification (**k**) of inverse correlation between ZFP36L2 IF and target mRNA (*GDF15*) FISH staining intensity.

Quantified on a per cell basis, we also find that there is an inverse correlation between the intensity of ZFP36L2 and target mRNA staining (**Fig.5j-k**), consistent with our interpretation that increased ZFP36L2 puncta formation correlates with mRNA degradation. We have added the intensity quantification to the manuscript and clarified this "buffering and clearing" model in the revised Results section (lines 449-452). In unstressed HISC conditions, target mRNAs are readily detected and show robust co-localization with ZFP36L2 foci.

We then asked whether the spatiotemporal dynamics of these transcripts in ZFP36L2 condensates varied during stress responses (**Fig. 5h**). Acute exposure to growth factor withdrawal or irinotecan– conditions that induce ZFP36L2 condensate formation (**Fig. 4f,g; Extended Data Fig. 8c-h**), decreased the percentage of ZFP36L2 foci positive for target mRNA expression as well as the intensity of mRNA staining within the ZFP36L2 foci (**Fig. 5g-h**). This reduction in mRNA abundance and localization in ZFP36L2 condensates became even more pronounced following long-term culture in growth factor free (IGFF) media for 10 days. Passaging organoids that had undergone long-term IGFF culture back into growth factor rich HISC medium, and thus alleviating mitogen deprivation-associated stress and inducing dedifferentiation (see **Fig. 2,3**), induced recovery of the mRNA/ZFP36L2 colocalization (**Fig. 5h,i**). On a per cell level, we observed an inverse correlation between the intensity of ZFP36L2 and target mRNA staining (**Fig. 5j,k**). These data support a model in which stress-induced ZFP36L2 foci engage and promote rapid degradation of target mRNAs, thereby reducing their steady-state abundance and apparent co-localization during stress. Together, these observations spatiotemporally link ZFP36L2 condensate dynamics with its stress-response mRNA targets: expression and colocalization is detectable at baseline, decreases during acute stress consistent with dynamic mRNA degradation within ZFP36L2 condensates, reaches a minimum under prolonged growth factor stress conditions, is reversible upon reintroduction of growth factors and is temporally coupled with dedifferentiation into an Lgr5+. ISC state.

This additional analysis directly addresses the Reviewer's concern and supports the interpretation that reduced target mRNA co-localization during stress reflects ZFP36L2-dependent target mRNA degradation rather than loss of target specificity.
