# Supplementary material for: ZFP36L2 orchestrates stress-adaptive plasticity in regeneration and cancer
Source: Nature. 2026 Aug 5;657(8133):1053–63. doi: 10.1038/s41586-026-10890-0 (PMC13600930; doi:10.1038/s41586-026-10890-0)
Supplement: Supplementary file 4 [file 41586_2026_10890_MOESM8_ESM.zip › Supplementary videos/Supp. Video. 2. MSK107Li ZFP36L2-GFP_Condensate expression post dissociation/Supp. Video 2. Legend.docx]

**Supplementary Video 2 | Live imaging tracking ZFP36L2-GFP condensate expression post dissociation to single-cell state.**

Representative live confocal imaging videos of wt ZFP36L2-GFP expressing MSK107Li organoids post dissociation to single cells. Organoids were imaged for 64 hours at 30-minute intervals at 63X, with Z-projection of the whole organoid.
