# Supplementary material for: ZFP36L2 orchestrates stress-adaptive plasticity in regeneration and cancer
Source: Nature. 2026 Aug 5;657(8133):1053–63. doi: 10.1038/s41586-026-10890-0 (PMC13600930; doi:10.1038/s41586-026-10890-0)
Supplement: Supplementary file 4 [file 41586_2026_10890_MOESM8_ESM.zip › Supplementary videos/Supp. Video. 3. FRAP videos for MSK107Li, OKG146P and OKG146Li/Supp. Video 3. Legend.docx]

**Supplementary Video 3 | Fluorescence Recovery After Photobleaching (FRAP) in wt ZFP36L2-GFP, fsZFP36L2-GFP and GFP organoids.**

Representative time-lapse videos of FRAP experiments performed on wt ZFP36L2-GFP, fsZFP36L2-GFP and GFP organoids in **a,** MSK107Li, **b,** OKG146P, and **c,** OKG146Li.
