# Supplementary material for: ZFP36L2 orchestrates stress-adaptive plasticity in regeneration and cancer
Source: Nature. 2026 Aug 5;657(8133):1053–63. doi: 10.1038/s41586-026-10890-0 (PMC13600930; doi:10.1038/s41586-026-10890-0)
Supplement: Supplementary file 4 [file 41586_2026_10890_MOESM8_ESM.zip › Supplementary videos/Supp. Video. 1. MSK107, OKG 146P and OKG 146Li GFP, ZFP36L2-GFP, fsZFP36L2-GFP_H2B mCherry constructs/Supp. Video 1. Legend.docx]

**Supplementary Video 1 | Live imaging of day 3 nuclear H2B-mCherry tagged wt ZFP36L2-GFP, fsZFP36L2-GFP and GFP organoids.**

Representative live confocal imaging videos of day 3 H2B-mCherry tagged wt ZFP36L2-GFP, fsZFP36L2-GFP and GFP organoids in **a,** MSK107Li, **b,** OKG146P, and **c,** OKG146Li. Organoids were imaged for 15 hours at 30-minute intervals at 63X, with Z-projection of the whole organoid.
